# Development of microflow ultra high performance liquid chromatography-mass spectrometry metabolomic assays for analysis of mammalian biofluids

**DOI:** 10.1007/s11306-024-02187-y

**Published:** 2024-10-25

**Authors:** Annie J. Harwood-Stamper, Caroline A. Rowland, Warwick B. Dunn

**Affiliations:** 1 School of Biosciences, University of Birmingham, Edgbaston, Birmingham, B15 2TT UK; 2 Defence Science and Technology Laboratory, Porton Down, Salisbury, SP4 0JQ UK; 3 Centre for Metabolomics Research, Department of Biochemistry, Cell and Systems Biology, Institute of Systems, Molecular and Integrative Biology, University of Liverpool, Biosciences Building, Crown Street, Liverpool, L69 7ZB UK

**Keywords:** Metabolomics, UHPLC-MS, Microflow liquid chromatography, Plasma, Urine, Tears

## Abstract

**Introduction and objectives:**

The application of untargeted metabolomics assays using ultra high performance liquid chromatography-mass spectrometry (UHPLC-MS) to study metabolism in biological systems including humans is rapidly increasing. In some of these studies there is a requirement to collect and analyse low sample volumes of biofluids (e.g. tear fluid) or low cell and tissue mass samples (e.g. tissue needle biopsies). The application of microflow, capillary or nano liquid chromatography (≤ 1.0 mm column internal diameter (i.d.)) theoretically should accomplish a higher assay sensitivity compared to analytical liquid chromatography (2.1–5.0 mm column internal diameter). To date, there has been limited research into microflow UHPLC-MS assays that can be applied to study samples of low volume or mass.

**Methods:**

This paper presents three complementary UHPLC-MS assays (aqueous C_18_ reversed-phase, lipidomics C_18_ reversed-phase and Hydrophilic Interaction Liquid Chromatography (HILIC)) applying 1.0 mm internal diameter columns for untargeted metabolomics. Human plasma and urine samples were applied for the method development, with porcine plasma, urine and tear fluid used for method assessment. Data were collected and compared for columns of the same length, stationary phase and stationary phase particle size but with two different column internal diameters (2.1 mm and 1.0 mm).

**Results and conclusions:**

All three assays showed an increase in peak areas and peak widths when applying the 1.0 mm i.d. assays. HILIC assays provide an advantage at lower sample dilutions whereas for reversed phase (RP) assays there was no benefit added. This can be seen in the validation study where a much higher number of compounds were detected in the HILIC assay. RP assays were still appropriate for small volume samples with hundreds of compounds being detected. In summary, the 1.0 mm i.d. column assays are applicable for small volume samples where dilution is required during sample preparation.

**Supplementary Information:**

The online version contains supplementary material available at 10.1007/s11306-024-02187-y.

## Introduction

Within metabolomic studies involving mammals, including humans, there is a vast range of sample types that are applied. These sample types are analysed using numerous different analytical platforms as no single platform would allow detection and quantification of an entire metabolome. Serum, plasma, and urine are the most commonly studied human-derived samples. The volume for these samples is typically not limited and therefore assay sensitivity is not normally a concern. However, some samples are limited in volume or mass and therefore the assay sensitivity for untargeted assays have to be considered with tear fluid (5–10µL) (Lam et al., 2014), tissue biopsies (1-5 mg) (Gehmlich et al., 2015) and cell 3D spheroids (< 10^4^ cells) (Rusz et al., 2019) being three examples. The collection of biological samples of low volume or mass can provide a number of advantages including less invasive sampling (e.g. dried blood spot collection (5–20µL collected) instead of venous blood collection (3-10mL collected) or the ability to collect samples which are biologically limited in their volume or mass (e.g. tear fluid).

For untargeted metabolomics studies of low mass or volume samples, the development of more sensitive analytical assays to allow the reproducible detection of the same number of metabolites as detected using larger volume/mass samples is required. Although technically difficult for low volume or mass samples, this can be achieved through pre-concentration of metabolites during sample collection and preparation. Another strategy that can be applied is the use of liquid chromatography (LC) columns of smaller internal diameters compared to the analytical LC columns of 2.1 mm internal diameter which are most routinely applied in metabolomics studies. Ultra High Performance Liquid Chromatography-Mass Spectrometry (UHPLC-MS) is the most frequently applied analytical platform for untargeted metabolomics applications. UHPLC has many advantages over the more traditionally applied HPLC as a result of using smaller diameter stationary phase particles (< 2.5 μm compared to 3–5 μm) and higher flow rates/velocities. Using smaller particles and higher flow velocities helps to reduce eddy diffusion which subsequently reduces peak broadening, leading to a higher chromatographic resolution and a potentially higher sensitivity (Wu et al., 2006; Plumb et al., 2004). Different stationary phases can be applied in a complimentary manner to increase the number of metabolites detected; for example, HILIC and C_18_ reversed-phase assays allow detection of water-soluble and lipid metabolites, respectively. (UHP)LC-MS uses four different classes of column based on their internal diameter: standard/analytical (≥ 2.1 mm i.d.), microbore (1.0–2.1 mm i.d.), capillary (0.1–1 mm i.d.) and nano (0.075 μm-0.1 mm i.d.) (Chen et al., 2007; Rozing, 2003).

The use of microbore columns instead of analytical columns can also, theoretically, increase sensitivity. By decreasing the column i.d. and maintaining the same flow velocity, an increased sensitivity is typically observed in cases where the injection volume is not too high so as to cause column overloading (Hopfgartner et al., 1993). However, a lower flow velocity for a smaller i.d. column, when compared to a larger i.d. column, can result in greater eddy diffusion and reduce or negate any increase in sensitivity. The use of lower flow velocities can also impact on peak broadening in the LC or electrospray ionisation source if appropriate i.d. tubing and a minimisation of dead volumes is not considered (Gray et al., 2015).

In previous research, it has been shown that the use of 1.0 mm i.d. UHPLC columns showed promise in providing assays of appropriate sensitivity and reproducibility. Studies describing the use of a 1.0 mm i.d. column in metabolomics research include the investigation of *E.coli* (Yanes et al., 2011), dystrophic retinas in rat models (Wang et al., 2016), dried blood spots collected from mice (Rahavendran et al., 2012), rat brain tissue (Lanckmans et al., 2006) and urine (Esmans et al., 1985). Only a small number of studies have been reported which perform a direct comparison between 2.1 mm and 1.0 mm i.d. columns for untargeted metabolomics assays and these have primarily focused on reversed phase assay (Gray et al., 2015; Fitz et al., 2022; Geller et al., 2022; King et al., 2019).

The purpose of the study reported here was to develop three complementary UHPLC assays applying 1.0 mm i.d. UHPLC columns and to subsequently compare their applicability to analytical UHPLC assays applying 2.1 mm i.d. columns with the same stationary phase type and particle size. Two commonly analysed mammalian biofluids (plasma and urine) were applied for assay development and characterisation whilst porcine plasma, urine and tear fluid samples were applied to assess the applicability of 1.0 mm i.d. columns for untargeted metabolomics analysis of low volume (< 5µL) samples.

## Materials and methods

### Materials

Methanol, water, isopropanol (IPA), acetonitrile and chloroform (HPLC grade) were purchased from VWR International (Loughborough, UK). Formic acid (HPLC grade) and ammonium formate (99%+ purity) were purchased from Sigma-Aldrich (Poole, UK). 5mL volumes of human urine and plasma from one male donor were purchased from BioIVT (Sussex, UK). These were delivered frozen, thawed for aliquoting, and were then frozen again at -80^o^C before being thawed for analysis, giving a total of two freeze/thaw cycles. Two millilitre porcine urine samples, 1mL porcine plasma samples and ~ 20µL porcine basal tear fluid samples were all collected across 13 hourly time points by the Defence Science and Technology Laboratory (Porton Down, UK). These porcine samples were obtained from non-recovery anaesthetised animals within a pre-existing programme of animal work carried out by Dstl in accordance with the UK Animals (Scientific Procedures) Act, 1986. Samples were delivered frozen and only thawed for analysis, giving a total of one freeze/thaw cycle.

### Sample extraction

Sample extraction applied three different solutions; ice-cold 50/50 (v/v) methanol/water (for the aqueous C_18_ reversed-phase assay), ice cold IPA (for the lipidomics C_18_ reversed-phase assay) and ice cold 3:1 acetonitrile/methanol (for the HILIC assay). For human and porcine plasma samples, protein precipitation was performed by mixing 50µL of plasma with 150µL of extraction solution followed by vortex mixing (30 s) and centrifugation (21,885 *xg*, 20 min, 4^o^C). For all neat human and porcine urine samples, dilution was performed by mixing 50µL of urine with 150µL of extraction solution followed by vortex mixing (30 s) and centrifugation (21,885 *xg*, 20 min, 4^o^C). The supernatant from the human plasma and human urine extraction solutions were then serially diluted (see Supplementary Information S1) with extraction solvent to prepare the following dilutions (1 in 4, 1 in 8, 1 in 16, 1 in 32, 1 in 64 and 1 in 128). Each new dilution was created using the next highest dilution sample (for example, the 1 in 16 dilution solution was prepared from the 1 in 8 dilution solution). All diluted sample solutions were vortex mixed (30 s) followed by centrifugation (4000 *xg*, 20 min, 4^o^C). For each assay two extraction replicates were prepared at each dilution and four technical replicates were injected (two from each extraction replicate).

For the porcine tear fluid samples, extraction was carried out using a biphasic liquid - liquid extraction method, where a chloroform/methanol/water 2:2:1.8 (v/v/v) solvent system was used to separate polar metabolites from lipid metabolites whilst also precipitating out proteins (Wu et al., 2008). Each tear fluid was weighed out, using the assumption that the density of tear fluid was equal to that of water; 1 g.mL^− 1^. Once the samples had thawed on ice, 8 µL.mg^− 1^ tear fluid of cold methanol was added to the sample and vortexed. Samples were then transferred to 1.8mL vials where 8 µL.mg^− 1^ tear fluid of chloroform and 4 µL.mg^− 1^ tear fluid of water were added. The samples were then vortexed for 30s and then sat at room temperature for 10 min. Samples were then centrifuged (2500 *xg*, 4^o^C) for 10 min. Following this, samples were left at room temperature for a further 5 min before removal of both the polar and lipid phases. Both phases were dried down under a stream of nitrogen gas and stored at -80^o^C, before being re-suspended in 40µL of solvent on the day of analysis.

### UHPLC-MS analysis

For data acquisition, a Vanquish™ Horizon UHPLC System was coupled to a Q Exactive Plus mass spectrometer (Thermo Fisher Scientific, Bremen, Germany). All data were collected separately in positive and negative ion modes, with data on the human samples collected first and the porcine data collected later. Mass calibration was performed in both ion modes applying the Pierce™ LTQ Velos ESI Positive Ion Calibration Solution and Pierce™ Negative Ion Calibration Solution to carry out the recommended manufacturer evaluation and calibration tests. QC samples were prepared using 1 in 4 dilutions made from each sample type separately. Ten QC samples were injected at the start of the run for column equilibration and then a further five were injected at the start of each porcine sample type for metabolite identification through MS/MS data acquisition. QC samples were also injected every 6th injection to ensure that the instrument response wasn’t drifting throughout the run. Solvent blank samples were injected after the 5th QC sample applied for column equilibration and at the end of each batch.

### C_18_ reversed-phase lipidomics (lipids) assay

Human plasma samples were analysed in both positive and negative ion mode on both i.d. columns. Porcine plasma and tear samples were analysed on the 1.0 mm i.d. column using both positive and negative ion mode. The columns used were a Waters^®^ ACQUITY UPLC^®^ BEH C_18_ (130Å, 1.7 μm, 2.1 mm x 100 mm) and a Waters^®^ ACQUITY UPLC^®^ BEH C_18_ (130Å, 1.7 μm, 1.0 mm x 100 mm).

For the 2.1 mm i.d. column, solvent A was 10 mM ammonium formate in 60/40 acetonitrile/water (v/v) + 0.1% formic and solvent B was 10 mm ammonium formate in 90/10 propan-2-ol/acetonitrile (v/v) + 0.1% formic acid. The gradient elution used was 0 to 1 min 99%A; 1 to 3 min 85%A with a curve = 5; 3 to 6 min 50%A with a curve = 5, 6 to 9 min; 5%A with a curve = 5, 9 to 10 min; 5%A, 10 to 10.5 min; 99%A with a curve = 5, 10.5 to 14 min; 99%A. The UHPLC parameters were flow rate = 0.40 mL.min^− 1^, column temperature = 55 °C and injection volume = 2µL. Mass spectrometer settings were scan range = 150–2000 *m/z*, sheath gas flow rate = 48; aux gas flow rate = 0, spray voltage (kV) positive ion mode = 3.2 and negative ion mode = 2.7, capillary temp = 350 °C; S-lens RF level = 60 and aux gas heater temperature = 400 °C. A high flow electrospray needle was used.

For the 1.0 mm i.d. column, solvent A was 10 mM ammonium formate in 60/40 acetonitrile/water (v/v) + 0.1% formic and solvent B was 10 mm ammonium formate in 90/10 propan-2-ol/acetonitrile (v/v) + 0.1% formic acid. The gradient elution used was 0 to 1 min 80%A; 1 to 10 min 0%A with a curve = 5; 10 to 14 min 0%A; 14 to 14.5 min 80%A with a curve = 5; 14.5 to 25 min 80%A. The UHPLC paramters were flow rate = 0.12 mL.min^− 1^, column temperature = 55 °C and injection volume = 2µL. Mass spectrometer settings were a scan range = 150–2000 *m/z*, sheath gas flow rate = 32 (positive ion mode) and 48 (negative ion mode), aux gas flow rate = 10 (positive ion mode) and 11 (negative ion mode), sweep gas flow rate = 1 (positive ion mode) and 2 (negative ion mode), spray voltage (kV) = 3.2 (positive ion mode) and 2.5 (negative ion mode), S-lens RF level of 50 and aux gas heater temp = 168 °C (positive ion mode) and 413 °C (negative ion mode). A high flow electrospray needle was used.

### C_18_ aqueous reversed-phase (RPaq) assay

Human urine samples were analysed in both positive and negative ion mode on both i.d. columns. Porcine urine and tear samples were analysed on the 1.0 mm i.d. column in both positive and negative ion mode. The columns used were a Thermo Scientific™ Hypersil GOLD™ aQ C_18_ (175 Å, 1.9 μm, 2.1 mm x 100 mm) and Thermo Scientific™ Hypersil GOLD™ aQ C_18_ (175 Å, 1.9 μm, 1.0 mm x 100 mm).

For the 2.1 mm i.d. column, solvent A was water/formic acid (99.9/0.1), and solvent B was acetonitrile/formic acid (99.9/0.1). The gradient used was 0 to 0.5 min 99%A; 0.5 to 2 min 50%A with a curve = 5; 2 to 10.5 min 1%A with a curve = 5; 10.5 to 11 min 99%A with a curve = 8; 11-11.5 min 99%A; 11.5–15.0 min 99%A. The UHPLC paramters were: flow rate = 0.30 mL.min^− 1^ for 0–11 min and 14.9–15.0 min and 0.40 mL.min^− 1^ for 11-14.9 min. The column temperature was 40 °C and the injection volume was 2µL. Mass spectrometer settings were scan range = 100–1500 *m/z*, sheath gas flow rate of 30, aux gas flow rate = 13, sweep gas flow rate = 0, spray voltage (kV) = 3.2 (positive ion mode) and 2.7 (negative ion mode), capillary temperature = 350 °C, S-lens RF level = 40 and aux gas heater temperature = 400 °C. A high flow electrospray needle was applied.

For the 1.0 mm i.d. column, solvent A was water/formic acid (99.9/0.1) and solvent B was acetonitrile/formic acid (99.9/0.1). The gradient used was 0 to 2 min 99%A; 2 to 7 min 50%A with a curve = 5; 7 to 10.5 min 1%A with a curve = 5; 10.5 to 11 min 1%A; 11-11.5 min 99%A with a curve = 5; 11.5–15.0 min 99%A. The flow rate was 0.13 mL.min^− 1^ = 0–11 min and 14.0–15 min and was 0.135 mL.min^− 1^ = 11-14.9 min. The column temperature was 40 °C and the injection volume was 2µL. Mass spectrometer settings were scan range = 100–1500 *m/z*, sheath gas flow rate = 30, aux gas flow rate = 13, sweep gas flow rate = 0, spray voltage (kV) = 3.2 (positive ion mode) and 2.7 (negative ion mode), capillary temp = 350 °C, S-lens RF level = 40 and aux gas heater temperature = 400 °C. A high flow electrospray needle was used.

### Hydrophilic interaction liquid chromatography (HILIC) assay

Huma plasma and urine samples were analysed in both positive and negative ion mode on both i.d. columns. Porcine plasma, urine and tear samples were analysed on the 1.0 mm i.d. column in both positive and negative ion mode. The columns used were a Waters^®^ ACQUITY UPLC^®^ BEH HILIC (130Å, 1.7 μm, 2.1 mm x 100 mm) and a Waters^®^ ACQUITY UPLC^®^ BEH HILIC (130Å, 1.7 μm, 1.0 mm x 100 mm).

For the 2.1 mm i.d. column, Positive ion mode solvent A was 10 mM ammonium formate in 95/5 acetonitrile/water (v/v) + 0.1% formic acid; solvent B was 10 mM ammonium formate in 50/50 acetonitrile/water (v/v) + 0.1% formic acid. Negative ion mode solvent A was 10 mM ammonium acetate in 95/5 acetonitrile/water (v/v) + 0.1% acetic acid; solvent B was 10 mM ammonium acetate in 50/50 acetonitrile/water (v/v) + 0.1% acetic acid. The gradient used was 0 to 0.5 min 99%A; 0.5 to 2 min 50%A with a curve = 5; 2 to 10.5 min 1%A with a curve = 5; 10.5 to 11 min 99%A with a curve = 8; 11-11.5 min 99%A; 11.5–15.0 min 99%A. The UHPLC paramters were: flow rate was 0.50 mL.min^− 1^ for 0–11 min and 14.9–15.0 min and 0.40 mL.min^− 1^ for 11-14.9 min. The column temperature was 40 °C and the injection volume was 2µL. Mass spectrometer settings were scan range = 100–1500 *m/z*, sheath gas flow rate = 30, aux gas flow rate = 13, sweep gas flow rate = 0, spray voltage (kV) = 3.2 (positive ion mode) and 2.7 (negative ion mode), capillary temperature = 350 °C, S-lens RF level = 40 and aux gas heater temperature = 400 °C. A high flow electrospray needle was applied.

For the 1.0 mm i.d. column, Positive ion mode solvent A was 10 mM ammonium formate in 95/5 acetonitrile/water (v/v) + 0.1% formic acid; solvent B was 10 mM ammonium formate in 50/50 acetonitrile/water (v/v) + 0.1% formic acid. Negative ion mode solvent A was 10 mM ammonium acetate in 95/5 acetonitrile/water (v/v) + 0.1% acetic acid; solvent B was 10 mM ammonium acetate in 50/50 acetonitrile/water (v/v) + 0.1% acetic acid. The gradient used was 0 to 1 min 99%A; 1 to 3 min 80%A with a curve = 3; 3 to 5 min 80%A; 5 to 5.5 min 75%A with a curve = 5; 5.5–9.5 min 5%A with a curve = 5; 9.5–10.0 min 5%A; 10-10.5 min 99%A with a curve of 5; 10.5–14 min 99%A with a curve of 5. The flow rate was 0.175 mL.min^− 1^ for 0-10.5 min and 13.9–14 min and was 0.225 mL.min^− 1^ for 10.5–13.9 min. The column temperature was 40 °C and the injection volume was 2µL. Mass spectrometer settings were scan range = 70-1050 *m/z*, sheath gas flow rate = 10, aux gas flow rate = 5, sweep gas flow rate = 2, spray voltage (kV) = 3.2 (positive ion mode) and 2.7 (negative ion mode), capillary temp = 320 °C, S-lens RF level = 30 and aux gas heater temperature = 440 °C. A high flow electrospray needle was used.

### Raw data processing and metabolite annotation

#### Human sample data

**Fig. 1:**
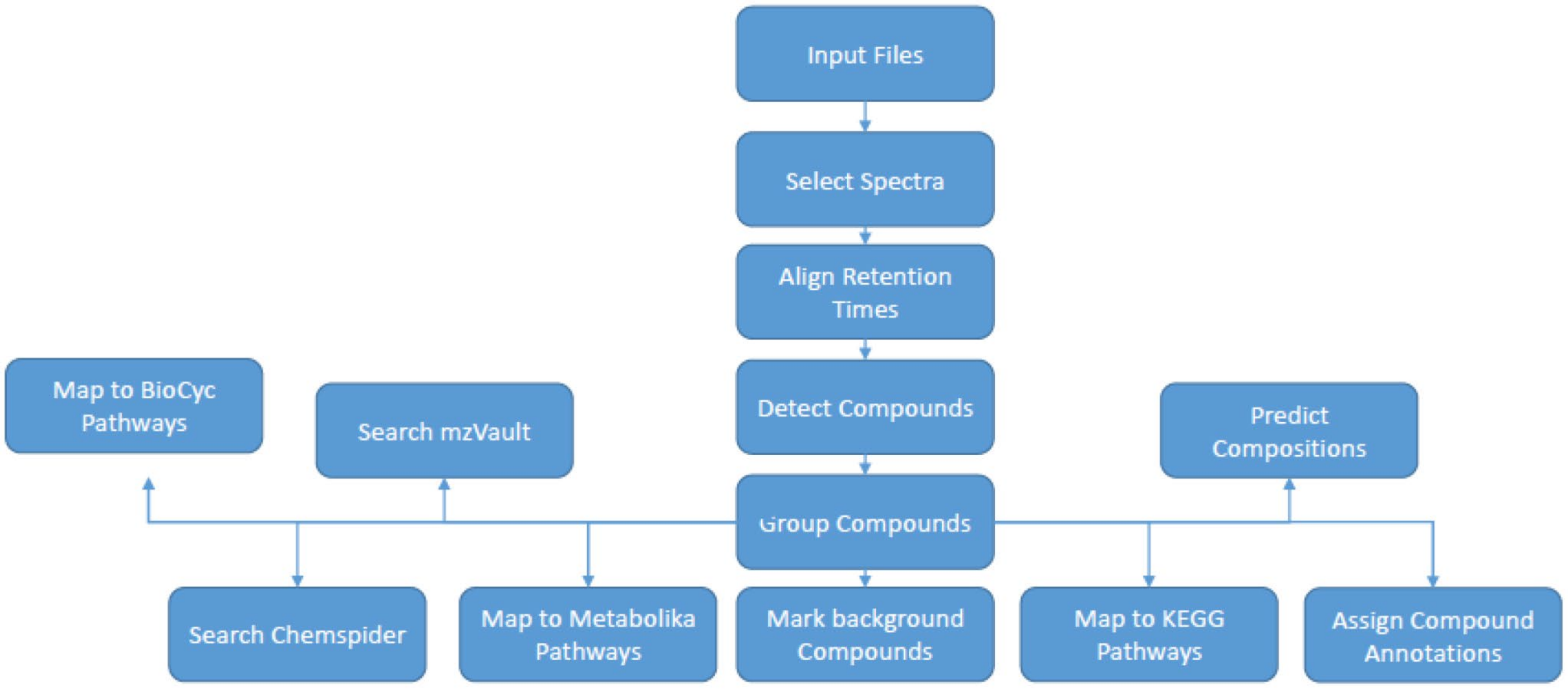
Compound Discoverer 3.0 raw data processing workflow applied for the processing of raw data collected for human plasma and urine with input files being the first process

All raw data processing steps (peak peaking and data integration) were performed in Compound Discoverer 3.0 (Thermo Scientific). Raw data files (.RAW format) for the human plasma and urine samples were imported into Compound Discoverer 3.0 (Thermo Scientific) where the data were processed by applying peak picking, peak integration and data integration across all samples at all dilutions for each sample type separately. One data matrix was constructed for each sample type/assay combination of compound (rows) and sample (columns) with the extracted ion chromatogram peak area for all metabolite features of the same compound reported where a compound was detected in a specific sample. After processing, the software was used to annotate the compounds detected using the Chemspider search modules in which MS1 data is matched to molecular formulae. All metabolites were annotated to level three as defined by the Metabolomics Standards Initiative (Sumner et al., 2007). MS2 data were not collected for this section of the work as the main focus was the chromatography and number of compounds detected rather than the annotations themselves. The Compound Discoverer workflow is presented in Fig. 1 and parameters used can be found in Supplementary Information S2.

#### Porcine sample data

**Fig. 2:**
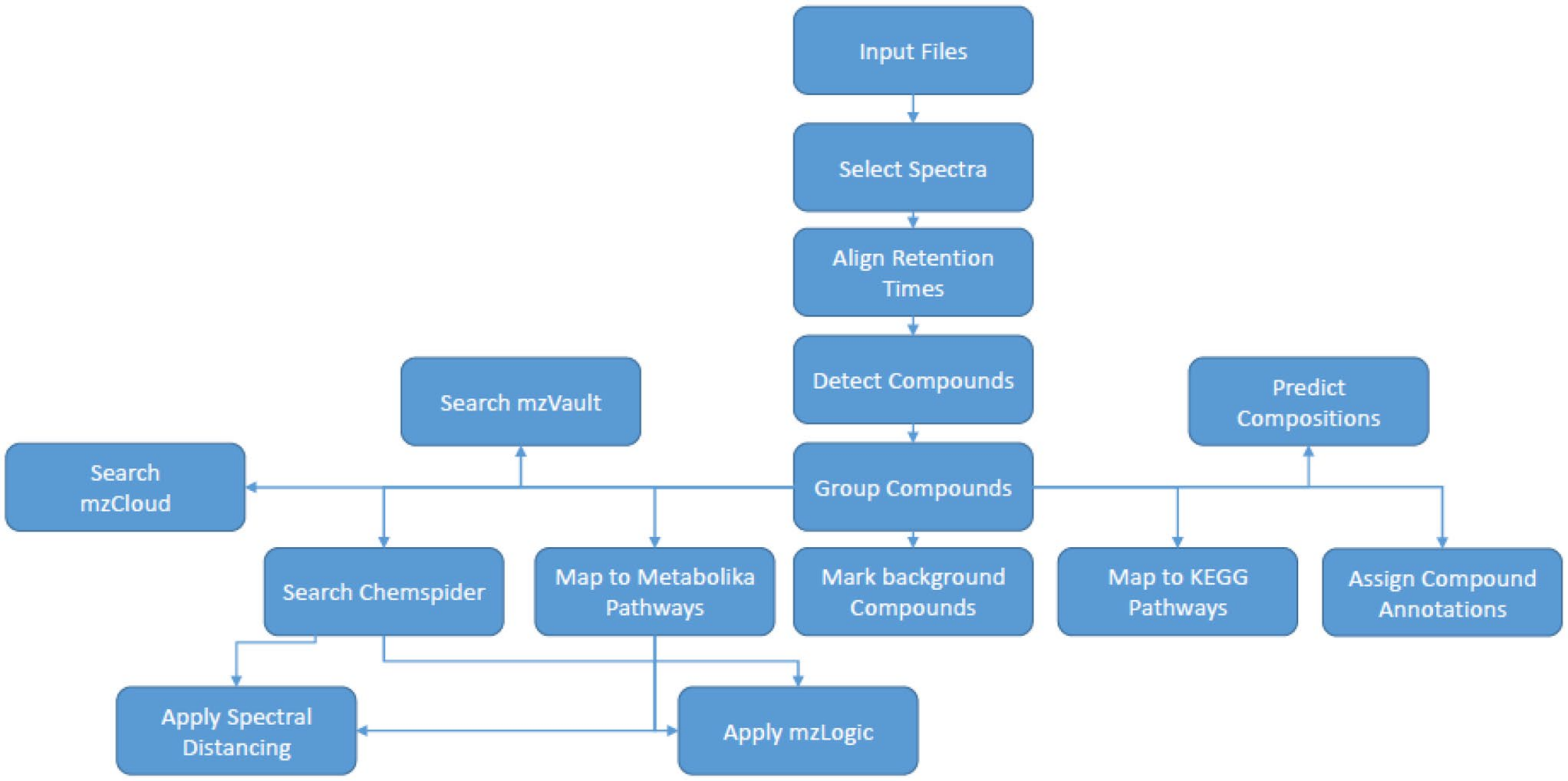
Compound Discoverer 3.0 raw data processing workflow applied for the processing of raw data collected for porcine plasma, urine and tears with input files being the first process

The porcine sample data files were processed in a similar workflow, using Compound Discoverer 3.1 (Thermo Scientific). After processing the software was used to annotate the compounds detected. MS1 data was matched to molecular formulae and metabolites using Chemspider searches. MS2 data were searched against mzCloud (Thermo Scientific) and an in-house MS/MS mass spectral library constructed with authentic chemical standards. All metabolites were annotated to level 2 or level 3 as defined by the Metabolomics Standards Initiative (Sumner et al., 2007) The Compound Discoverer workflow is presented in Fig. 2 and parameters used can be found in Supplementary Information S3.

#### Human and porcine sample data

Following export of the data from Compound Discoverer as .csv files, blank filtering was applied in Microsoft Excel to remove compounds whose mean peak area in the blank samples was greater than 10% of the mean peak area in the QC samples. Subsequently, QC response filtering was applied to remove compounds whose QC sample peak area relative standard deviation was greater than 30%. Relative standard deviations were calculated in Microsoft Excel using raw peak areas with no normalisation applied and were calculated as [(mean/standard deviation) * 100] and were reported as a percentage.

### Data analysis

#### Human samples

Comparisons for the number of compounds reported, the mean peak area and mean peak width were carried out for each UHPLC column at each sample dilution. This was completed for each dilution within a column so that the effect of sample dilution could be determined along with the effects caused by changing the column i.d. For peak width and peak area comparisons, the compounds per file outputs from Compound Discoverer were used to compare the full width half max (FWHM, minutes) and chromatographic peak area of peaks present across each dilution. Prior to comparisons, the datasets were filtered by removing peaks with a FWHM > 0.2 min on the basis that those peaks were most likely to be solvent peaks and/or were peaks of poor chromatographic resolution or shape. For comparing the number of compounds reported, filters removed peaks that were not present in 75% of replicates in the dilution being studied and which were reported with a relative standard deviation (RSD) > 30%. Box and whisker plots were constructed in R (v4.0.0) using the ggplot2 package.

#### Porcine samples

Outliers were discovered and removed post processing by summing the peak areas within each sample. Samples with total peak areas much lower than others of the same sample type were omitted from any further analysis. This was done to improve confidence in results by removing any significant bias. Filtering was then applied to each sample type to remove compounds that were not present in a minimum of two samples (regardless of how many samples were present). These samples were then filtered to remove any compounds that had a RSD > 30% for QC samples. To determine whether metabolites were detected in one, two or three of the sample types (plasma, urine, tear fluid), compounds which were within 0.001Da molecular weight of each other and the retention times were within 0.1 min of each other for the same assay, were classed as a matching compound. Datasets were checked for retention time shift before compound matching was conducted. Venn diagrams were plotted in Microsoft Powerpoint.

### Multivariate and statistical analysis

Principal Components Analysis was performed using MetaboAnalyst (v5) (Pang et al., 2021). Data treatment was applied as defined previously (Di Guida et al., 2016). PCA analyses applied missing value imputation (kNN (feature-wise), no data filtering, normalisation by sum, log transformation and Pareto scaling.

## Results and discussion

The application of microbore columns to the metabolomic analysis of mammalian biofluids and tissues offers potential advantages in relation to lower solvent volume and sample volume/mass required as well as increased chromatographic resolution and sensitivity. However, there have been only a small number of comparisons of 1.0 mm i.d. to 2.1 mm i.d. columns for mammalian sample types (Gray et al., 2015; Fitz et al., 2022; Geller et al., 2022; King et al., 2019). Typical chromatograms for each assay, sample type and column i.d. are available in Supplementary Information S4.

### Method development applying human plasma and urine samples

**Fig. 3:**
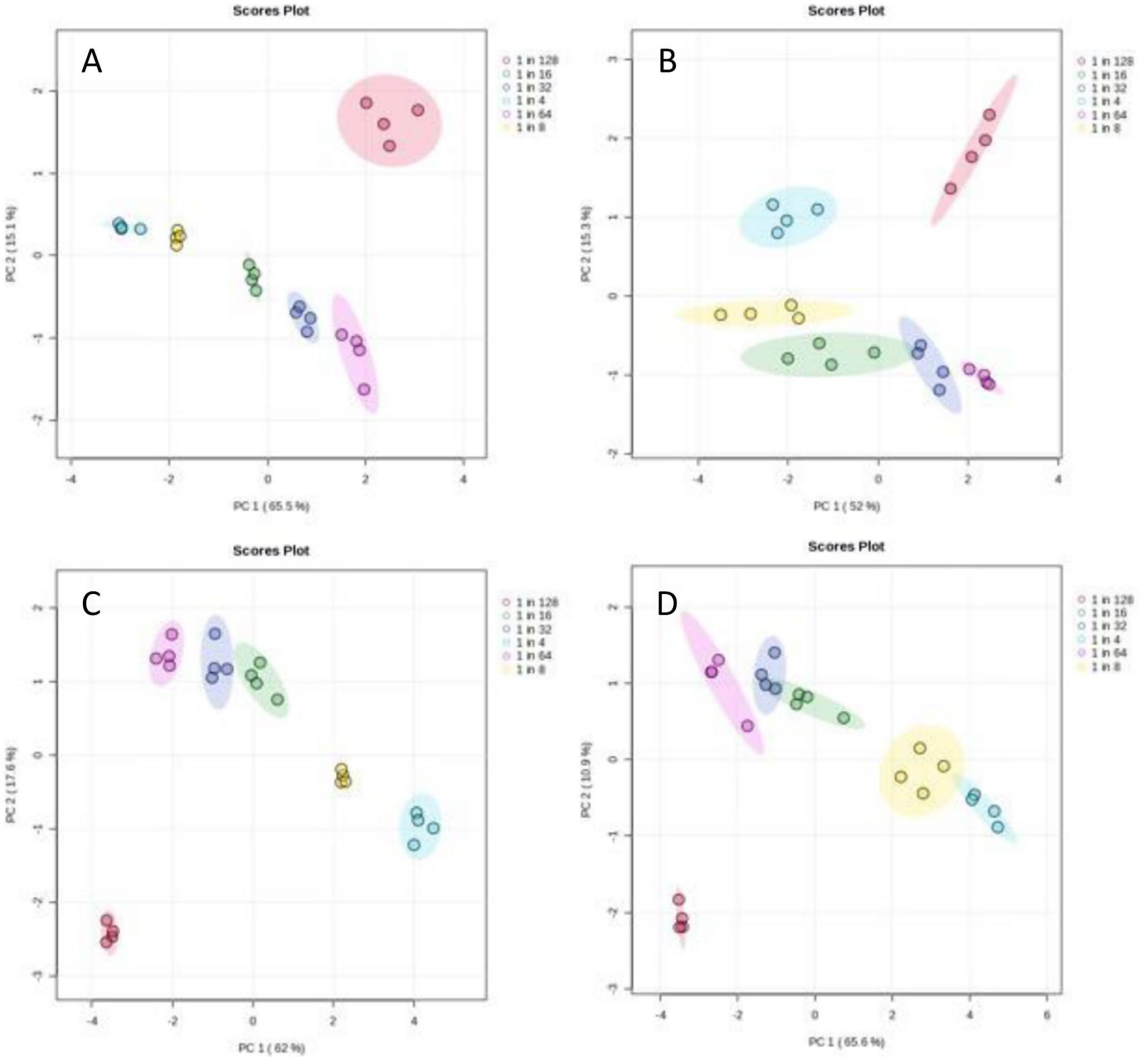
Principal Components Analysis scores plots (for PC1 vs. PC2) for (**A**) plasma samples analysed applying a HILIC negative ion mode method using a 2.1 mm i.d. column, (**B**) plasma samples analysed applying a HILIC negative ion mode method using a 1.0 mm i.d. column, (**C**) urine samples analysed applying a reversed phase aqueous positive ion mode method using a 2.1 mm i.d. column and (**D**) urine samples analysed applying a reversed phase aqueous positive ion mode method using a 1.0 mm i.d. column. Red symbols represent a 1 in 128 dilution, purple symbols represent a 1 in 64 dilution, dark blue symbols represent a 1 in 32 dilution, green symbols represent a 1 in 16 dilution, yellow symbols represent a 1 in 8 dilution and cyan represent a 1 in 4 dilution. Elipses represent 95% confidence limits for each dilution class

***Reproducibility***: PCA scores plots were used to visualise the distribution of the data and identify any trends in relation to sample dilution or outliers. Examples for plasma applying a HILIC negative ion mode assay and for urine applying a reversed phase aqueous assay can be found in Fig. 3 and all scores and loadings plots can be viewed in Supplementary Information S5. The PCA plots for both the 2.1 mm and the 1.0 mm i.d. columns show similar levels of clustering and variance between sample dilutions for all of the three assays and the major separation was observed related to dilution. Relative standard deviations (RSD) were calculated at each dilution for each assay and sample type (Supplementary Information S6) and demonstrated similar RSD ranges when comparing 2.1 mm i.d. column data to 1.0 mm i.d. column data. It can be concluded that a similar level of reproducibility and distribution related to sample dilution for both column internal diameters (1.0 and 2.1 mm) were observed. Short and long-term reproducibility over batches of up to one thousand samples have been demonstrated previously (Gray et al., 2015; Gadara et al., 2024).

***Comparison of number of detected compounds***: The number of compounds detected in each dilution for the same sample type and different column IDs were compared. The number of compounds reported in each dilution decreased for both the 1.0 mm and 2.1 mm i.d. columns, defining that dilution effects were observed as expected, although this decrease was not always linear. When standard sample dilution methods (1 in 4 dilution) were investigated, the 1.0 mm i.d. assays did not detect as many compounds when compared to data for the 2.1 mm i.d. assays. There was one exception, the HILIC positive ion mode assay which for plasma detected a significantly higher number of compounds for the 1.0 mm i.d. column. This can be seen in Supplementary Information S7. The complete set of results and summary are available in Supplementary Information S8 and S9. In previous research, similar numbers of metabolites have been reported for both column types and this matches similiarly to the work presented here (Geller et al., 2022). However, using shorter analysis times with higher velocities and faster gradient elution does reduce the number of metabolites detected (Gray et al., 2016).

**Fig. 4:**
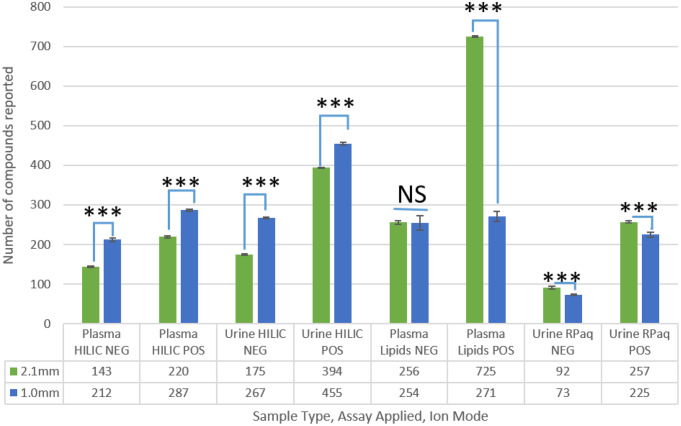
The average number of reported compounds in human plasma and urine samples following a 1 in 16 dilution for each of three UHPLC-MS assays. Compounds which were present in 75% of replicates and had a relative standard deviation (RSD) < 30% are reported. 2.1 mm i.d. column (green) and 1.0 mm i.d. column (Blue) *** has a significance of *p* < 0.001, NS has no significance

The 1 in 16 dilution data showed that all of the HILIC assays for both plasma and urine detected significantly more compounds in the 1.0 mm i.d. assay than the 2.1 mm i.d. assay (Fig. 4) and this conclusion was observed for the 1 in 8 dilution data also for plasma and urine (negative ion mode). This suggests that the 1.0 mm i.d. HILIC assays could operate at higher sensitivity at larger sample dilutions which was what was expected. This trend in the HILIC assays continues with all further dilutions (1 in 32 to 1 in 128) containing more detected compounds in the 1.0 mm i.d. assay compared to the 2.1 mm i.d. assay. Both the lipidomics assays and reversed phase aqueous assays, which both apply C_18_ columns but from different commercial sources and with different particle sizes, did not follow the same trend for detected compounds as for the HILIC assays. Instead, for these assays the 2.1 mm i.d. assay detected either a similar number of compounds or more compounds in the majority of the sample dilutions studied.

**Fig. 5:**
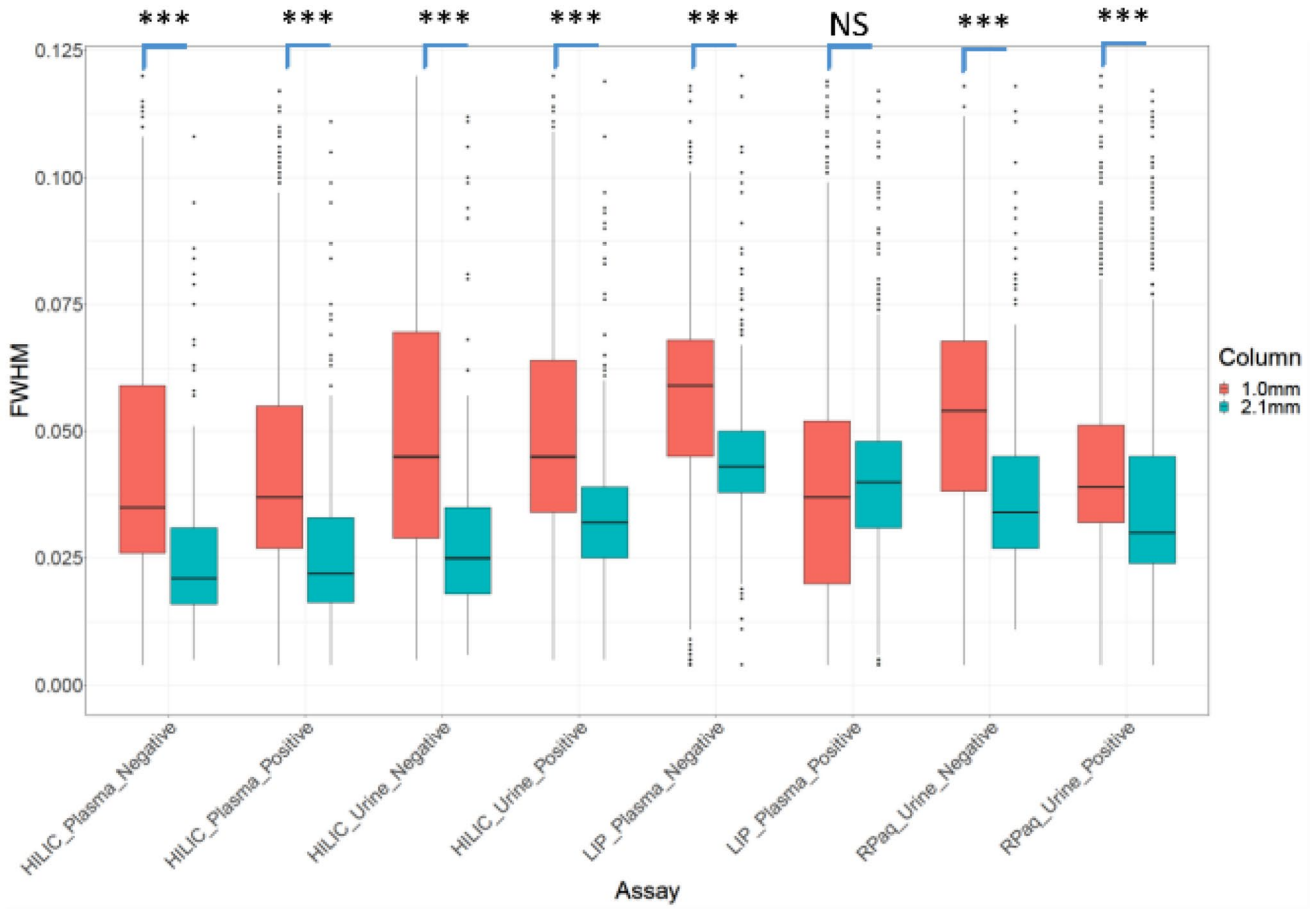
The distribution of chromatographic peak widths in the 1 in 16 dilution of each assay type for both the 2.1 mm i.d. (blue) and 1 mm i.d. (red) columns for HILIC, lipidomics (LIP) and reversed phase aqueous (RPaq). Compounds which were present in 75% of replicates and had a relative standard deviation (RSD) < 30% are reported. *** has a significance of < 0.001, NS has no significance. FWHM has units of minutes

***Chromatographic peak widths and associated chromatographic resolution***: The full width half max (FWHM) chromatographic peak widths of all reported compounds were investigated to determine the effect of column internal diameter and the associated change in flow rate on chromatographic peak widths. Figure 5 shows the distribution of chromatographic peak widths (FWHM) for all reported compounds for all assays, sample types and column IDs for 1 in 16 diluted samples. The median peak width is generally higher for the 1.0 mm i.d. column compared to the 2.1 mm i.d. column, with the exception of the lipidomics assays, where the median peak widths were similar.

These trends were observed across all dilutions (see Supplementary Information S10) and so it can be concluded that the peak widths in the 1.0 mm i.d. assays are larger than observed in the 2.1 mm i.d. assays. There are many reasons why this could be occurring. The first possible reason could be the flow rate and associated flow velocities applied for the 1.0 mm i.d. columns. UHPLC chromatographic resolution is influenced by the flow velocity with lower flow velocities typically being sub-optimal for a given column i.d. and length. The smaller i.d. columns were operated at lower flow rates to allow operation below the UHPLC column back pressure limit of 1200 BAR. We calculated the flow velocities for both i.d. columns and the 1.0 mm i.d. column had a higher flow velocity (1019 cm/hour for a 0.13 mL.min^− 1^ flow rate) compared to the 2.1 mm i.d. column (693 cm/hour for a 0.40 mL.min^− 1^ flow rate). Therefore, non-optimal flow velocity is not a potential reason for wider chromatographic peaks as a result of eddy diffusion as the flow velocity is 1.5 times higher for the 1.0 mm i.d. column compared to the 2.1 mm i.d. column. The second possible reason for wider chromatographic peaks is band broadening occurring at the time of injection as the same injection volume (2µL) was applied for both column IDs and would mean that the smaller i.d. column would have an approximately 4.4 times wider band width just after injection than for the 2.1 mm i.d. column. A third possible reason could be that at lower flow rates for the 1.0 mm i.d. column band broadening can occur in post-column tubing including the electrospray ion source needle as has been reported previously (Gray et al., 2015). Supplementary Information S11 visualises the FWHM peak width for all peaks before any quality filtering for all assays, sample types and column i.d. In some cases the mean peak width was similar across the run (e.g. lipid assays for plasma samples on both i.d. columns) whereas there were cycles of higher and lower peak widths for other cases (e.g. analysis of urine applying HILIC assays). The reason for peak broadening can not be derived from the data collected. Further assessment of band broadening was not performed as this would require injection of 0.40µL volumes to ensure the same injection band width was not a factor contributing to band broadening and this volume is too low to reproducibly inject on the UHPLC system applied.

For all assay-sample type combinations there were no significant changes in peak width as sample dilution increased (Supplementary Information S12) with one exception, the lipids negative assay applied to the analysis of plasma which showed the same peak width for the 2.1 mm assay for all dilutions whereas the peak widths increased for the 1.0 mm assay as sample dilution increased.

#### Chromatographic peak areas

The distribution of peak areas was investigated to determine whether lower numbers of reported compounds was a result of lower reported peaks areas. Each assay displayed a small drop in peak area as the dilution of the sample increased (Supplementary Information S13). This shows that the peak area was proportional to the absolute concentration of compound present in the sample, which is expected and reported commonly in analytical chemistry. Data for the 1 in 16 dilutions for all samples and assays are shown in Supplementary Information S14. The results show that although the distributions for the two column i.d. overlap considerably, the peak areas for the 1.0 mm i.d. columns are significantly larger when compared to the 2.1 mm i.d. columns for HILIC assays (negative ion mode) and Lipidomics assay (negative ion mode). Peak areas from the 1.0 mm i.d. assays were expected to be higher due to either an increased chromatographic resolution leading to enhanced signal-to-noise ratios and/or as a result of the lower flow rates applied for the 1.0 mm i.d. columns which allows greater efficiency in ion formation and desolvation in the electrospray ion source. Increased sensitivity with narrowbore columns has been reported previously (Gardara et al., 2024; Fitz et al., 2022; Gray et al., 2015).

### Application of 1.0 mm i.d. columns to study different biofluids

**Fig. 6:**
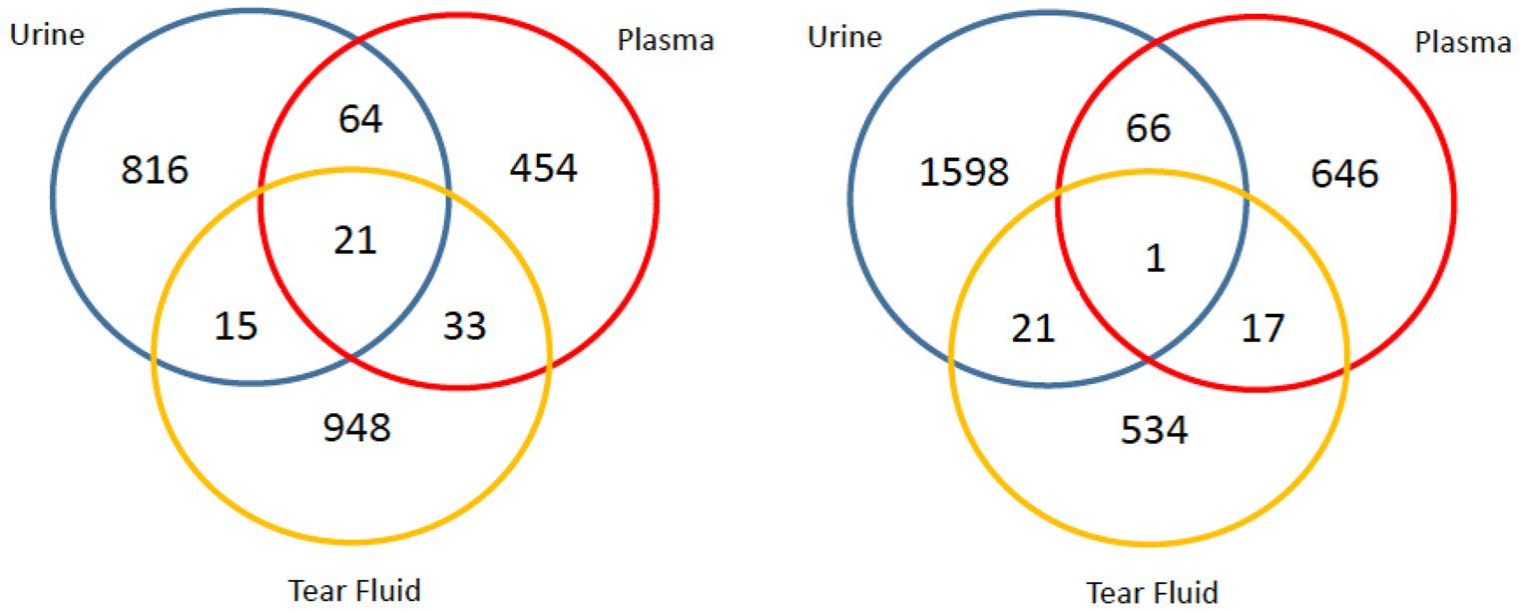
Venn diagram displaying the number of compounds uniquely detected for porcine urine (blue), plasma (red) and tear fluid (yellow) and the number of compounds detected in two or three sample types applying a HILIC negative ion mode assay (left panel) and HILIC positive ion mode assay (right panel)

Previous research has shown the application of microbore LC columns to the analysis of urine (Gray et al., 2015, 2016), plasma (Fitz et al., 2022; King et al., 2020), dried blood spots (Feng et al., 2023), single cerebral organoids (Gadara et al., 2024) and urine/brain (Geller et al., 2022) applying reversed phase (including lipidomics) and ion pairing chromatography-mass spectrometry. Chen and colleagues have characterised the human tear metabolome previously applying 2.1 mm i.d. columns (Chen et al., 2011). We applied 1.0 mm i.d. columns to analyse porcine plasma, urine and tear samples using three different assays. This research was conducted for (1) the characterisation of the tear metabolome, (2) to assess the overlap between the tear, plasma, and urine metabolomes, and (3) to assess whether the 1.0 mm i.d. assay could be applied to analyse small volume samples including tears. This would support research for whether tear fluid would be an appropriate sample type for metabolomics research. Results are available in supplementary information (Supplementary Information S15 and S16) and an example is included in Fig. 6. All assays demonstrated that hundreds or thousands of compounds (as reported by Compound Discoverer) were detected in each sample type, similar to the numbers of compounds regularly detected in published studies. The developed 1.0 mm i.d. assays are therefore appropriate to be applied for untargeted metabolomic studies of mammalian biofluids including low volume tear samples which were diluted during extraction by a factor of 6.8 and then concentrated by a factor of 5 upon reconstitution for polar metabolites and 3 for non-polar metabolites.

Applying the HILIC assay to all three sample types showed both pairwise and three-way overlaps of polar compounds (Supplementary Information S16). There was some concern that the urine-plasma overlap should have been larger than what was seen for both the positive and negative ion modes of this assay type. This is because in the Human Metabolome Database (HMDB) there are 25,372 plasma metabolites and 4,237 urine metabolites of which 3,668 metabolites are present in both metabolomes (though it should be noted that this includes lipid metabolites, which were not compared for the porcine plasma and urine during this research).

For the non-polar assays, all showed a small overlap (< 70) in compounds between the tear fluid and the plasma/urine, as can be seen in Supplementary Information S15. As a larger overlap between tear fluid and plasma, and tear fluid and urine was expected, further data exploration was performed to investigate if there was a scientific reason why these results was observed. Single ion chromatograms of common lipids were plotted to look into the differences between tears and plasma, which showed that some of the commonly observed metabolites in plasma are not detected in tears. One possibility for the lack of detection could be due to significant differences in metabolite concentrations between the two sample types.

## Conclusions

The results from the 1.0 mm and 2.1 mm i.d. comparison showed that at standardly used dilutions (1 in 4), the 2.1 mm i.d. assay was more sensitive than the 1.0 mm i.d. assay. None of the assays showed a true increase in sensitivity at a low dilution/high concentration but when samples become more dilute/lower concentration, the 1.0 mm i.d. HILIC assays would be suitable to apply and in most cases similar number of compounds were detected at higher dilutions when comparing 2.1 mm i.d. and 1.0 mm i.d. assays. The reproducibility of 1.0 mm i.d. assays were good and comparable with the 2.1 mm i.d. assays for the same injection volume. Chromatographic peak widths and total peak areas were higher for the 1.0 mm i.d. assays compared to the 2.1 mm i.d. assays. In conclusion, the application of 1.0 mm i.d. columns is recommended for the analysis of highly diluted samples where a similar number of or more compounds are typically detected. However, for samples where > 50µL volumes are available and where sample dilutions are ≥ 1 in 4 then 2.1 mm i.d. column assays are recommended because they provide detection of a similar number or greater number of compounds.

There are some limitations to the study which the readers should be aware of. Firstly, the correct injection volume ratio should be 1/4 for the 1.0/2.1 mm i.d. columns to ensure the same band width was introduced on to the top of the column whereas a ratio of 1/1 was applied to ensure the same mass of material was injected and this may influence the results presented. Secondly, the linear velocities for the two different i.d. columns applied were not the same and this will influence band broadening in the column and in post-column tubing.

## Electronic supplementary material

Below is the link to the electronic supplementary material.

Supplementary Material 1

## Data Availability

Data available on request.
