# Supplementary material for: Development of microflow ultra high performance liquid chromatography-mass spectrometry metabolomic assays for analysis of mammalian biofluids
Source: Metabolomics. 2024 Oct 25;20(6):120. doi: 10.1007/s11306-024-02187-y (PMC11511728; doi:10.1007/s11306-024-02187-y)
Supplement: Supplementary file 1 — Supplementary Material 1 [file 11306_2024_2187_MOESM1_ESM.pptx]

## Slide 1
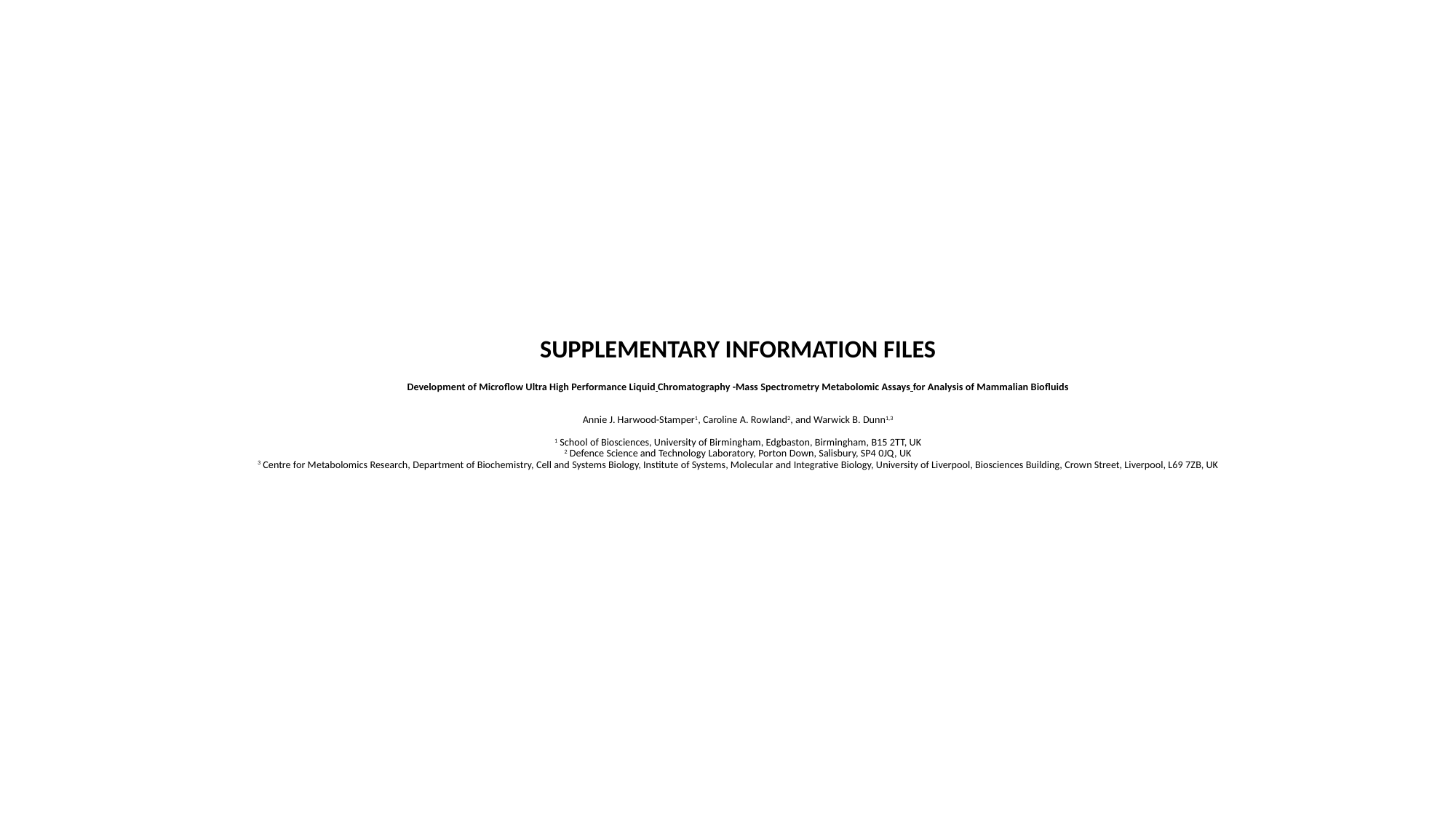

# SUPPLEMENTARY INFORMATION FILES Development of Microflow Ultra High Performance Liquid Chromatography -Mass Spectrometry Metabolomic Assays for Analysis of Mammalian Biofluids     Annie J. Harwood-Stamper1, Caroline A. Rowland2, and Warwick B. Dunn1,3   1 School of Biosciences, University of Birmingham, Edgbaston, Birmingham, B15 2TT, UK 2 Defence Science and Technology Laboratory, Porton Down, Salisbury, SP4 0JQ, UK 3 Centre for Metabolomics Research, Department of Biochemistry, Cell and Systems Biology, Institute of Systems, Molecular and Integrative Biology, University of Liverpool, Biosciences Building, Crown Street, Liverpool, L69 7ZB, UK

## Slide 2
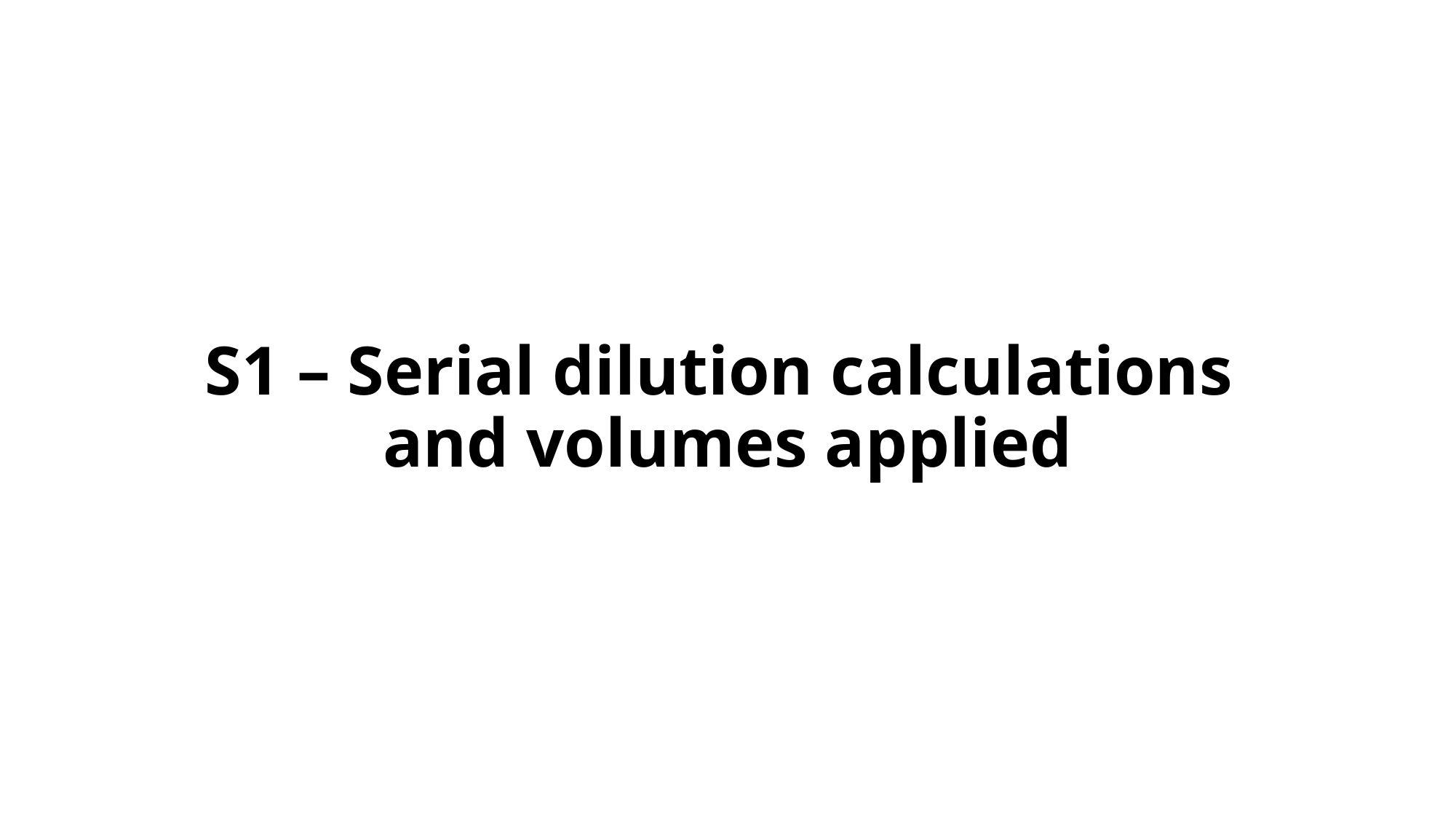

# S1 – Serial dilution calculations and volumes applied

## Slide 3
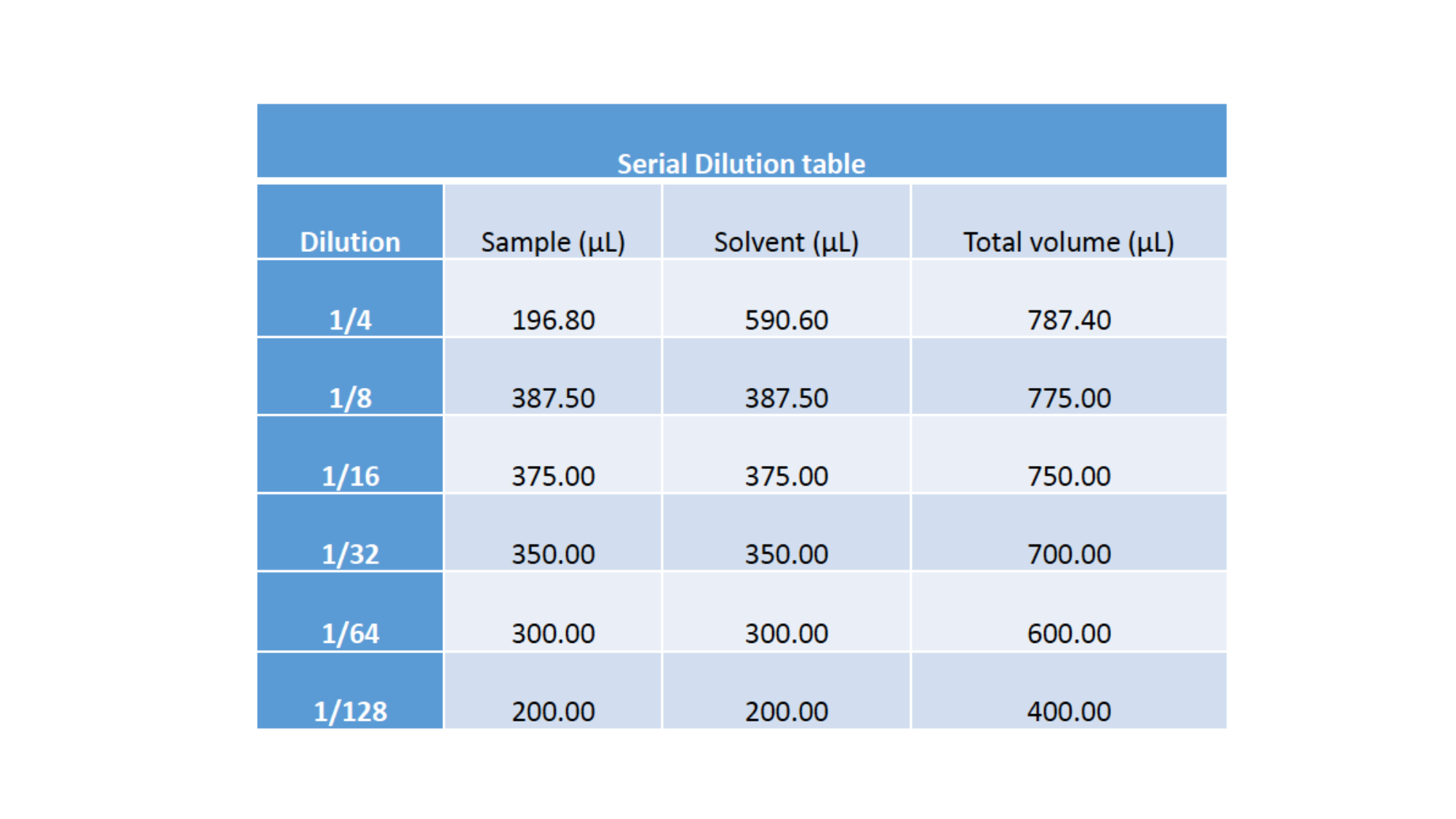

## Slide 4
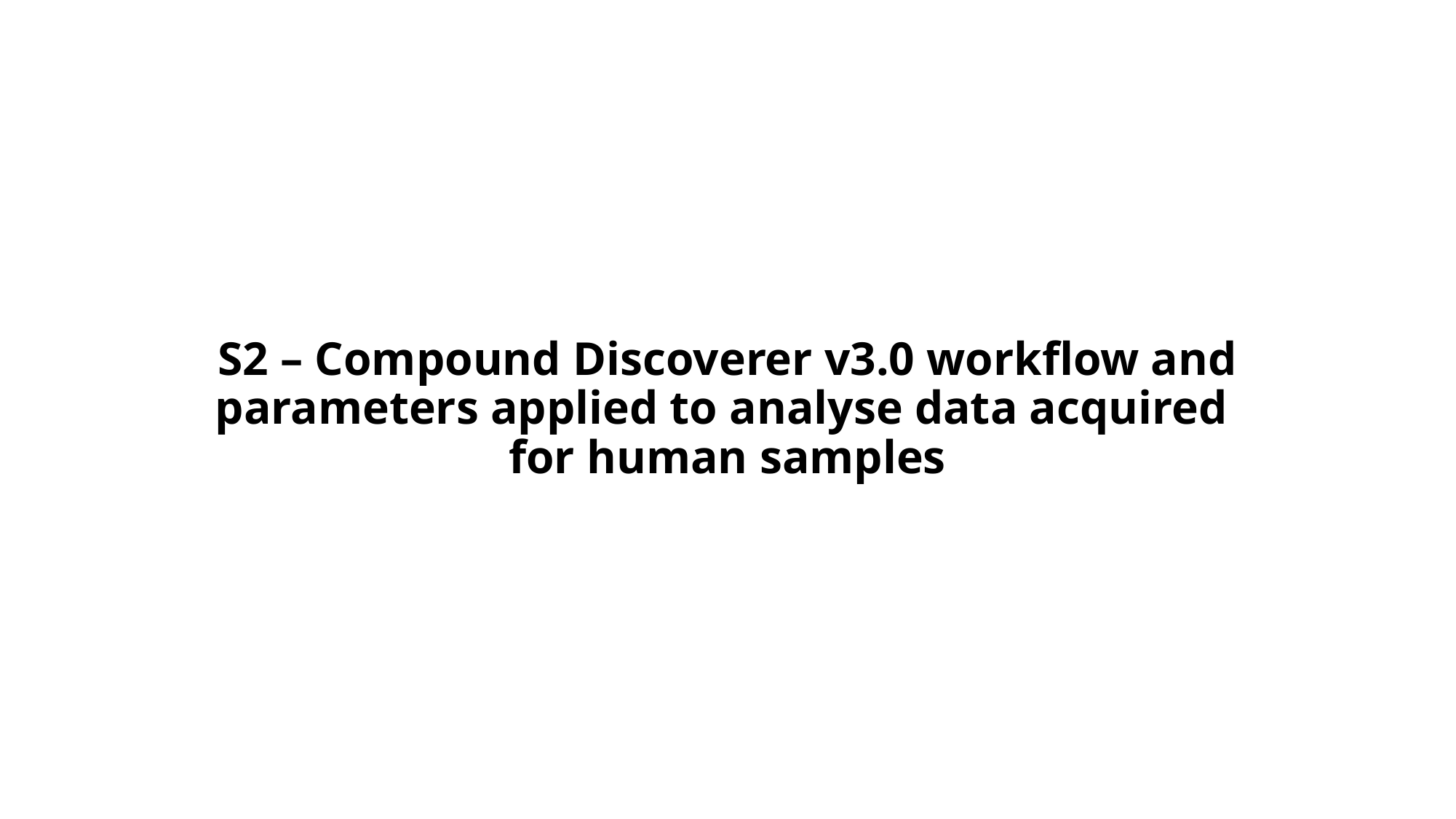

# S2 – Compound Discoverer v3.0 workflow and parameters applied to analyse data acquired for human samples

## Slide 5
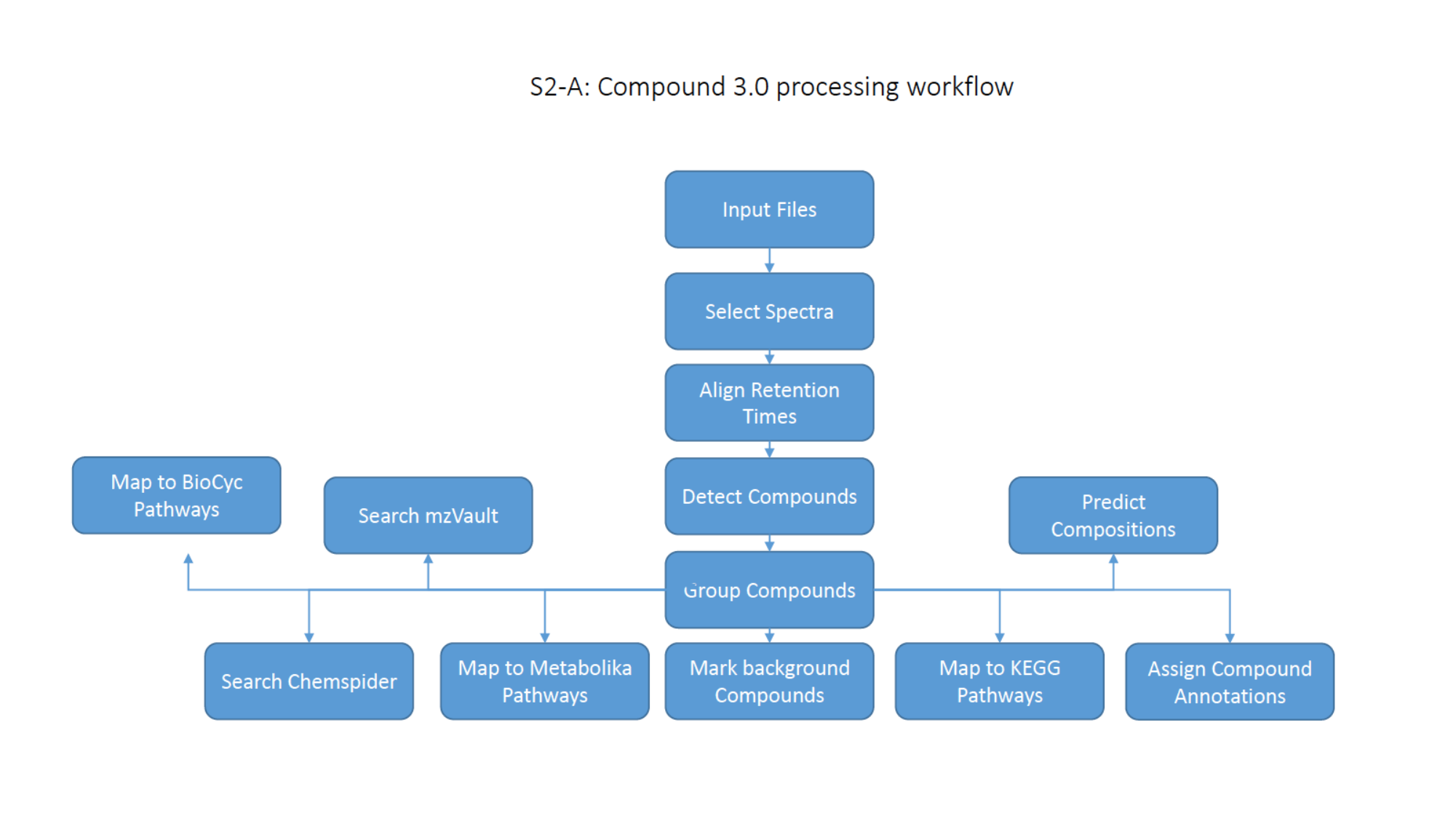

## Slide 6
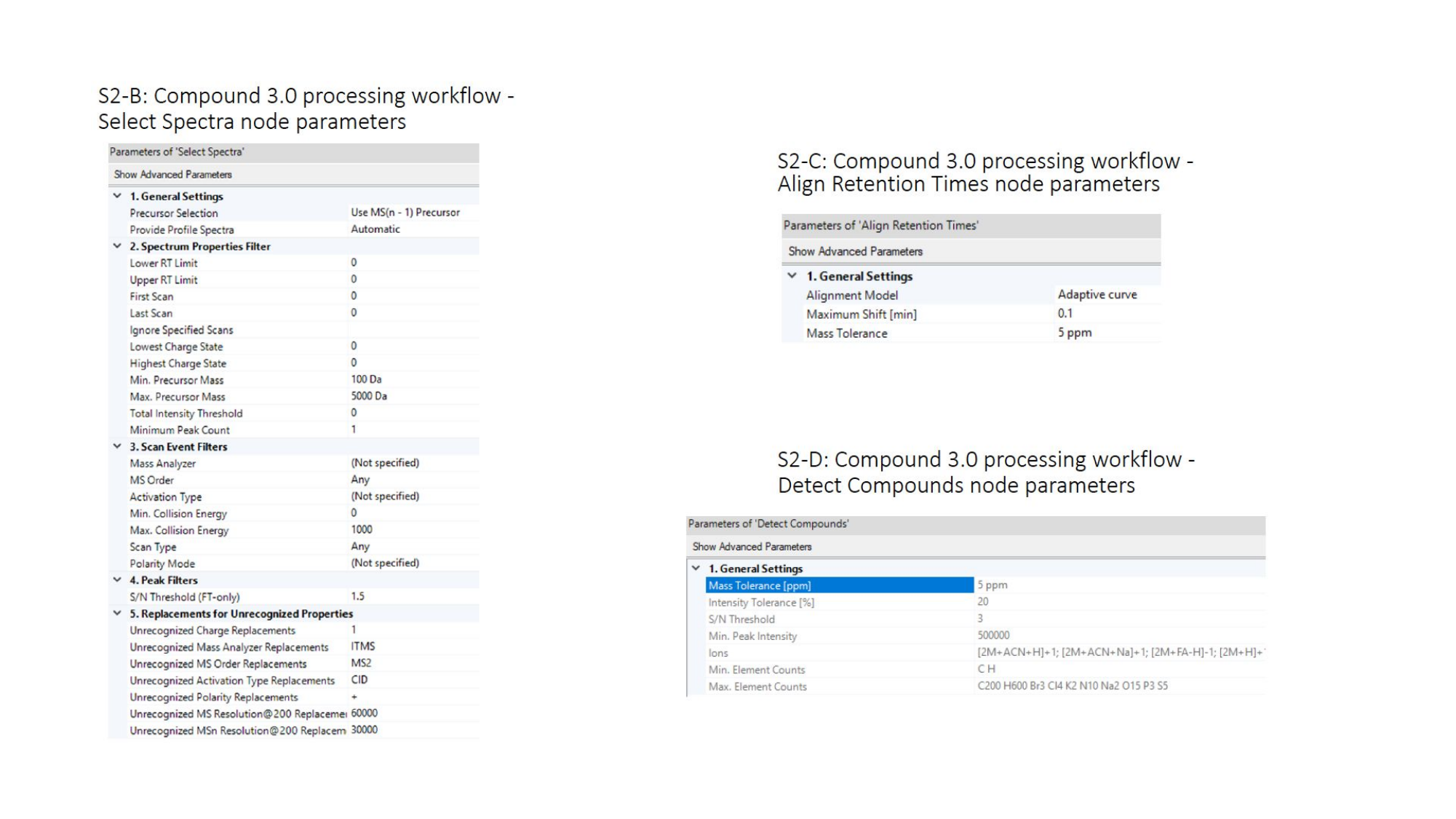

## Slide 7
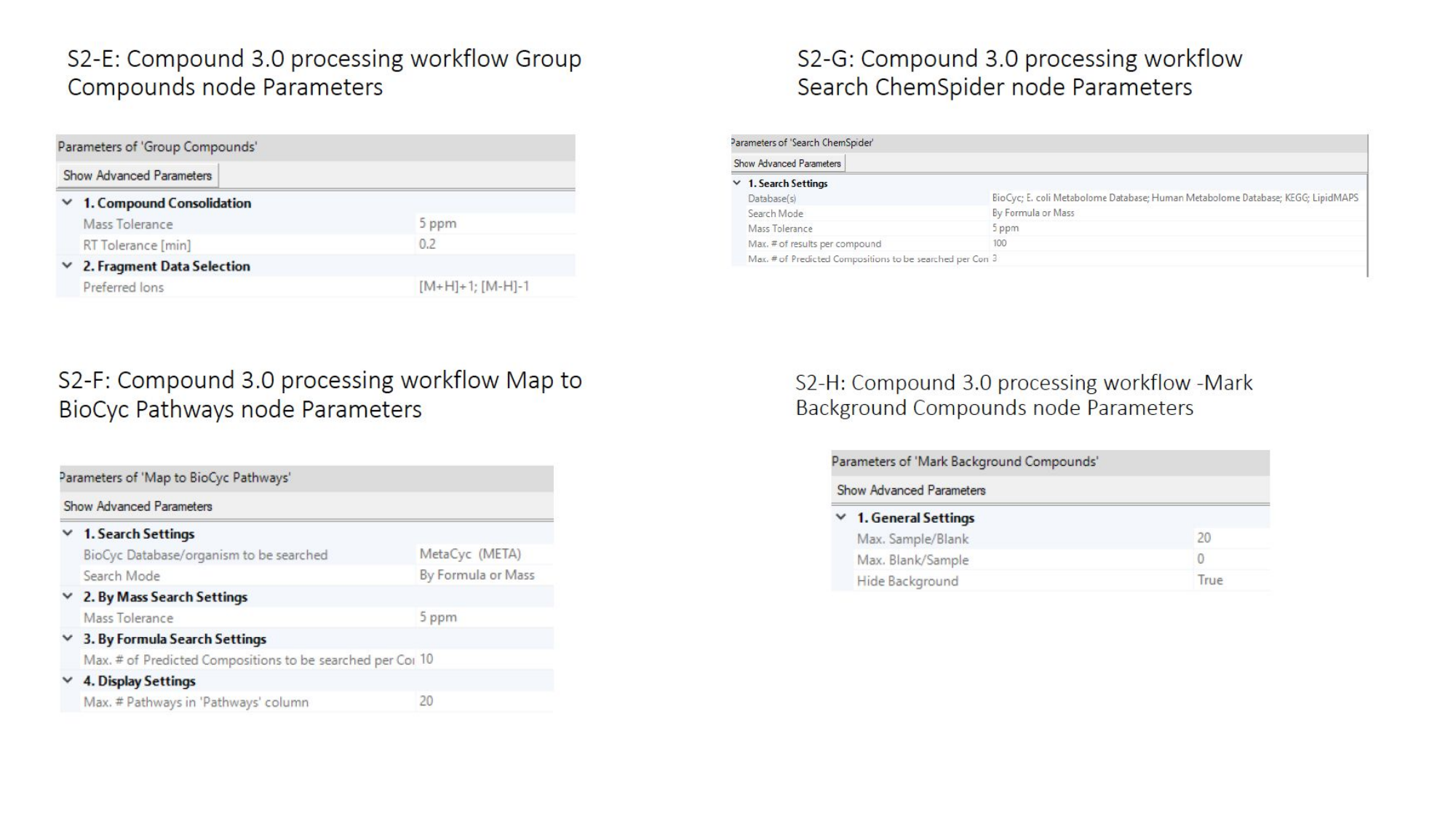

## Slide 8
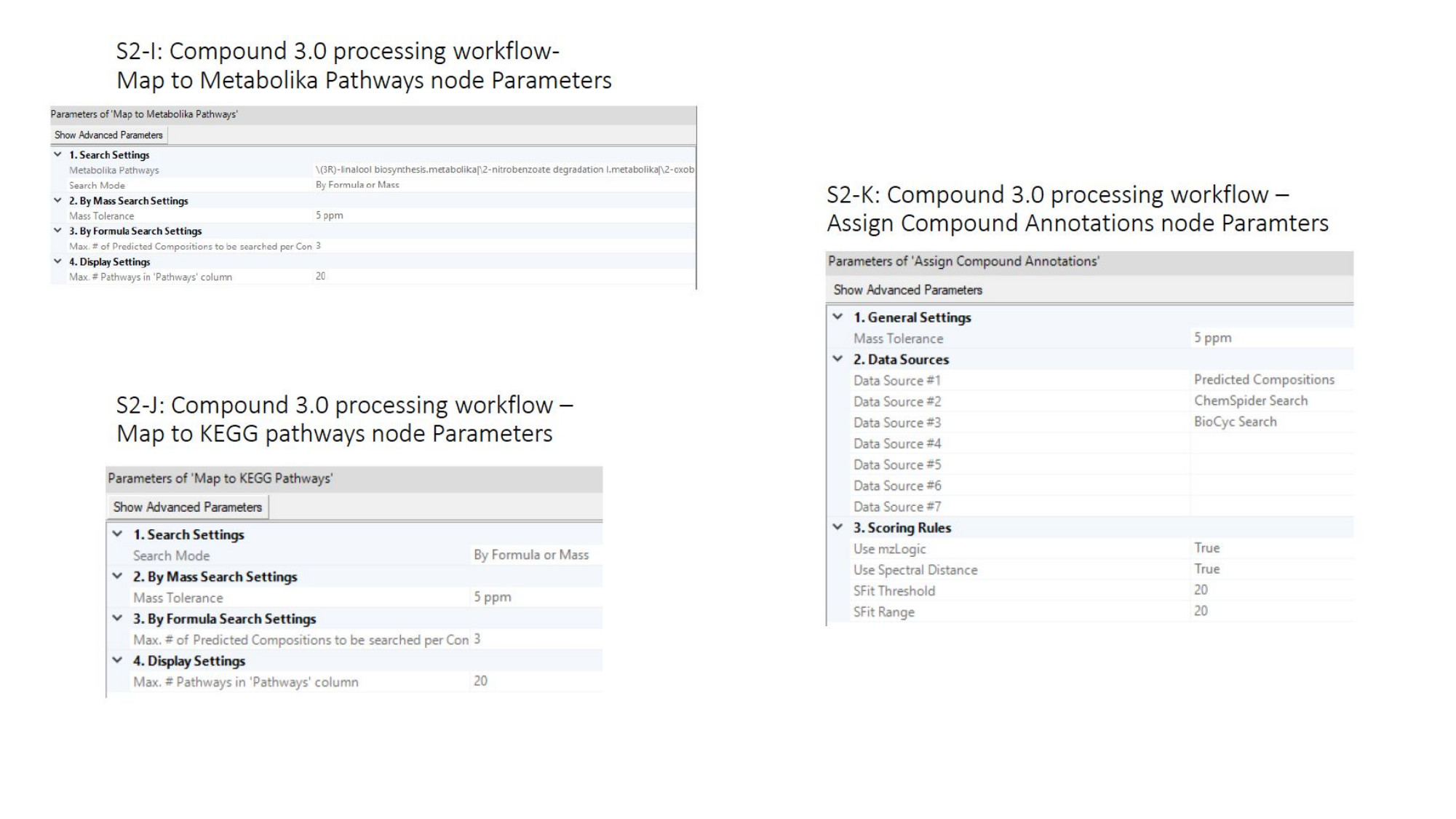

## Slide 9
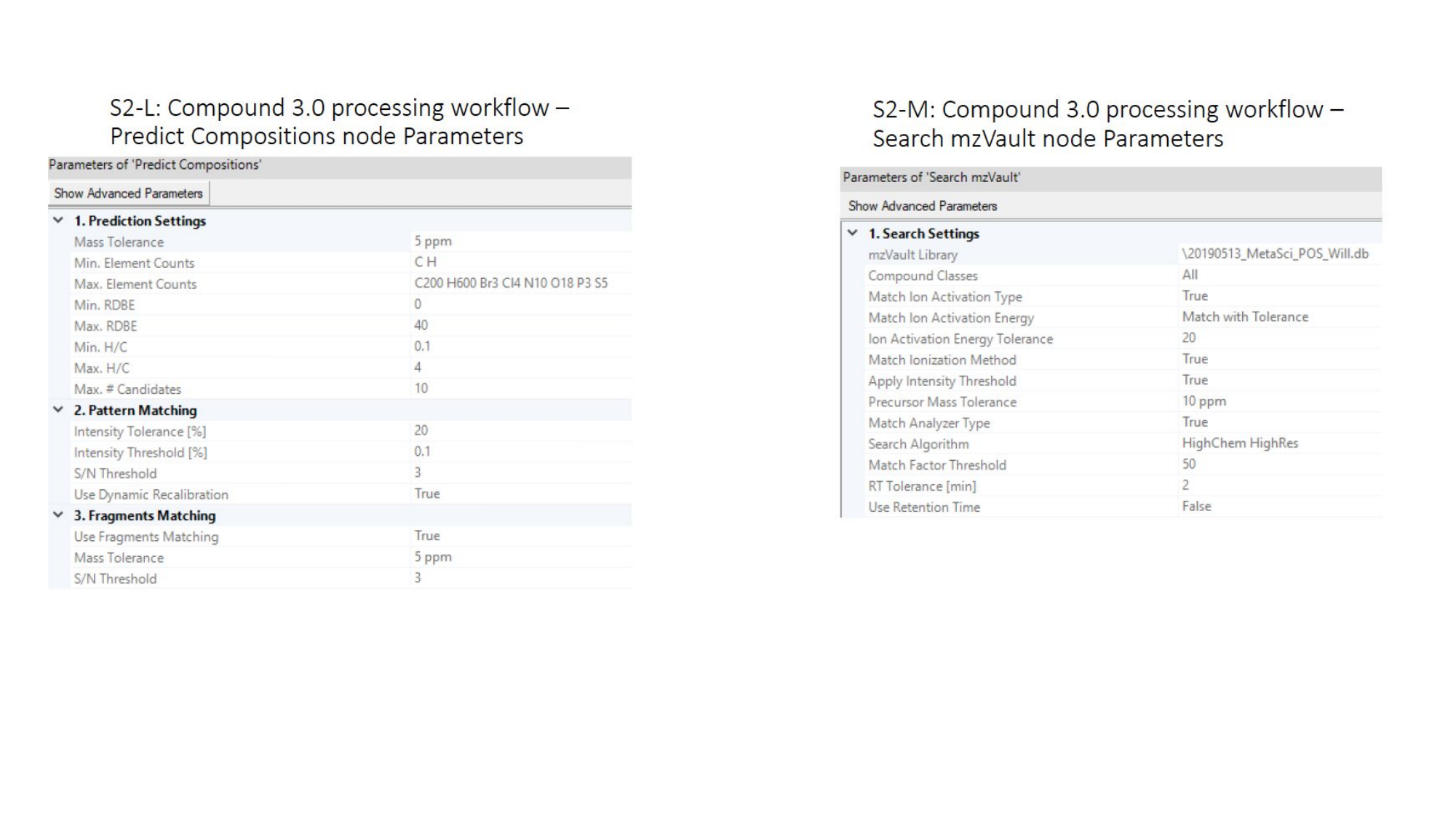

## Slide 10
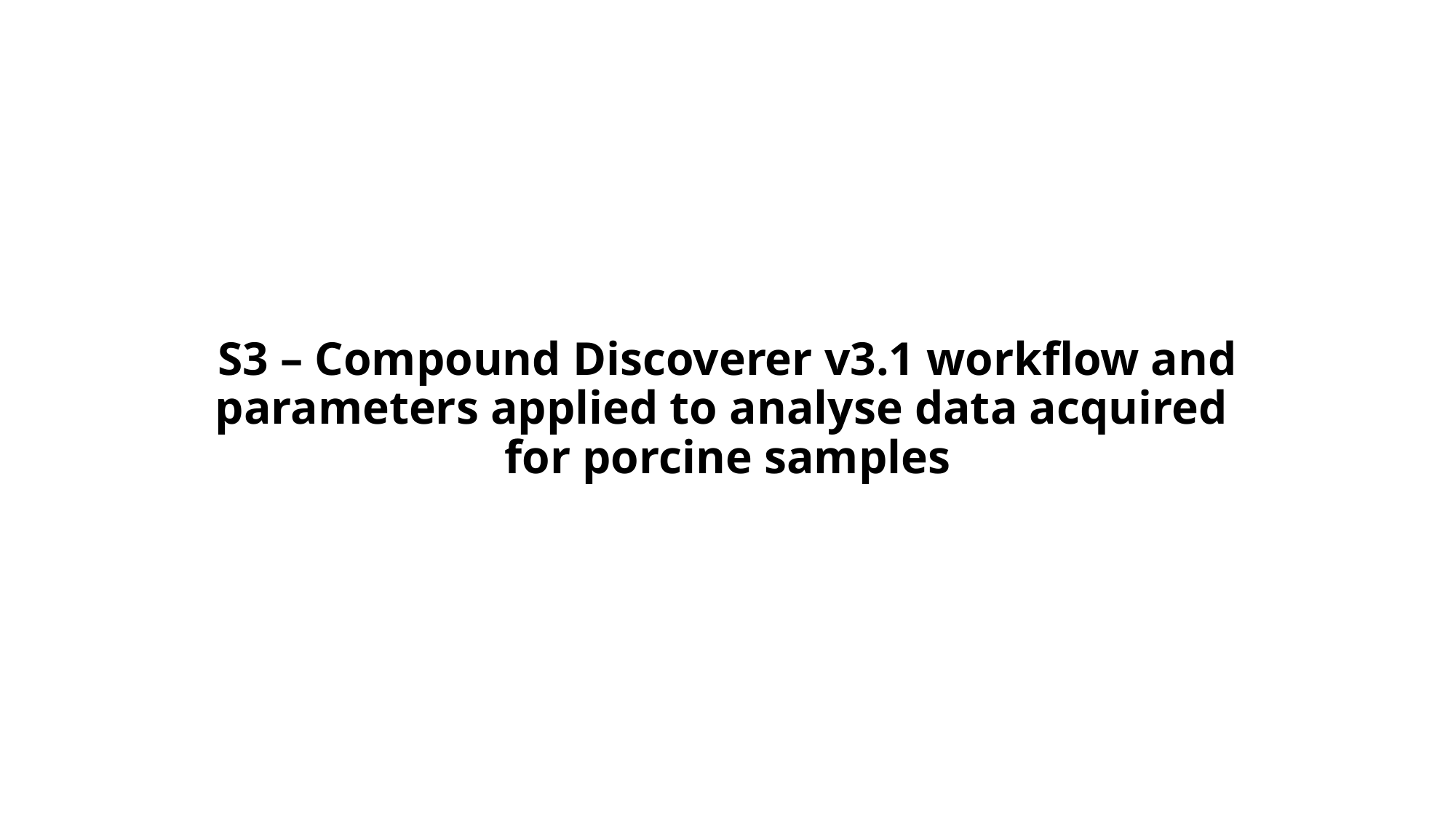

# S3 – Compound Discoverer v3.1 workflow and parameters applied to analyse data acquired for porcine samples

## Slide 11
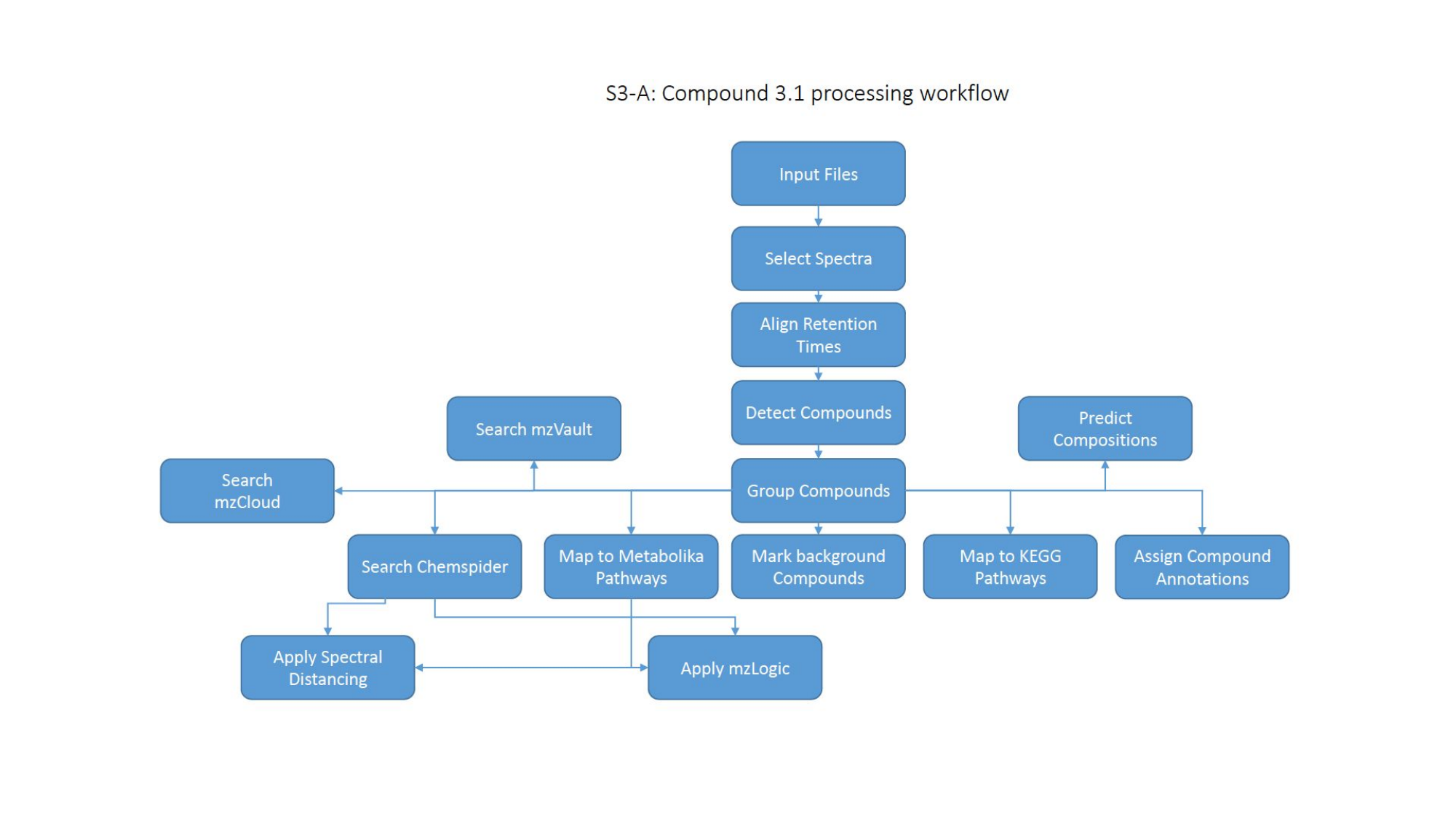

## Slide 12
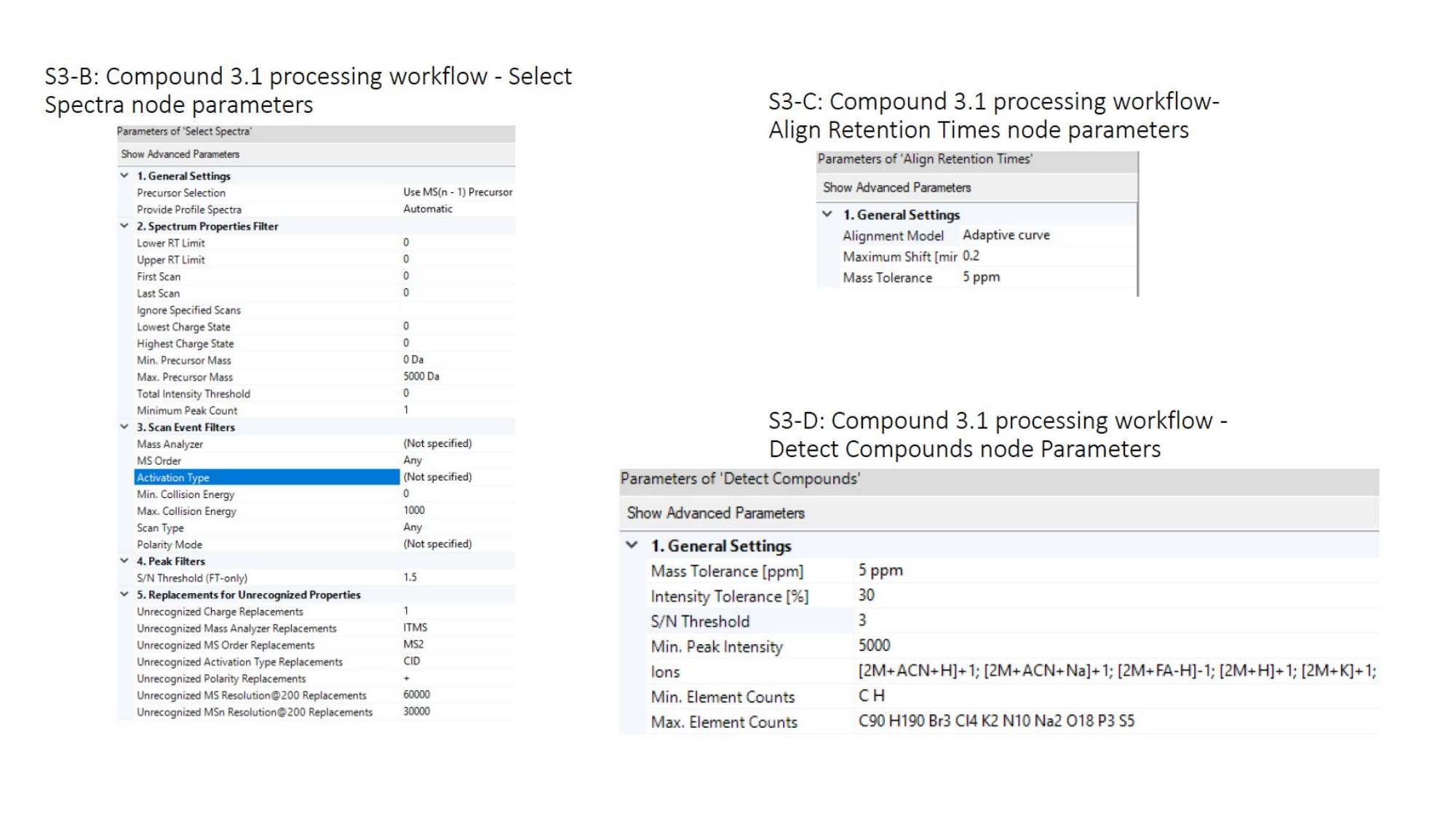

## Slide 13
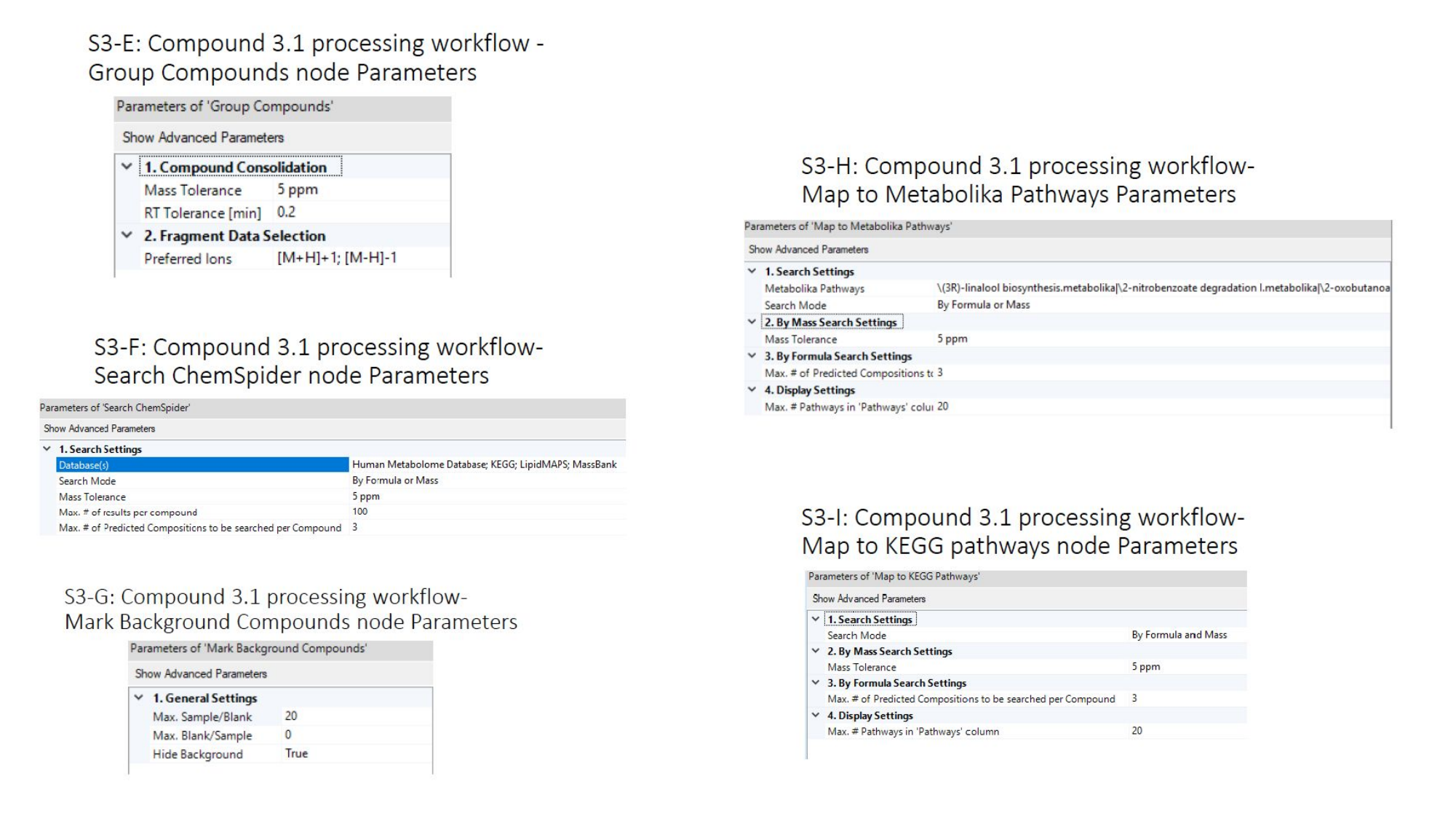

## Slide 14
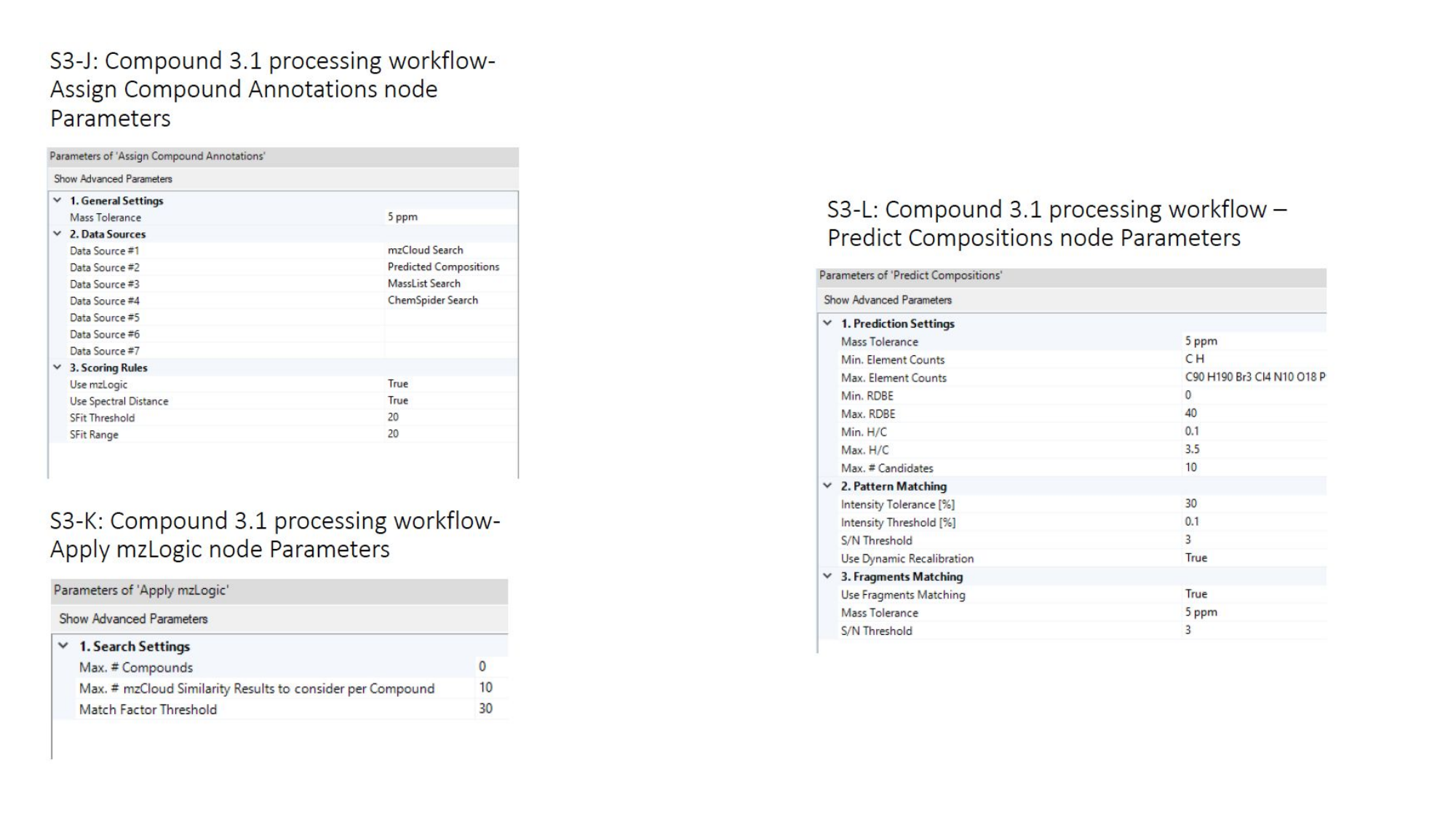

## Slide 15
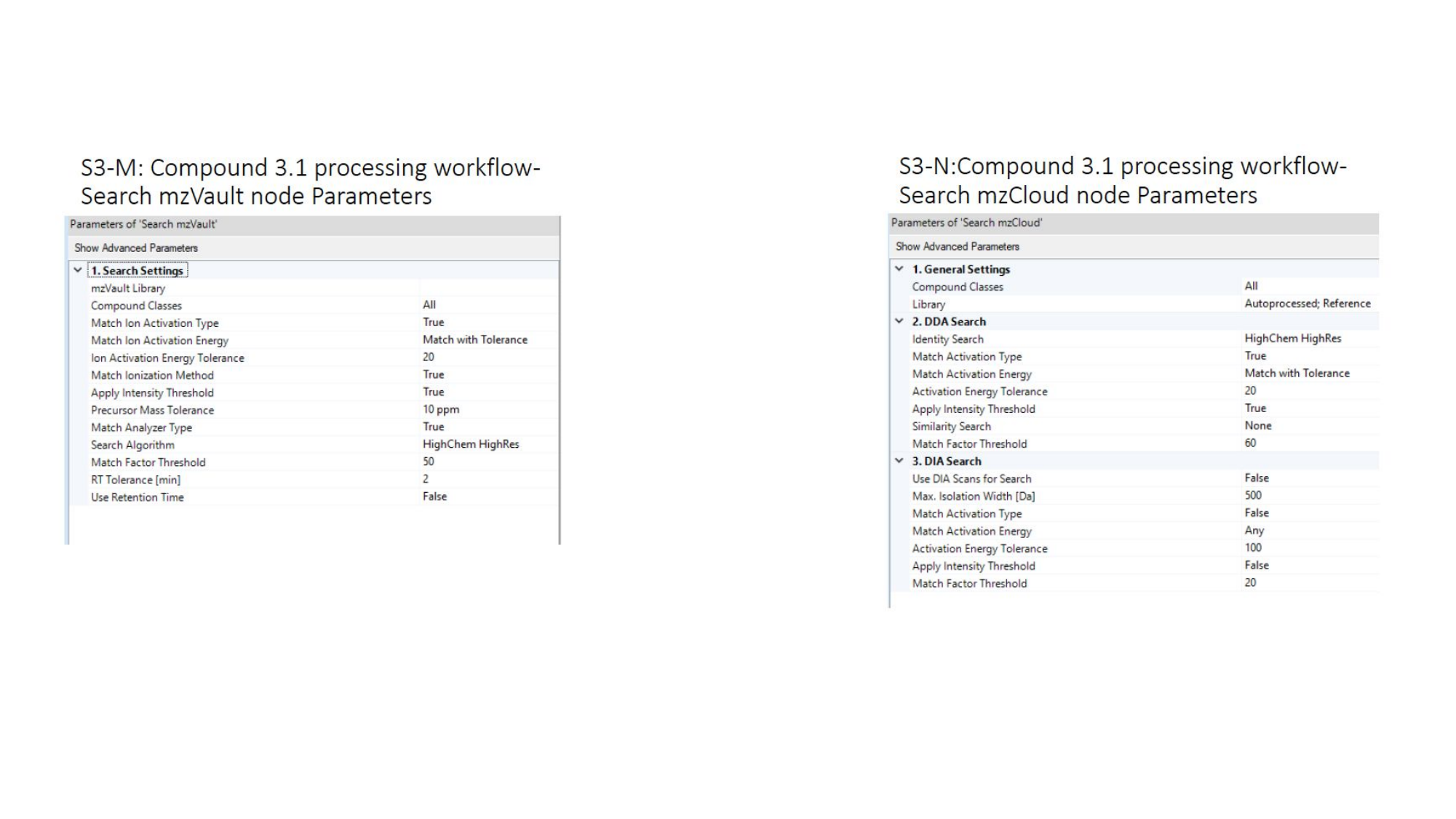

## Slide 16
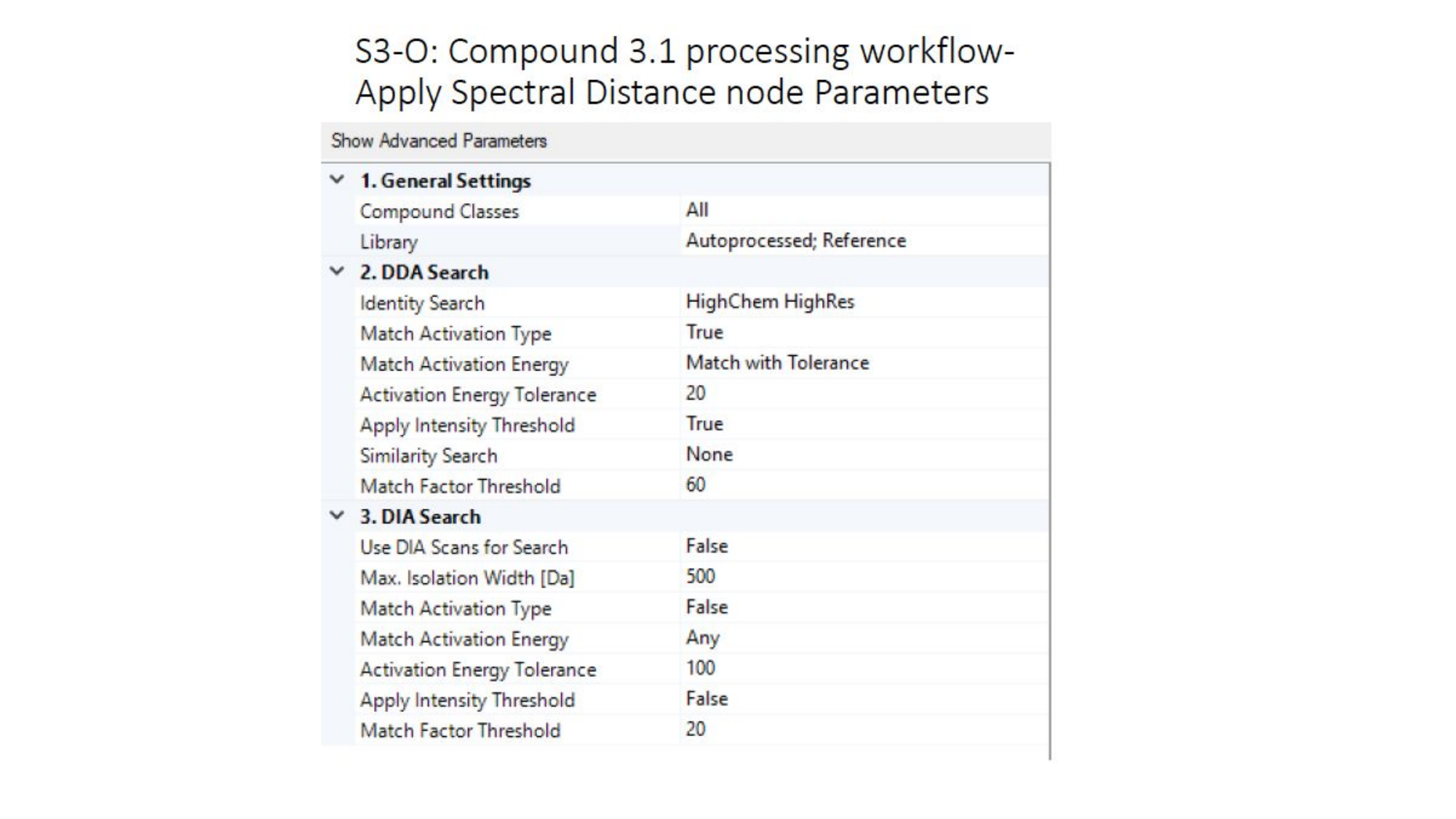

## Slide 17
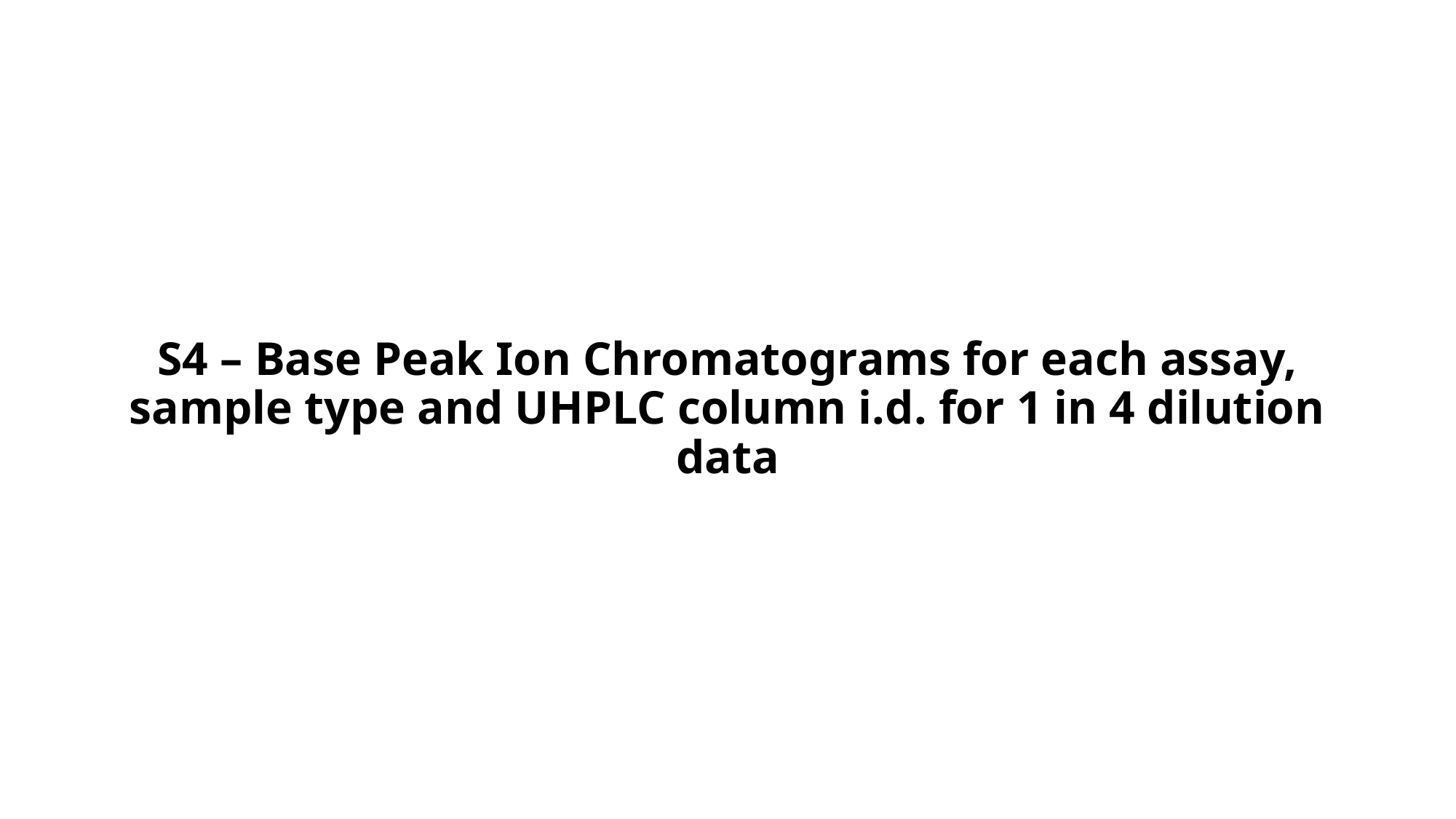

# S4 – Base Peak Ion Chromatograms for each assay, sample type and UHPLC column i.d. for 1 in 4 dilution data

## Slide 18
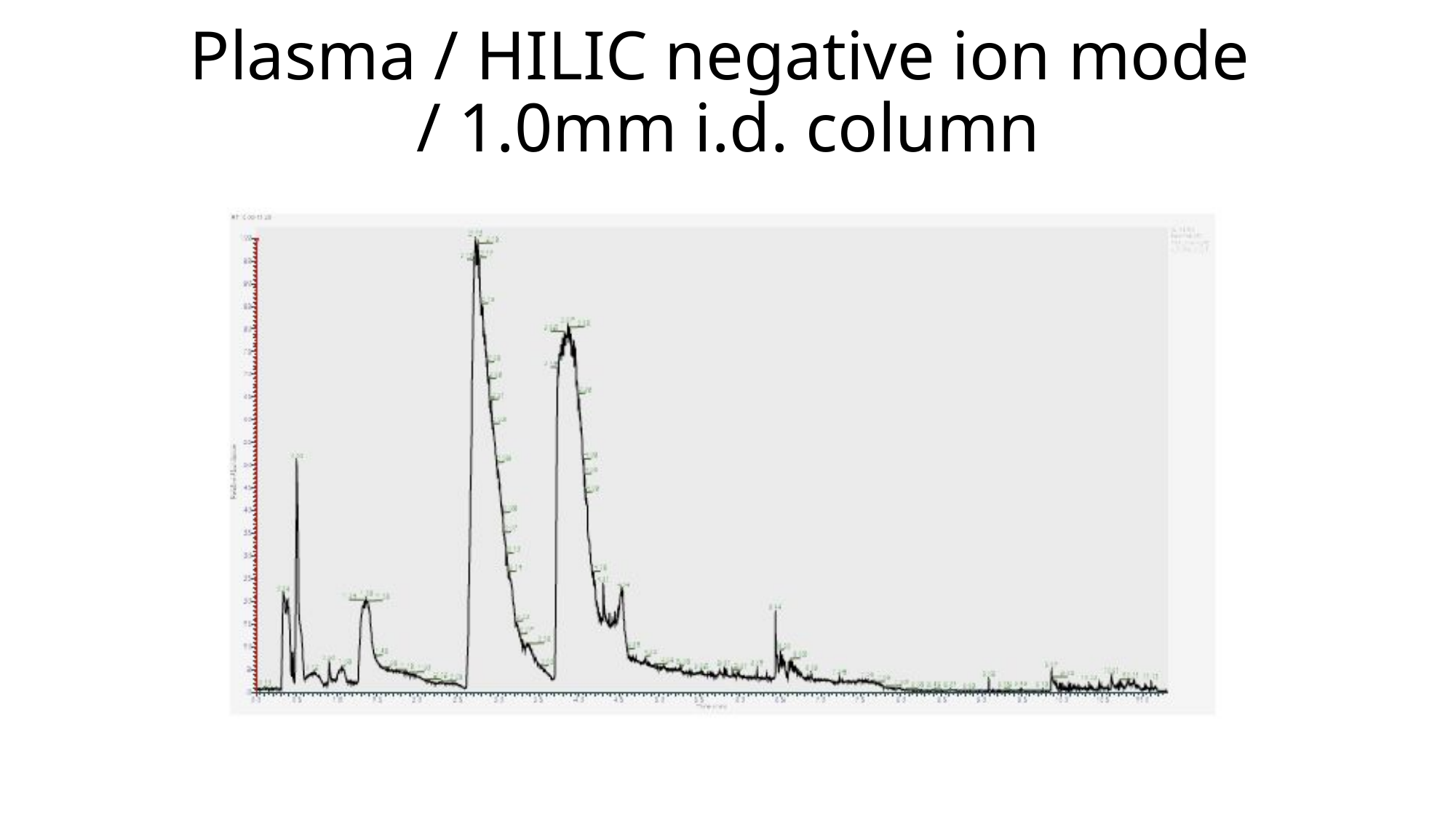

# Plasma / HILIC negative ion mode / 1.0mm i.d. column

## Slide 19
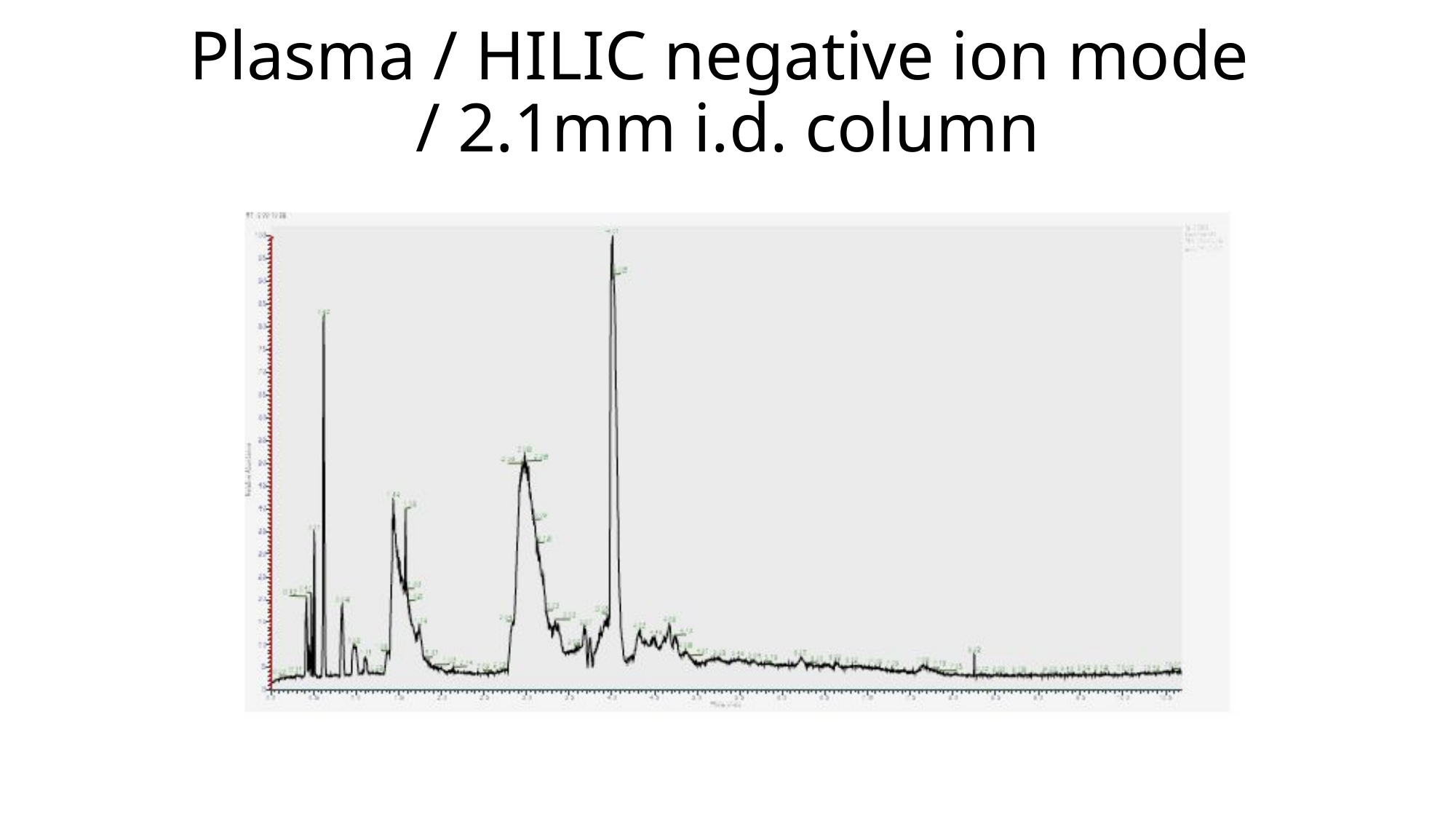

# Plasma / HILIC negative ion mode / 2.1mm i.d. column

## Slide 20
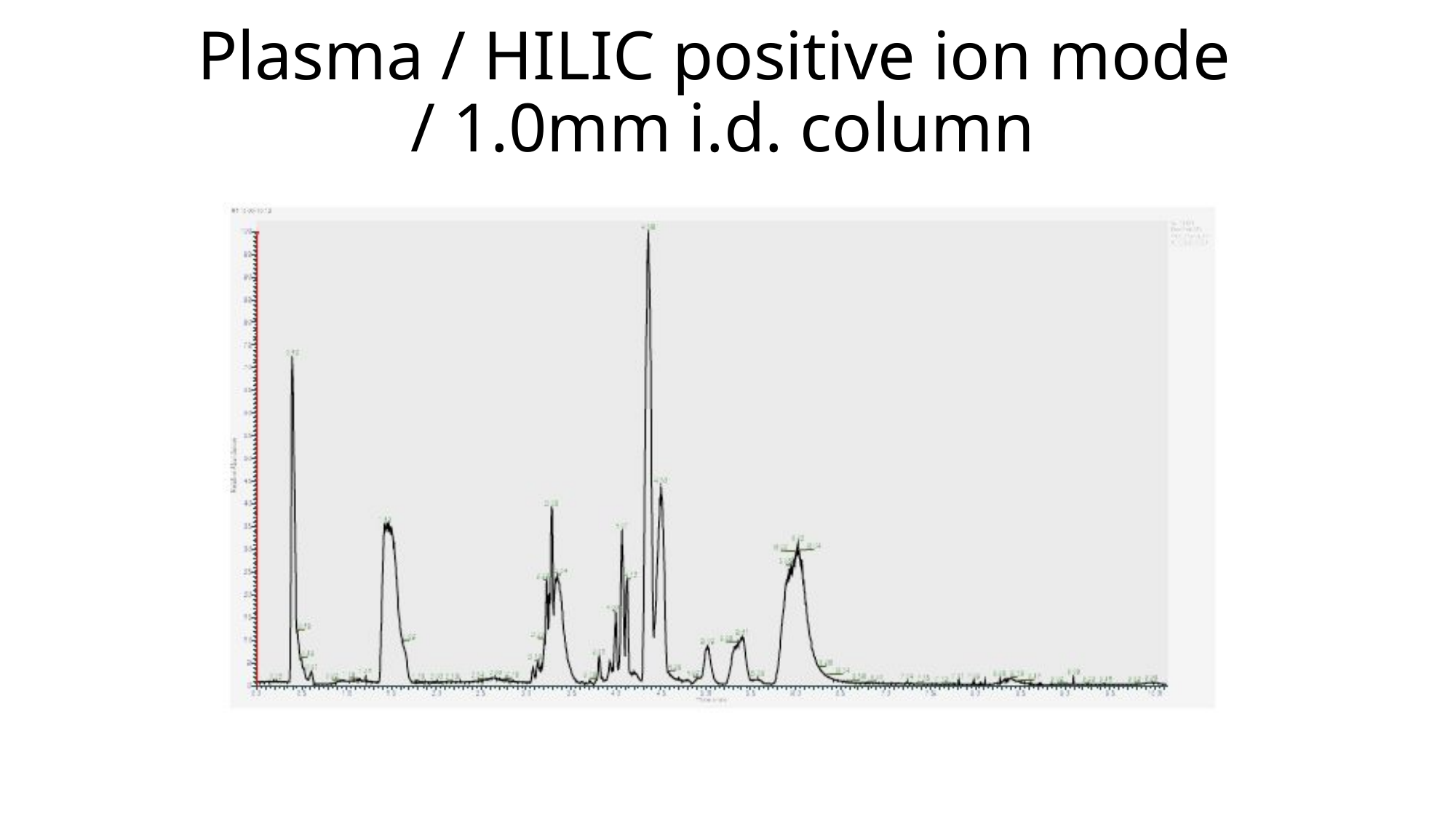

# Plasma / HILIC positive ion mode / 1.0mm i.d. column

## Slide 21
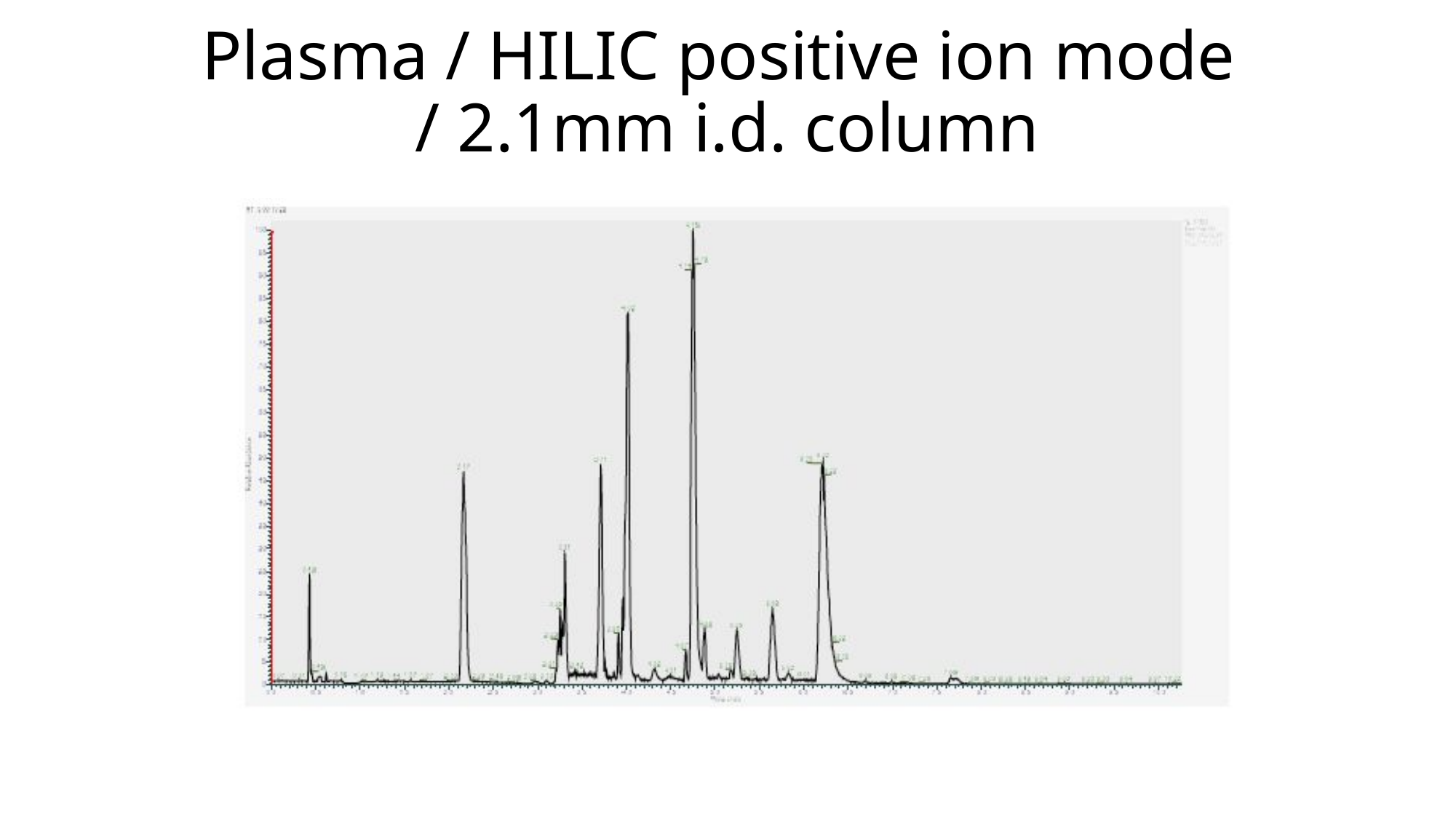

# Plasma / HILIC positive ion mode / 2.1mm i.d. column

## Slide 22
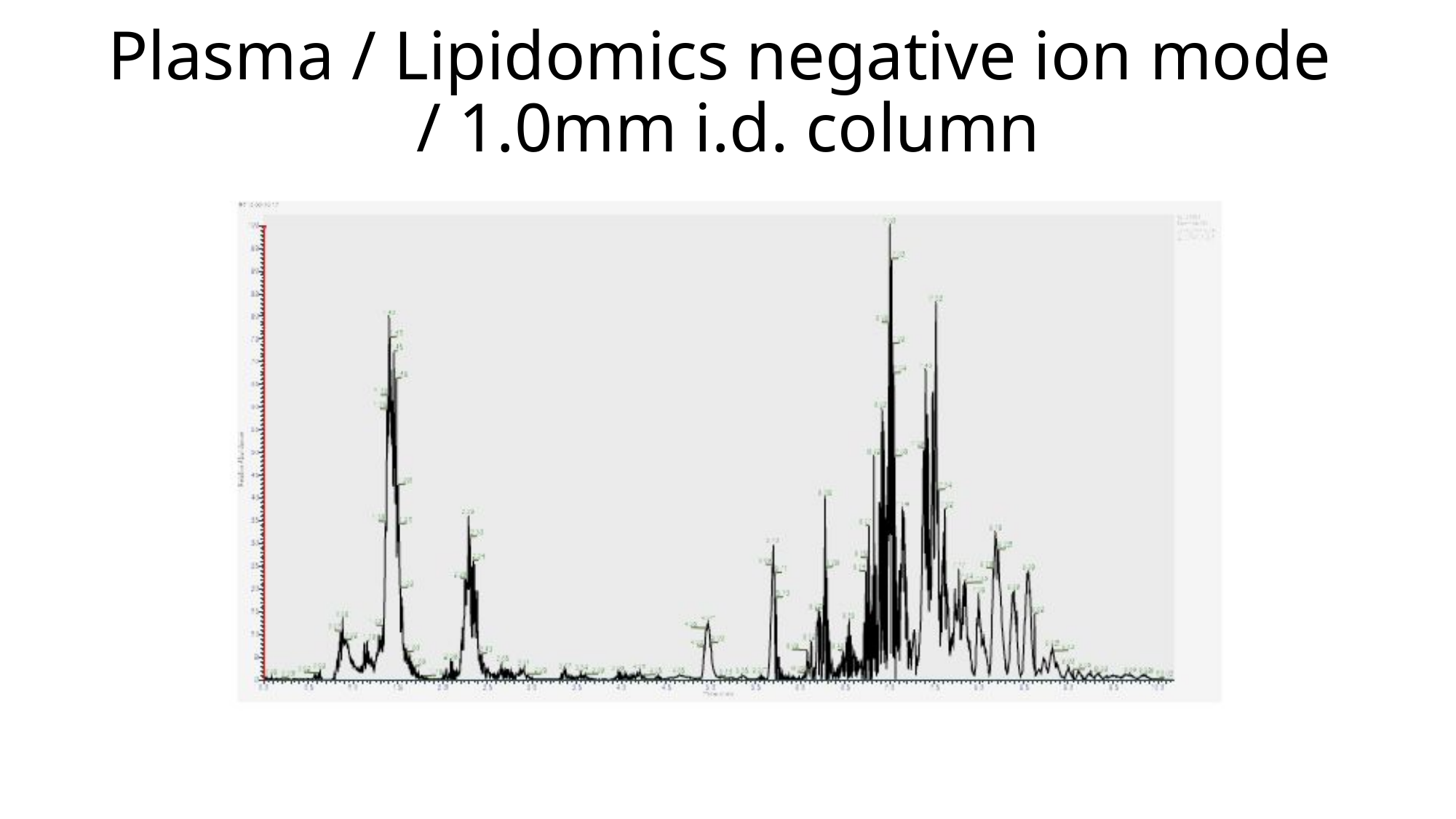

# Plasma / Lipidomics negative ion mode / 1.0mm i.d. column

## Slide 23
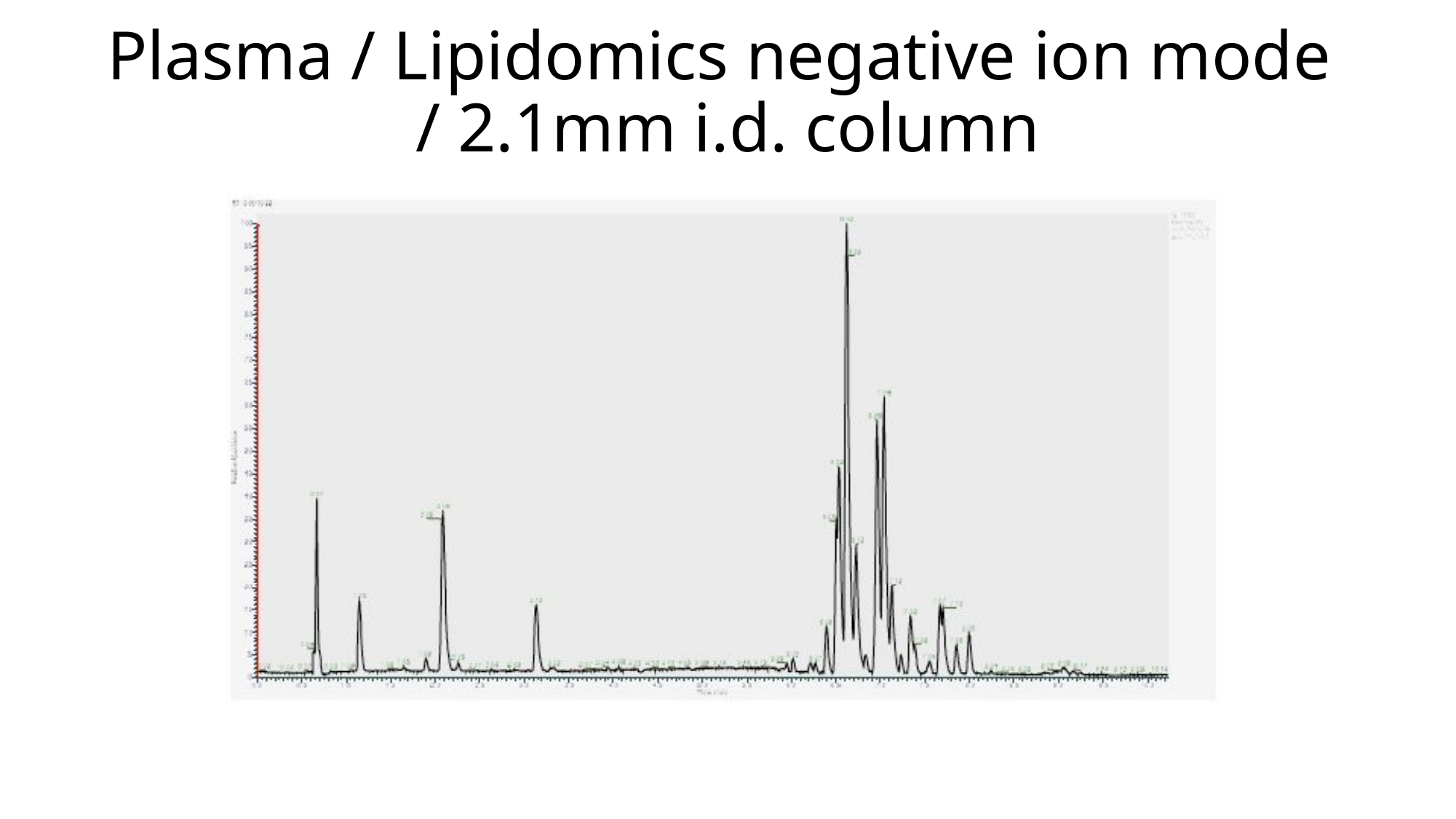

# Plasma / Lipidomics negative ion mode / 2.1mm i.d. column

## Slide 24
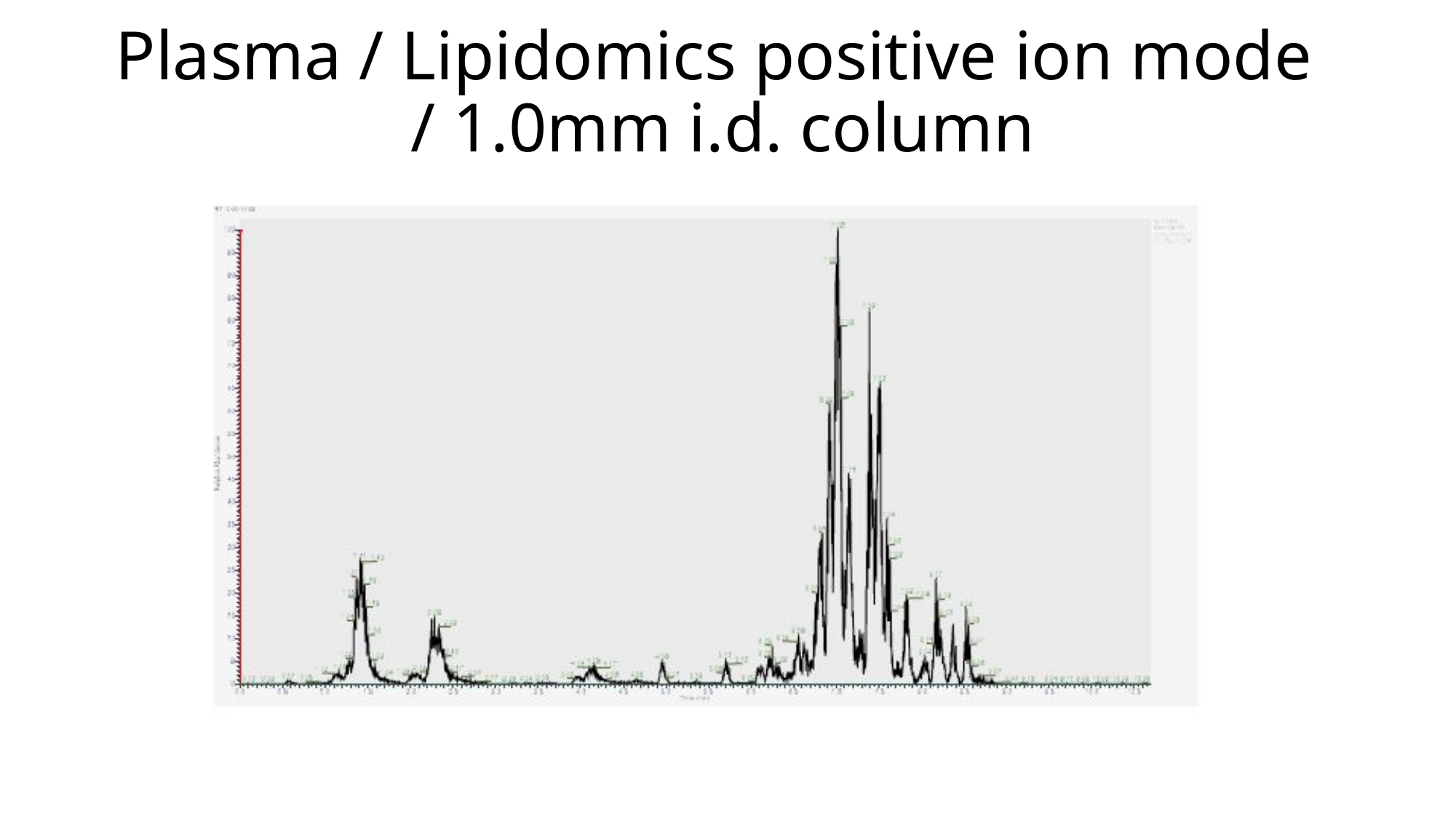

# Plasma / Lipidomics positive ion mode / 1.0mm i.d. column

## Slide 25
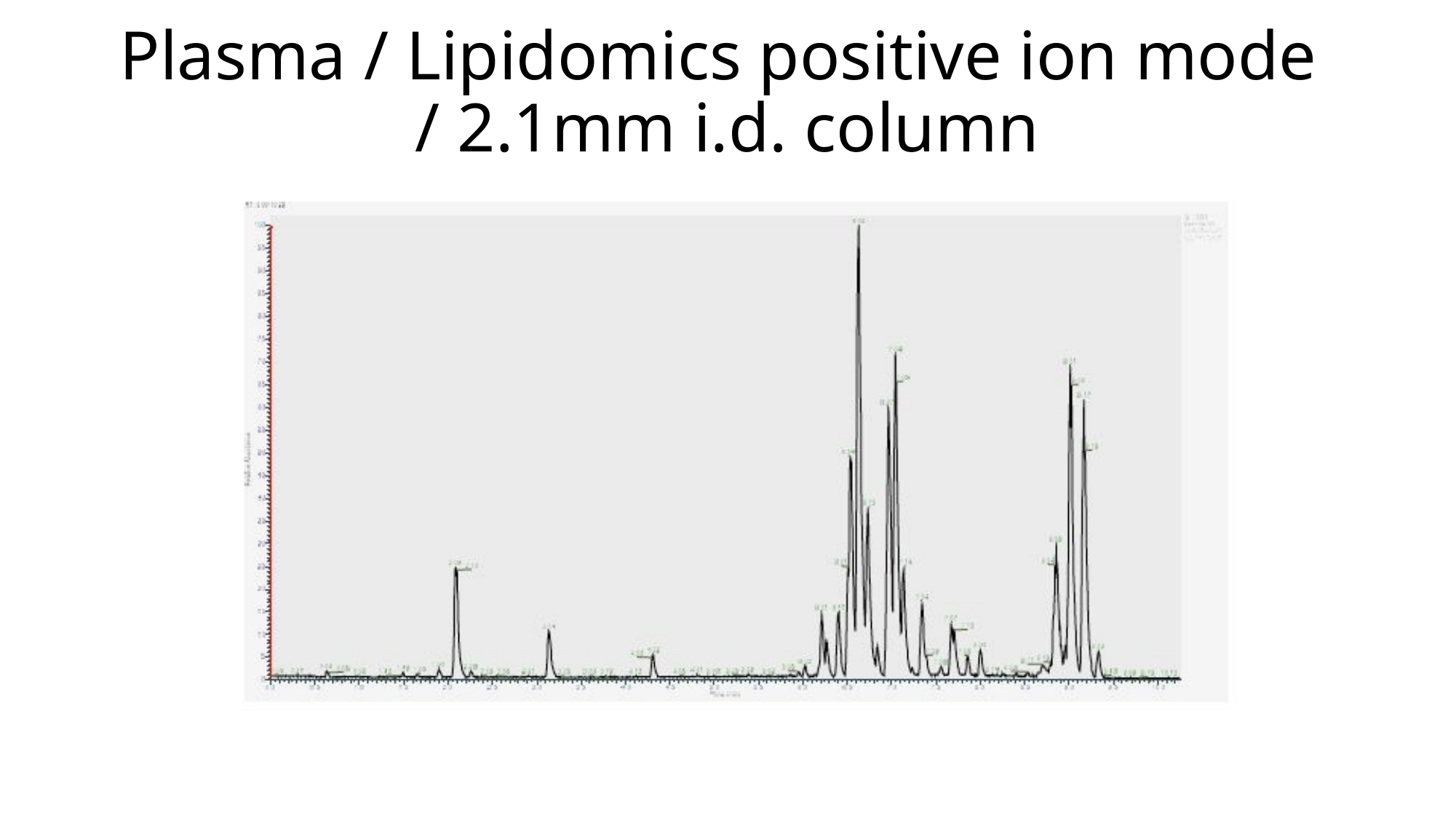

# Plasma / Lipidomics positive ion mode / 2.1mm i.d. column

## Slide 26
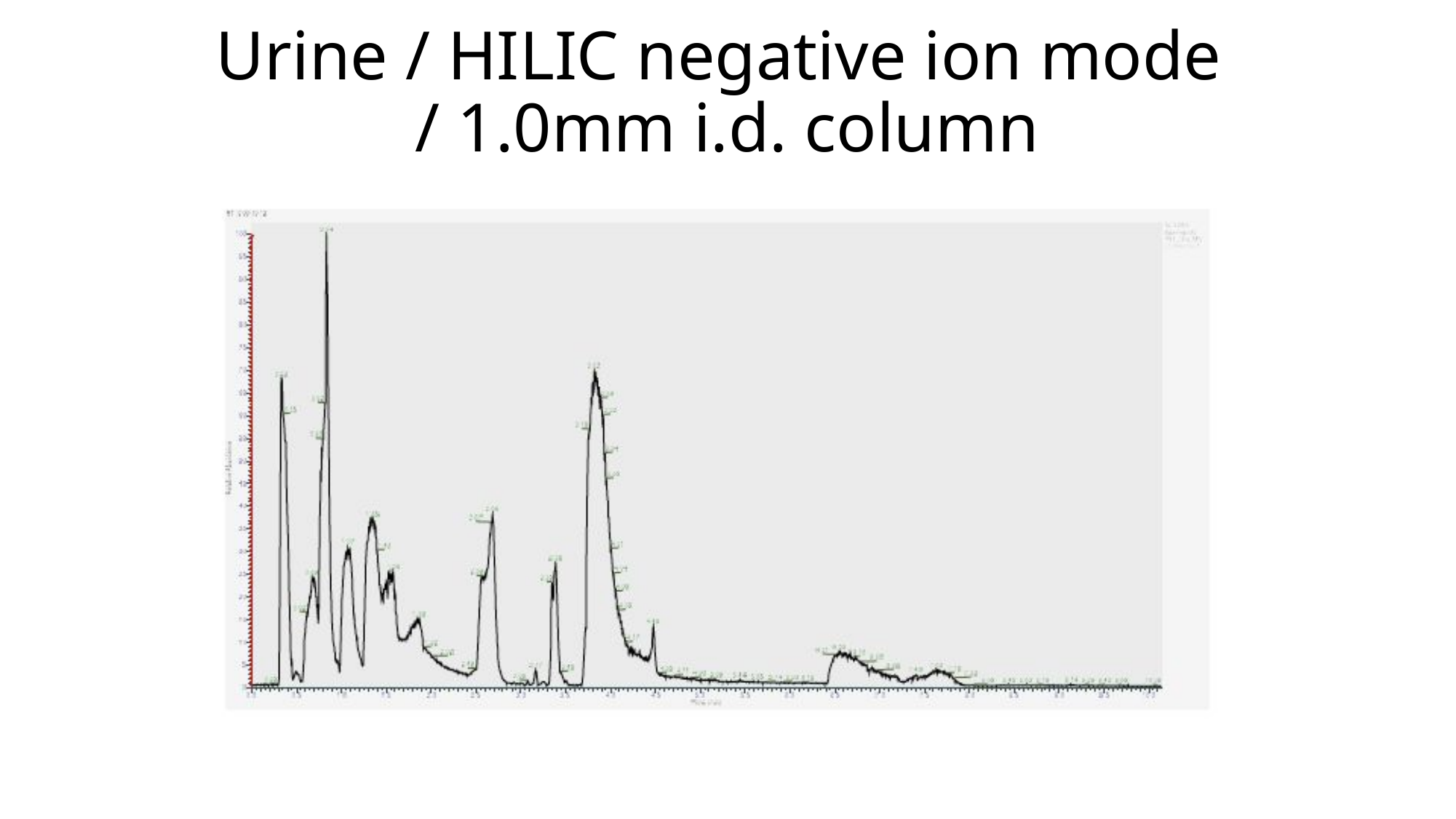

# Urine / HILIC negative ion mode / 1.0mm i.d. column

## Slide 27
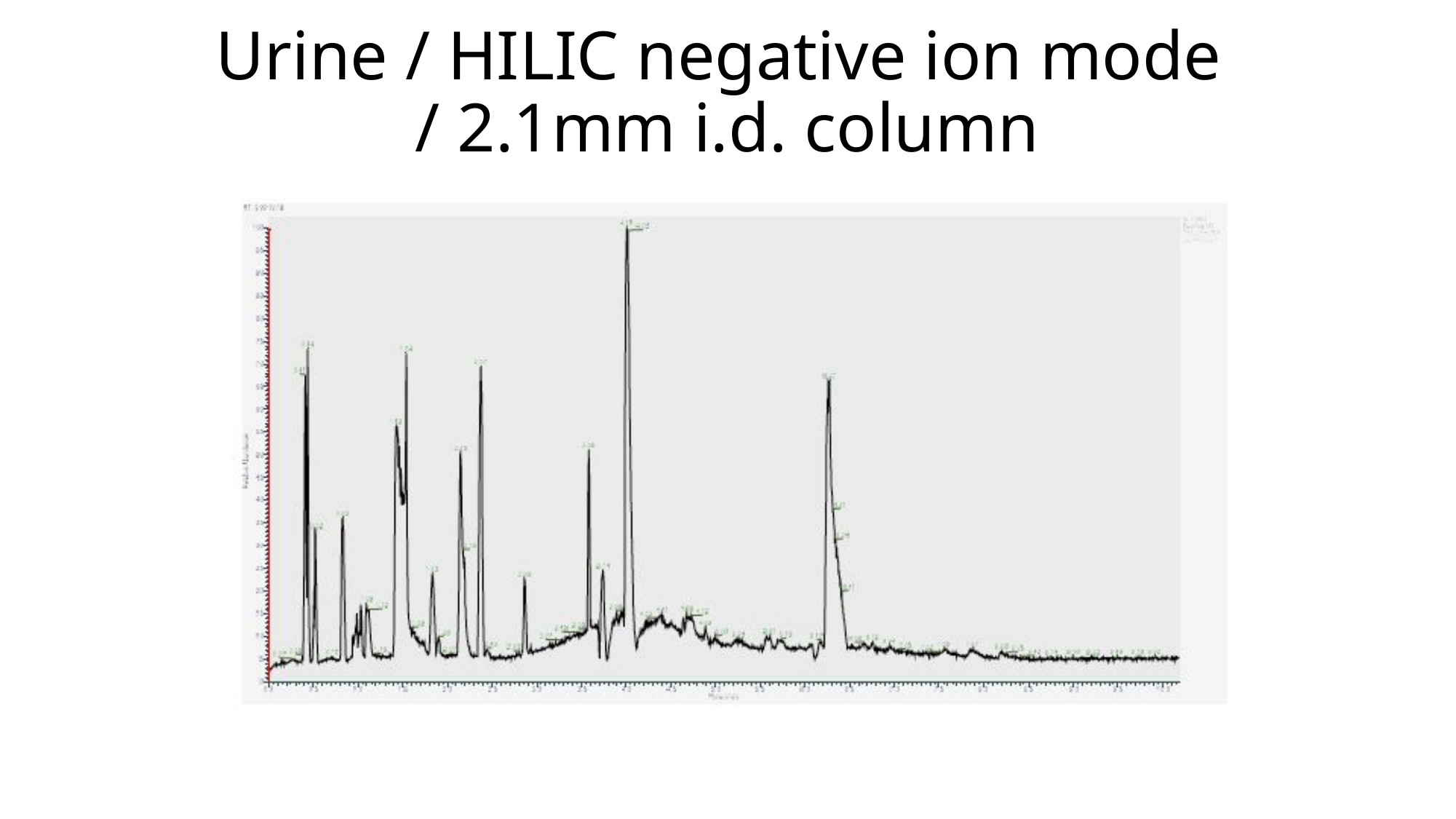

# Urine / HILIC negative ion mode / 2.1mm i.d. column

## Slide 28
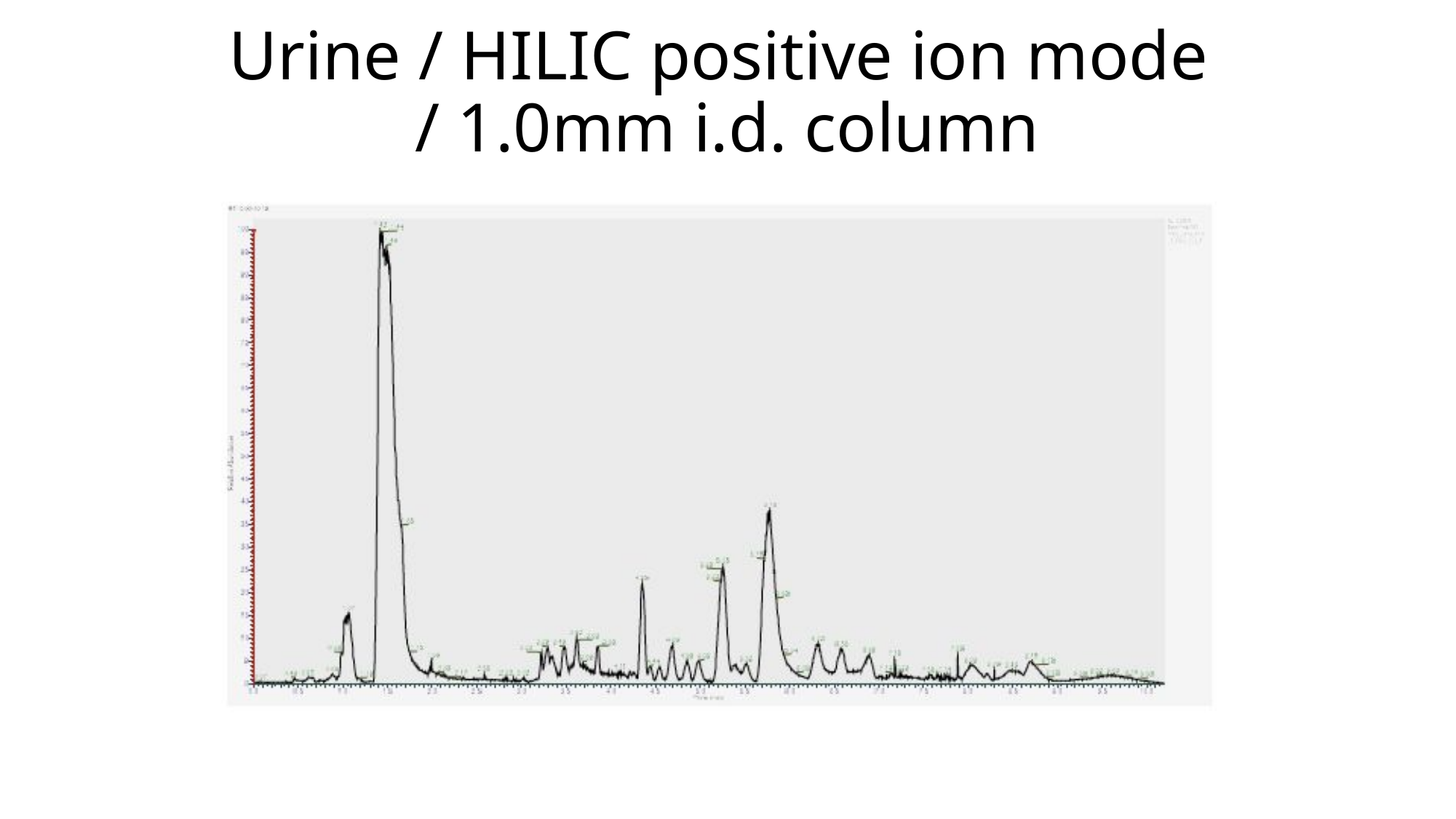

# Urine / HILIC positive ion mode / 1.0mm i.d. column

## Slide 29
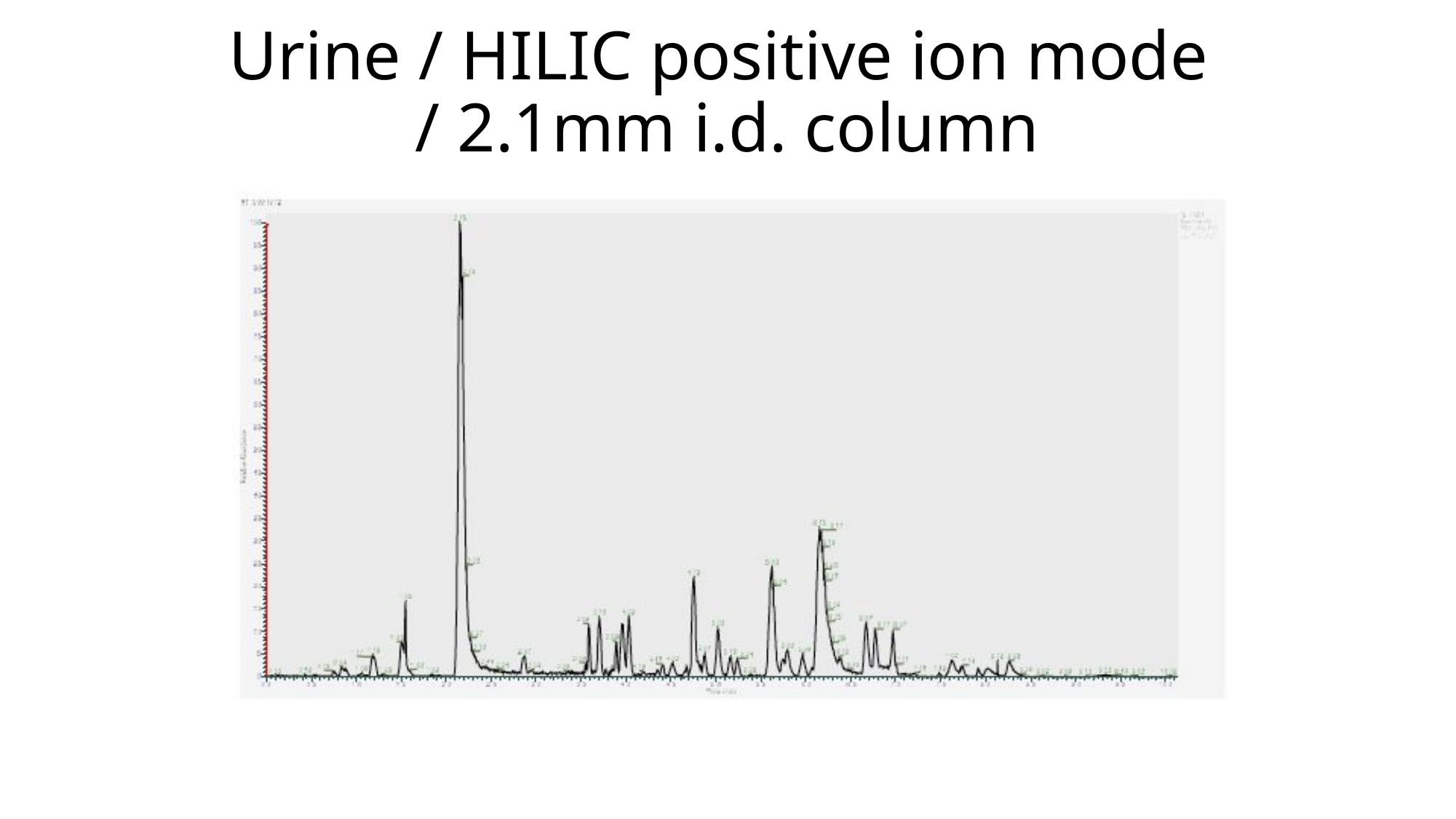

# Urine / HILIC positive ion mode / 2.1mm i.d. column

## Slide 30
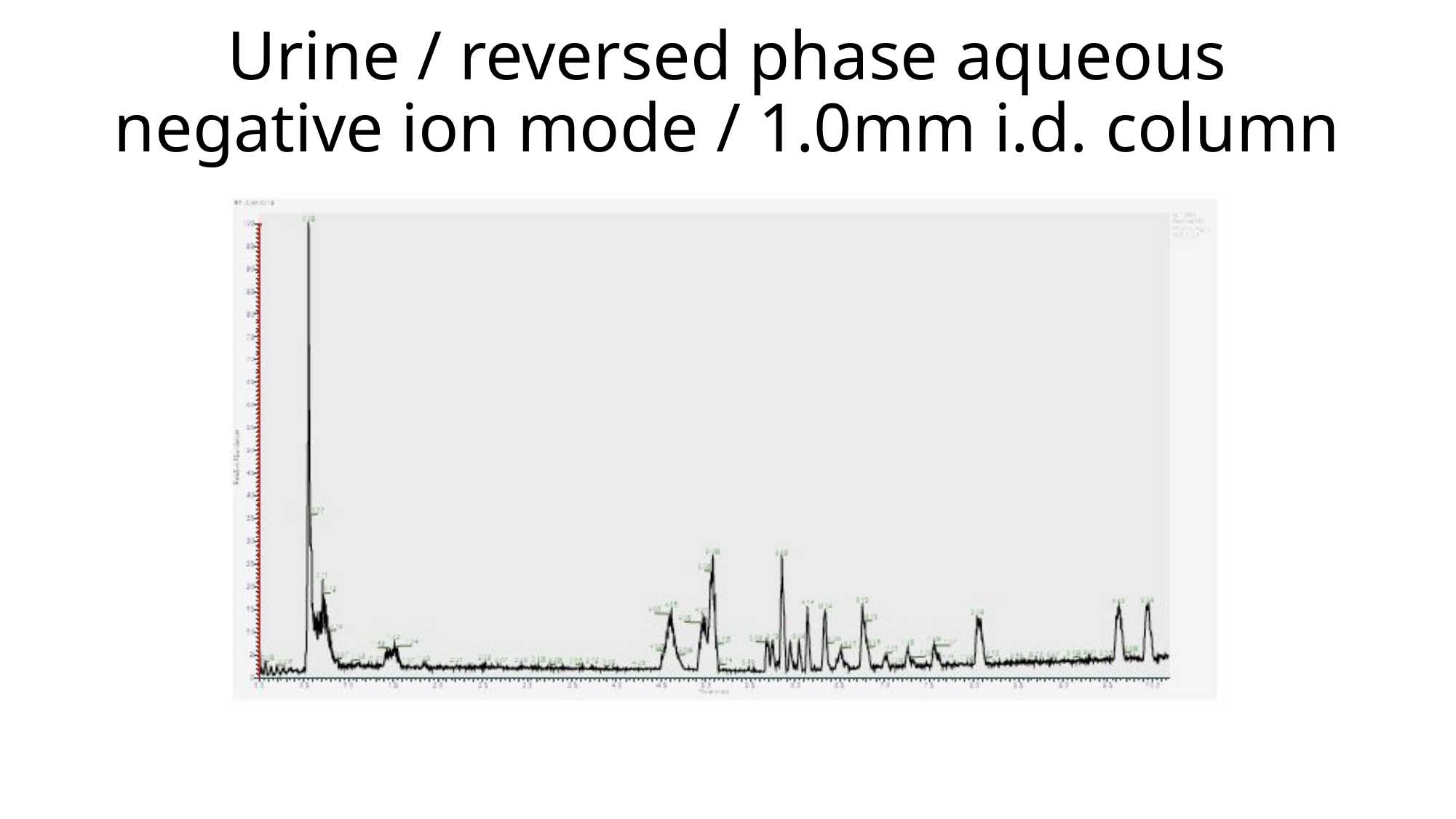

# Urine / reversed phase aqueous negative ion mode / 1.0mm i.d. column

## Slide 31
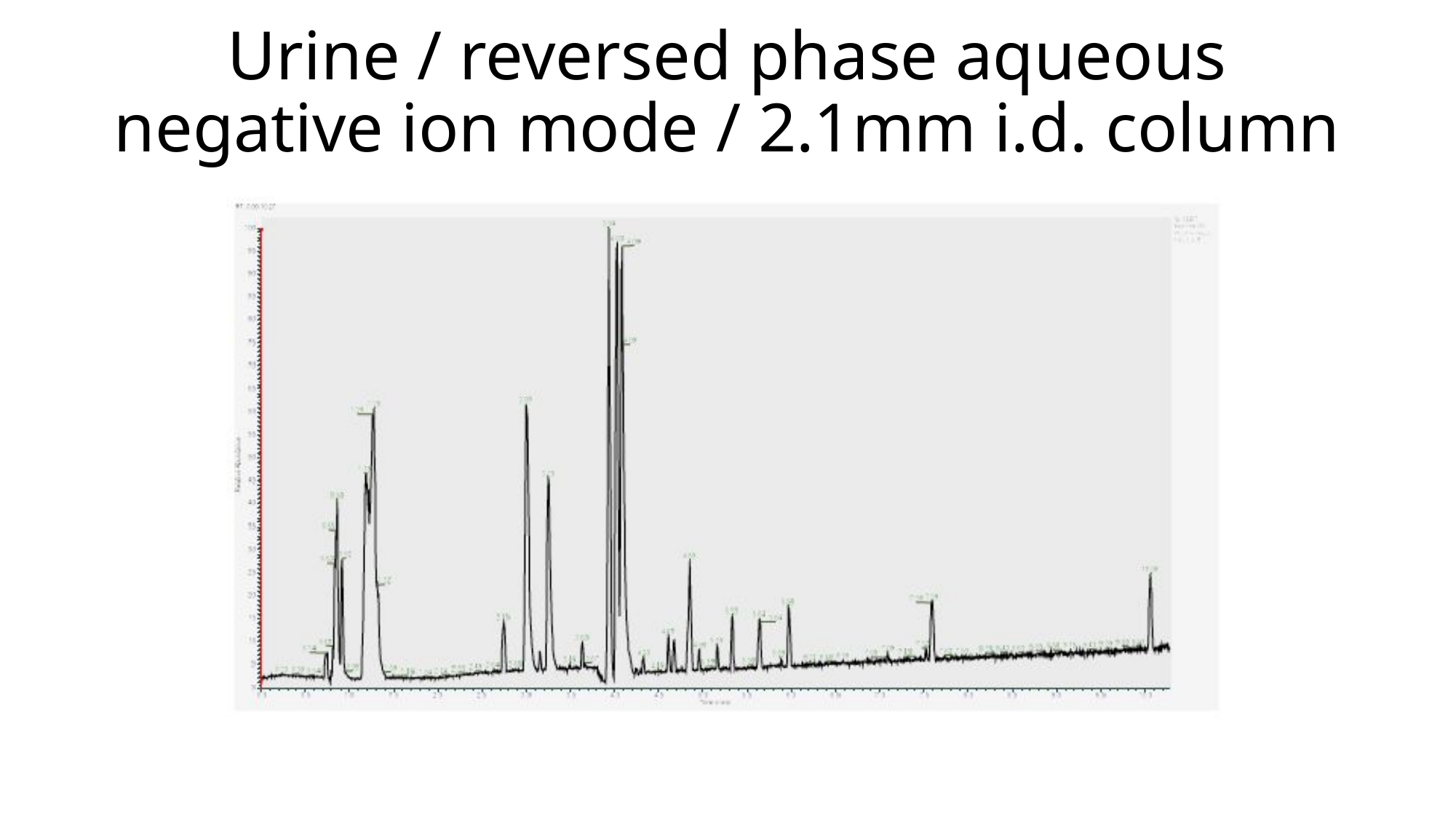

# Urine / reversed phase aqueous negative ion mode / 2.1mm i.d. column

## Slide 32
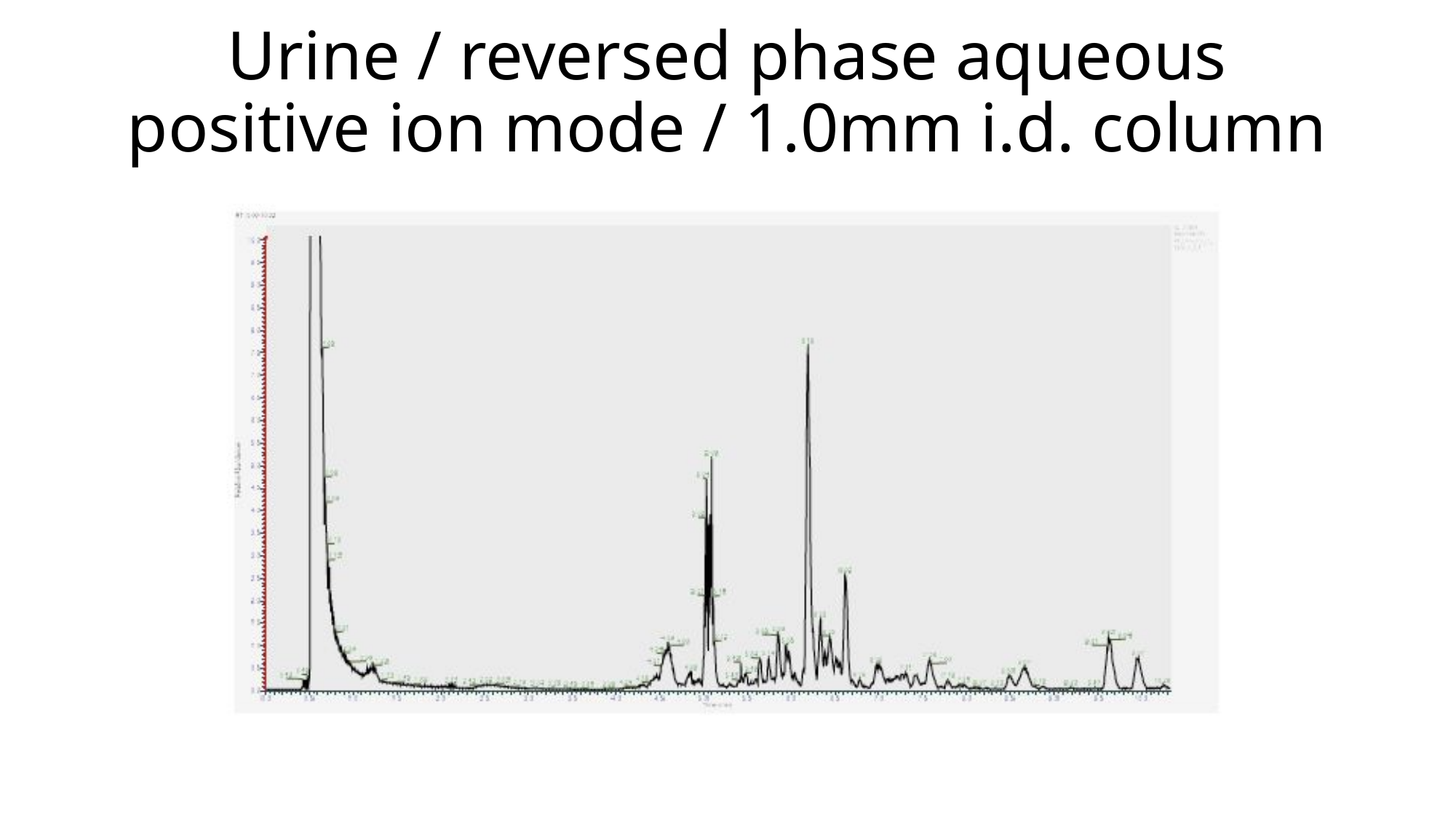

# Urine / reversed phase aqueous positive ion mode / 1.0mm i.d. column

## Slide 33
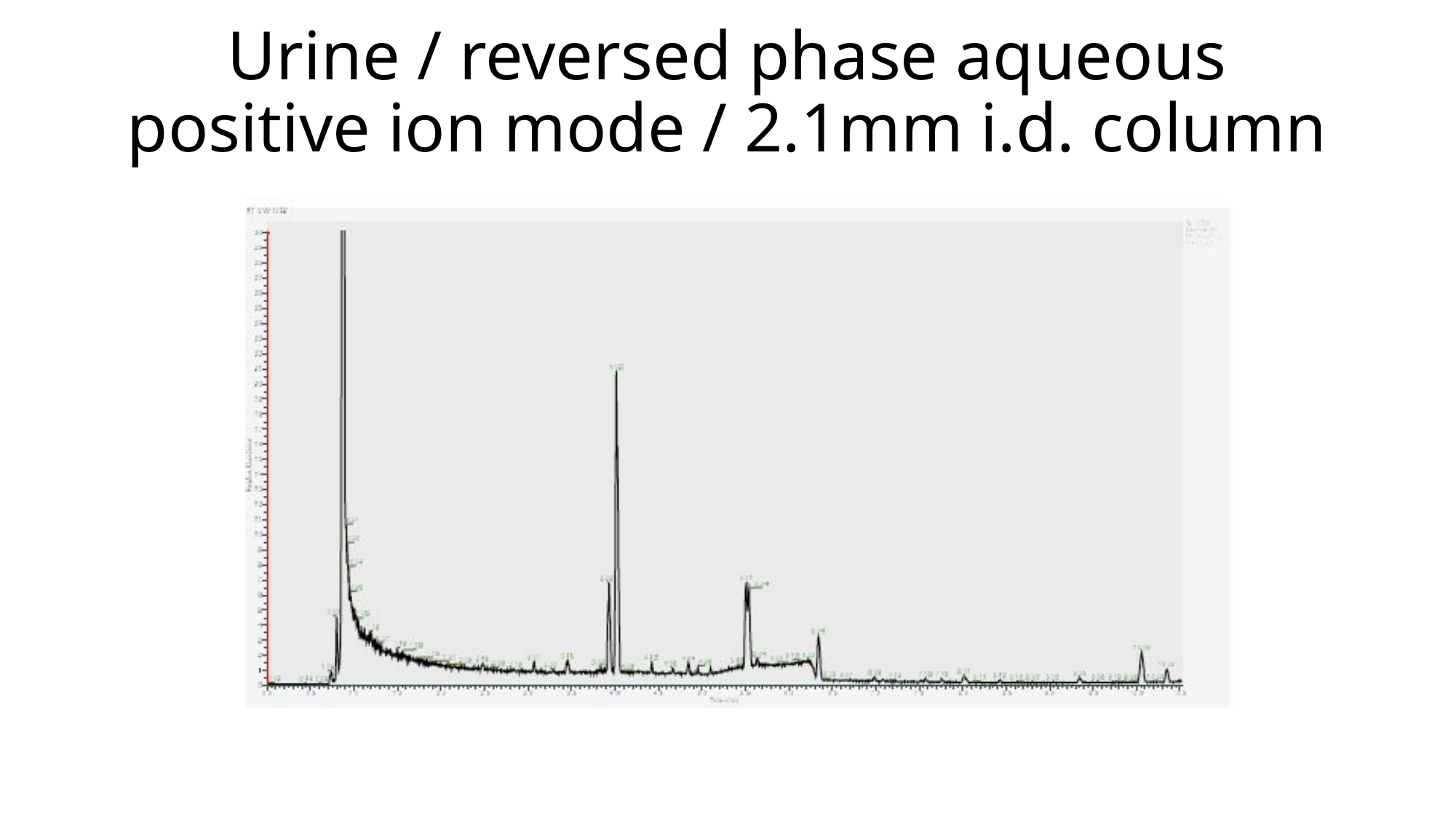

# Urine / reversed phase aqueous positive ion mode / 2.1mm i.d. column

## Slide 34
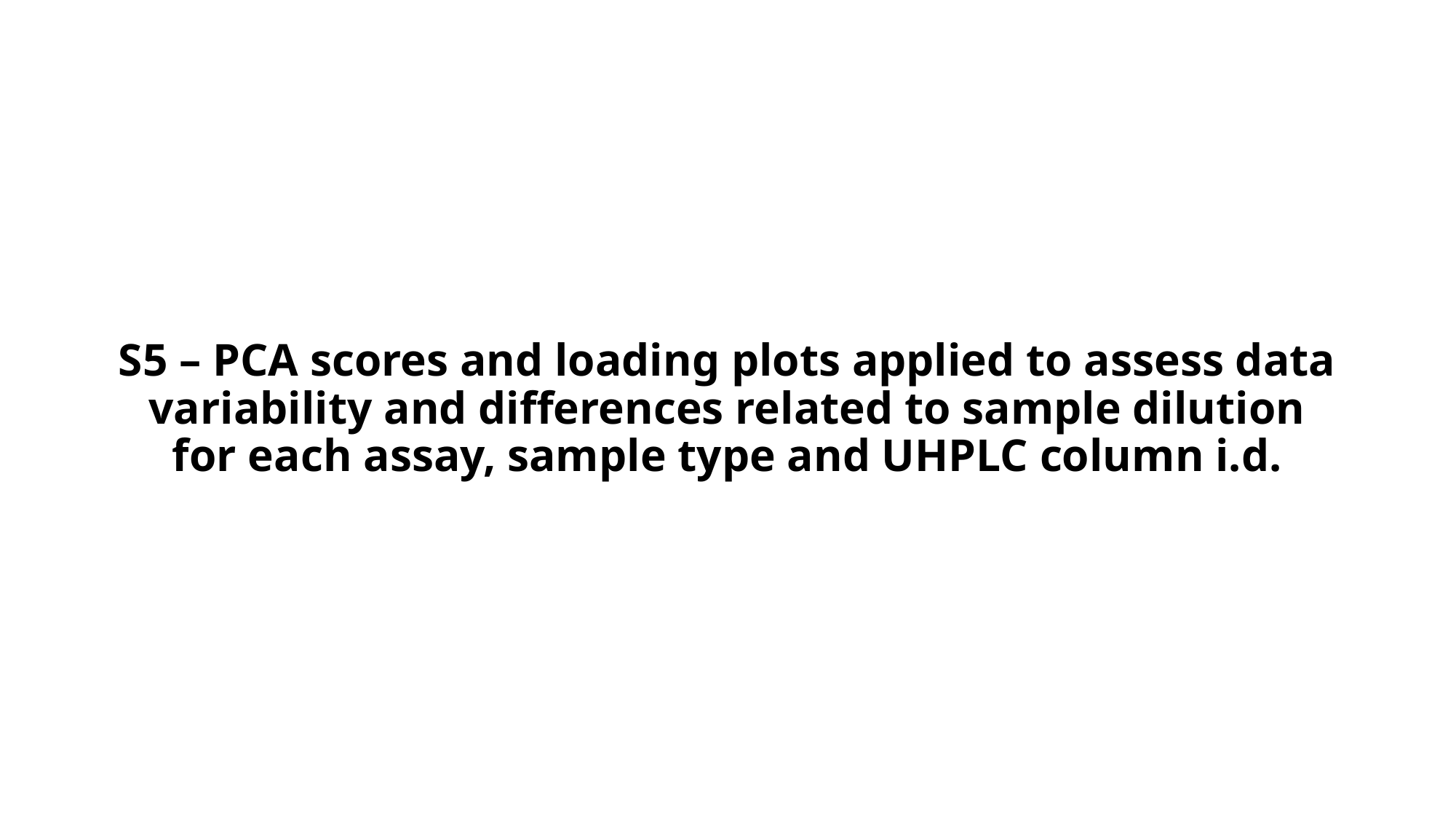

# S5 – PCA scores and loading plots applied to assess data variability and differences related to sample dilution for each assay, sample type and UHPLC column i.d.

## Slide 35
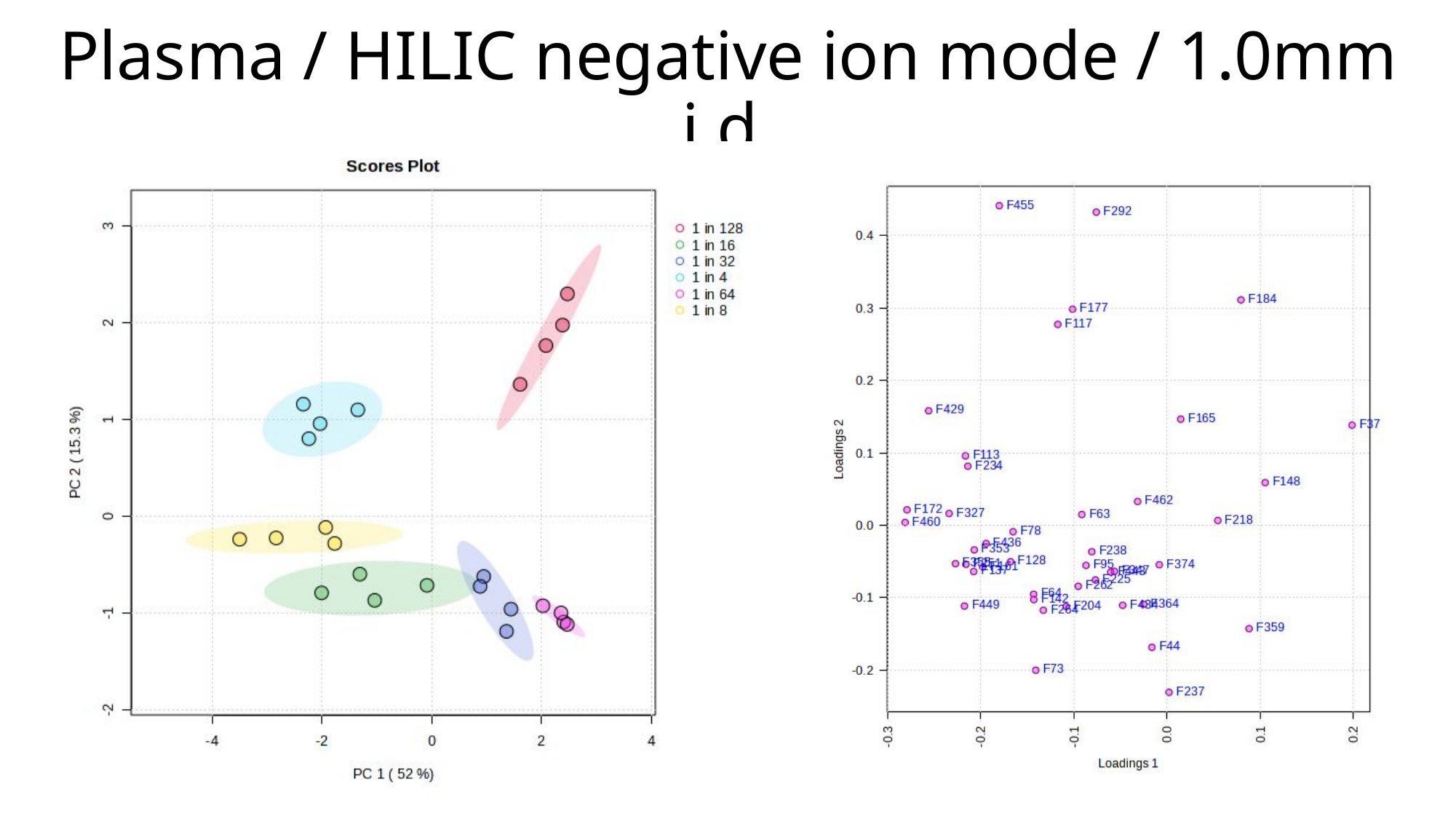

# Plasma / HILIC negative ion mode / 1.0mm i.d.

## Slide 36
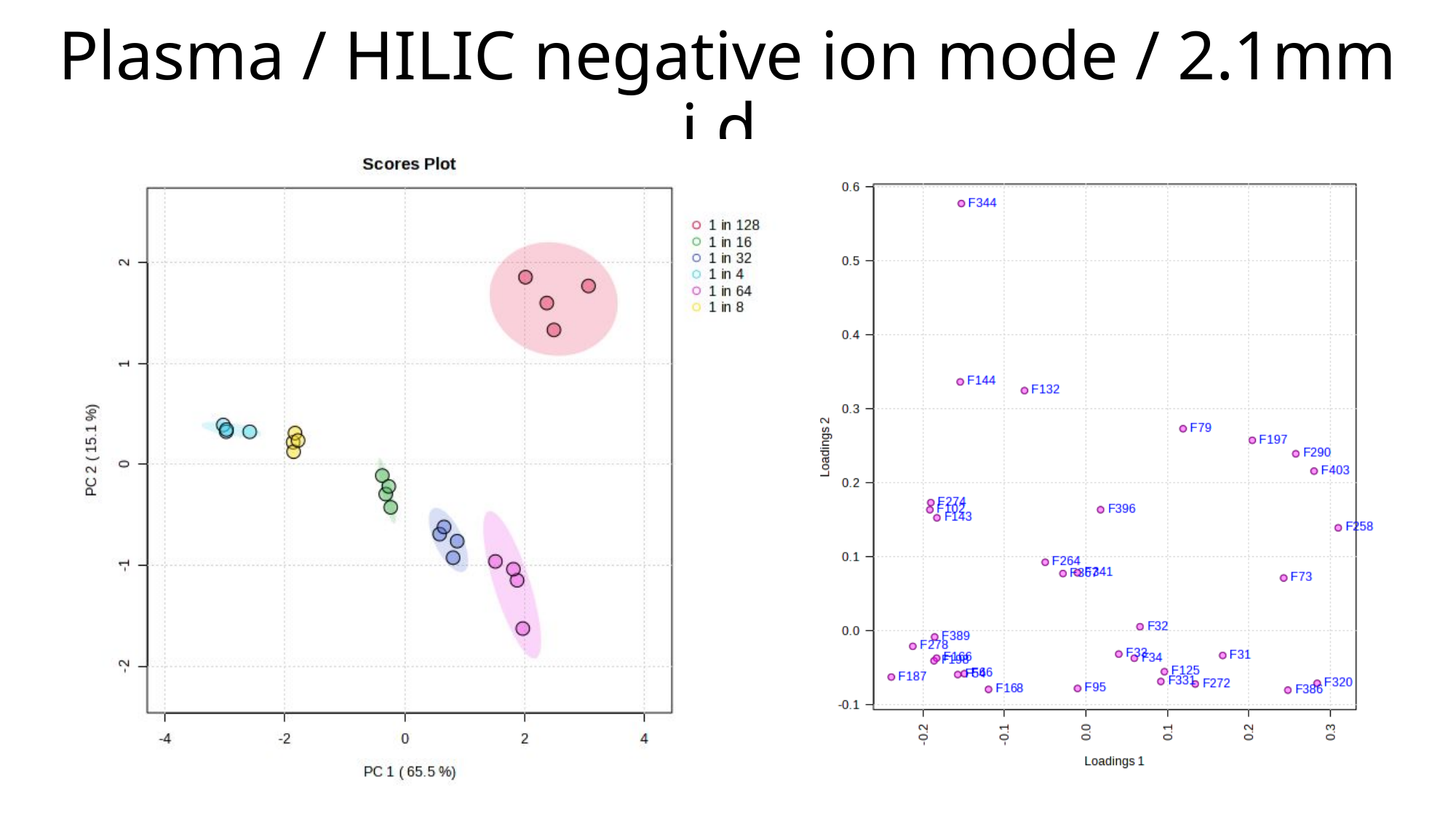

# Plasma / HILIC negative ion mode / 2.1mm i.d.

## Slide 37
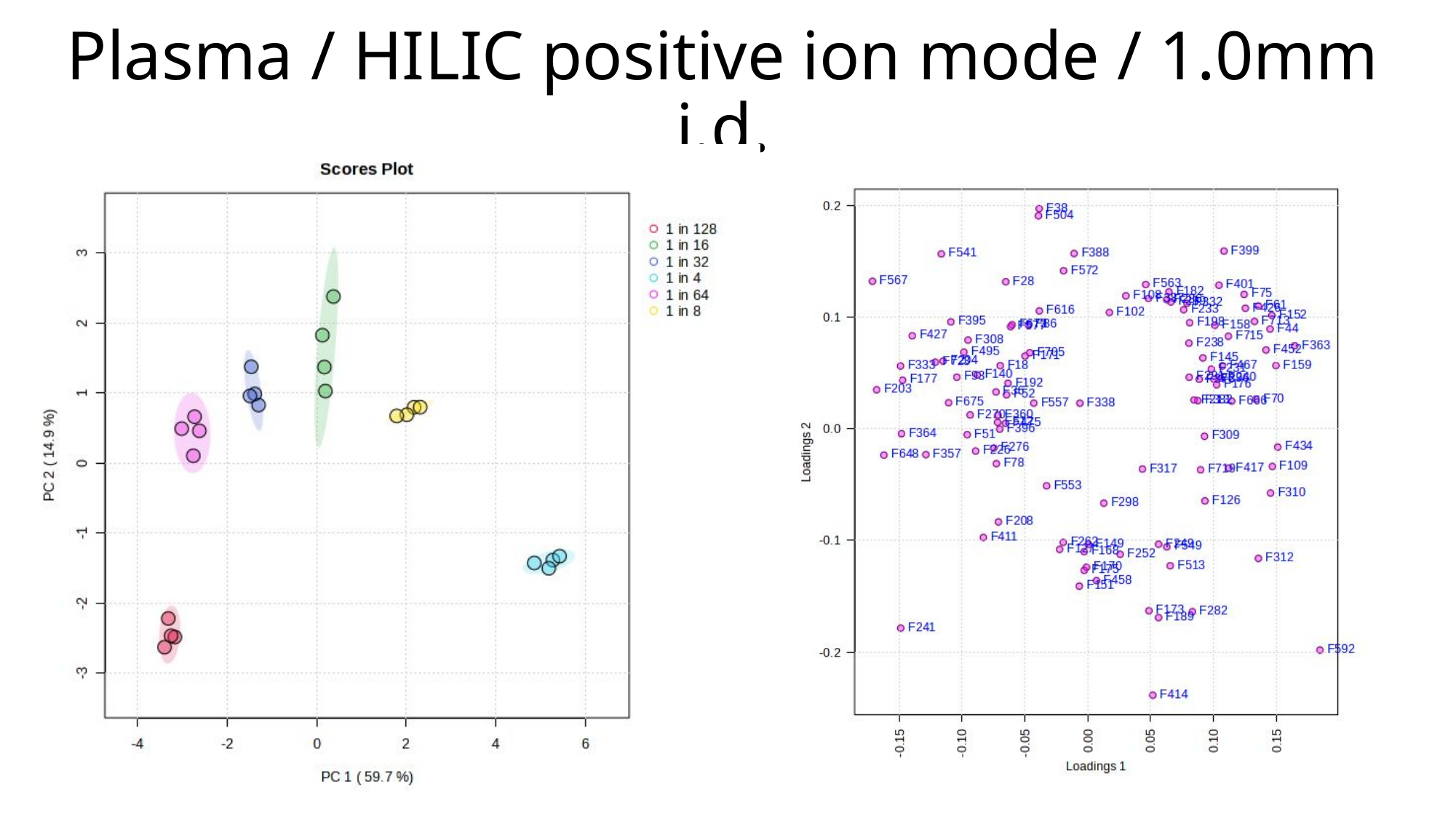

# Plasma / HILIC positive ion mode / 1.0mm i.d.

## Slide 38
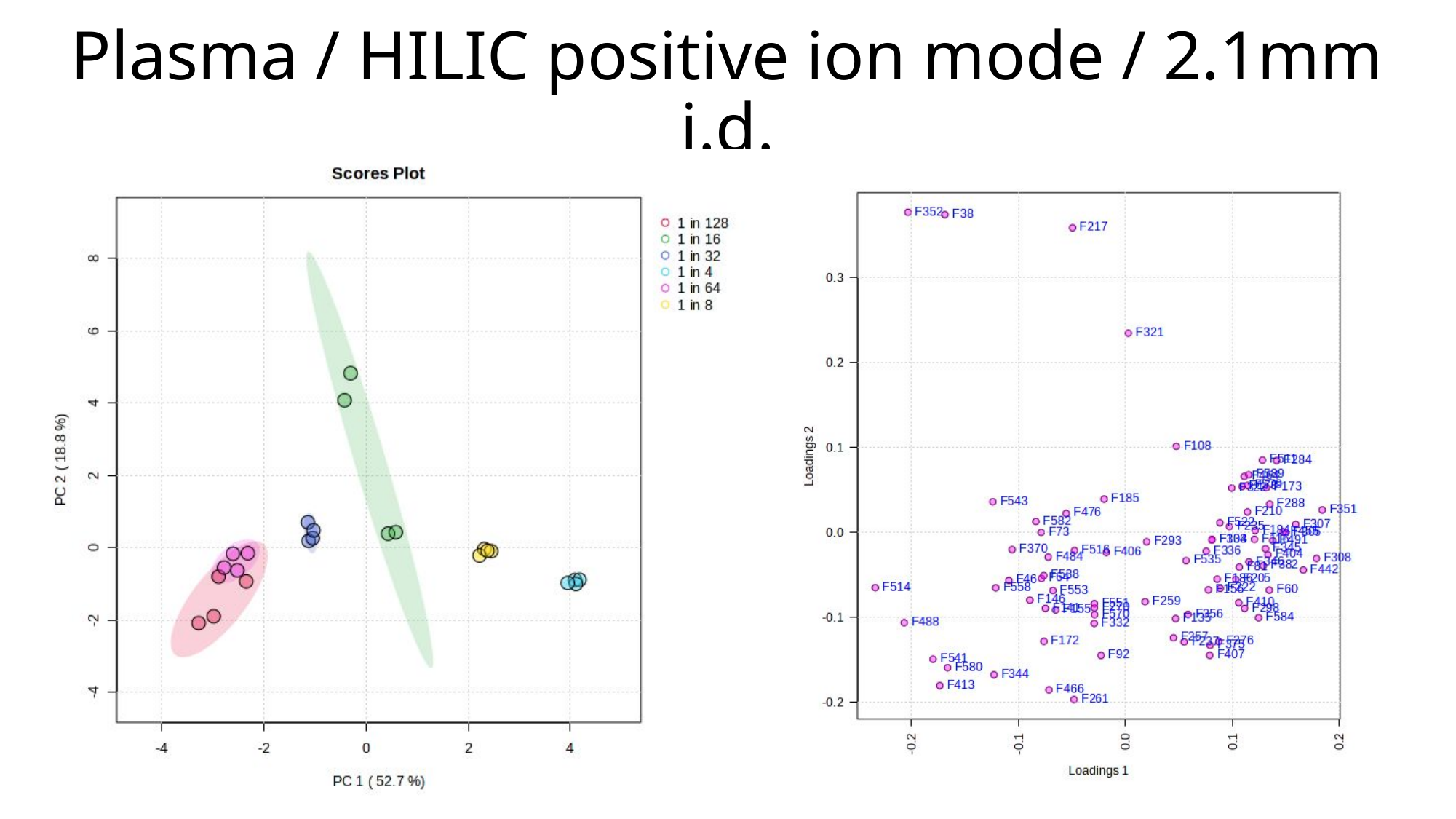

# Plasma / HILIC positive ion mode / 2.1mm i.d.

## Slide 39
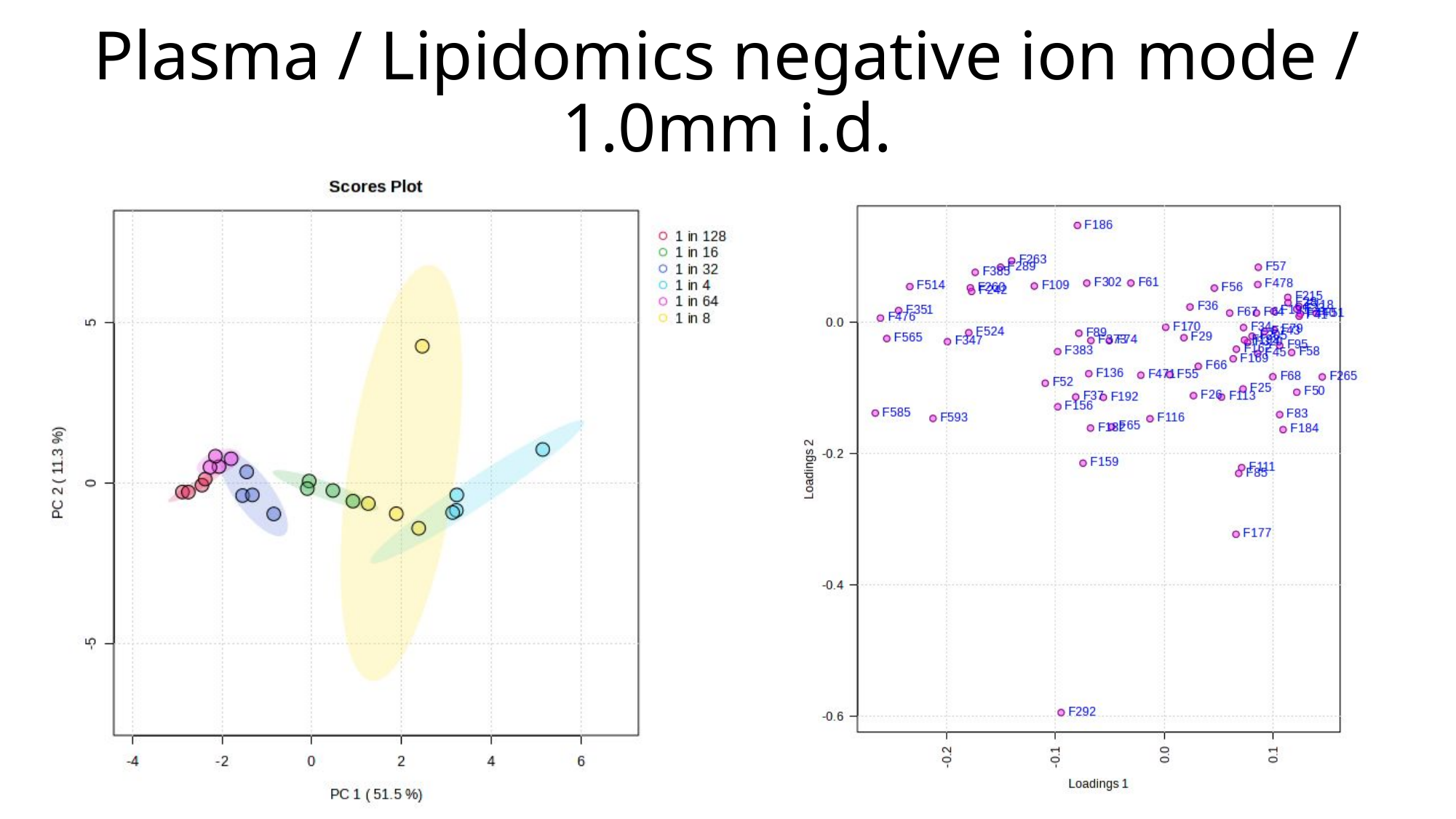

# Plasma / Lipidomics negative ion mode / 1.0mm i.d.

## Slide 40
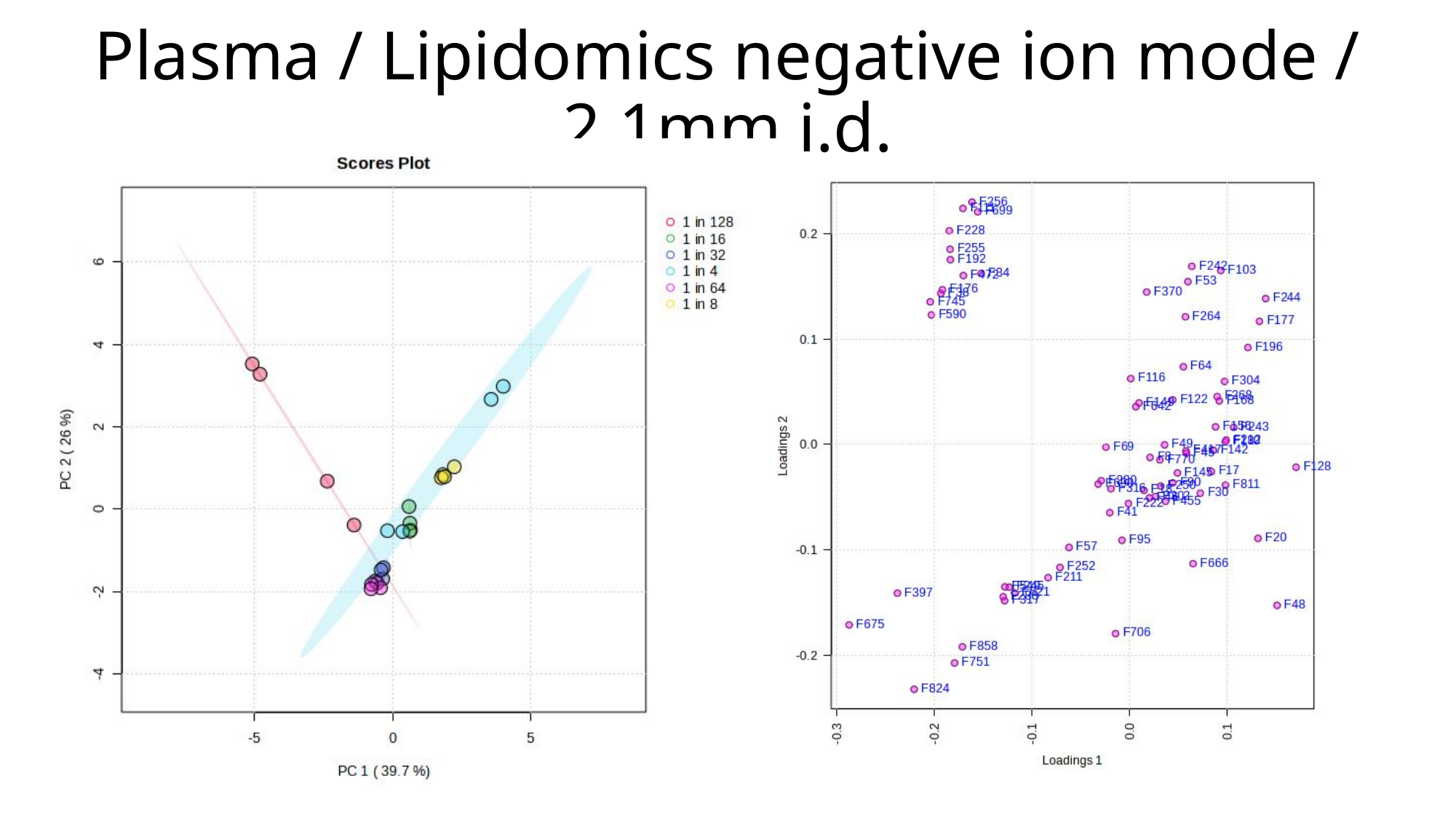

# Plasma / Lipidomics negative ion mode / 2.1mm i.d.

## Slide 41
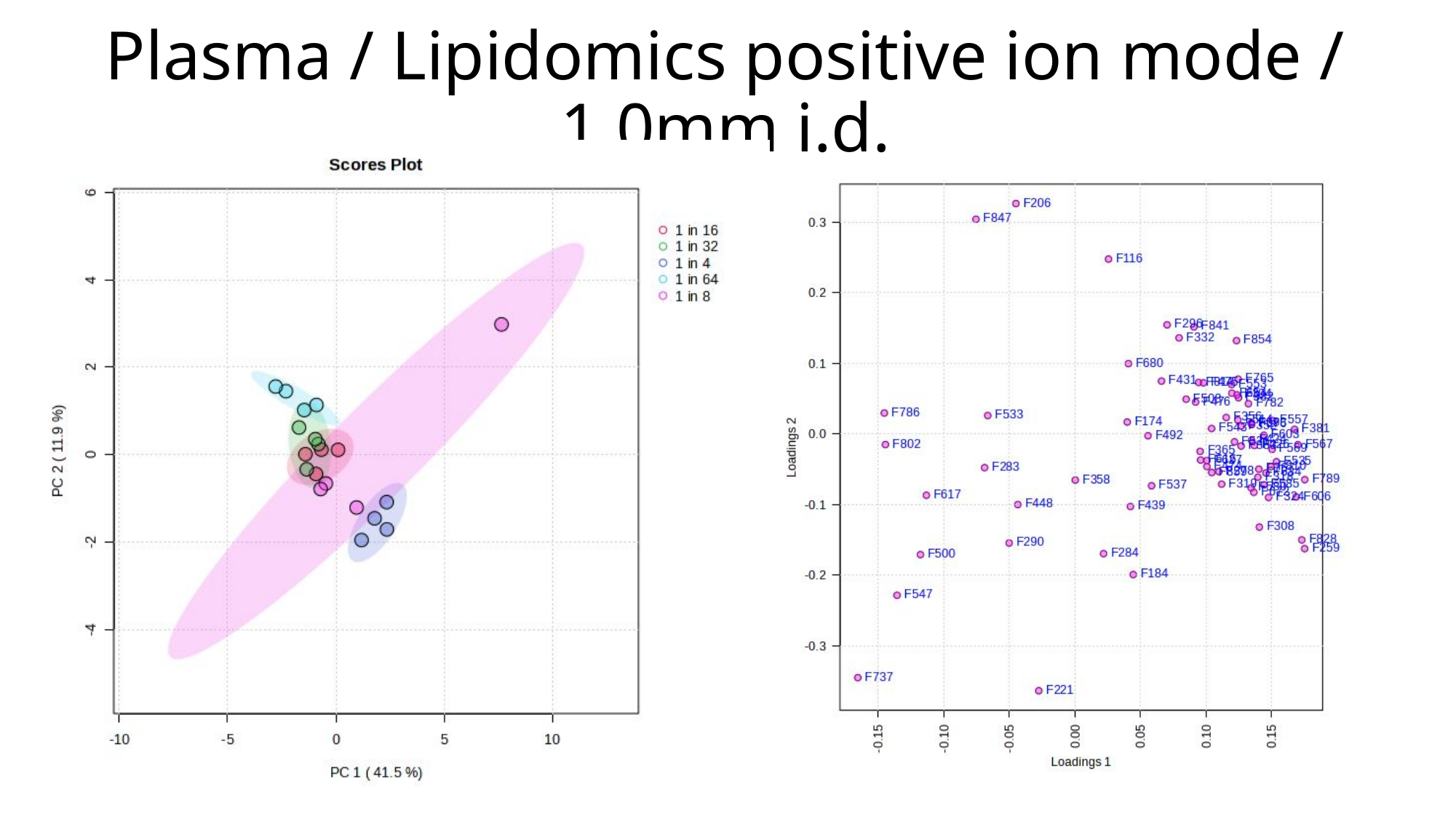

# Plasma / Lipidomics positive ion mode / 1.0mm i.d.

## Slide 42
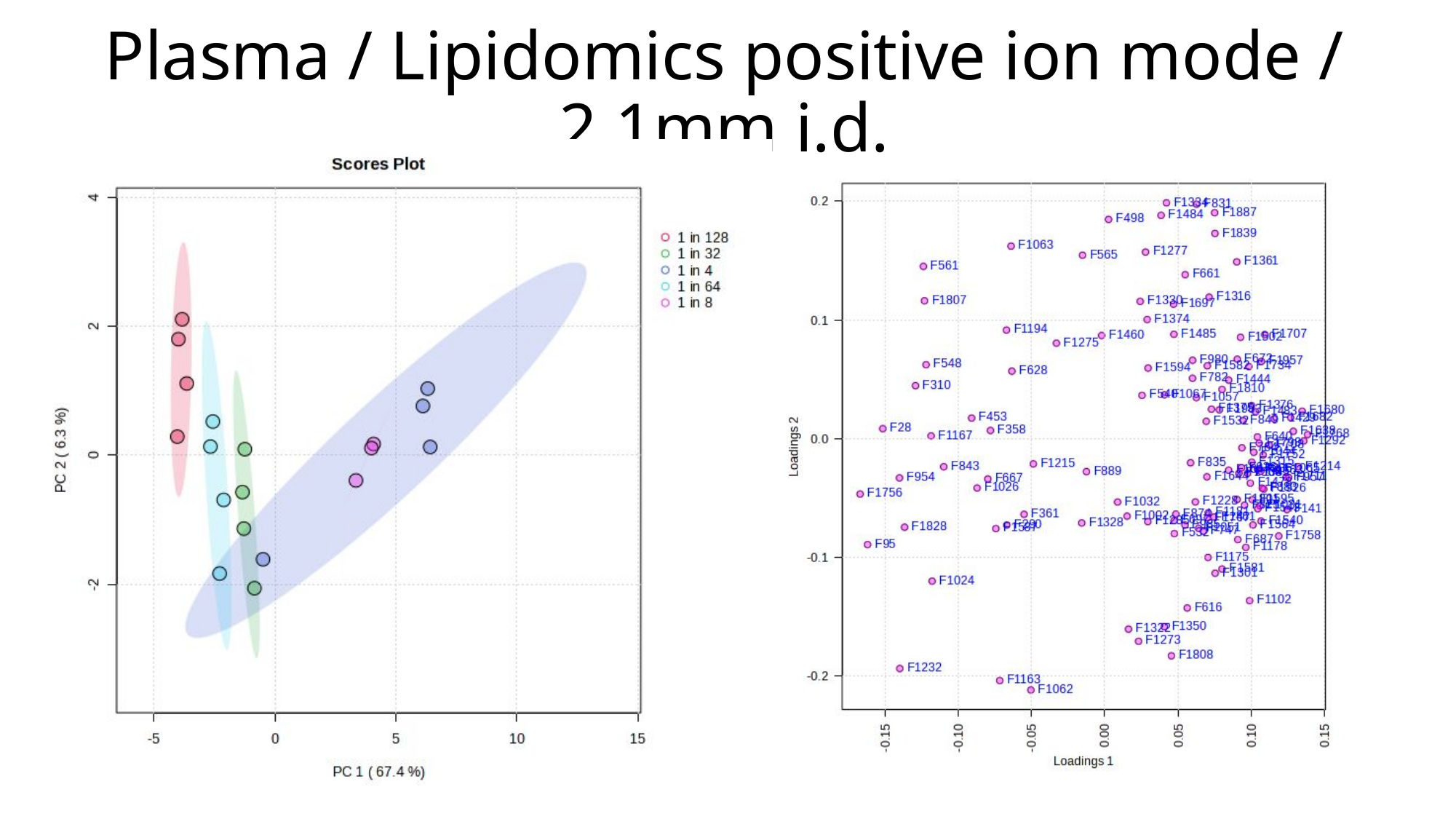

# Plasma / Lipidomics positive ion mode / 2.1mm i.d.

## Slide 43
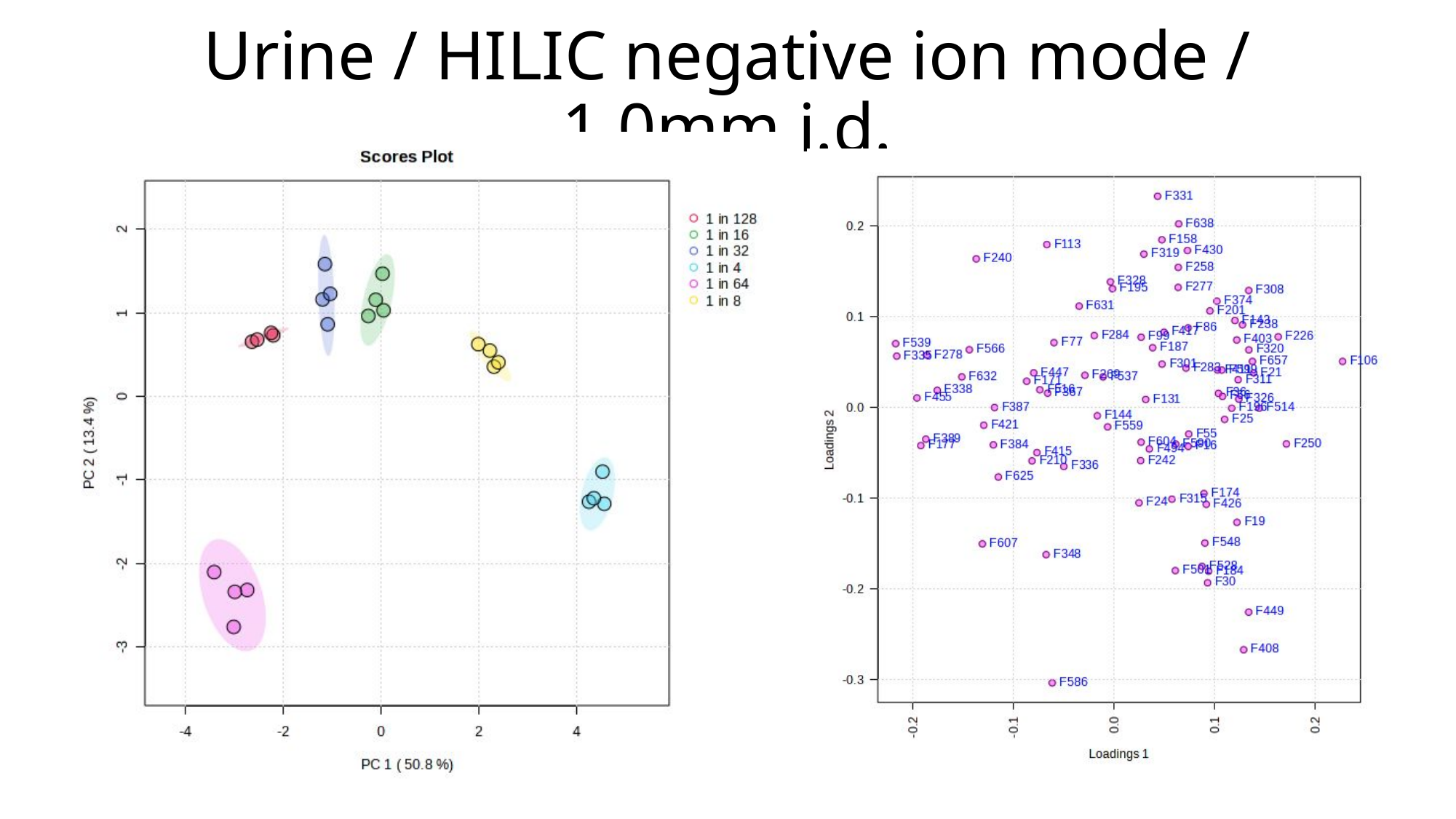

# Urine / HILIC negative ion mode / 1.0mm i.d.

## Slide 44
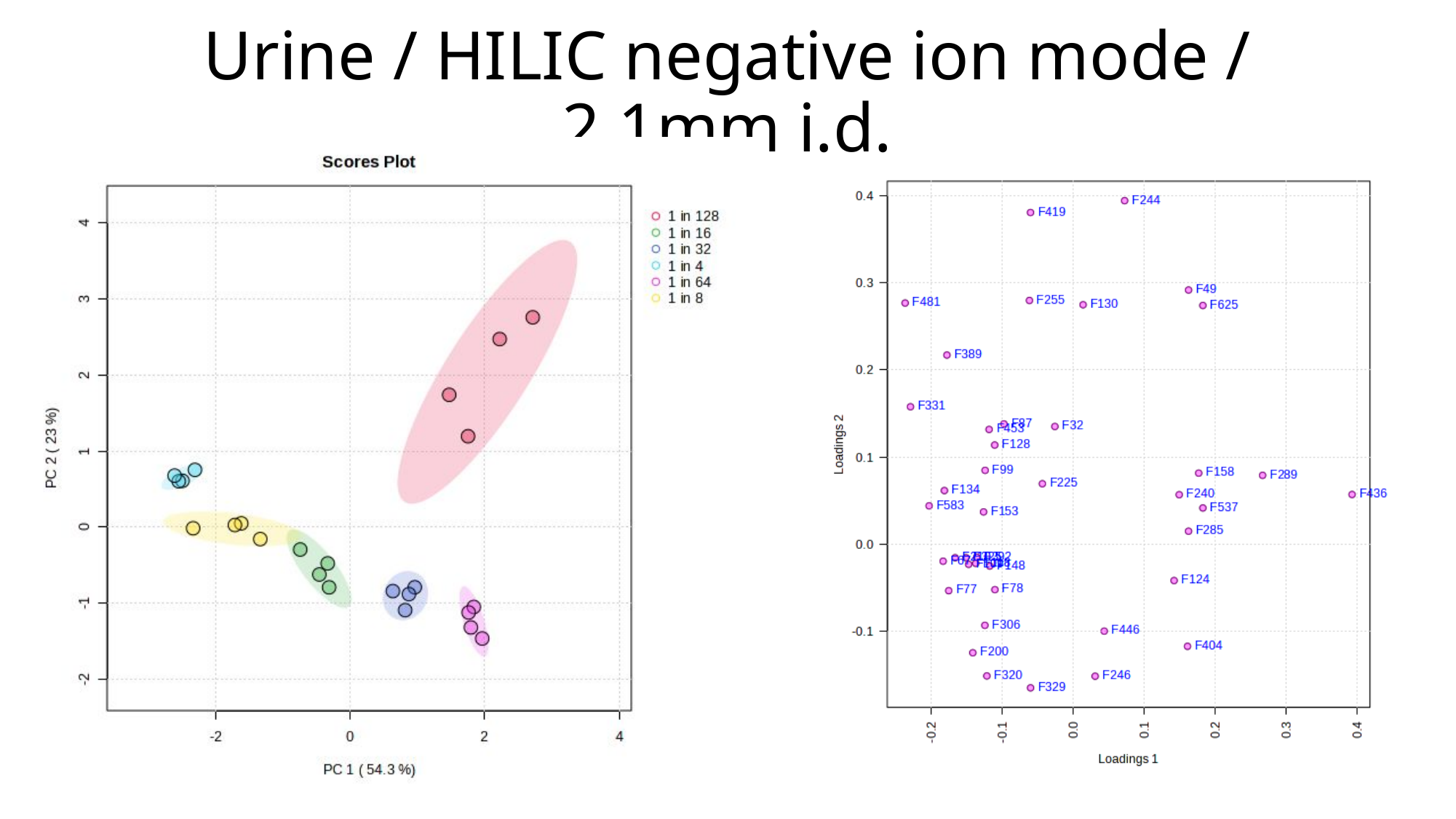

# Urine / HILIC negative ion mode / 2.1mm i.d.

## Slide 45
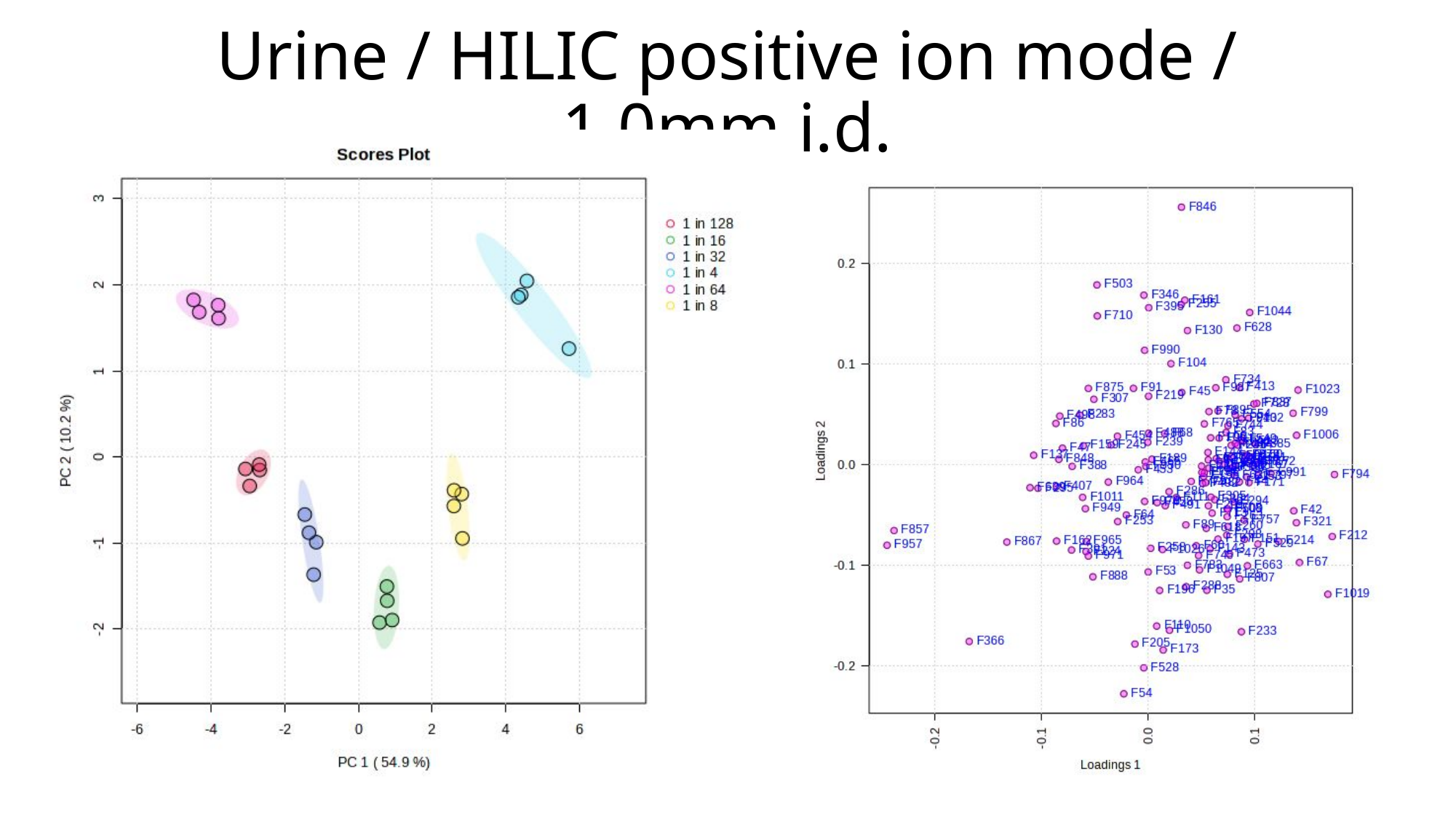

# Urine / HILIC positive ion mode / 1.0mm i.d.

## Slide 46
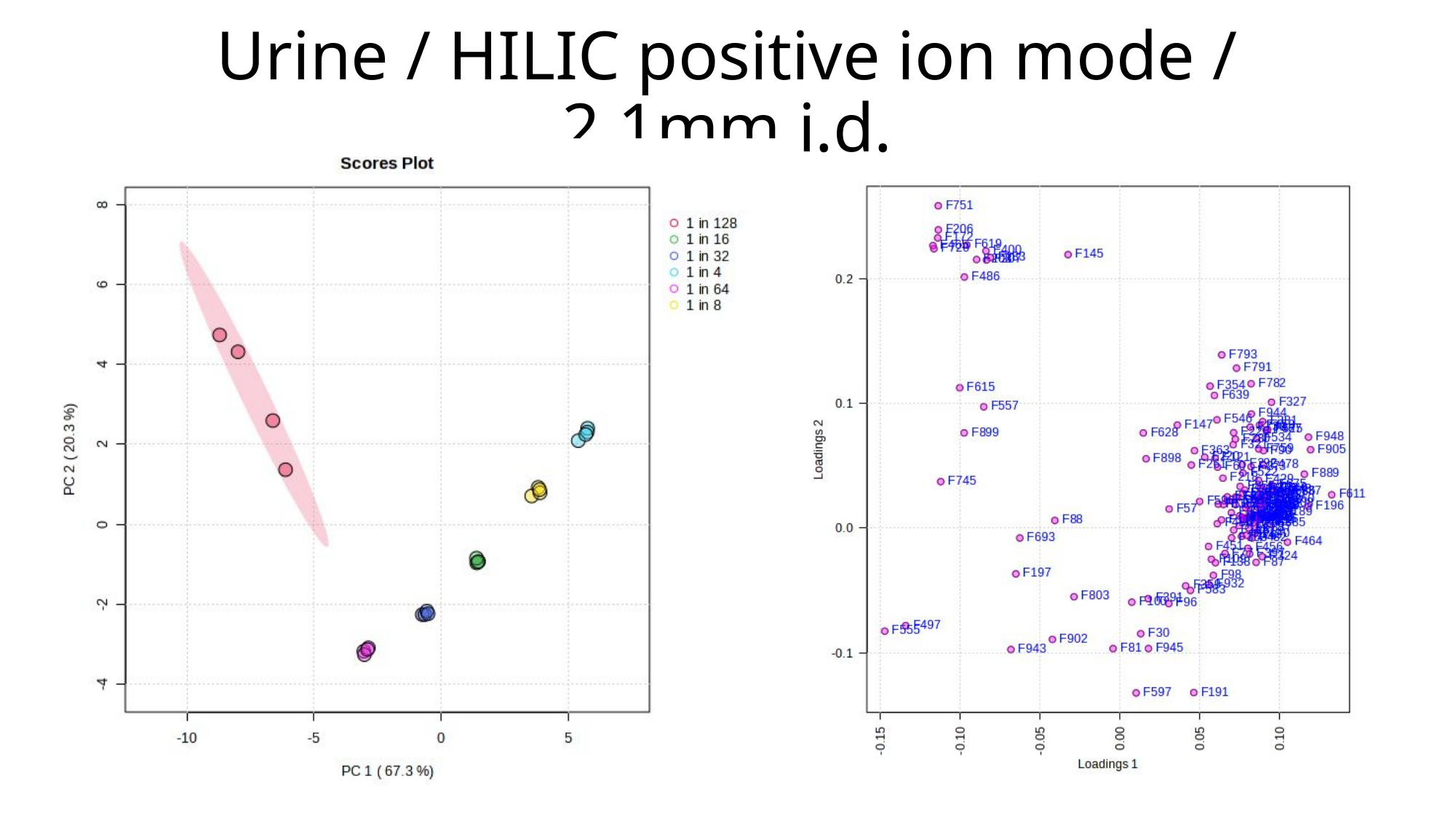

# Urine / HILIC positive ion mode / 2.1mm i.d.

## Slide 47
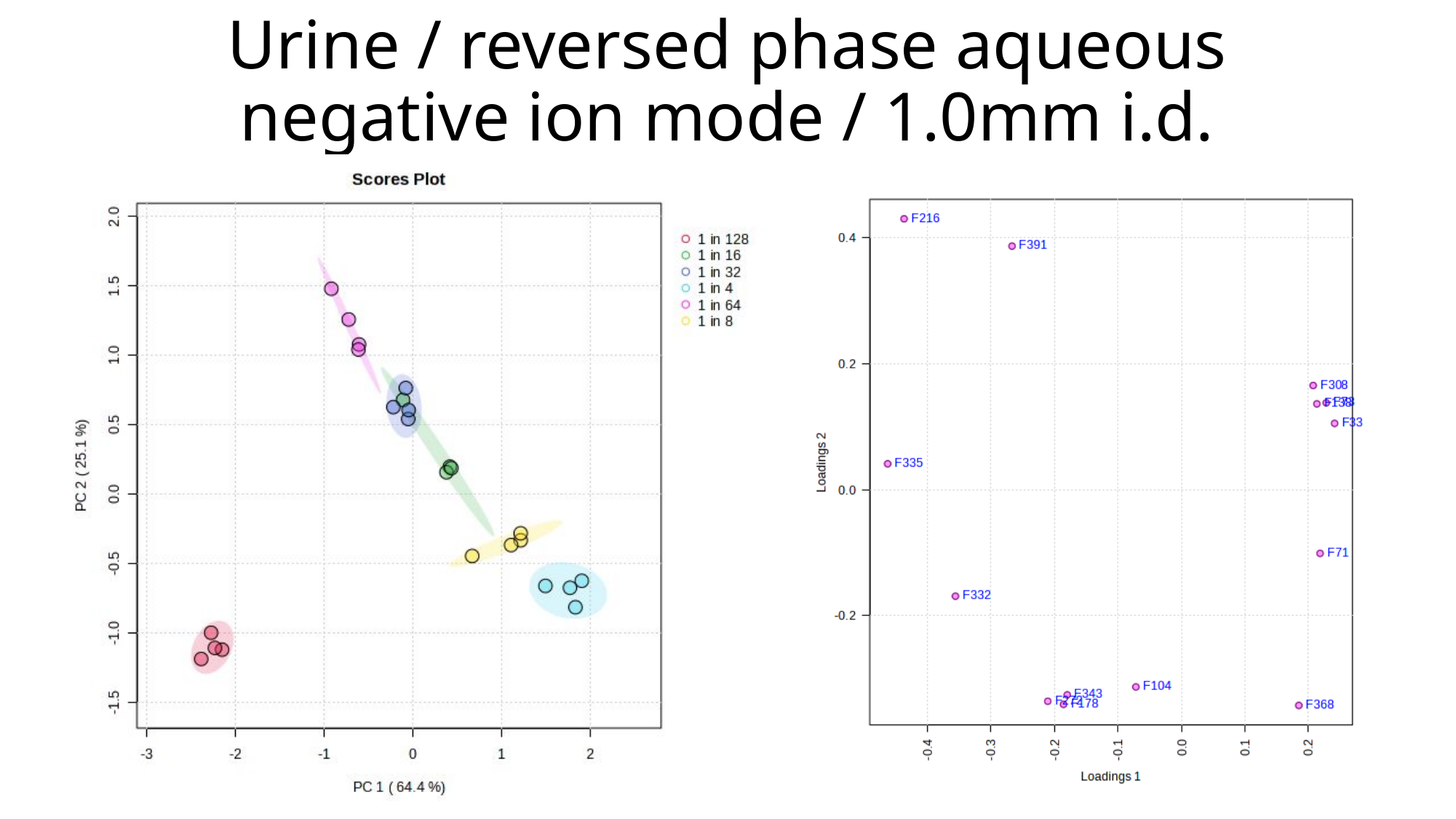

# Urine / reversed phase aqueous negative ion mode / 1.0mm i.d.

## Slide 48
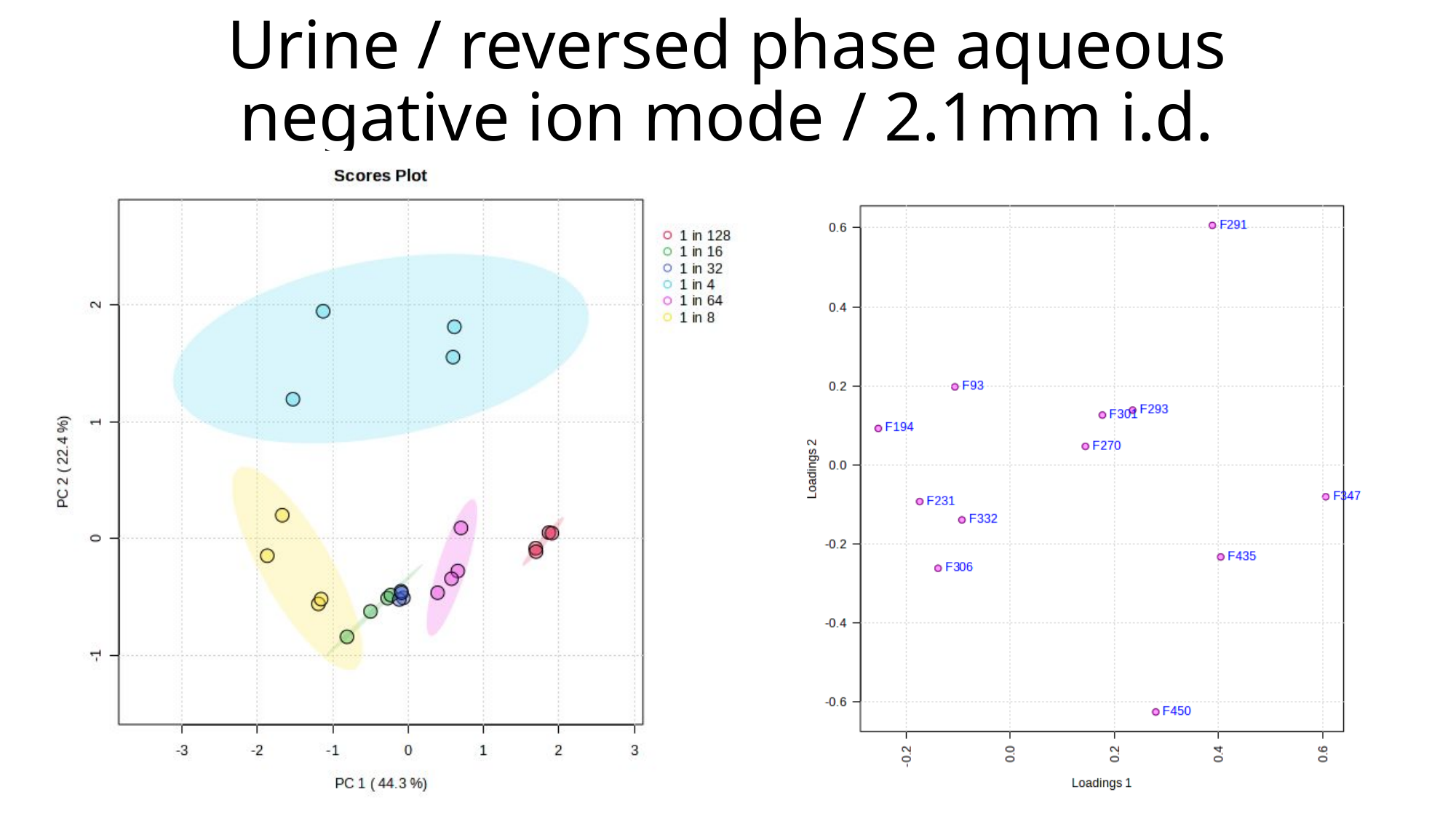

# Urine / reversed phase aqueous negative ion mode / 2.1mm i.d.

## Slide 49
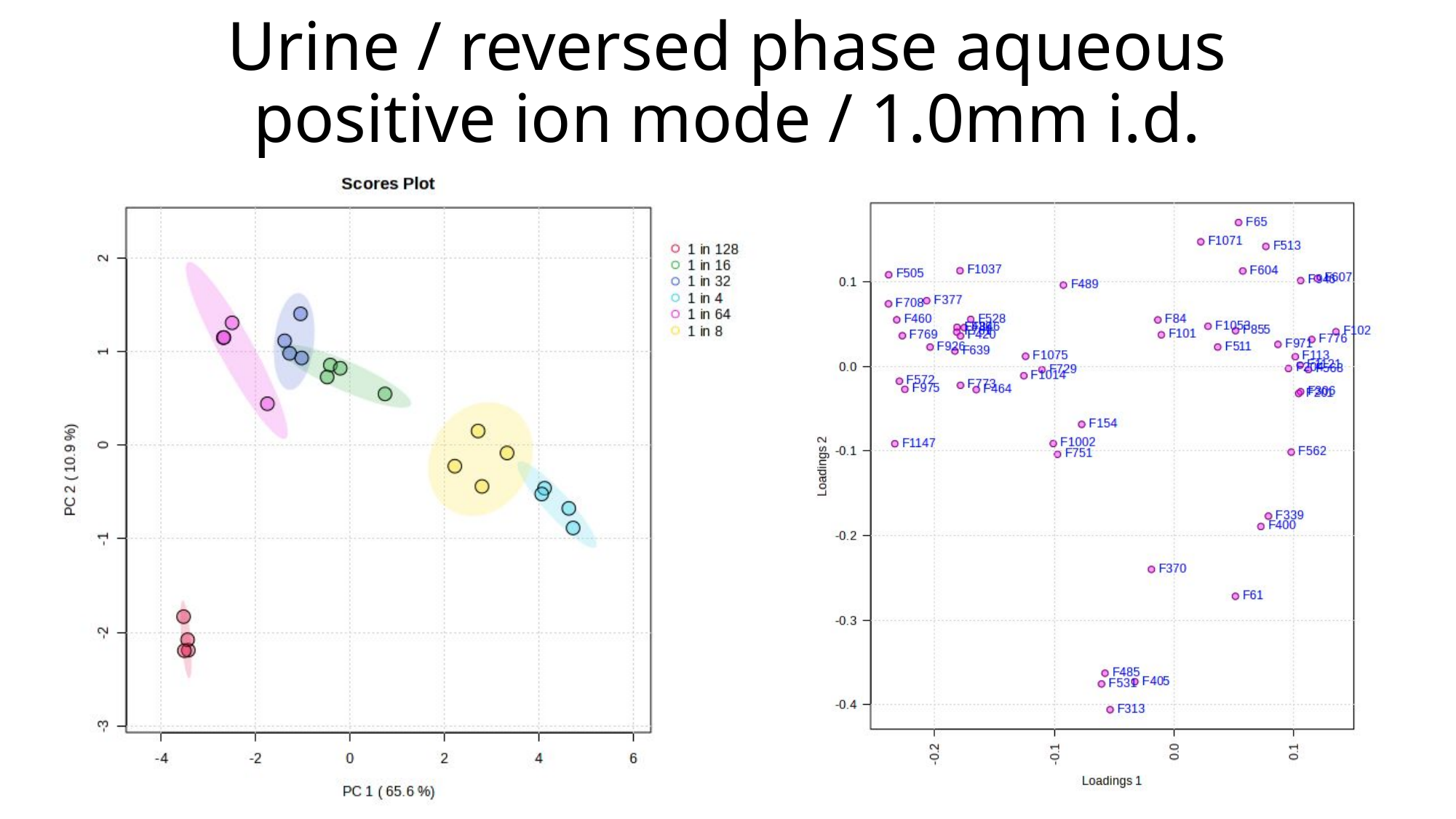

# Urine / reversed phase aqueous positive ion mode / 1.0mm i.d.

## Slide 50
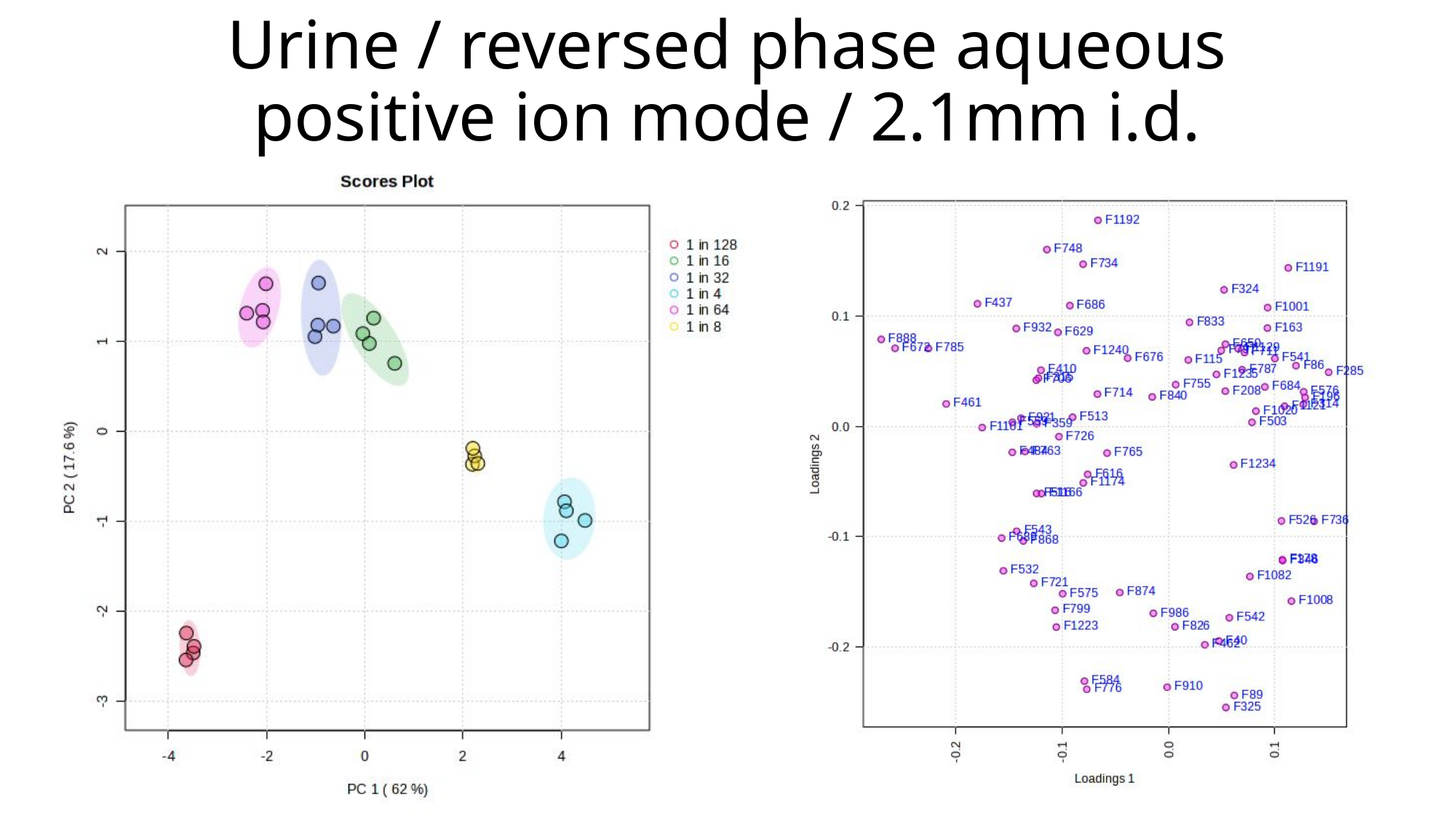

# Urine / reversed phase aqueous positive ion mode / 2.1mm i.d.

## Slide 51
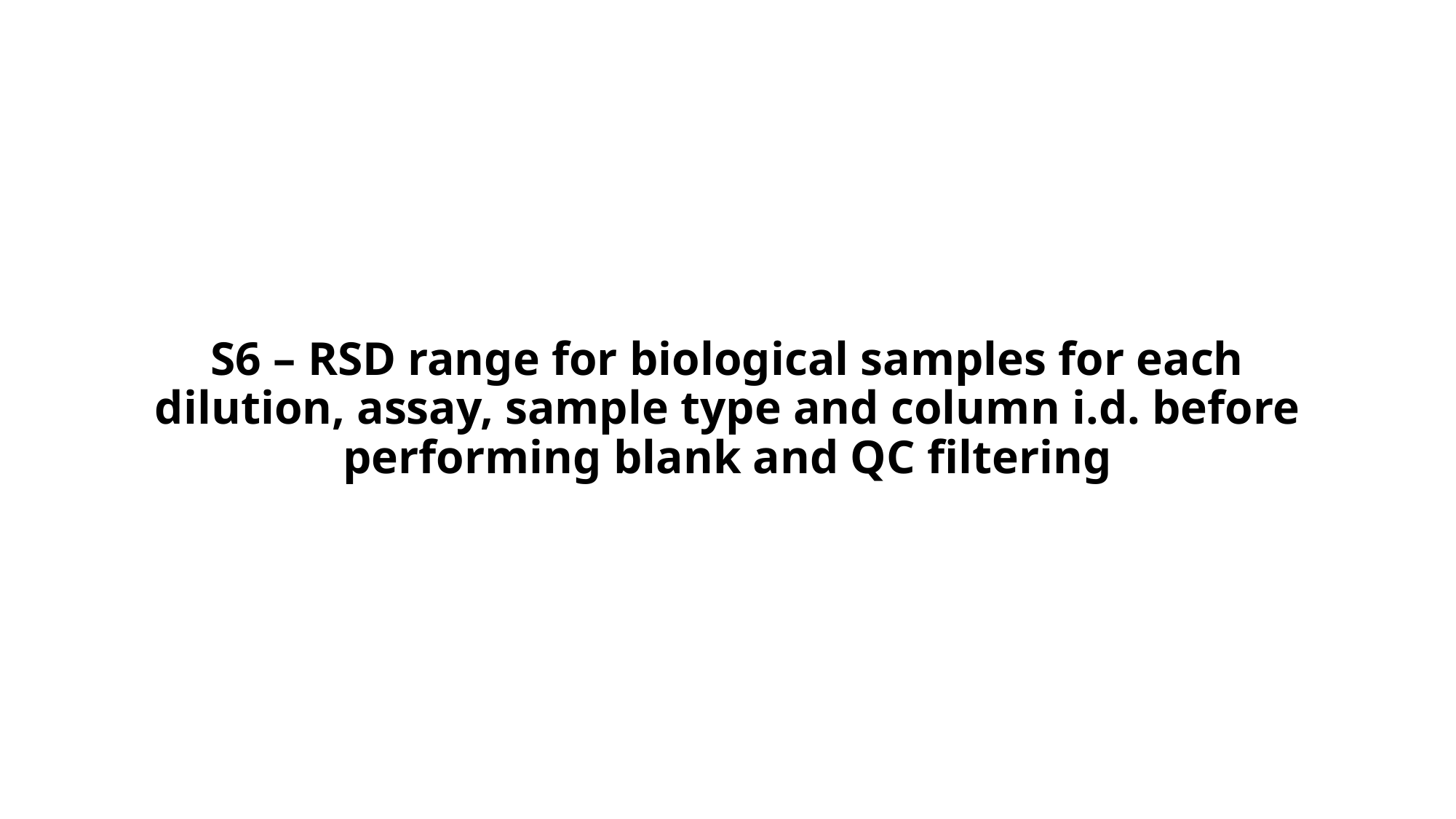

# S6 – RSD range for biological samples for each dilution, assay, sample type and column i.d. before performing blank and QC filtering

## Slide 52
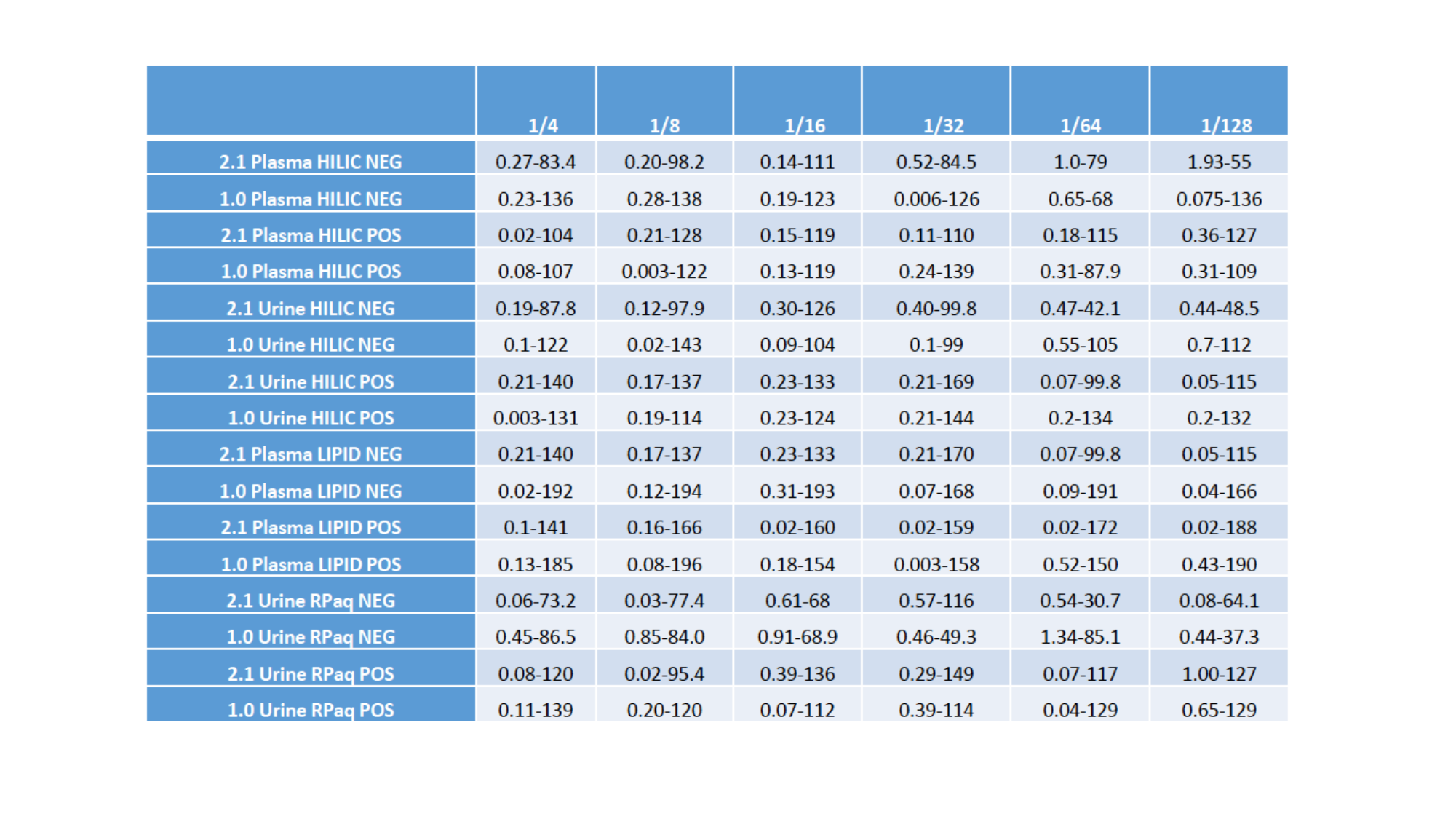

## Slide 53
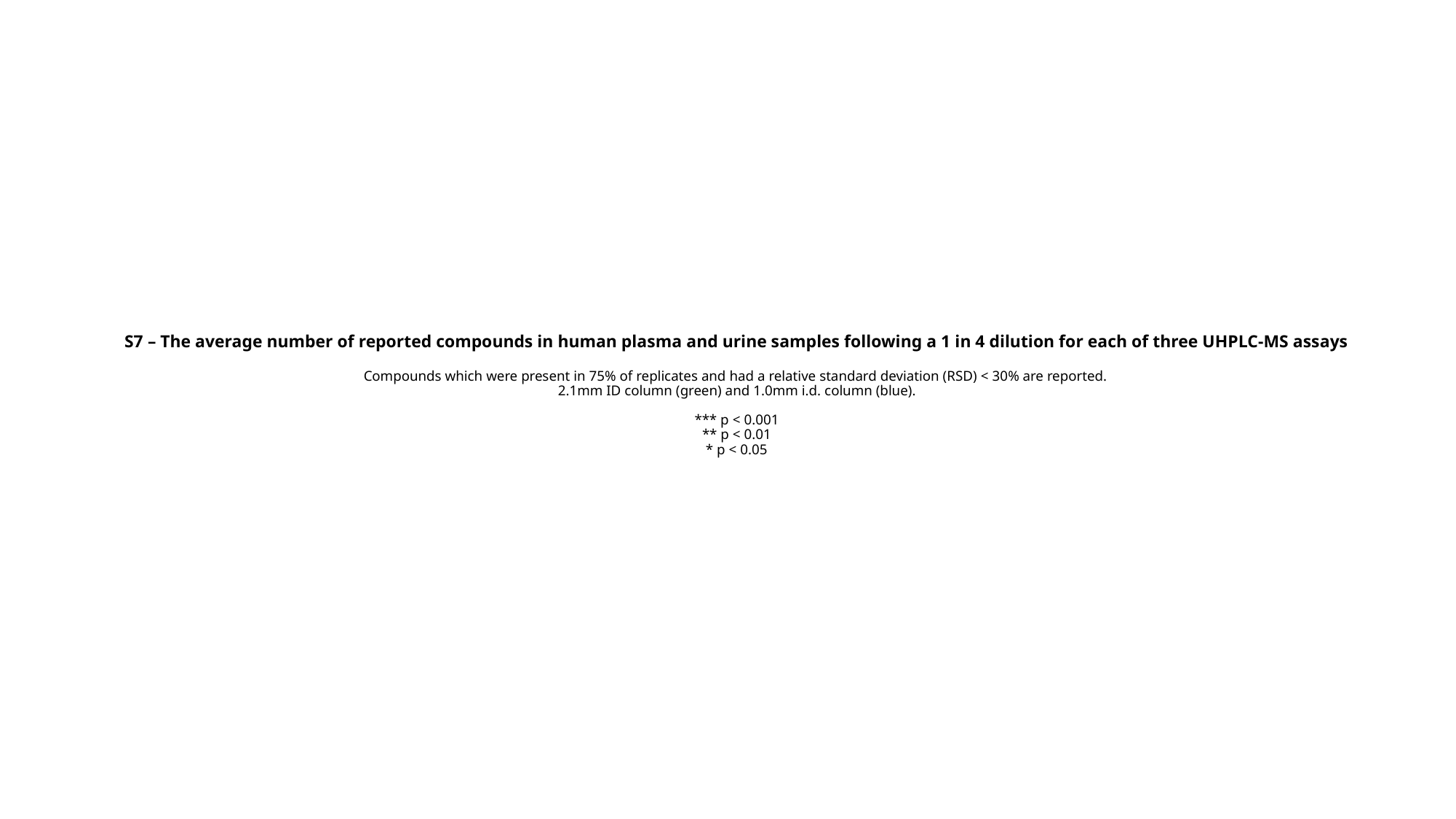

# S7 – The average number of reported compounds in human plasma and urine samples following a 1 in 4 dilution for each of three UHPLC-MS assays Compounds which were present in 75% of replicates and had a relative standard deviation (RSD) < 30% are reported. 2.1mm ID column (green) and 1.0mm i.d. column (blue). *** p < 0.001 ** p < 0.01 * p < 0.05

## Slide 54
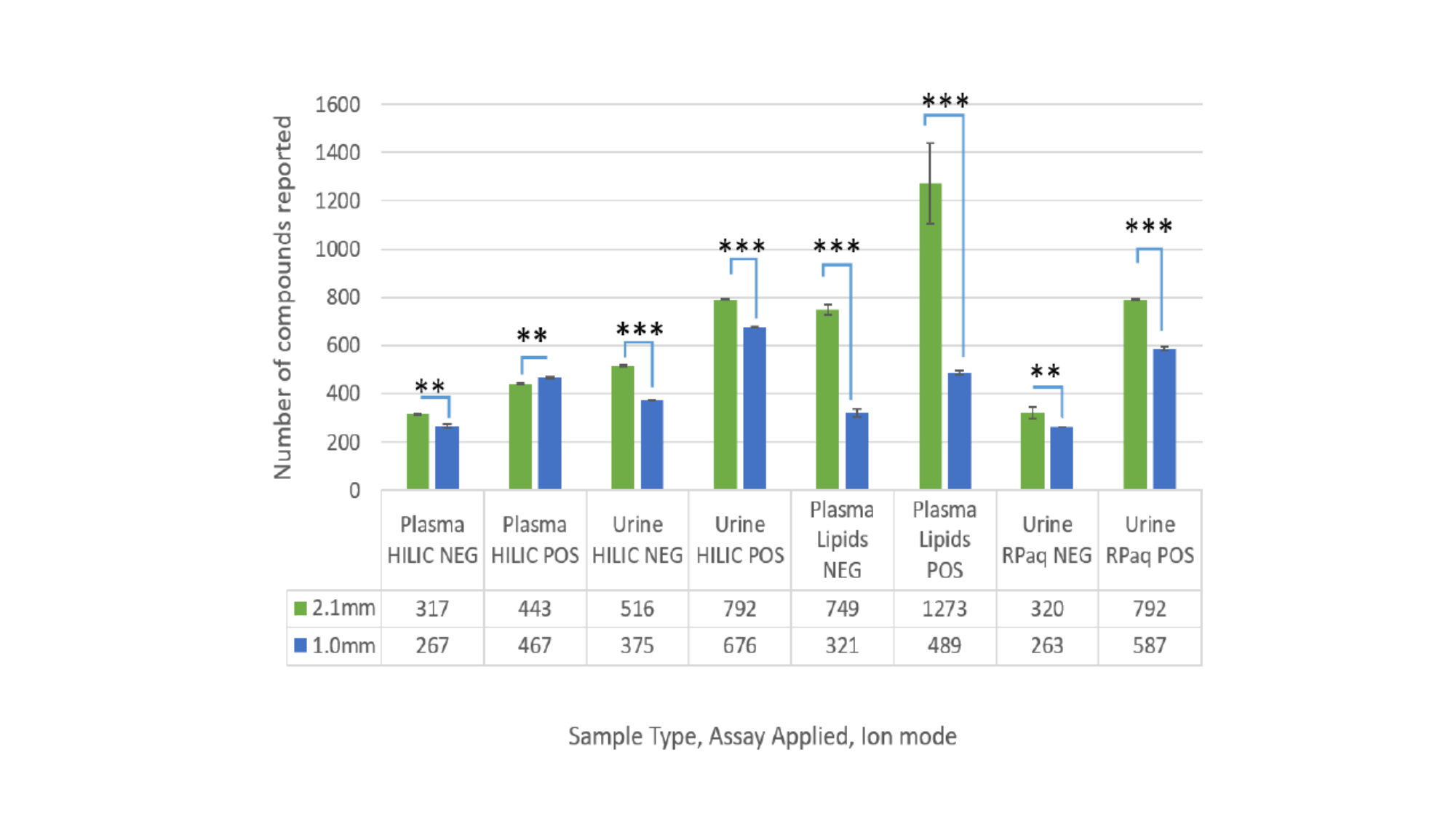

## Slide 55
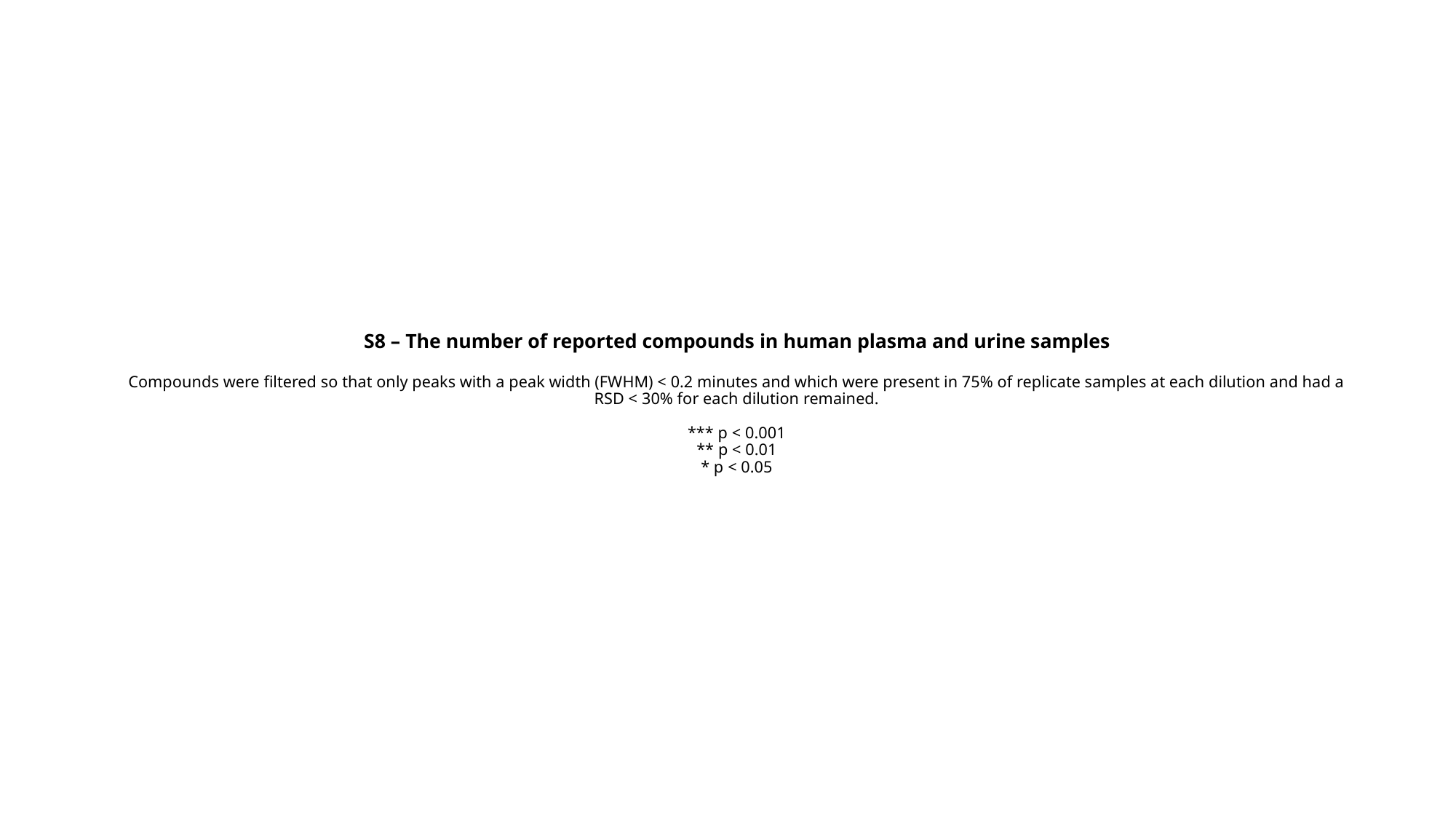

# S8 – The number of reported compounds in human plasma and urine samples Compounds were filtered so that only peaks with a peak width (FWHM) < 0.2 minutes and which were present in 75% of replicate samples at each dilution and had a RSD < 30% for each dilution remained. *** p < 0.001 ** p < 0.01 * p < 0.05

## Slide 56
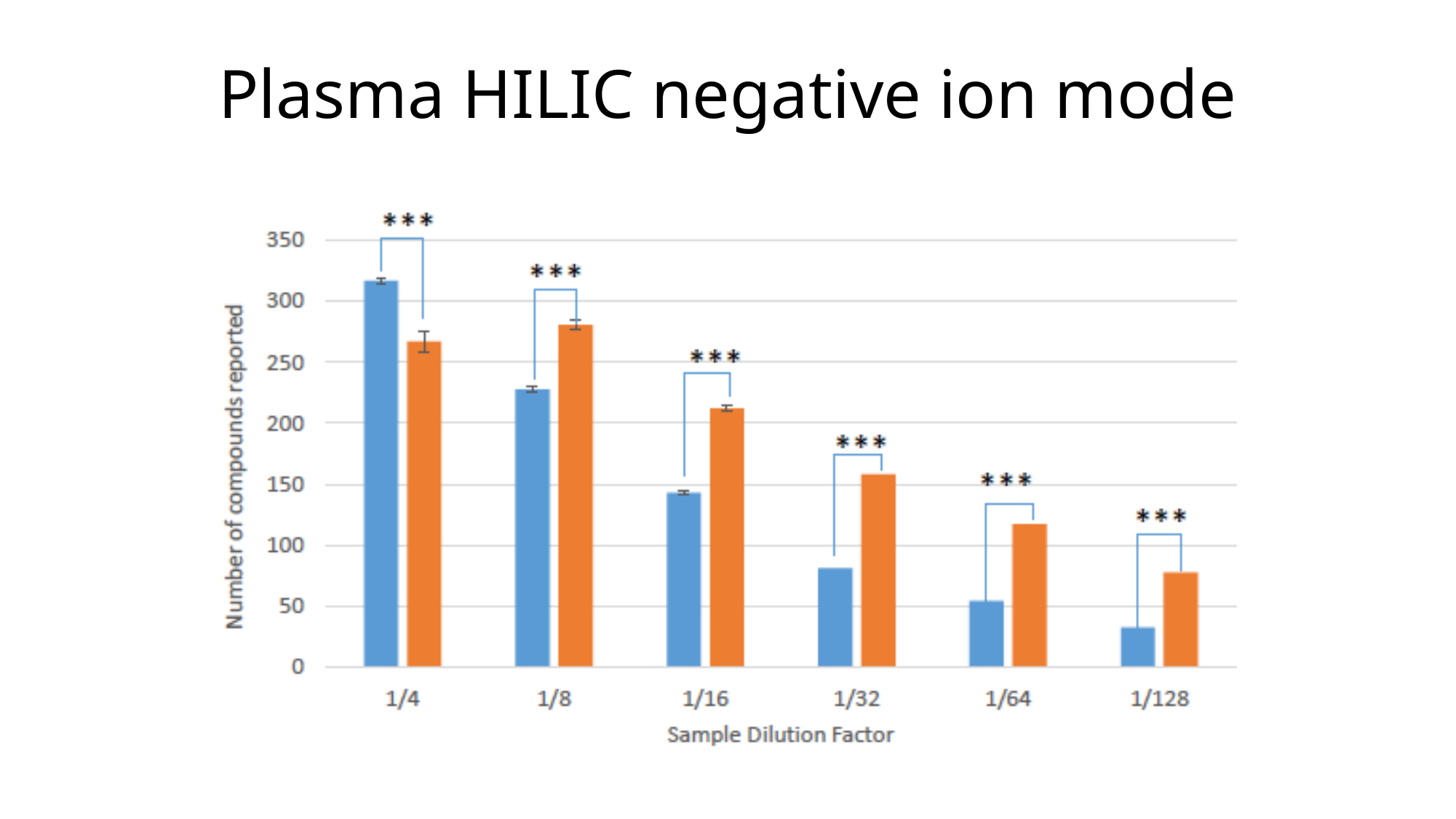

# Plasma HILIC negative ion mode

## Slide 57
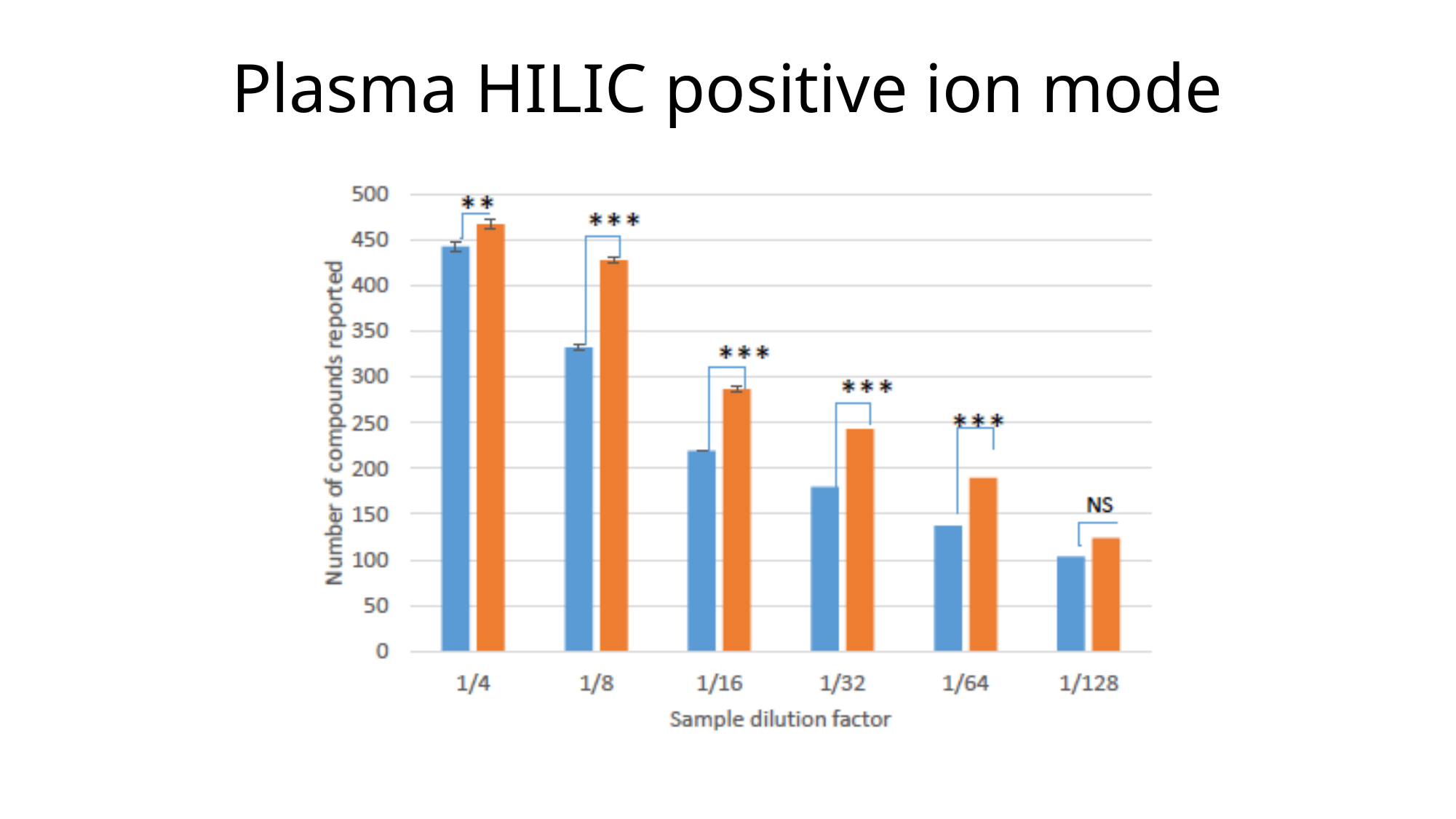

# Plasma HILIC positive ion mode

## Slide 58
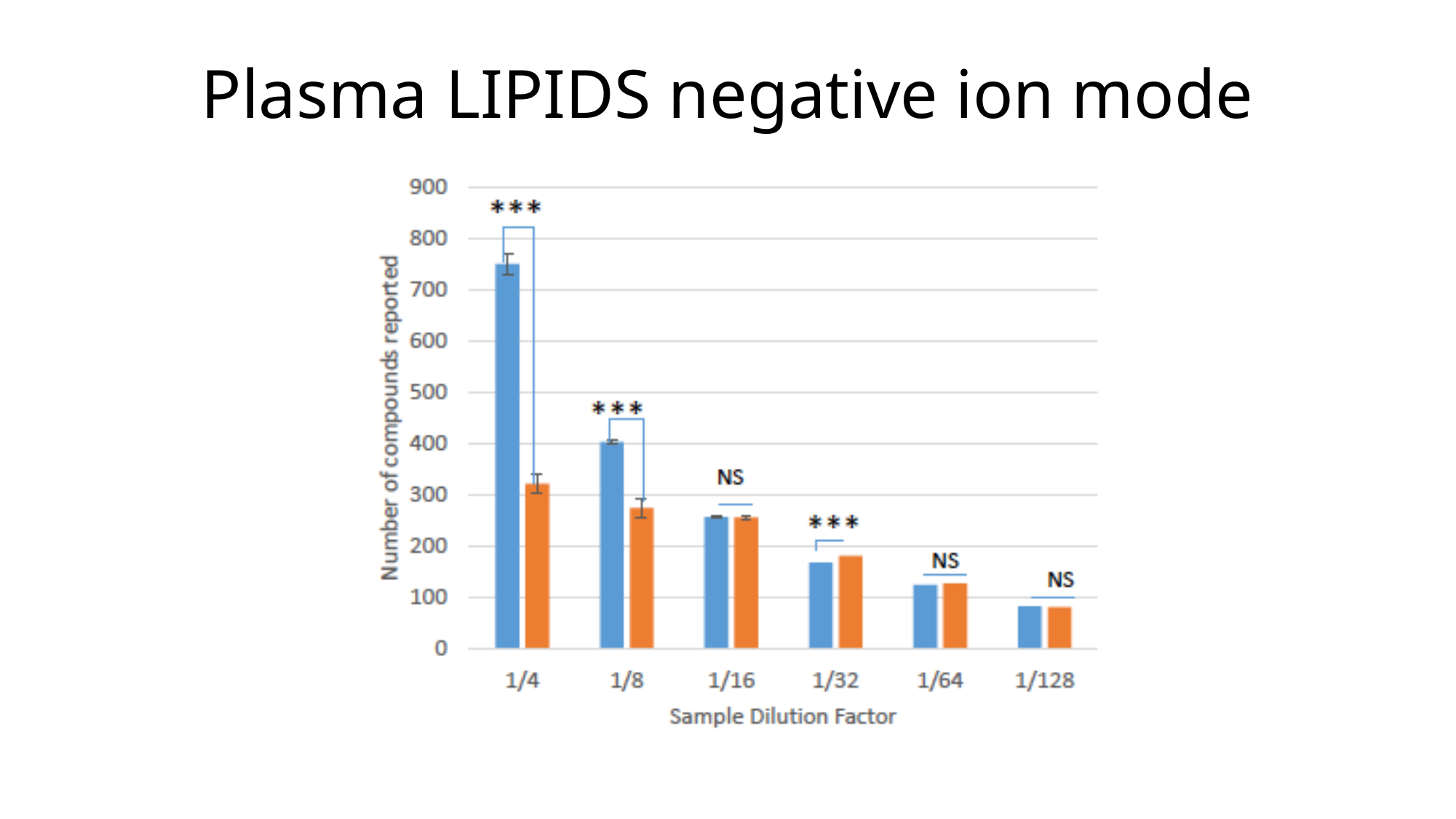

# Plasma LIPIDS negative ion mode

## Slide 59
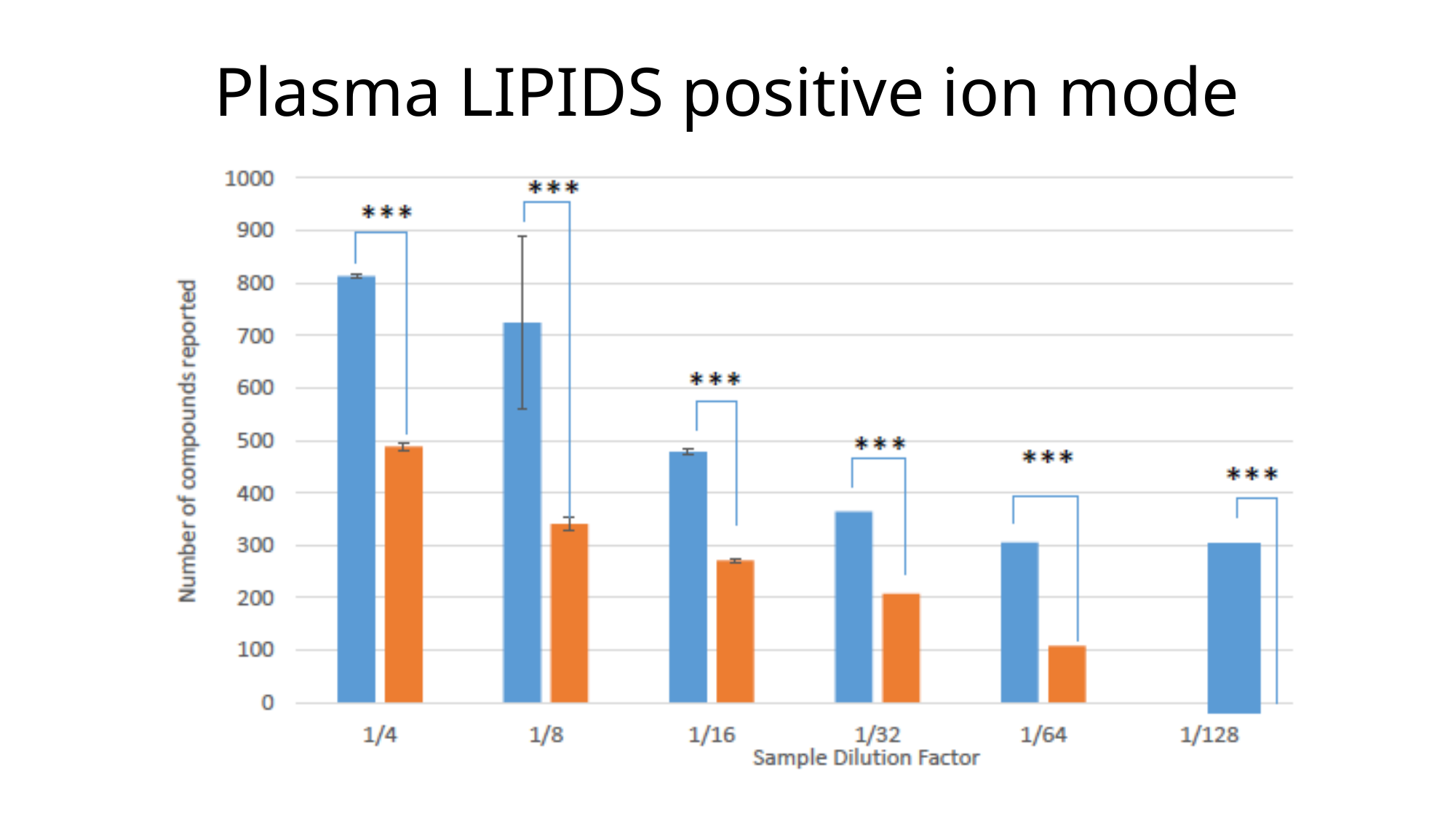

# Plasma LIPIDS positive ion mode

## Slide 60
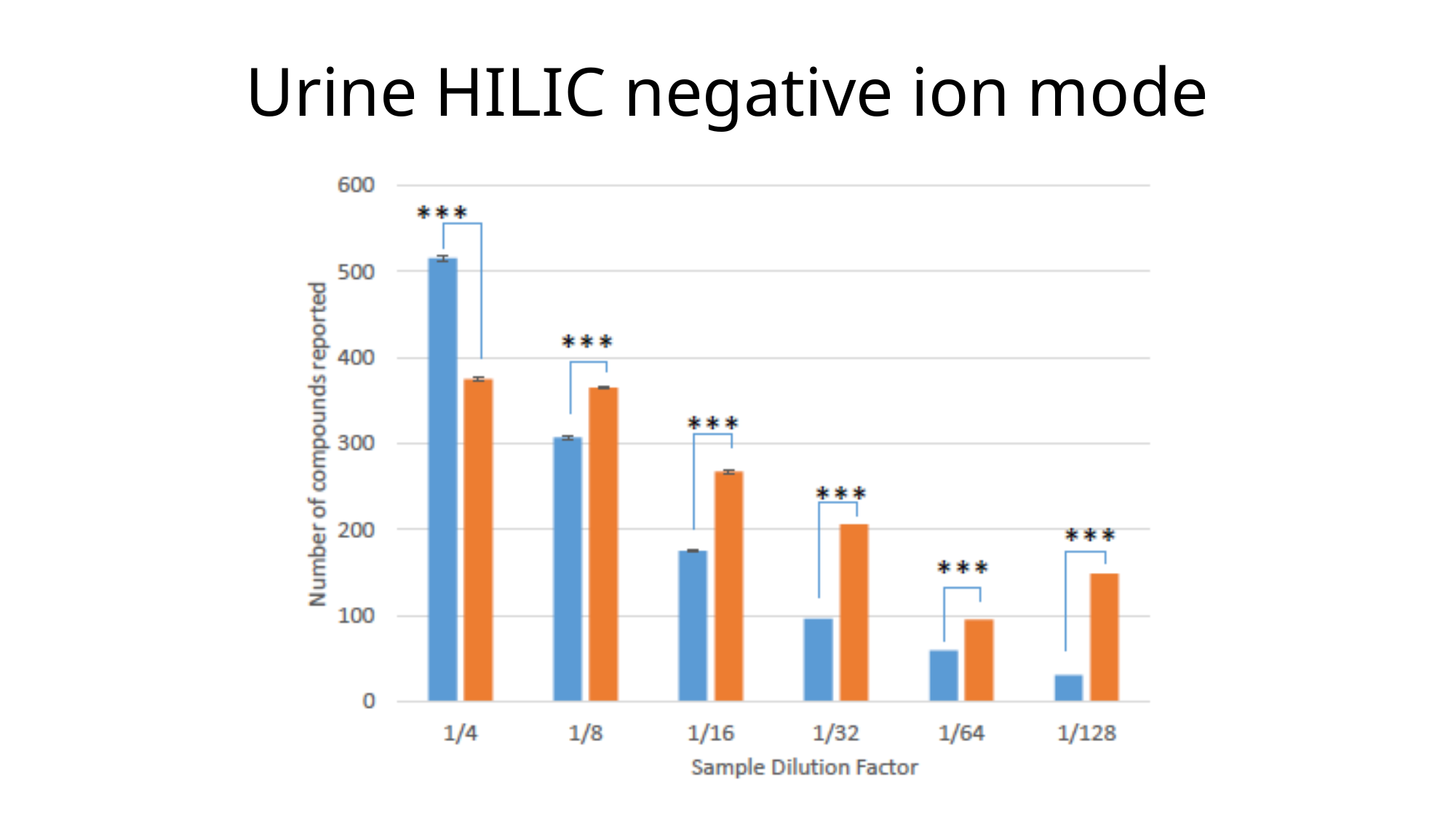

# Urine HILIC negative ion mode

## Slide 61
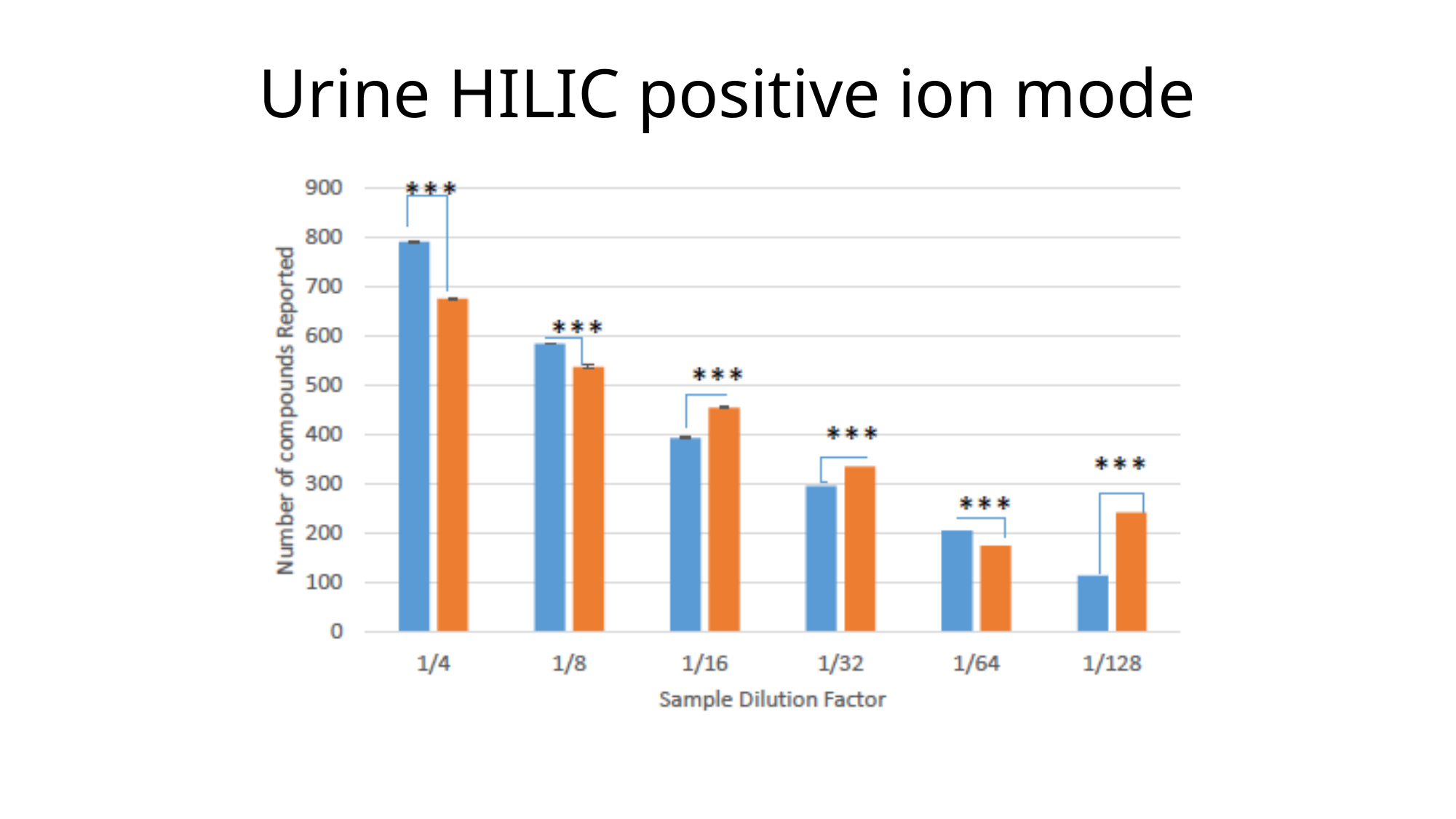

# Urine HILIC positive ion mode

## Slide 62
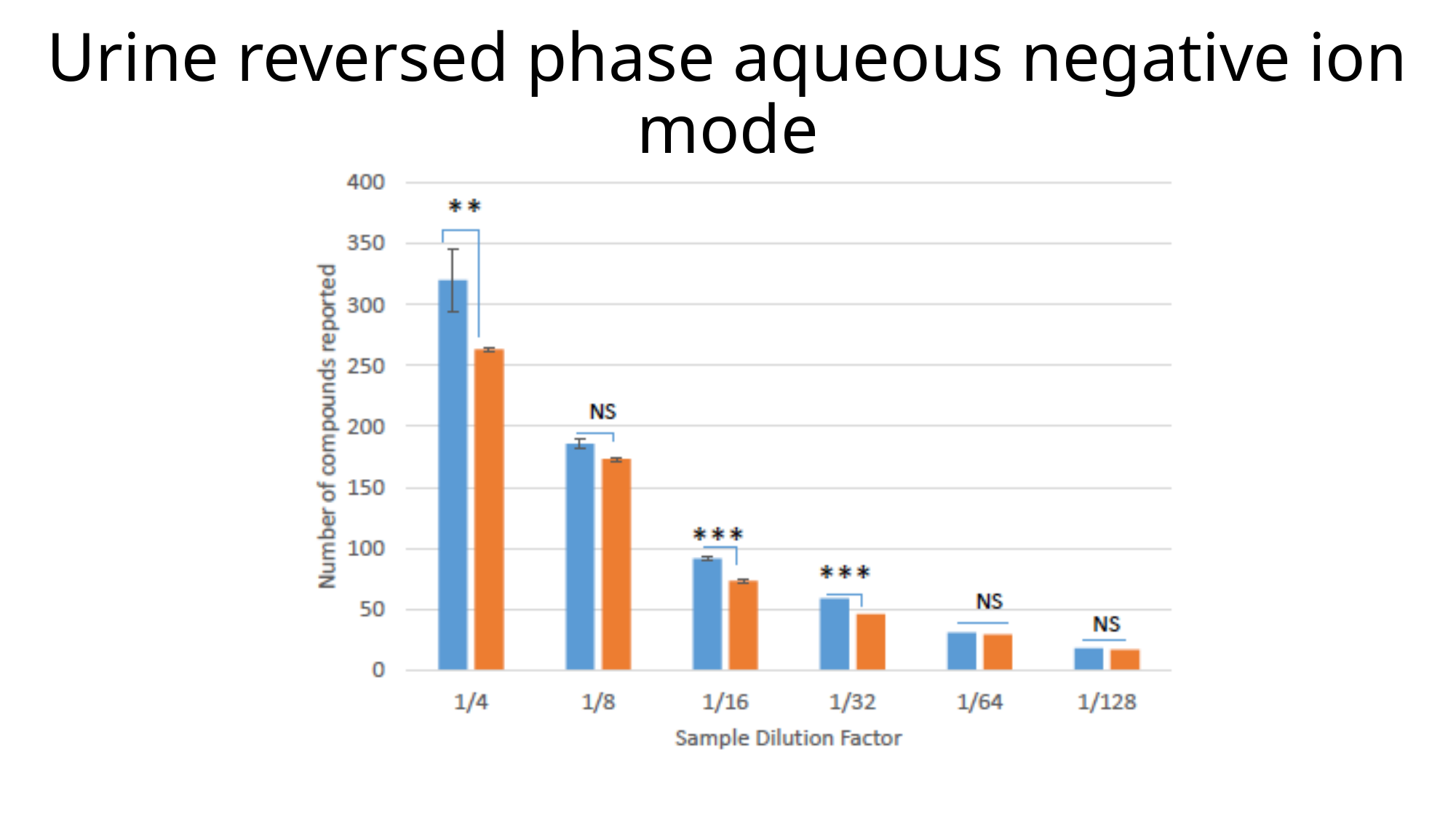

# Urine reversed phase aqueous negative ion mode

## Slide 63
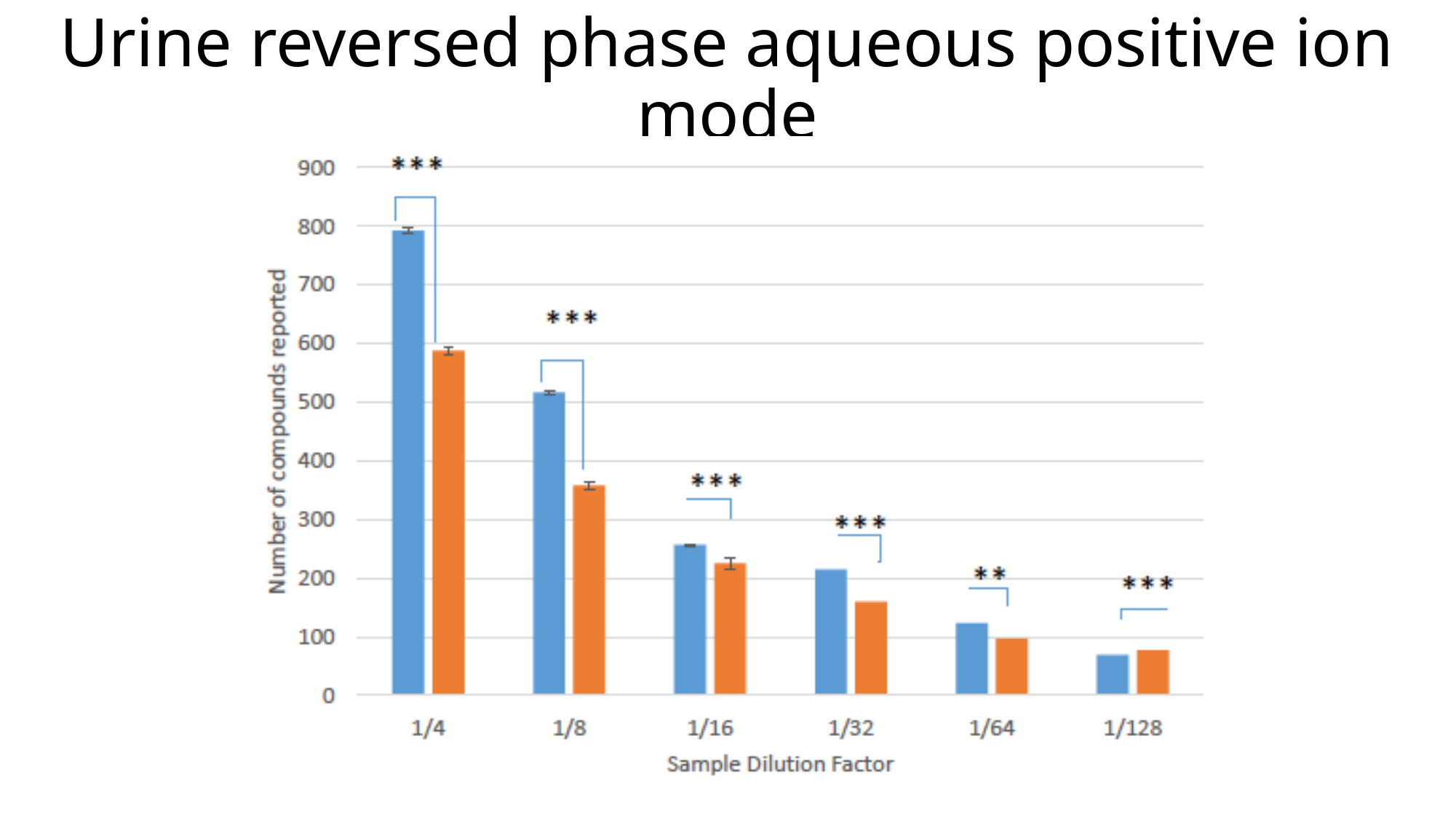

# Urine reversed phase aqueous positive ion mode

## Slide 64
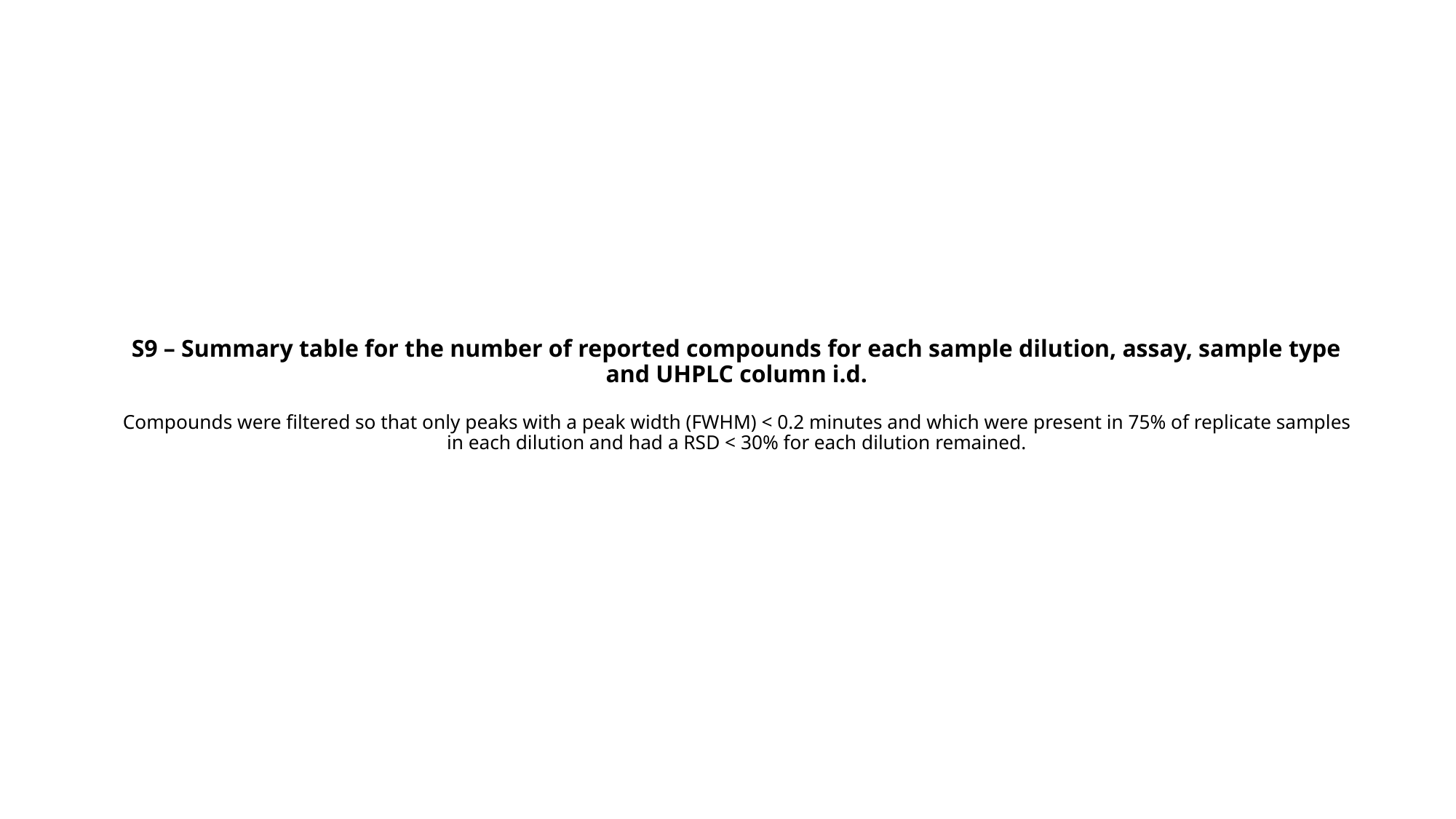

# S9 – Summary table for the number of reported compounds for each sample dilution, assay, sample type and UHPLC column i.d. Compounds were filtered so that only peaks with a peak width (FWHM) < 0.2 minutes and which were present in 75% of replicate samples in each dilution and had a RSD < 30% for each dilution remained.

## Slide 65
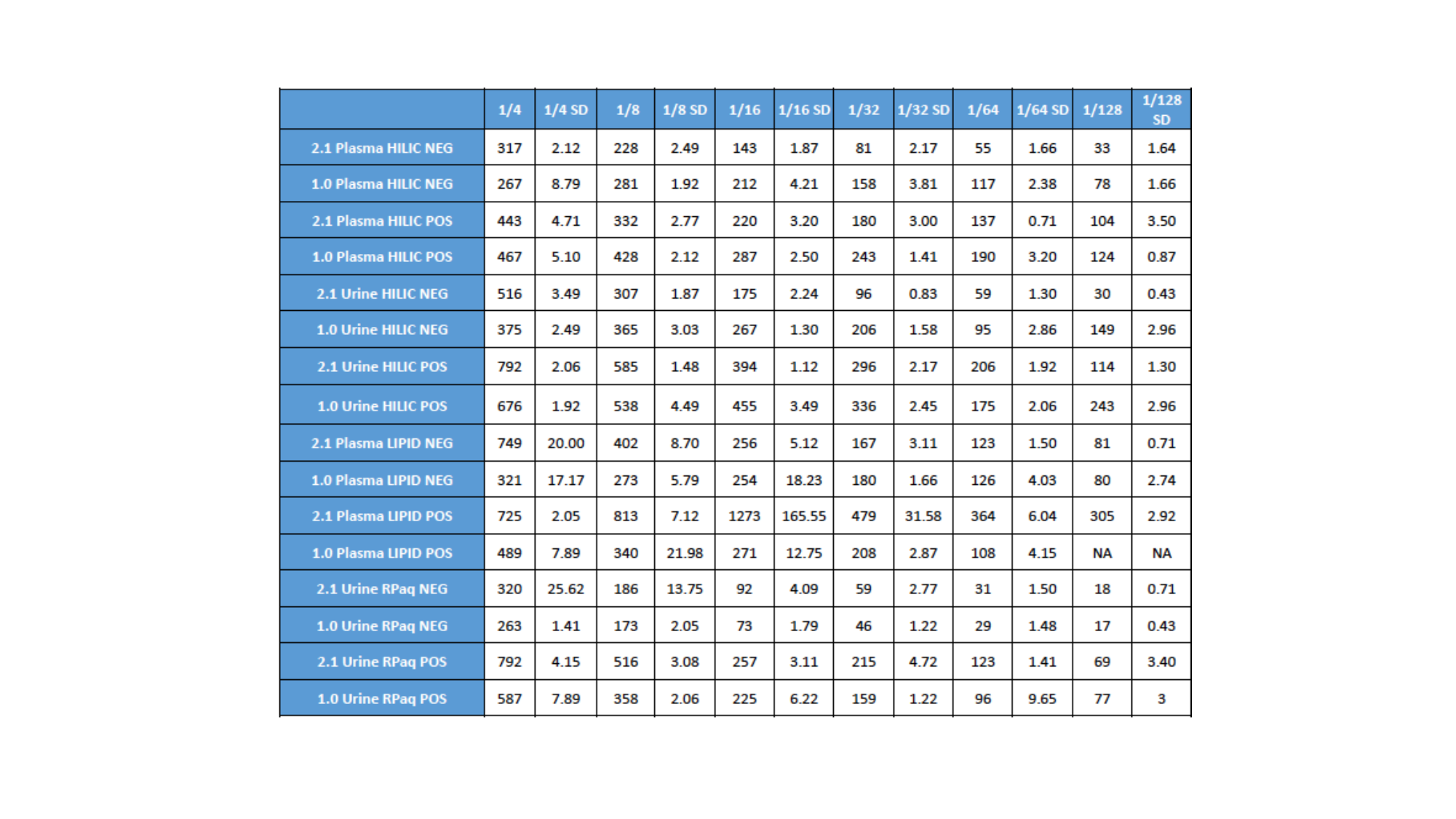

## Slide 66
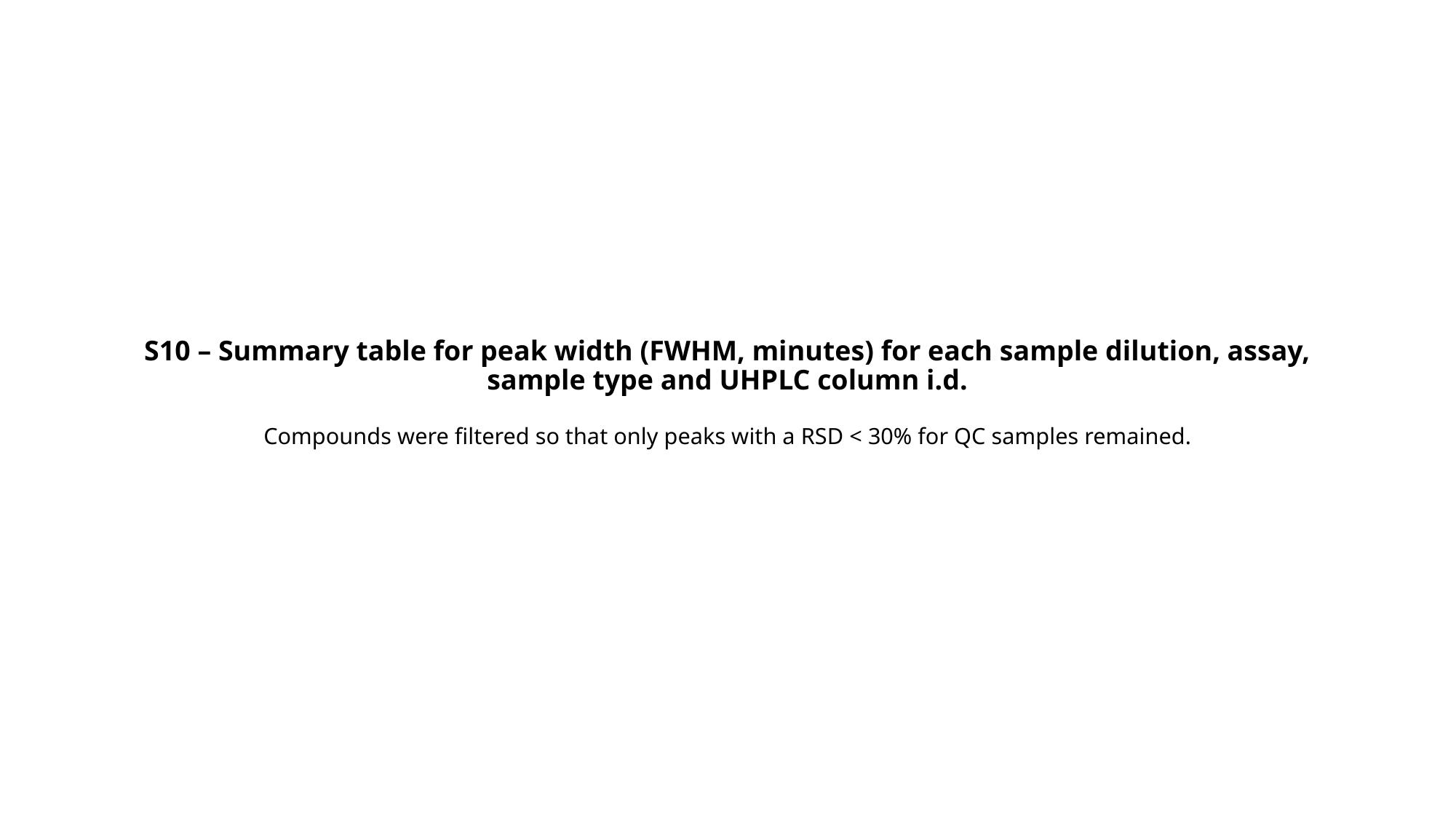

# S10 – Summary table for peak width (FWHM, minutes) for each sample dilution, assay, sample type and UHPLC column i.d. Compounds were filtered so that only peaks with a RSD < 30% for QC samples remained.

## Slide 67
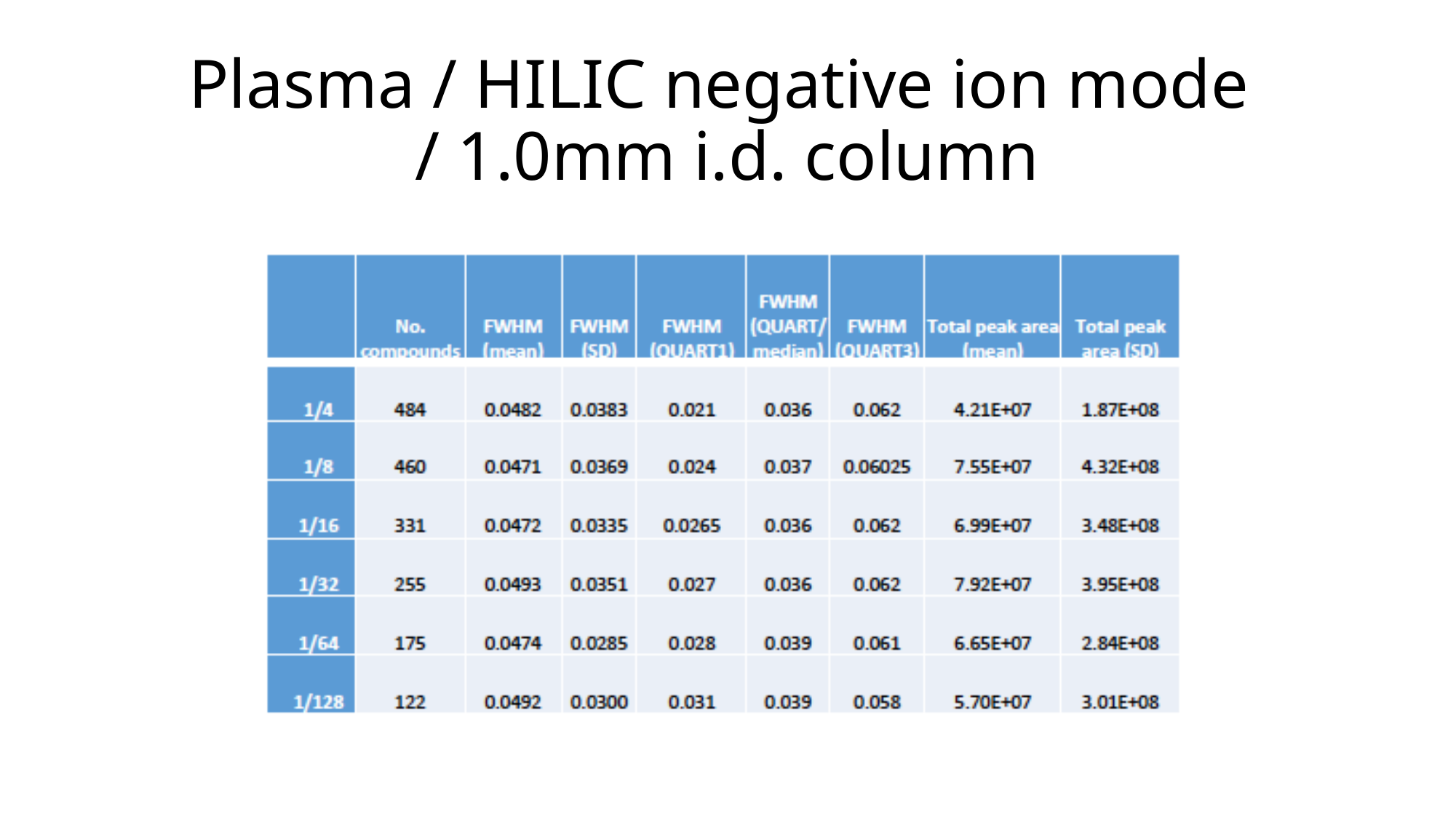

# Plasma / HILIC negative ion mode / 1.0mm i.d. column

## Slide 68
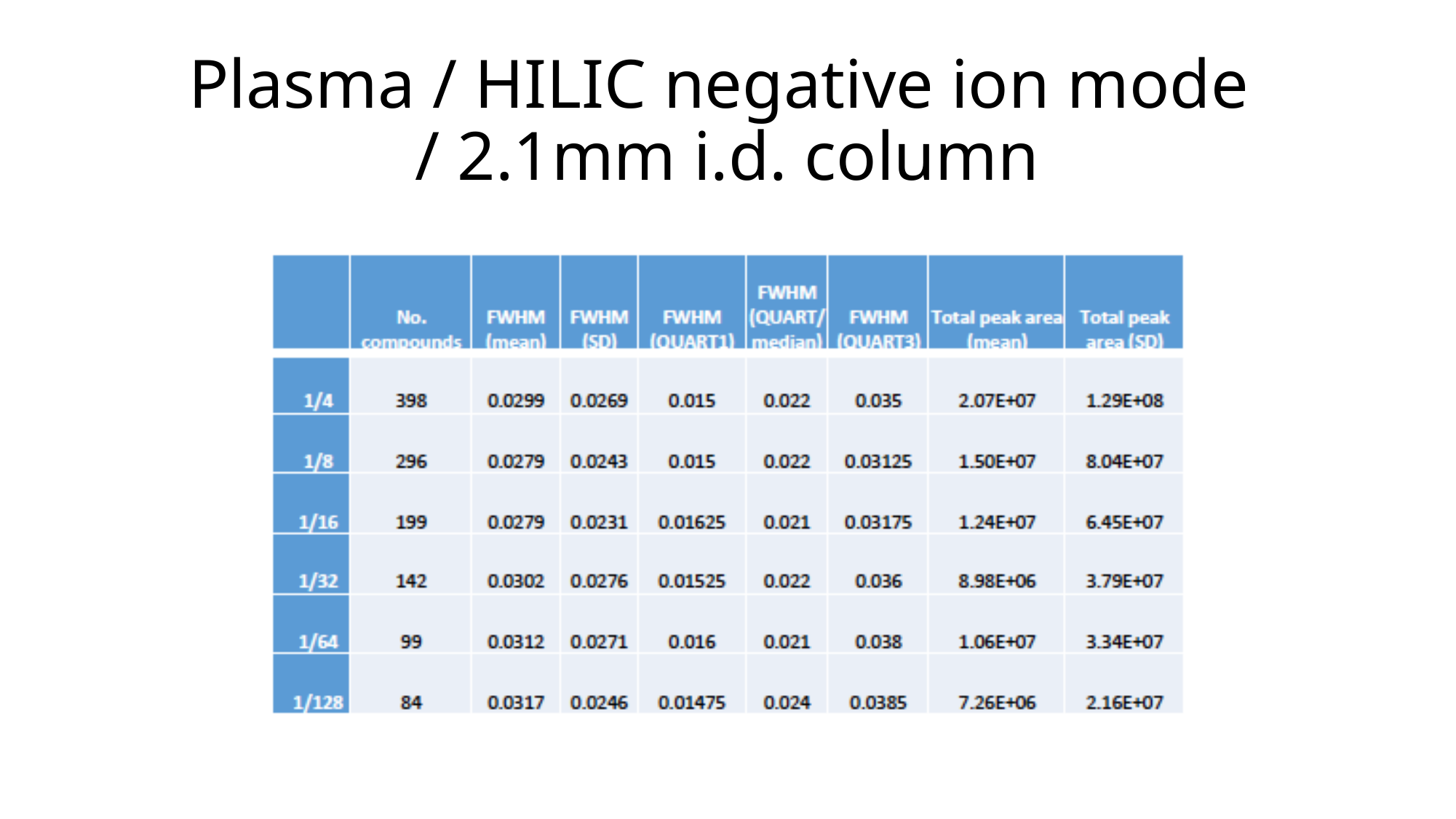

# Plasma / HILIC negative ion mode / 2.1mm i.d. column

## Slide 69
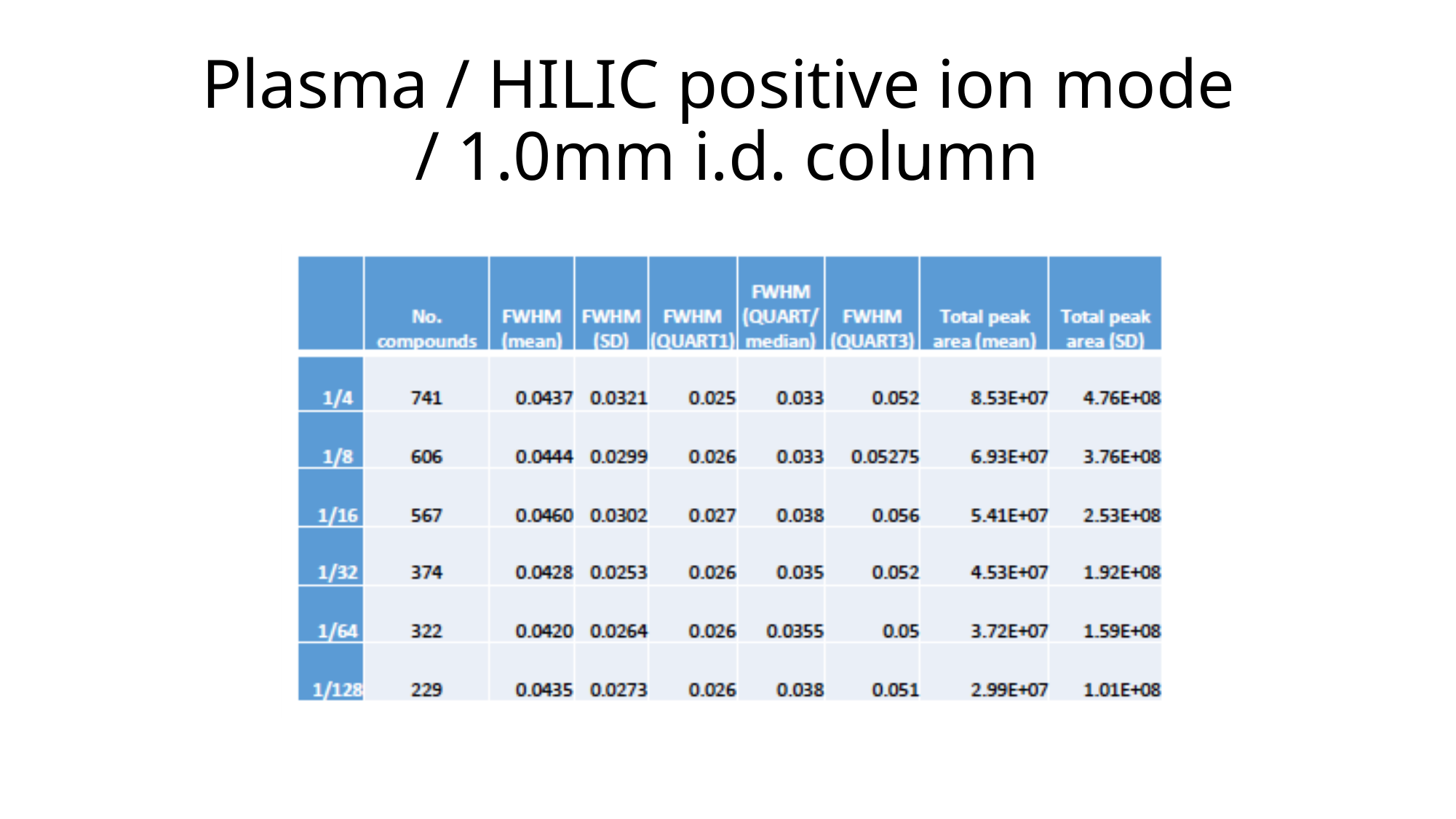

# Plasma / HILIC positive ion mode / 1.0mm i.d. column

## Slide 70
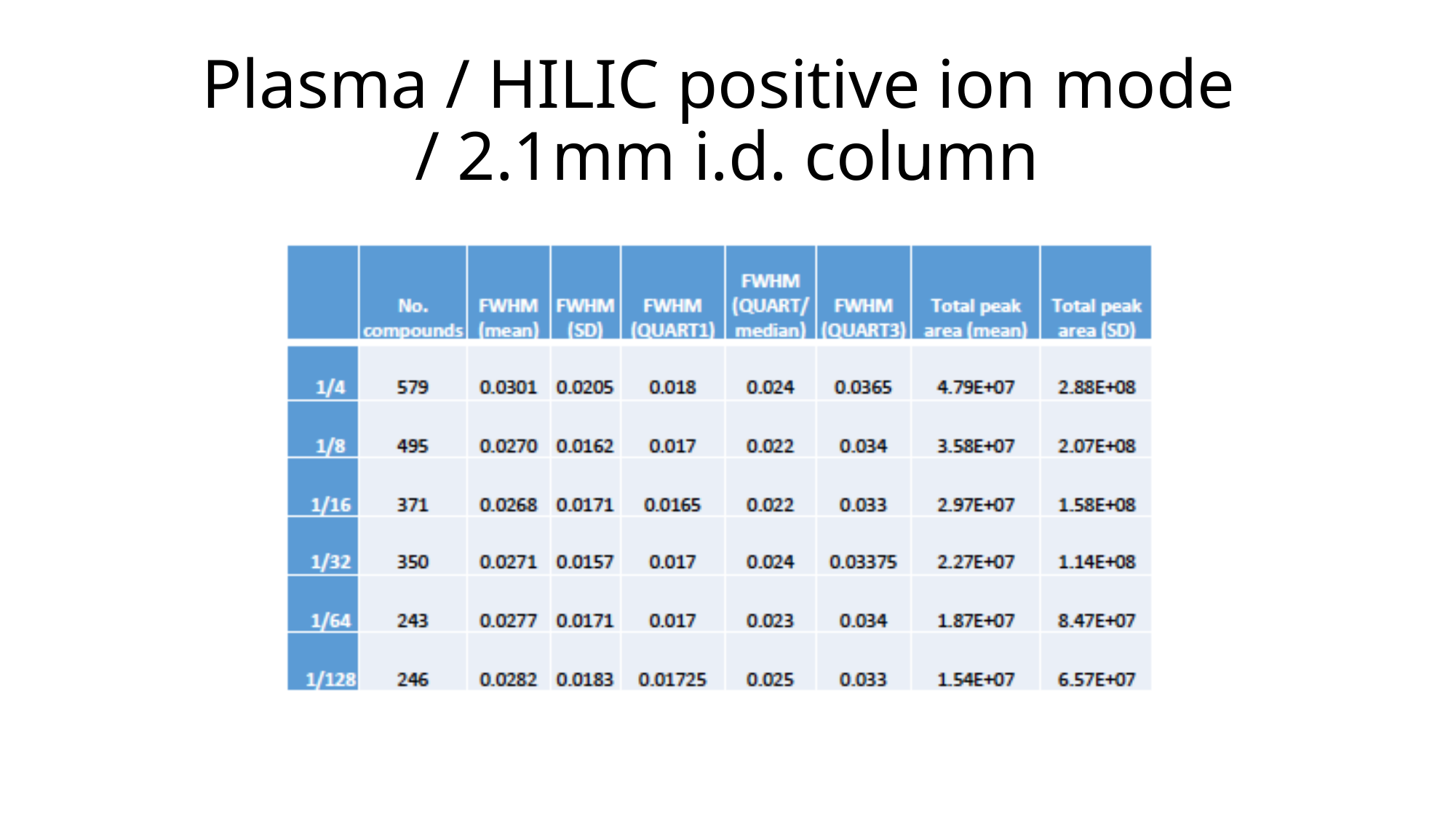

# Plasma / HILIC positive ion mode / 2.1mm i.d. column

## Slide 71
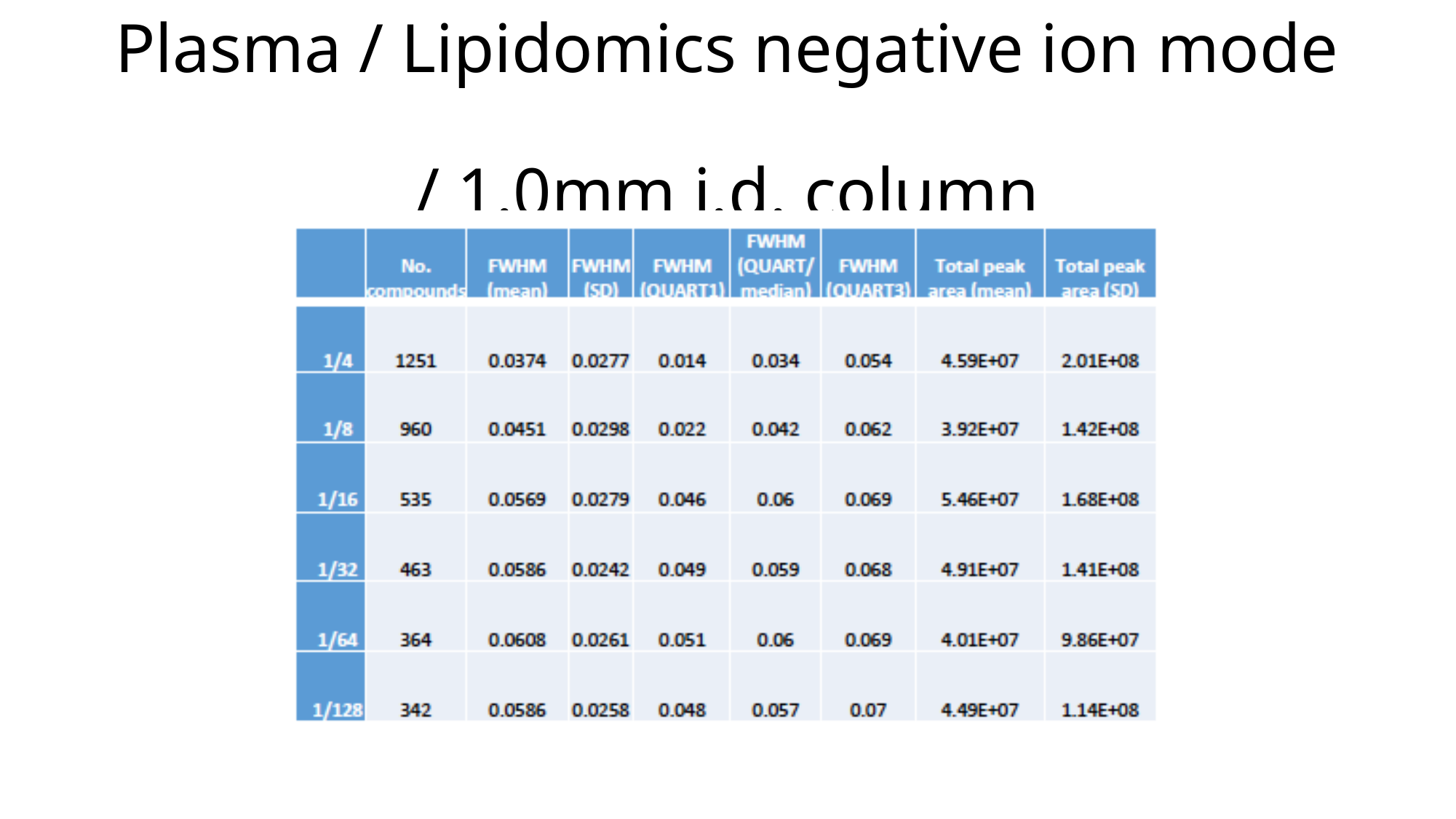

# Plasma / Lipidomics negative ion mode / 1.0mm i.d. column

## Slide 72
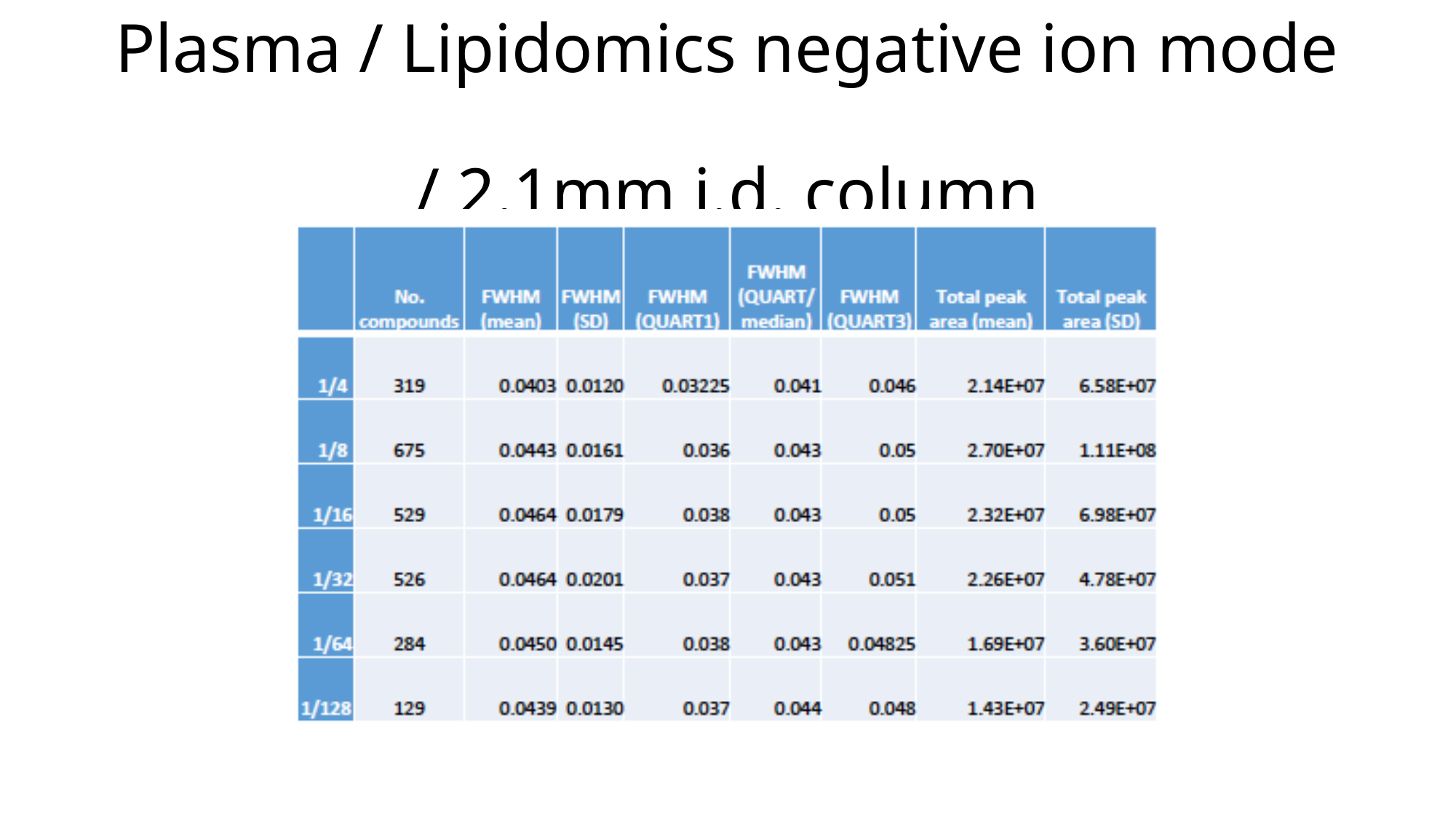

# Plasma / Lipidomics negative ion mode / 2.1mm i.d. column

## Slide 73
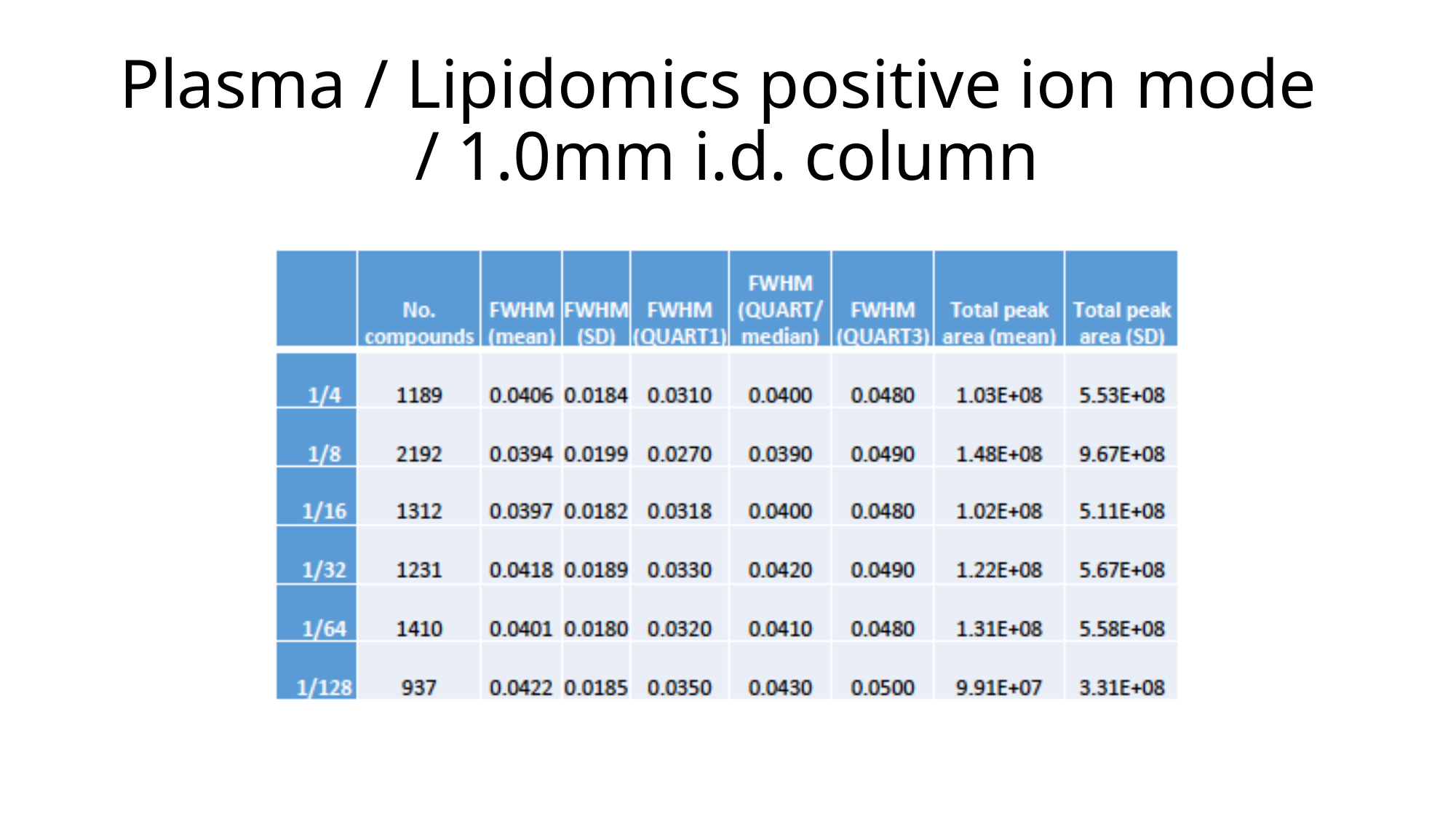

# Plasma / Lipidomics positive ion mode / 1.0mm i.d. column

## Slide 74
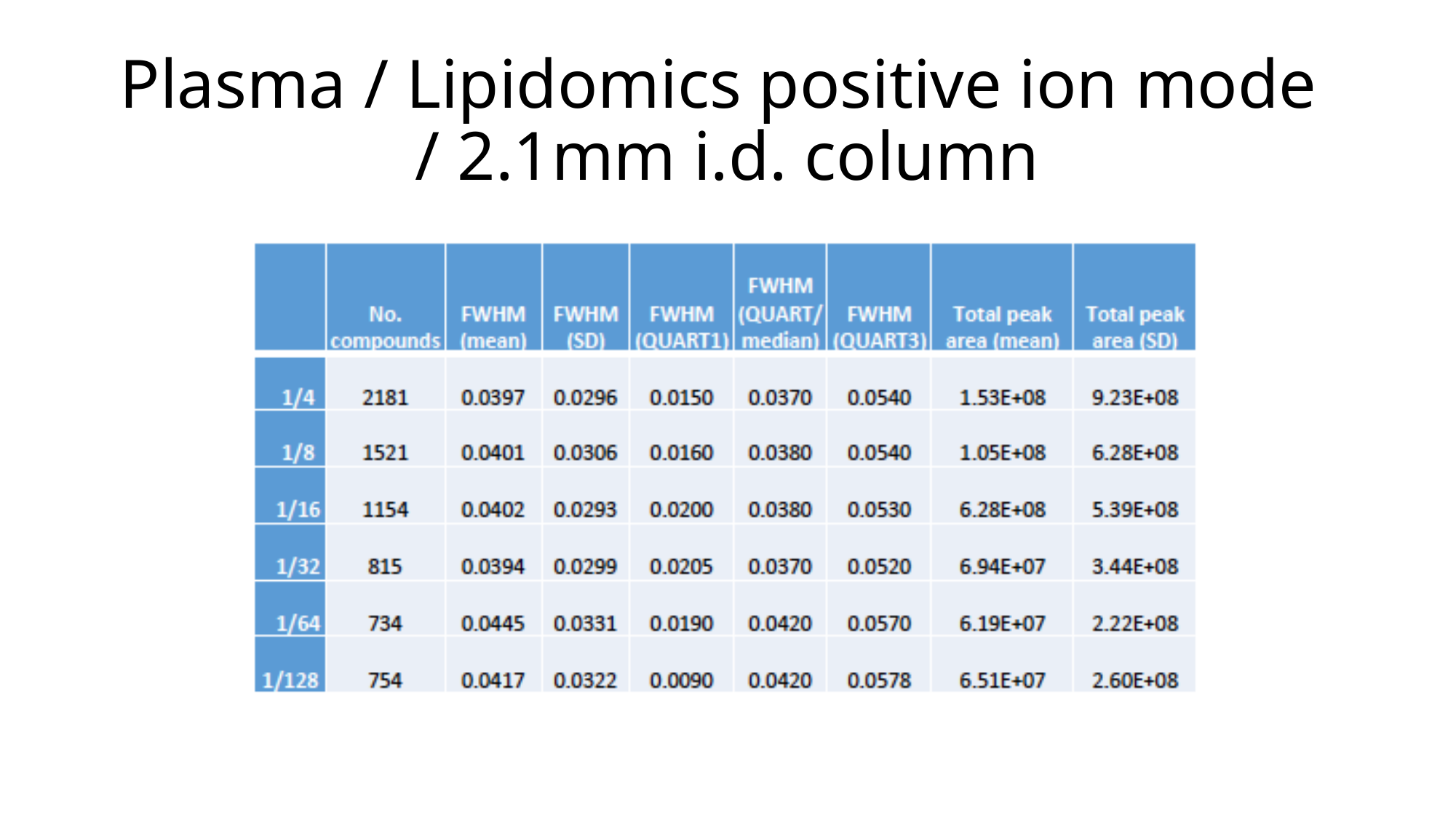

# Plasma / Lipidomics positive ion mode / 2.1mm i.d. column

## Slide 75
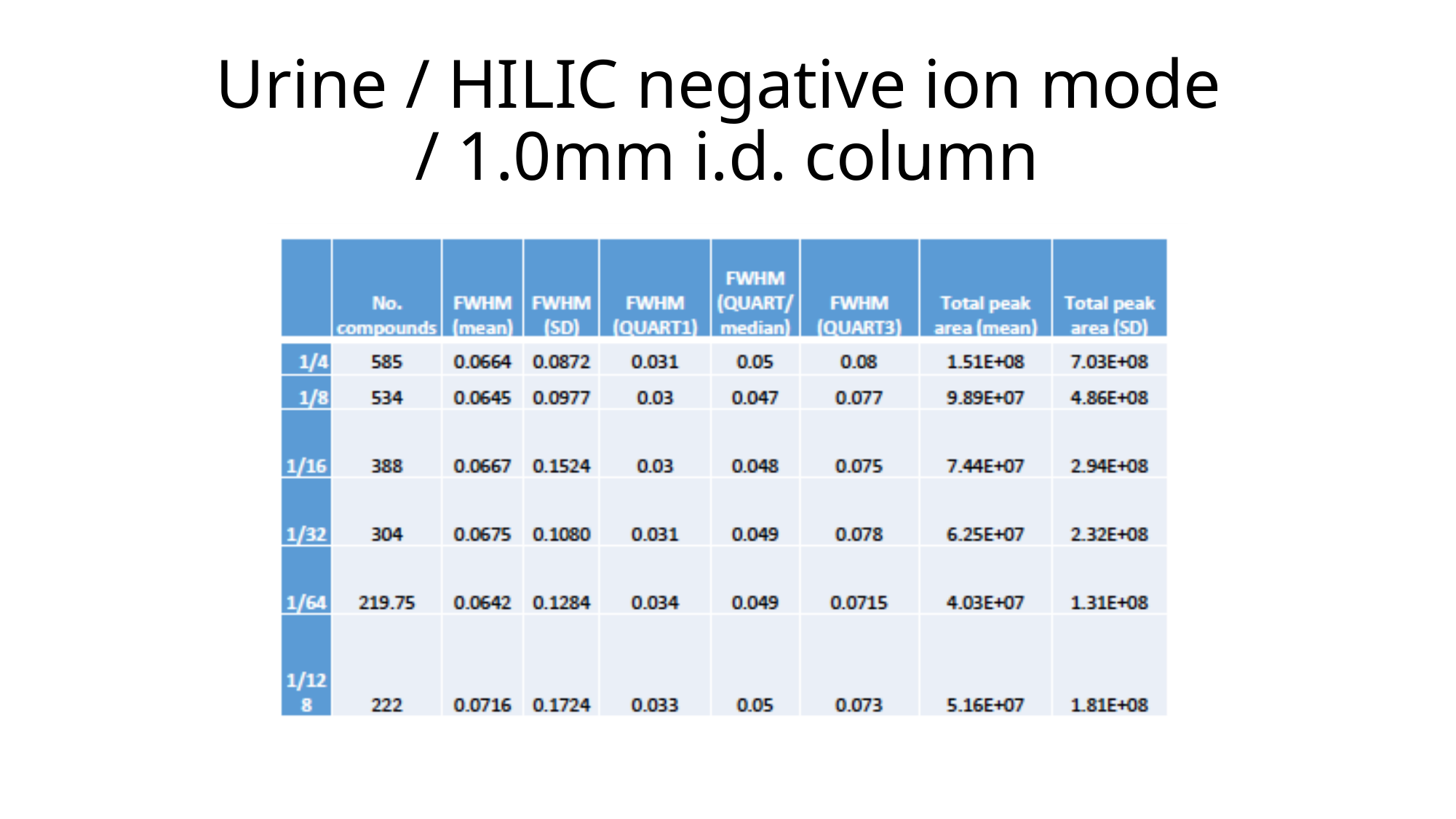

# Urine / HILIC negative ion mode / 1.0mm i.d. column

## Slide 76
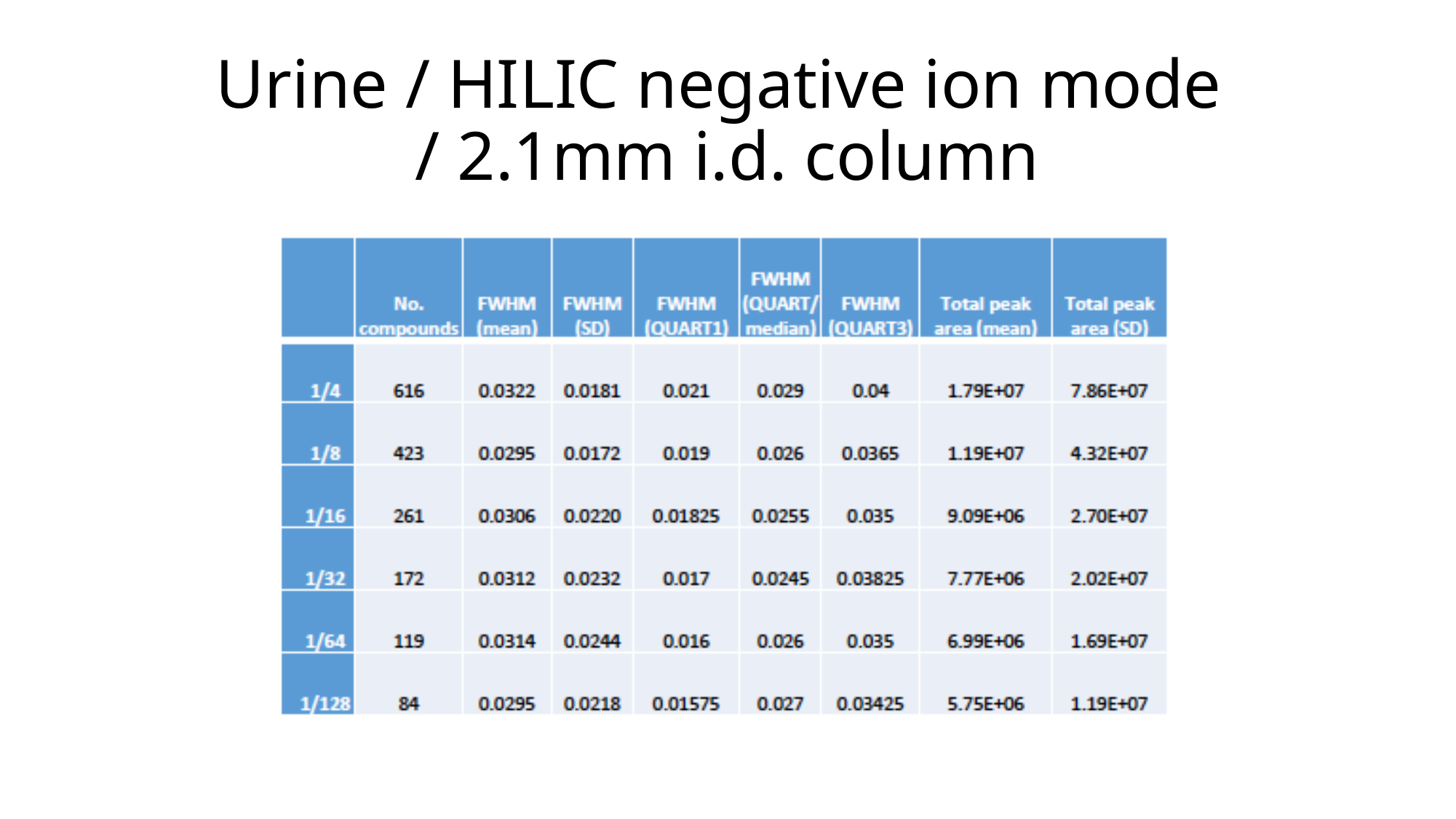

# Urine / HILIC negative ion mode / 2.1mm i.d. column

## Slide 77
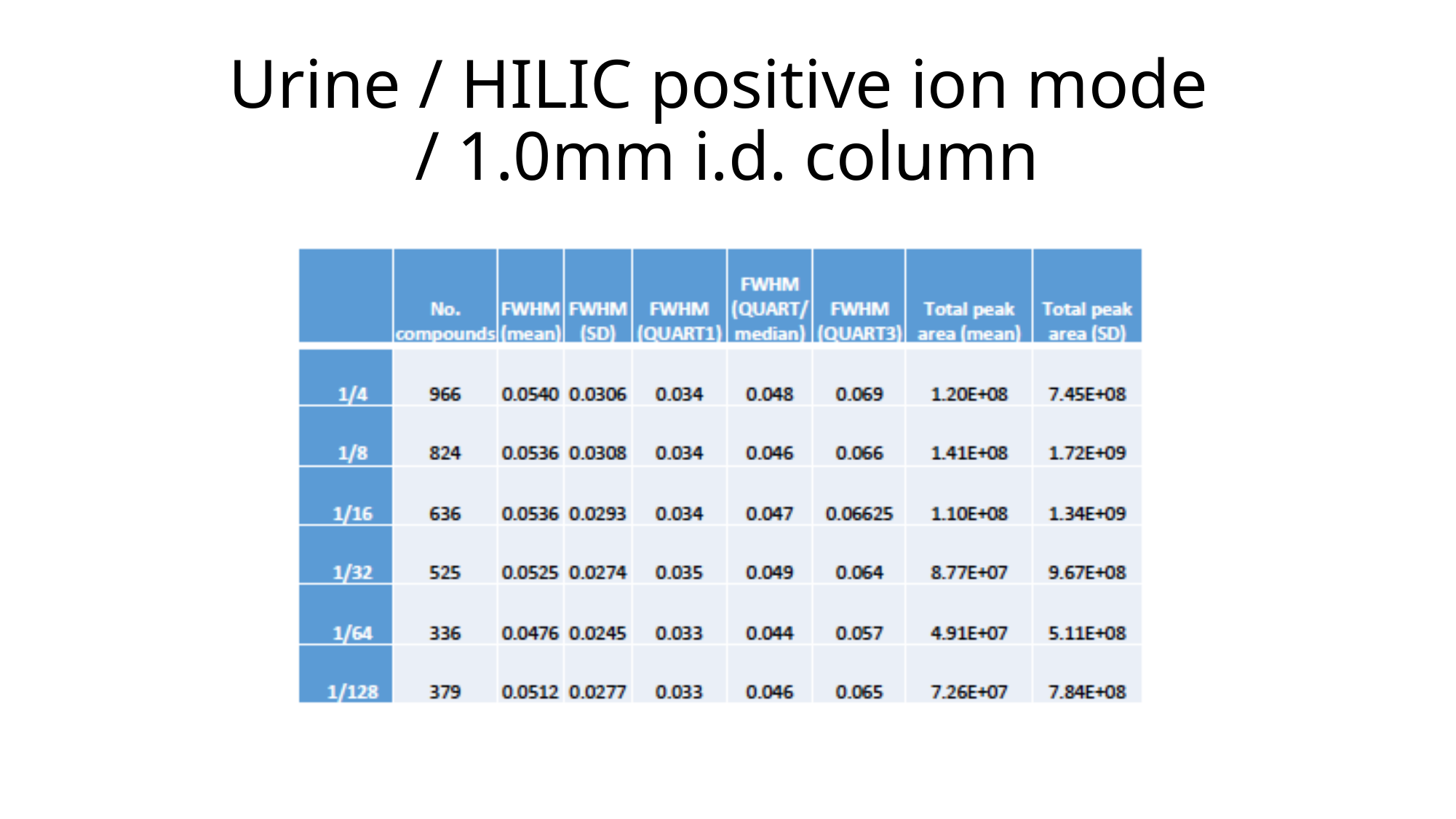

# Urine / HILIC positive ion mode / 1.0mm i.d. column

## Slide 78
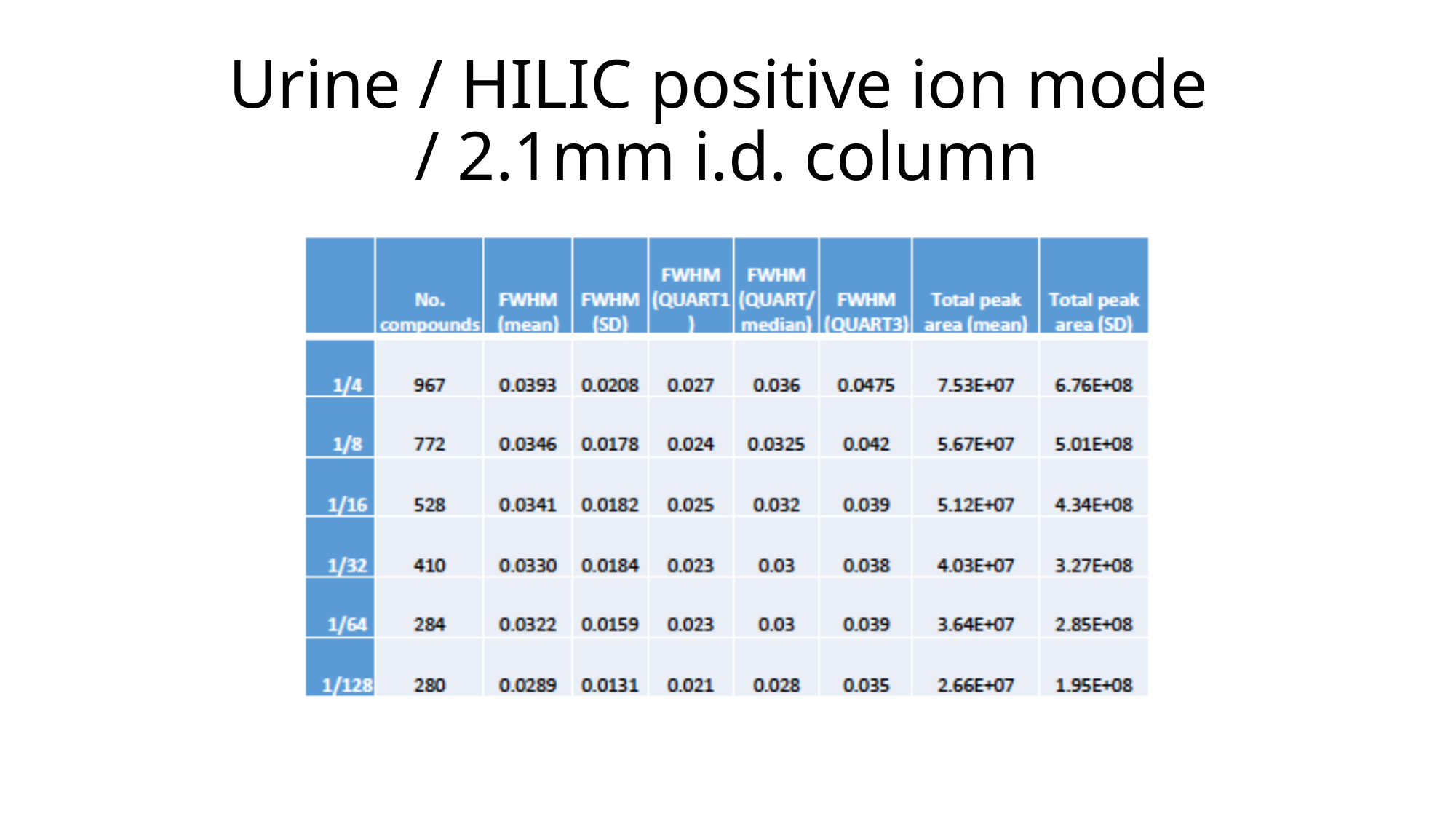

# Urine / HILIC positive ion mode / 2.1mm i.d. column

## Slide 79
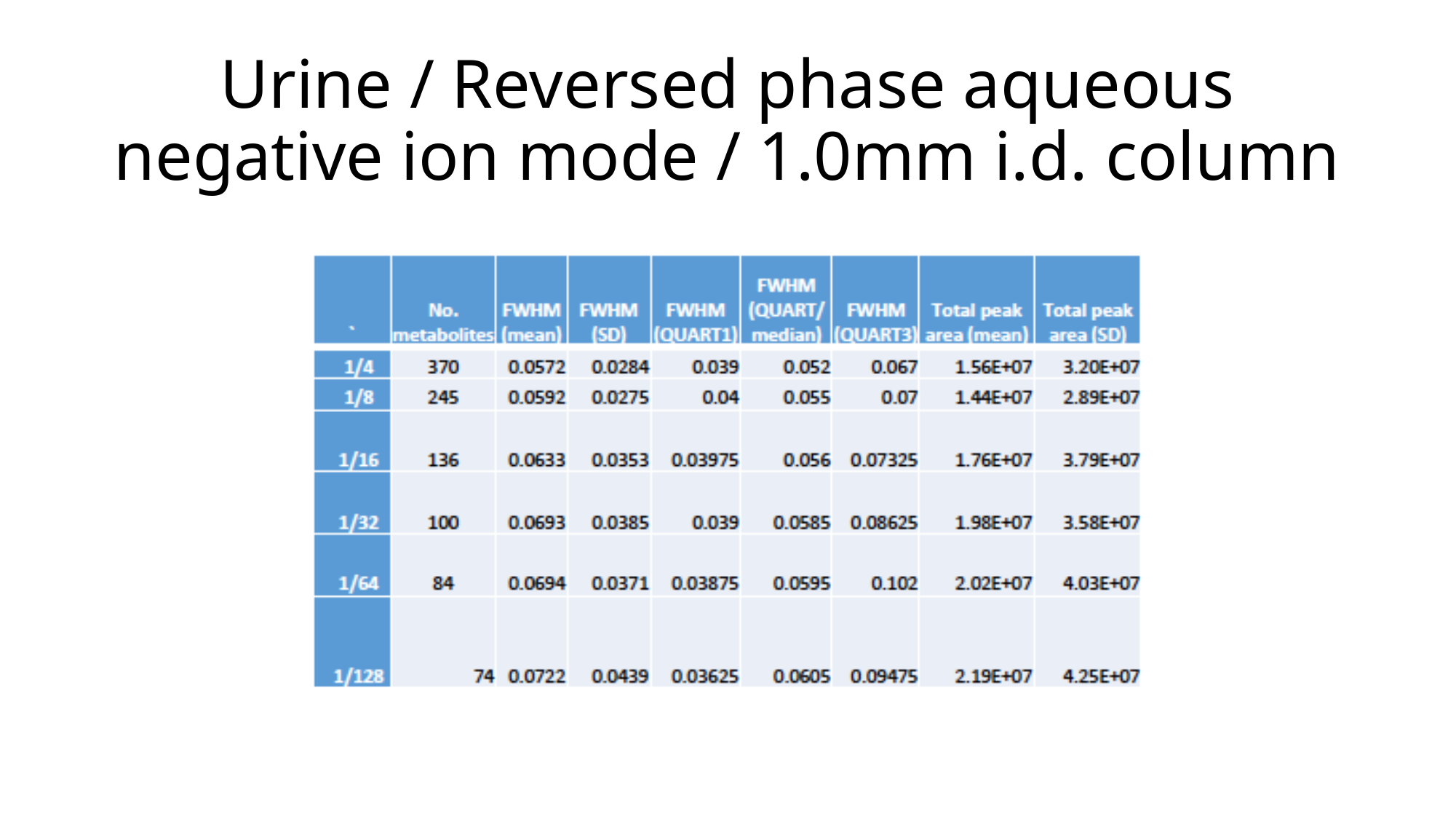

# Urine / Reversed phase aqueous negative ion mode / 1.0mm i.d. column

## Slide 80
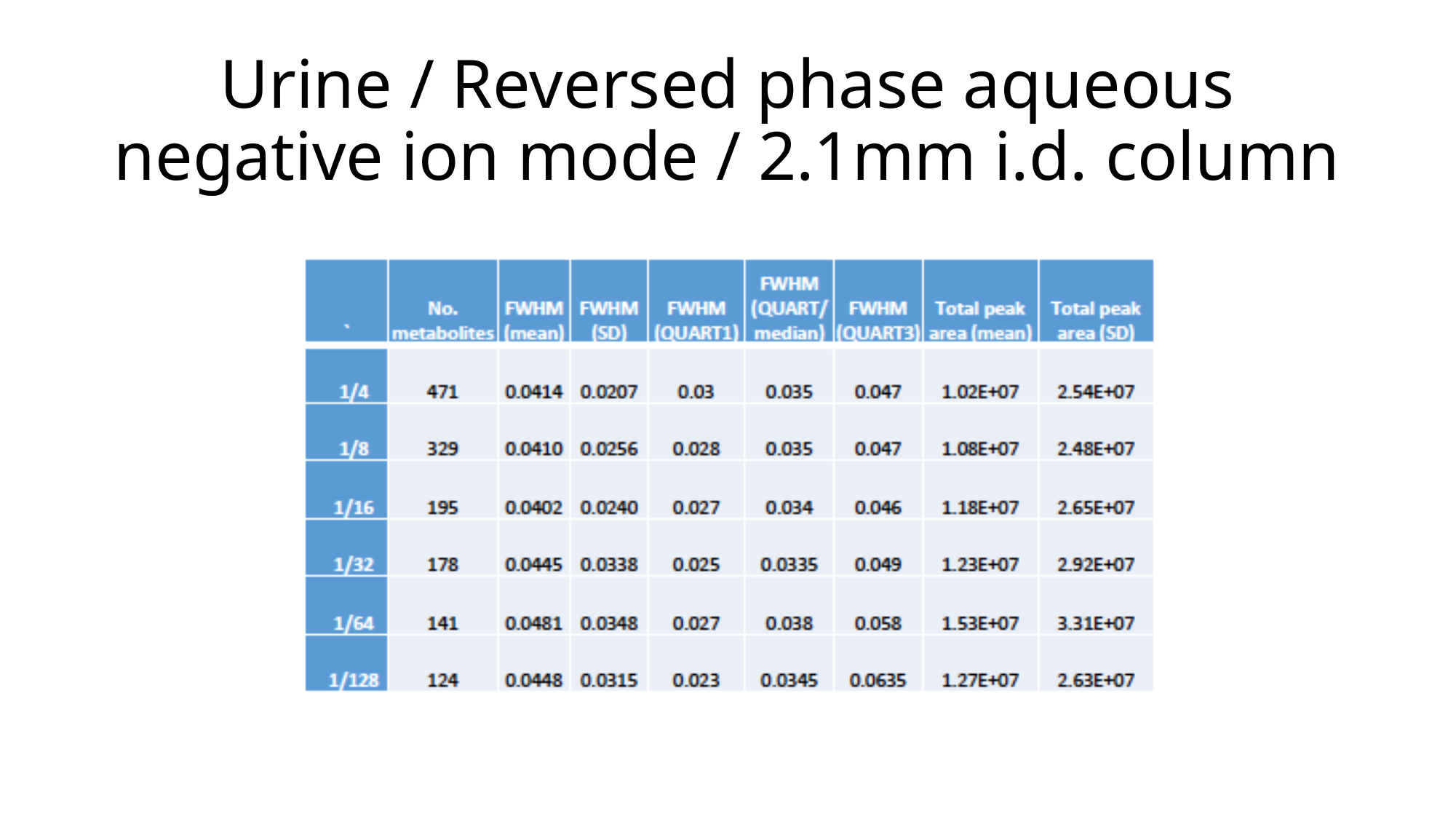

# Urine / Reversed phase aqueous negative ion mode / 2.1mm i.d. column

## Slide 81
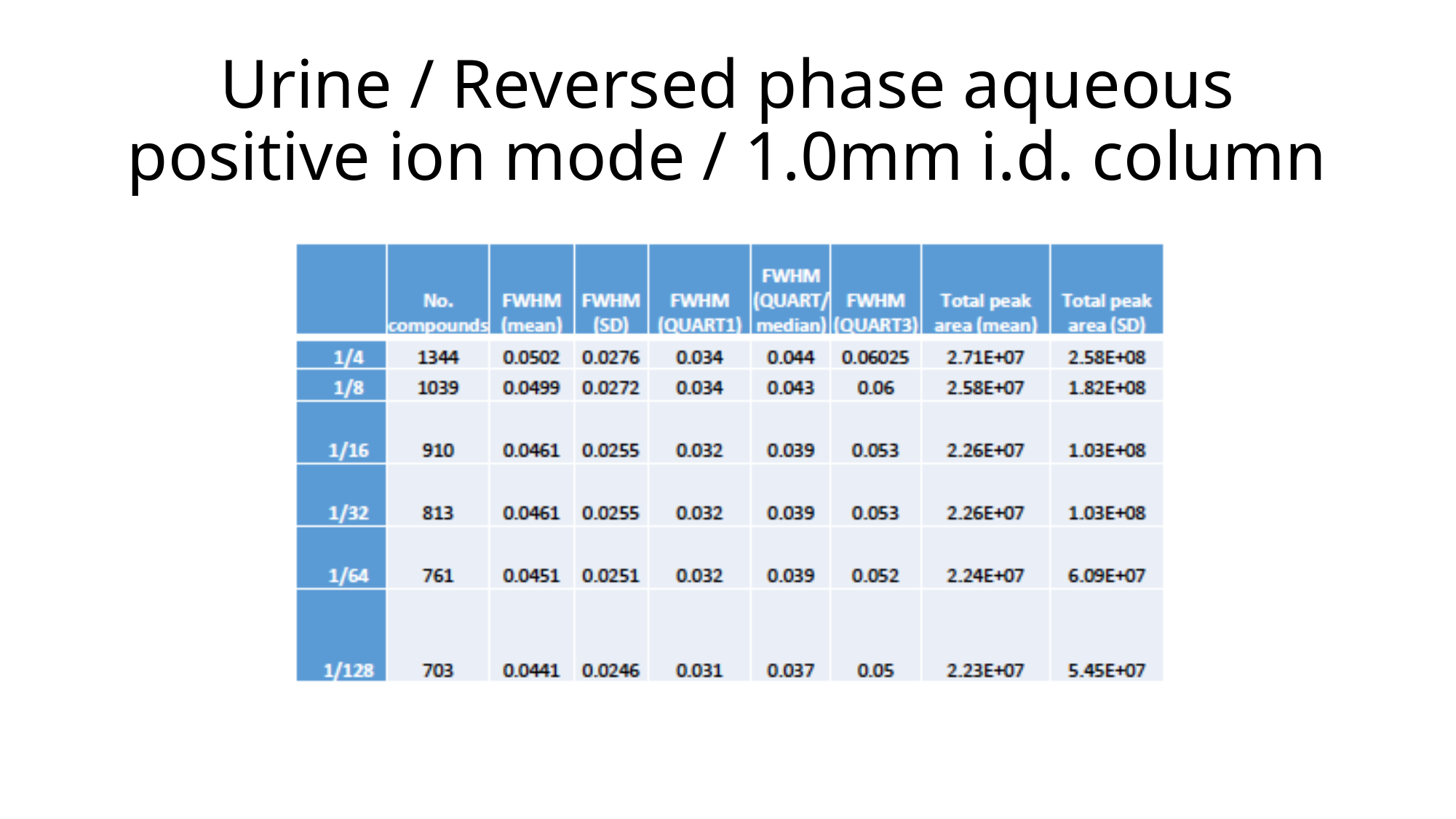

# Urine / Reversed phase aqueous positive ion mode / 1.0mm i.d. column

## Slide 82
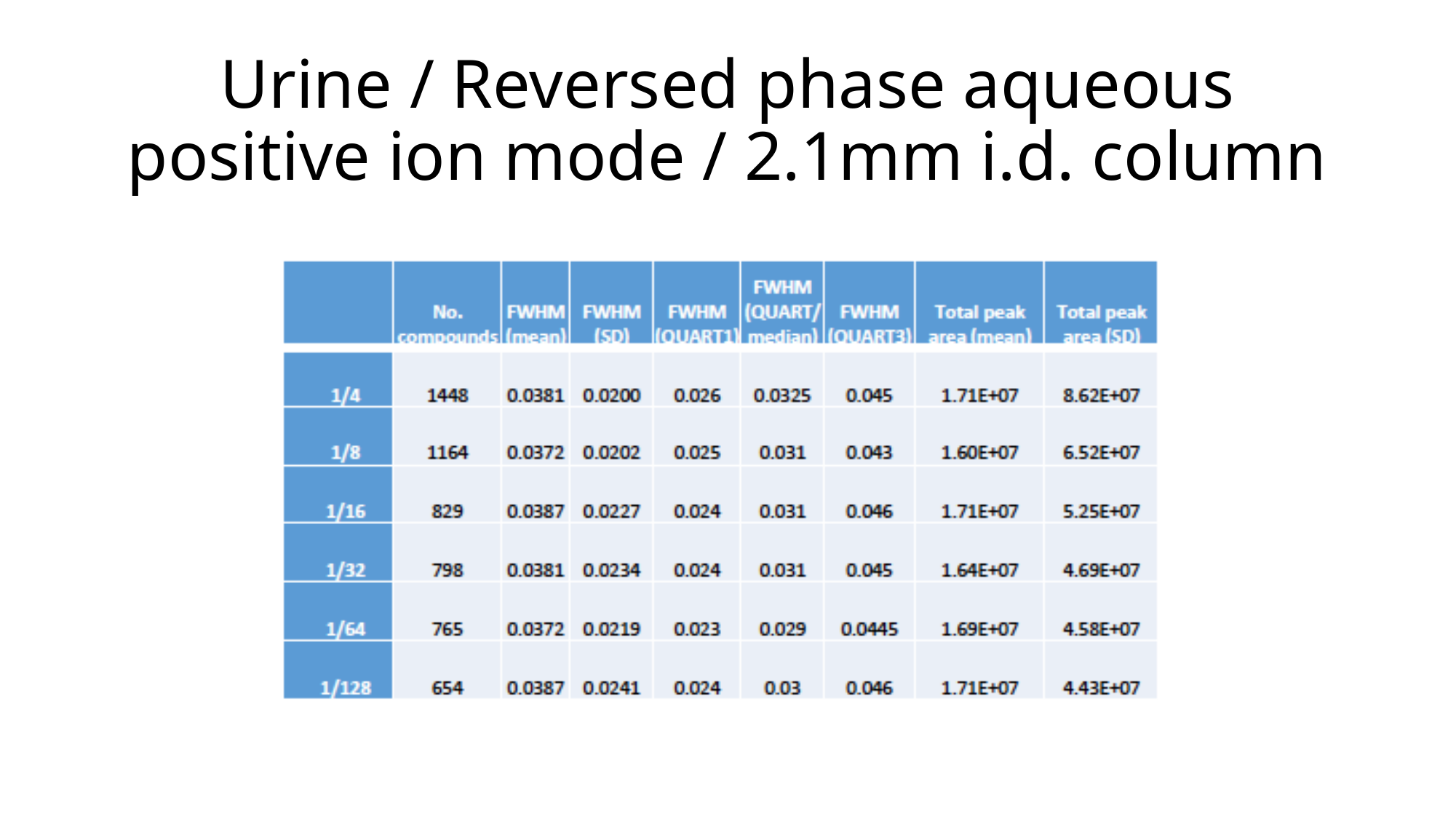

# Urine / Reversed phase aqueous positive ion mode / 2.1mm i.d. column

## Slide 83
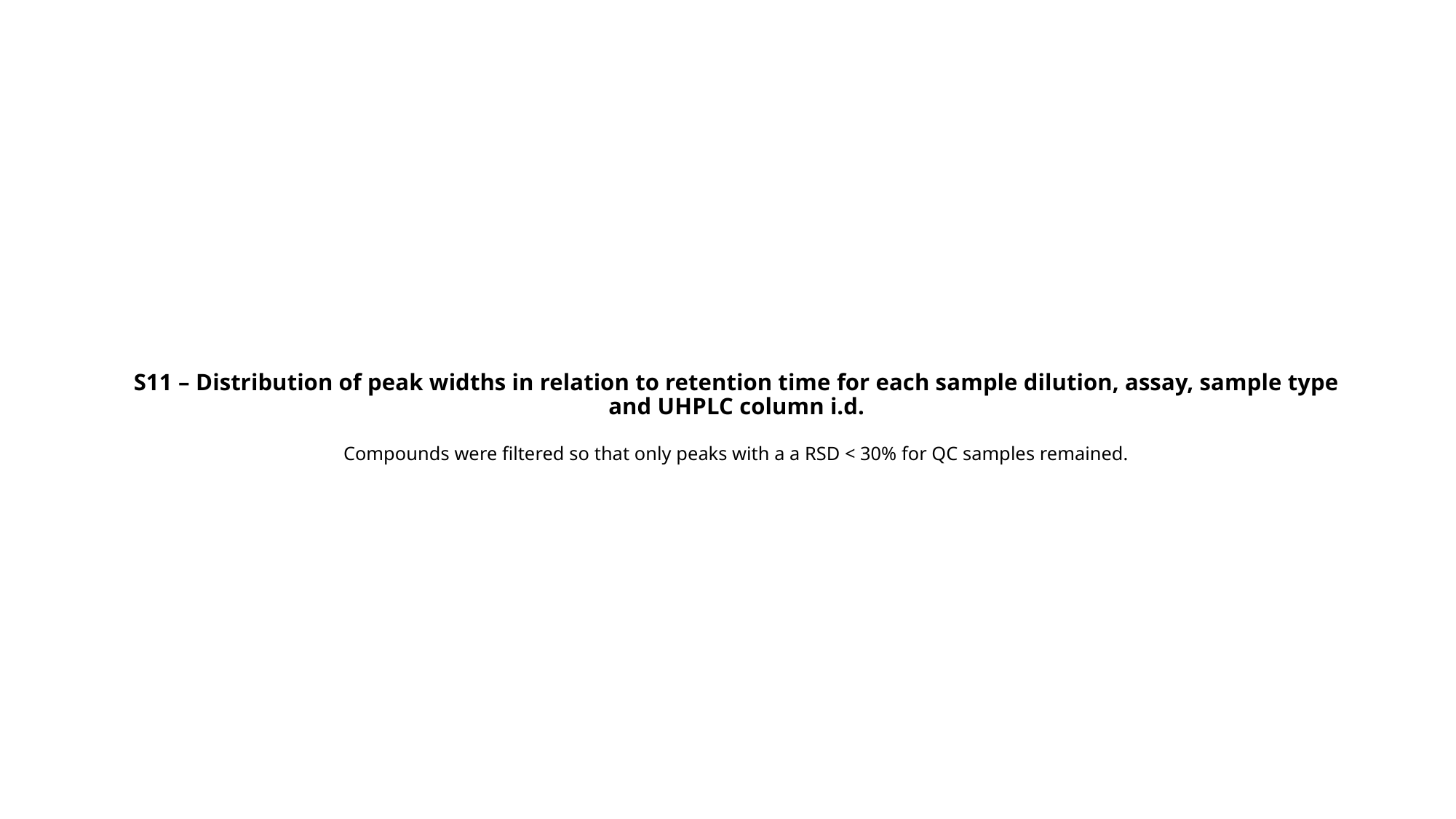

# S11 – Distribution of peak widths in relation to retention time for each sample dilution, assay, sample type and UHPLC column i.d. Compounds were filtered so that only peaks with a a RSD < 30% for QC samples remained.

## Slide 84
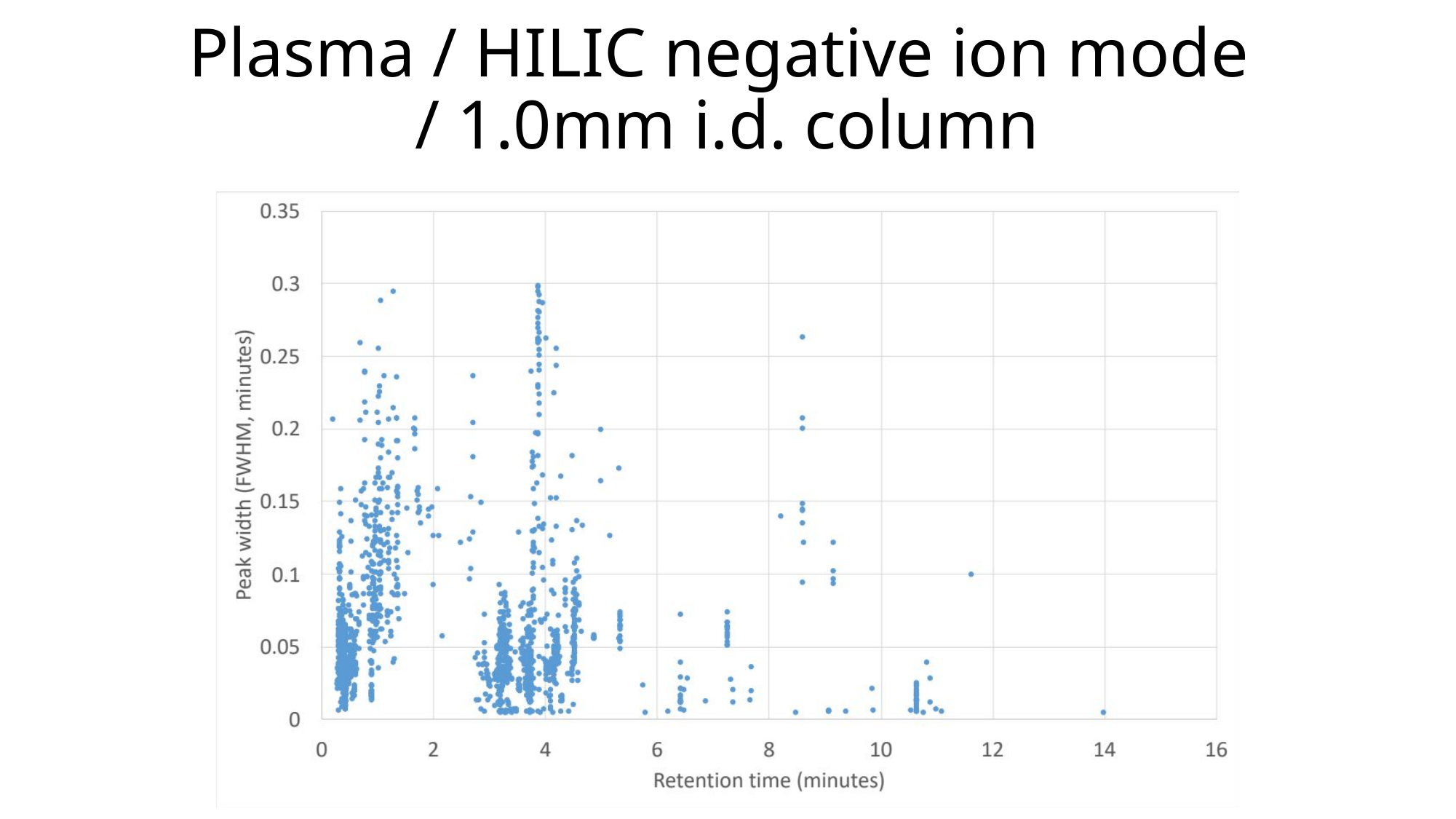

# Plasma / HILIC negative ion mode / 1.0mm i.d. column

## Slide 85
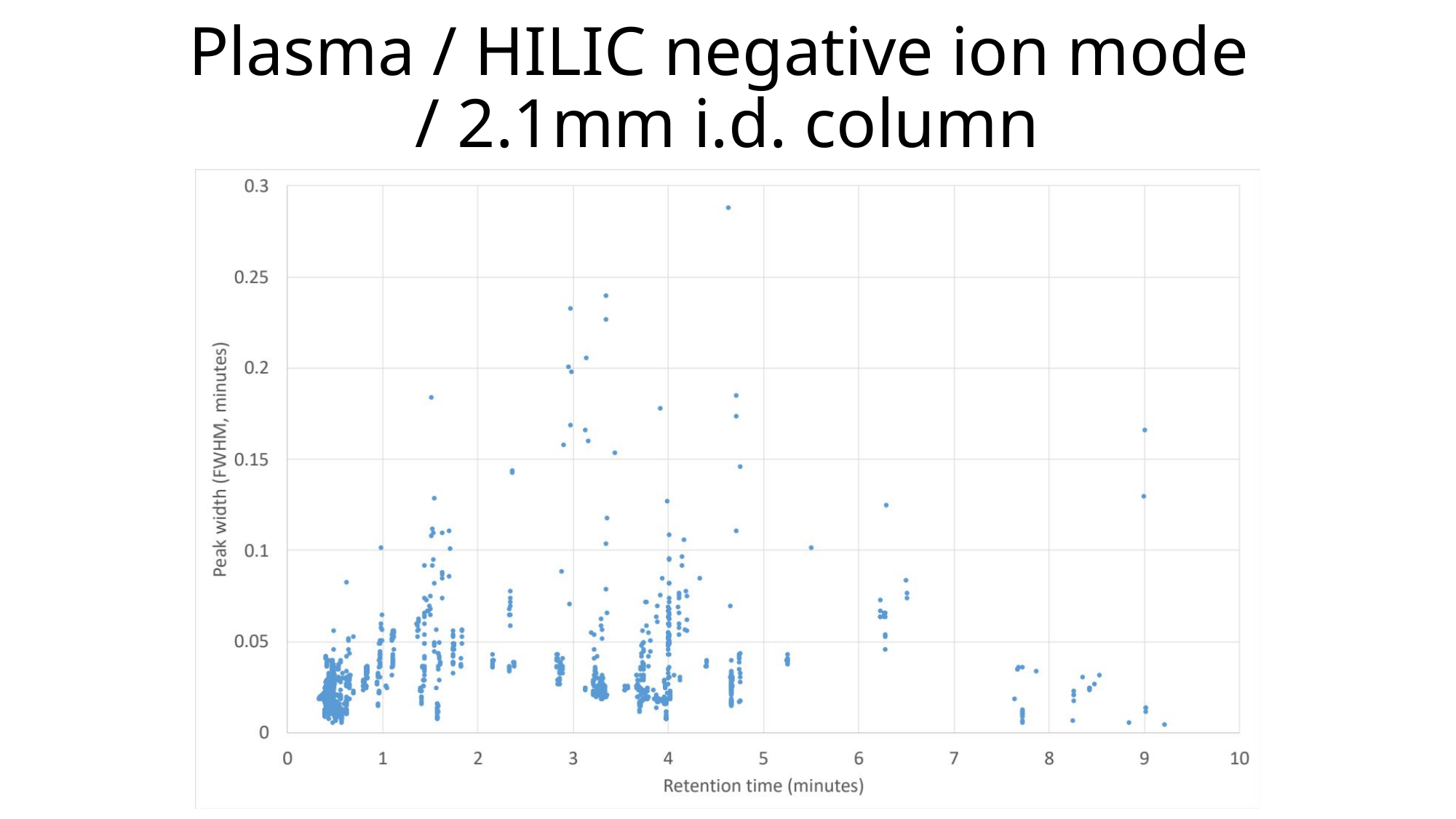

# Plasma / HILIC negative ion mode / 2.1mm i.d. column

## Slide 86
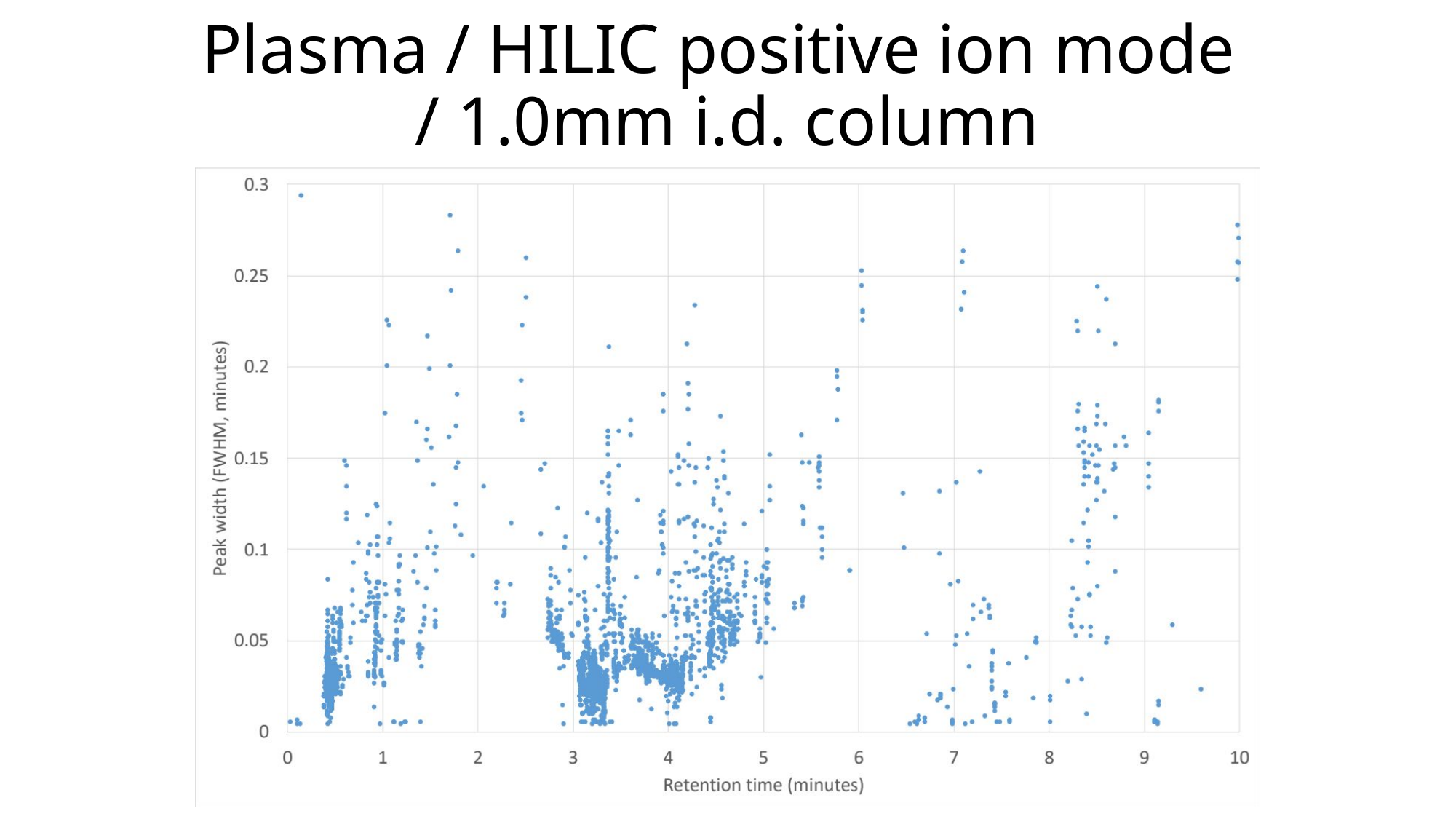

# Plasma / HILIC positive ion mode / 1.0mm i.d. column

## Slide 87
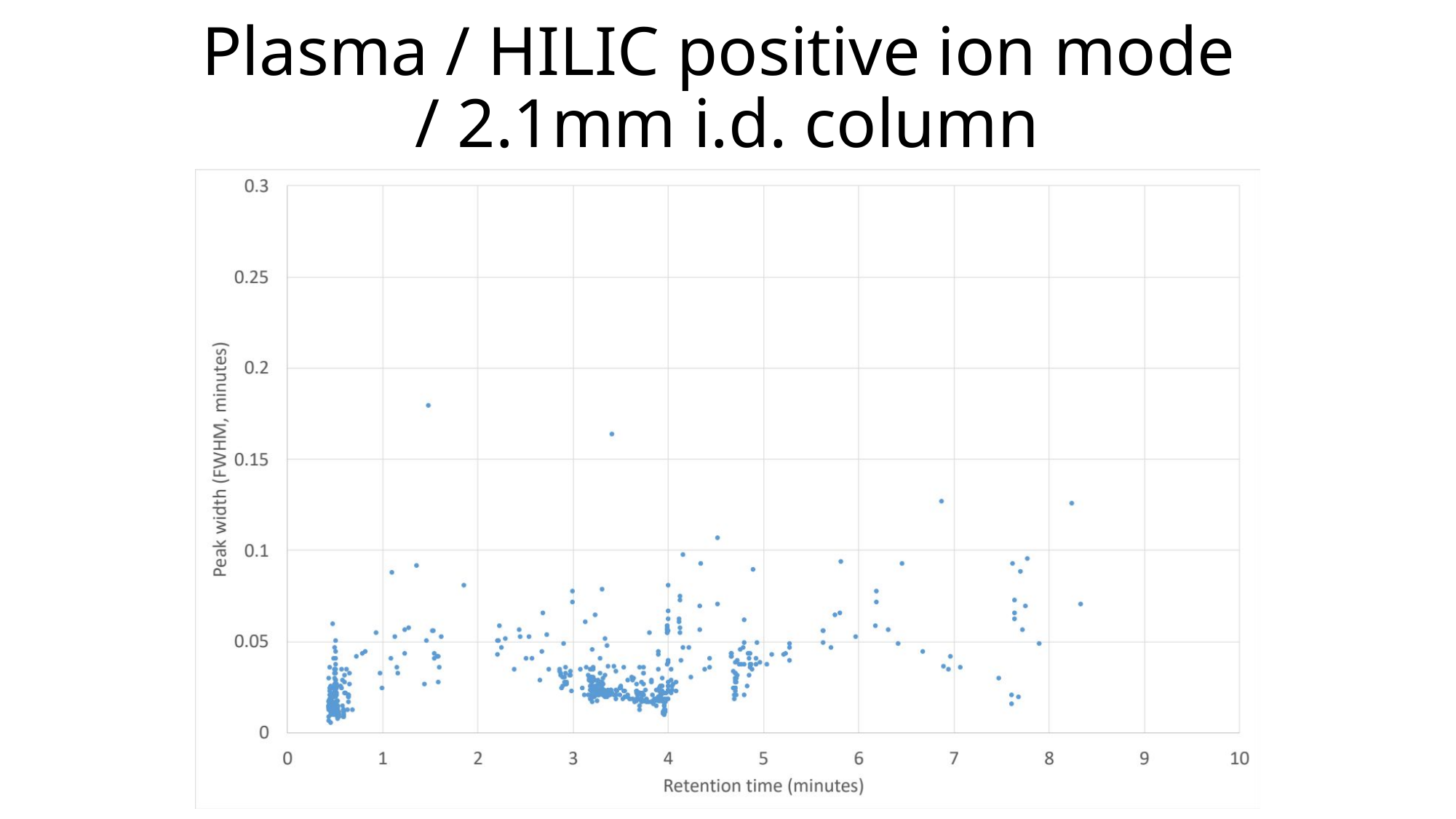

# Plasma / HILIC positive ion mode / 2.1mm i.d. column

## Slide 88
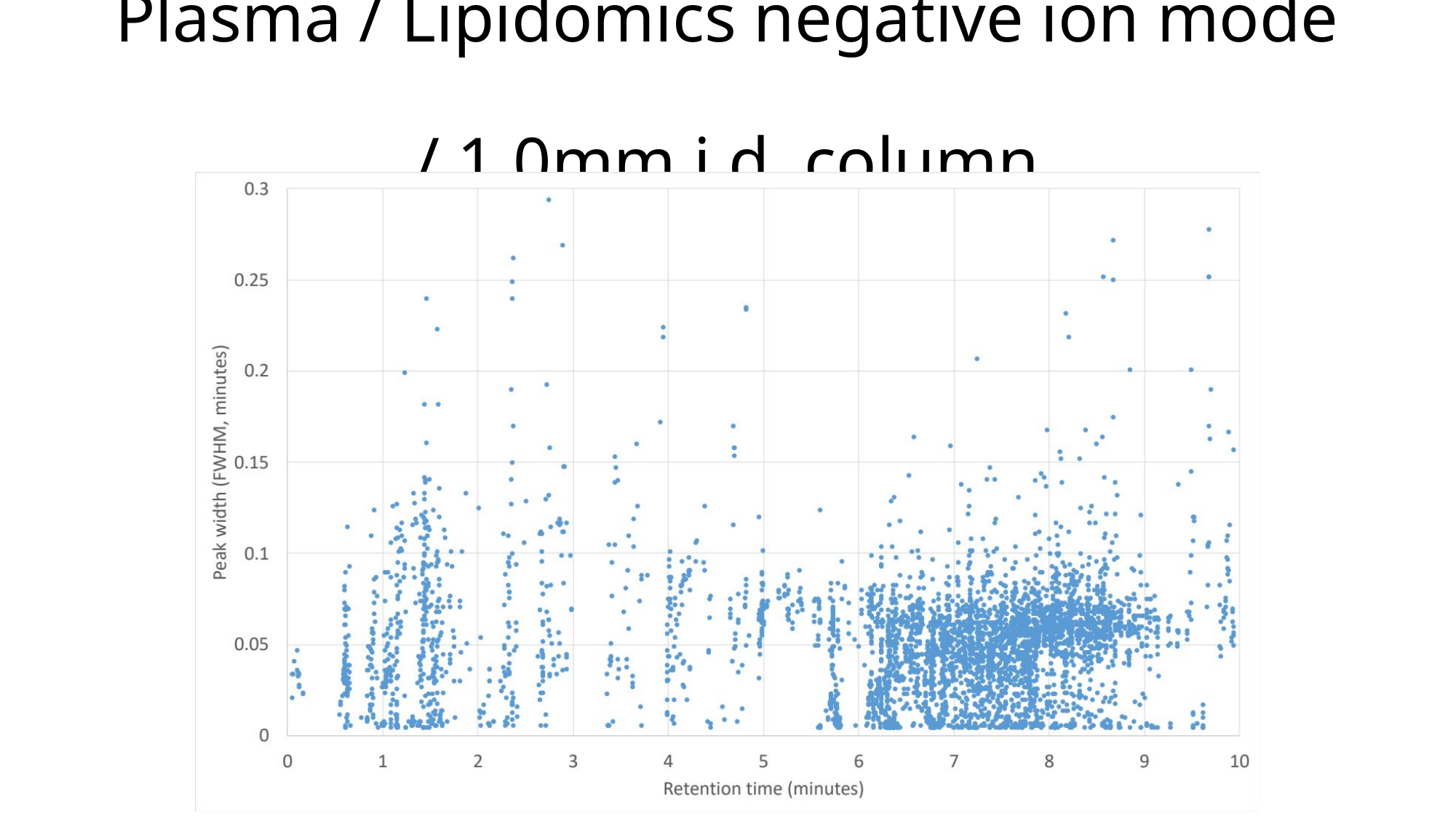

# Plasma / Lipidomics negative ion mode / 1.0mm i.d. column

## Slide 89
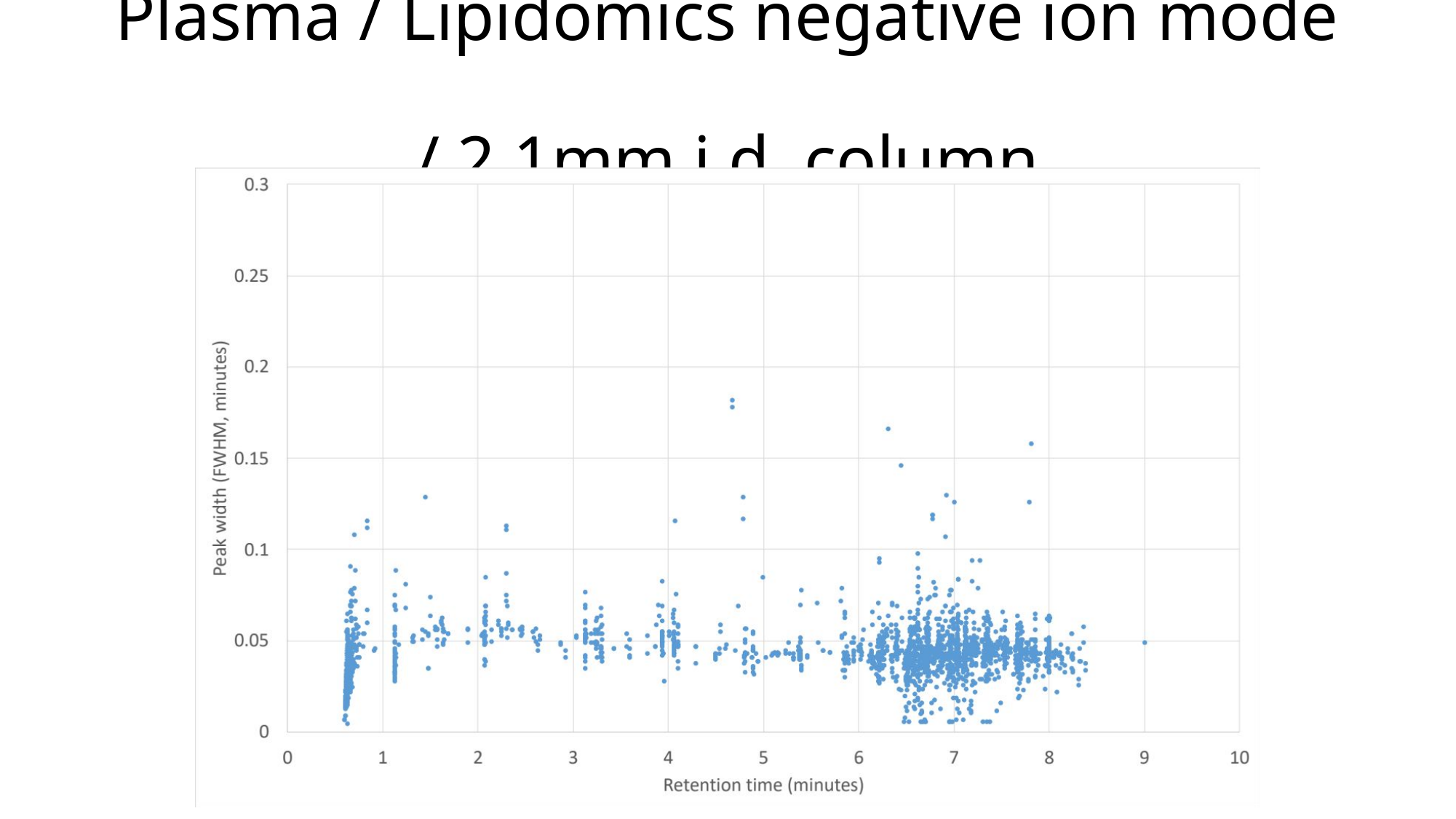

# Plasma / Lipidomics negative ion mode / 2.1mm i.d. column

## Slide 90
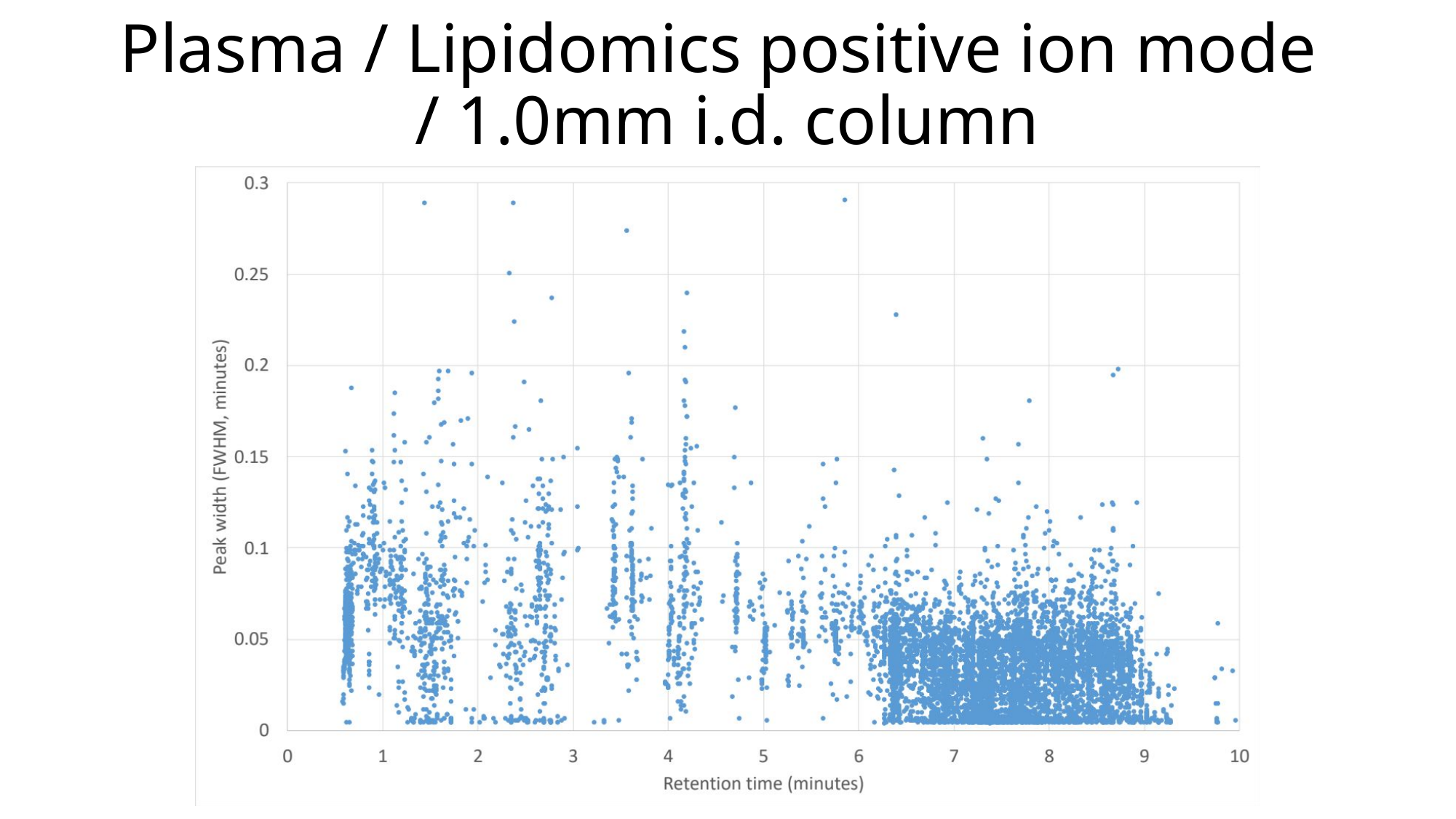

# Plasma / Lipidomics positive ion mode / 1.0mm i.d. column

## Slide 91
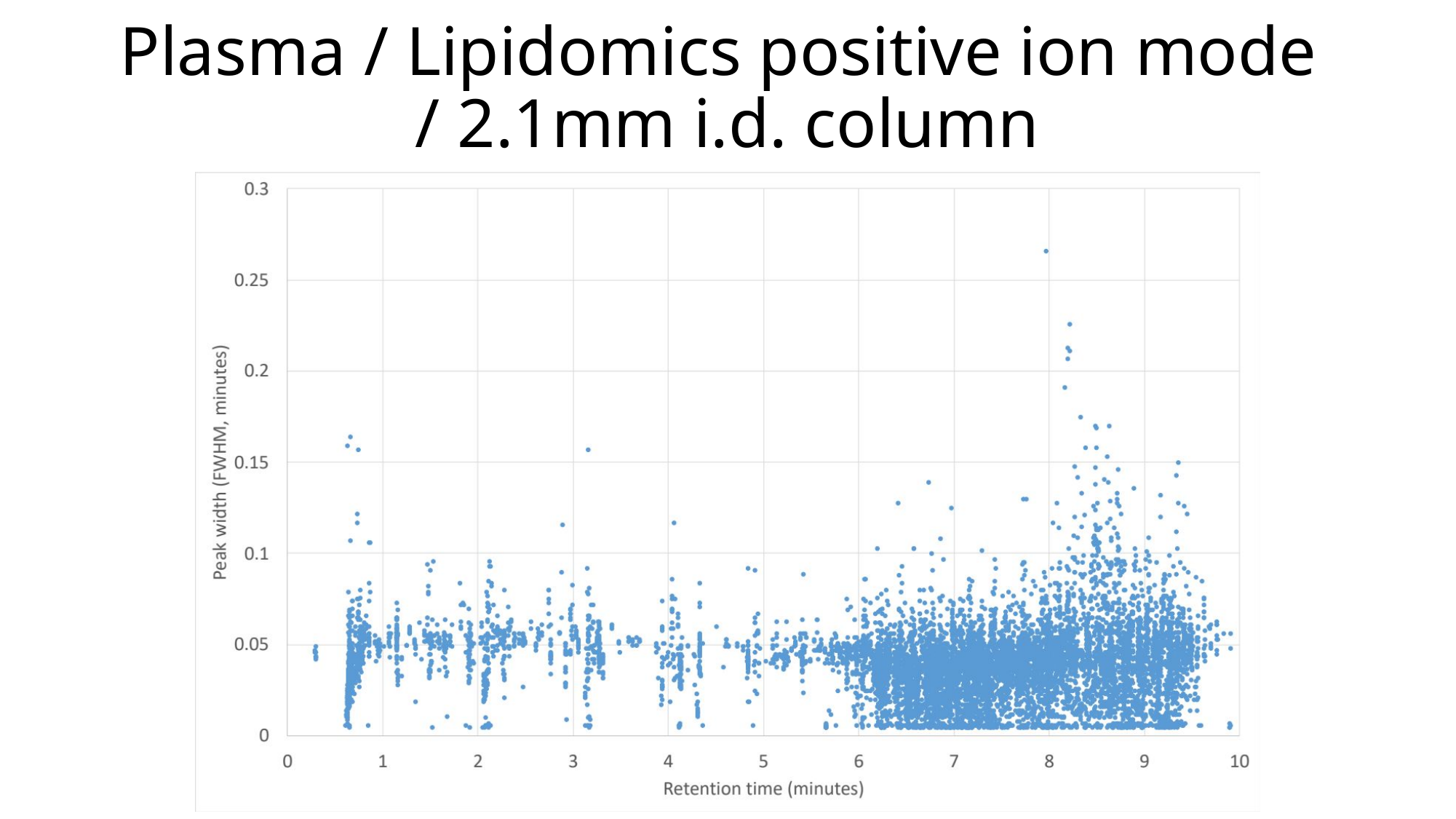

# Plasma / Lipidomics positive ion mode / 2.1mm i.d. column

## Slide 92
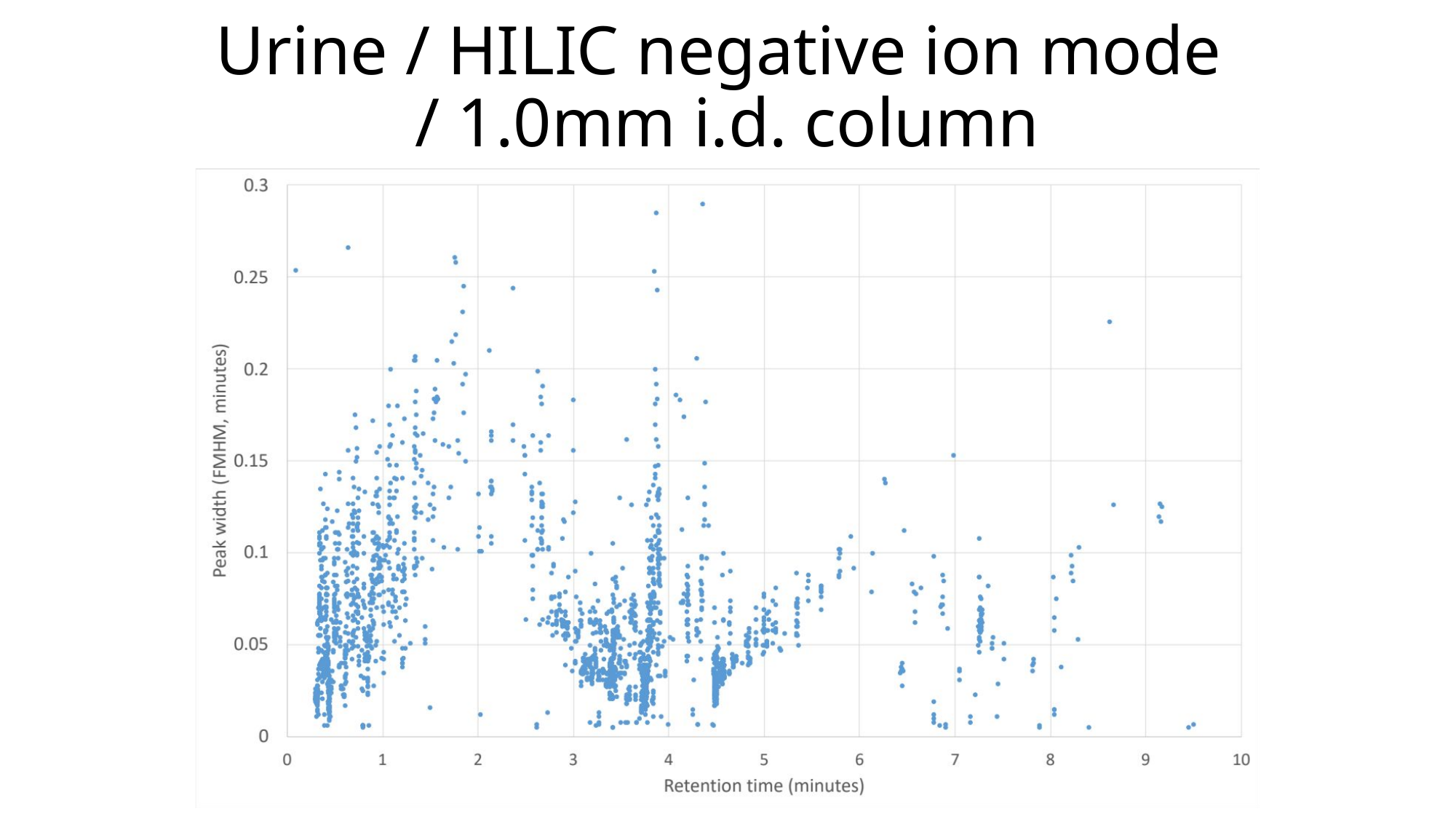

# Urine / HILIC negative ion mode / 1.0mm i.d. column

## Slide 93
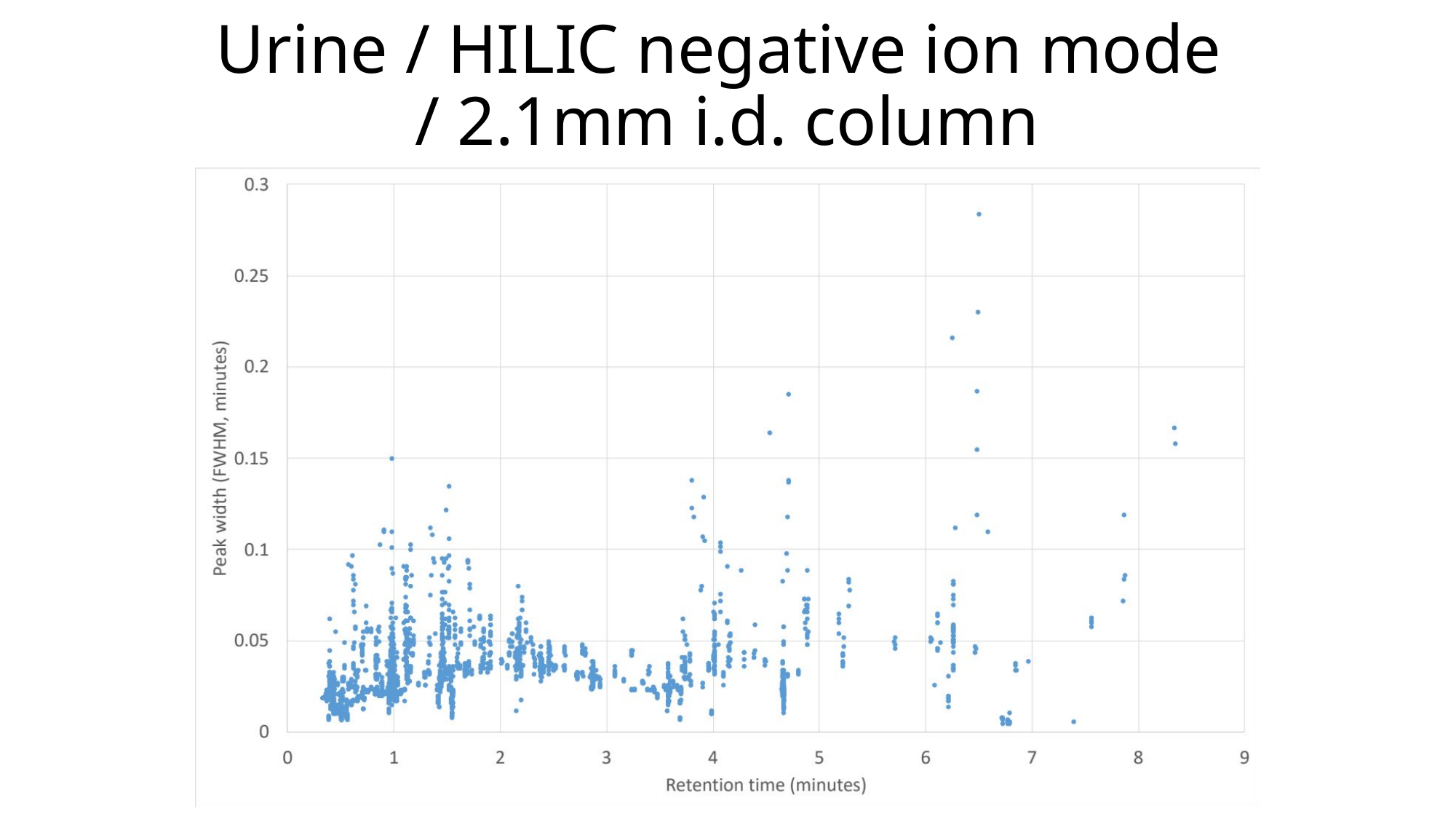

# Urine / HILIC negative ion mode / 2.1mm i.d. column

## Slide 94
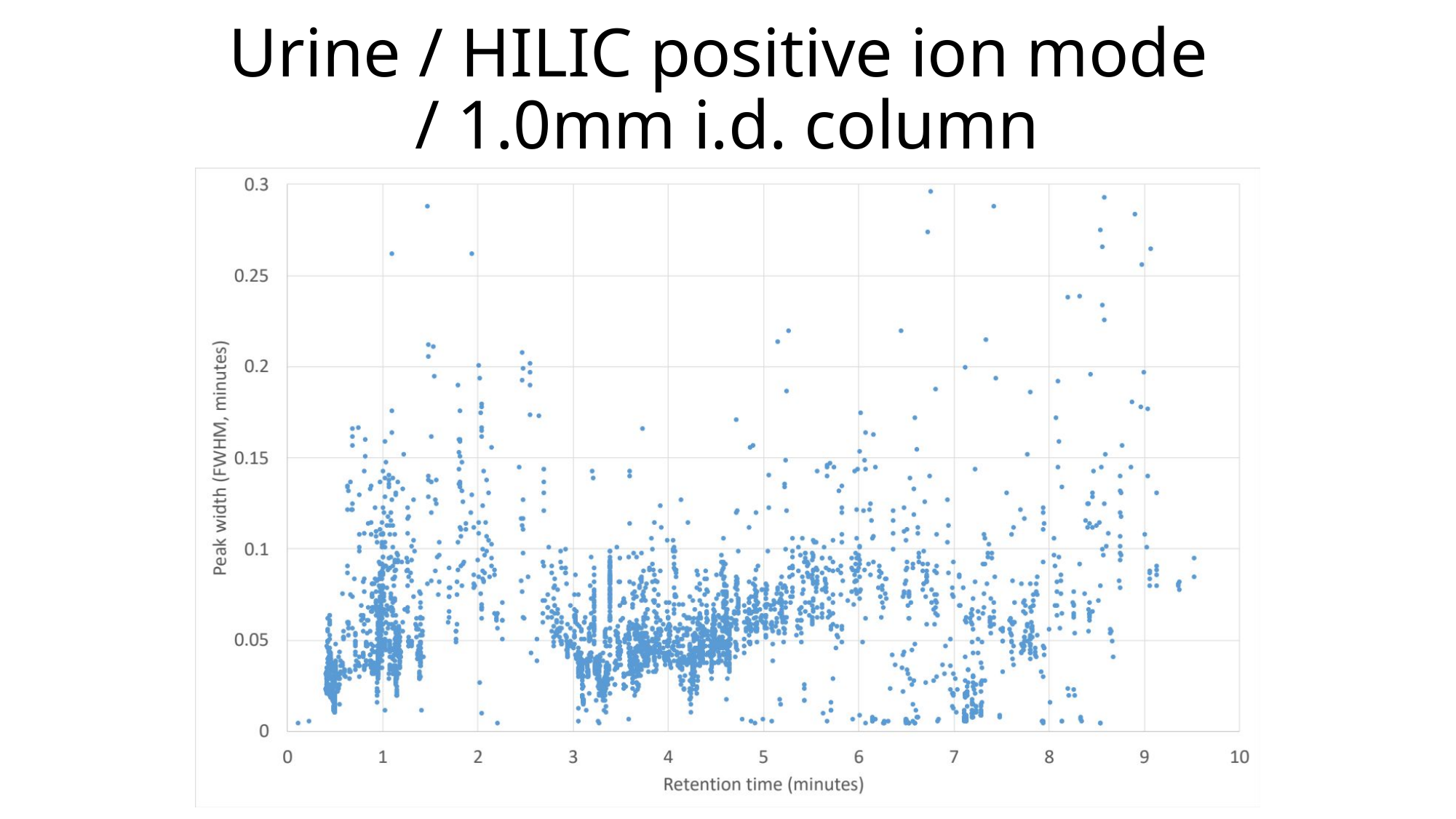

# Urine / HILIC positive ion mode / 1.0mm i.d. column

## Slide 95
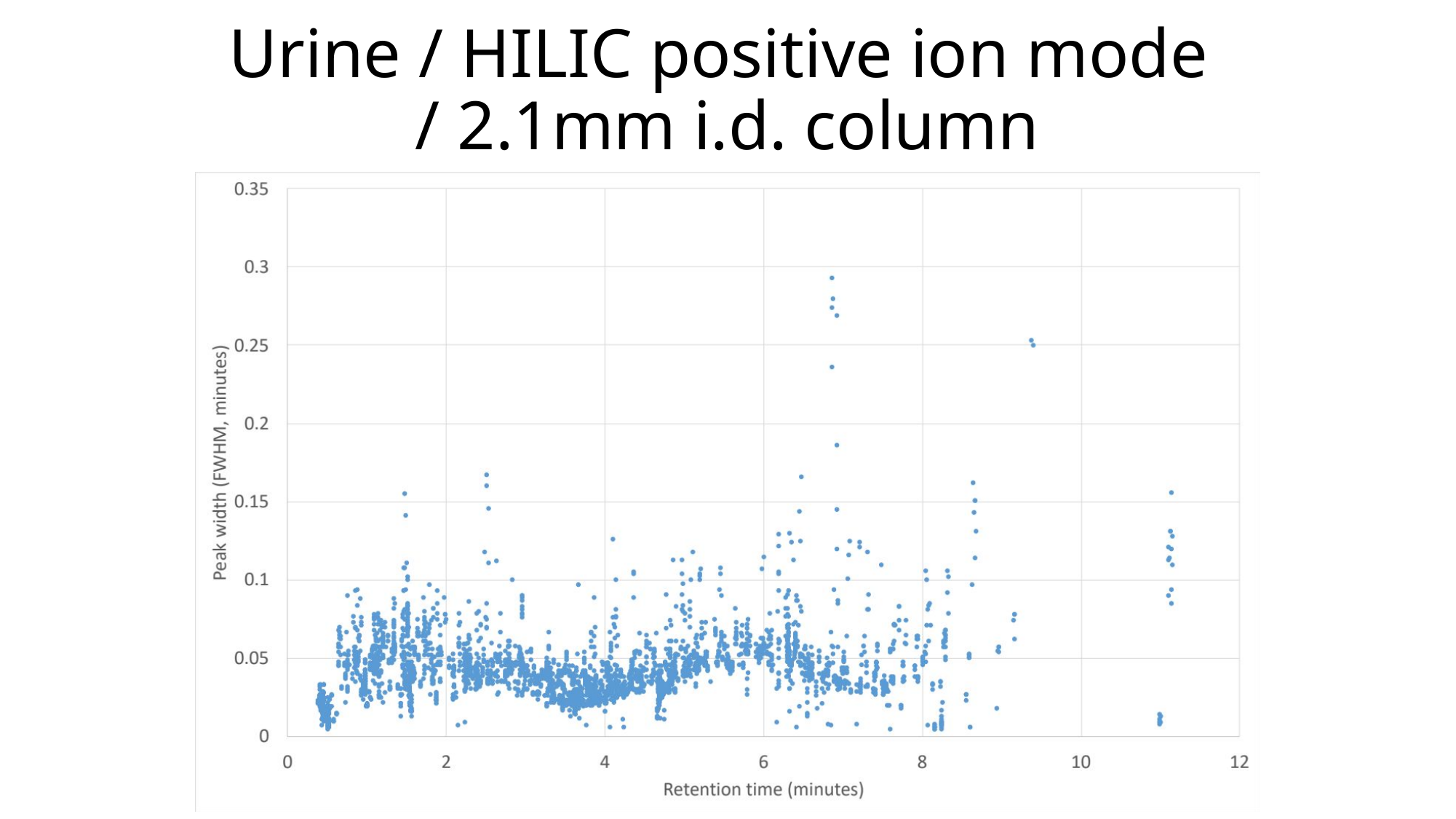

# Urine / HILIC positive ion mode / 2.1mm i.d. column

## Slide 96
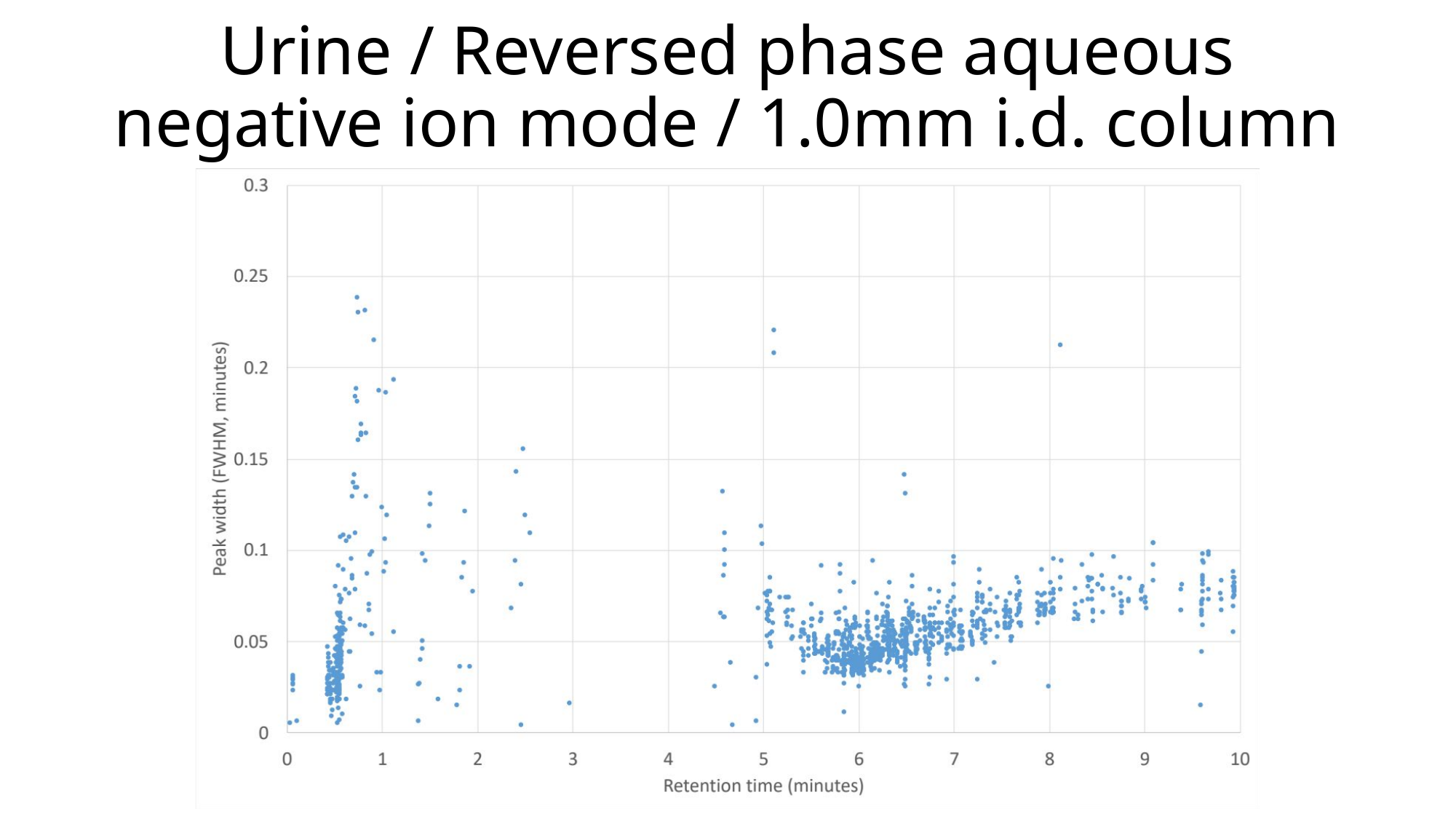

# Urine / Reversed phase aqueous negative ion mode / 1.0mm i.d. column

## Slide 97
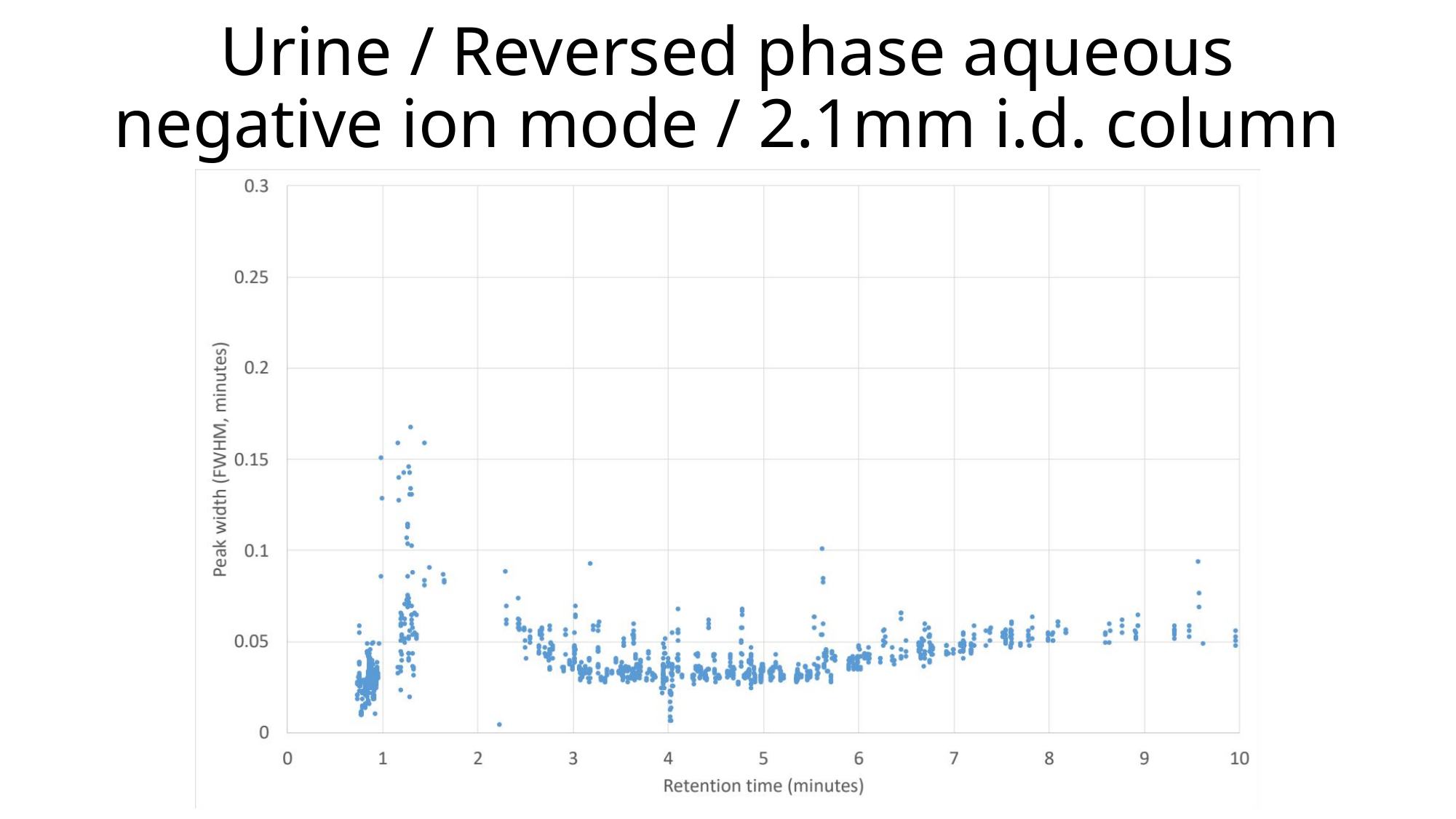

# Urine / Reversed phase aqueous negative ion mode / 2.1mm i.d. column

## Slide 98
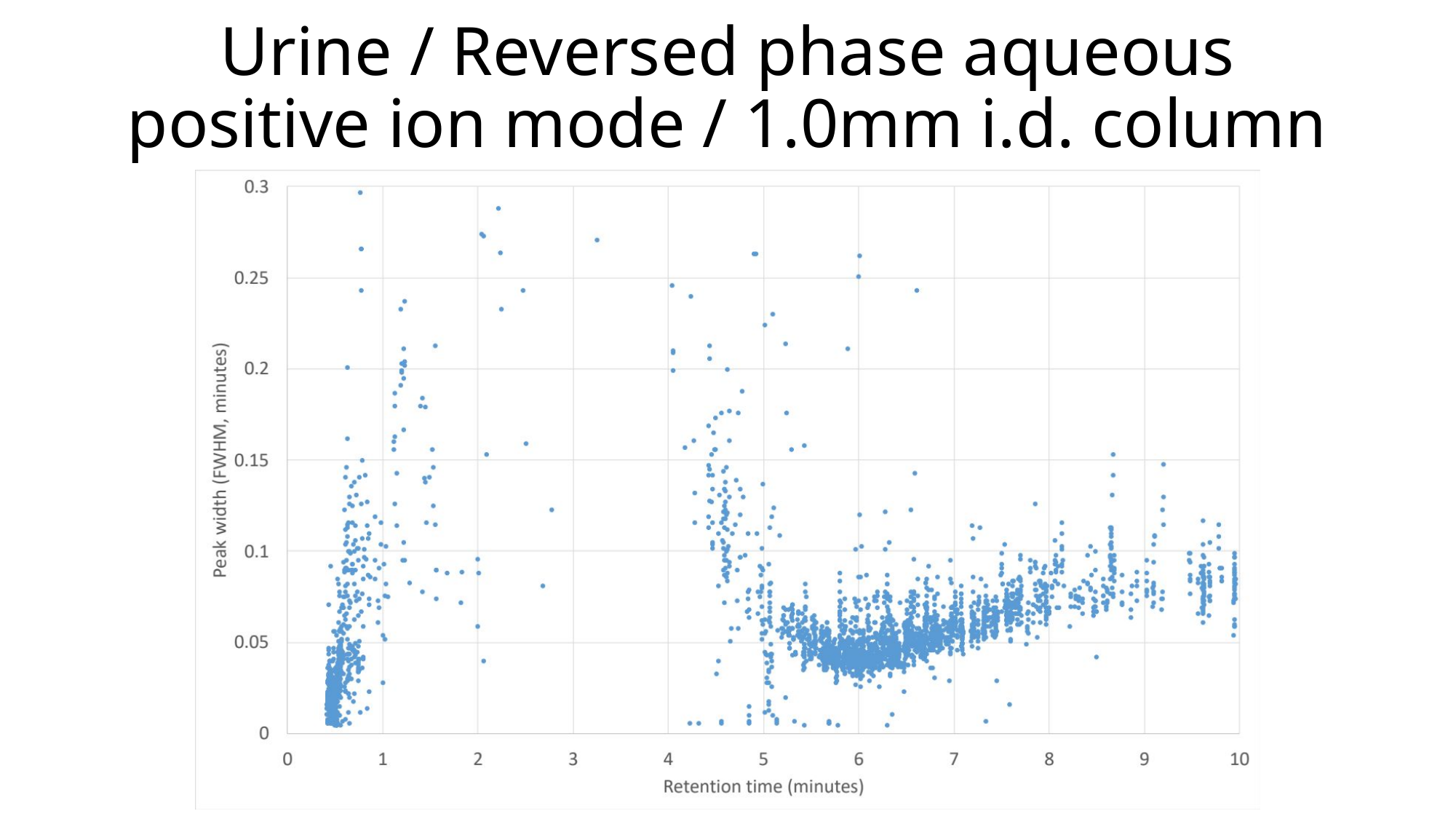

# Urine / Reversed phase aqueous positive ion mode / 1.0mm i.d. column

## Slide 99
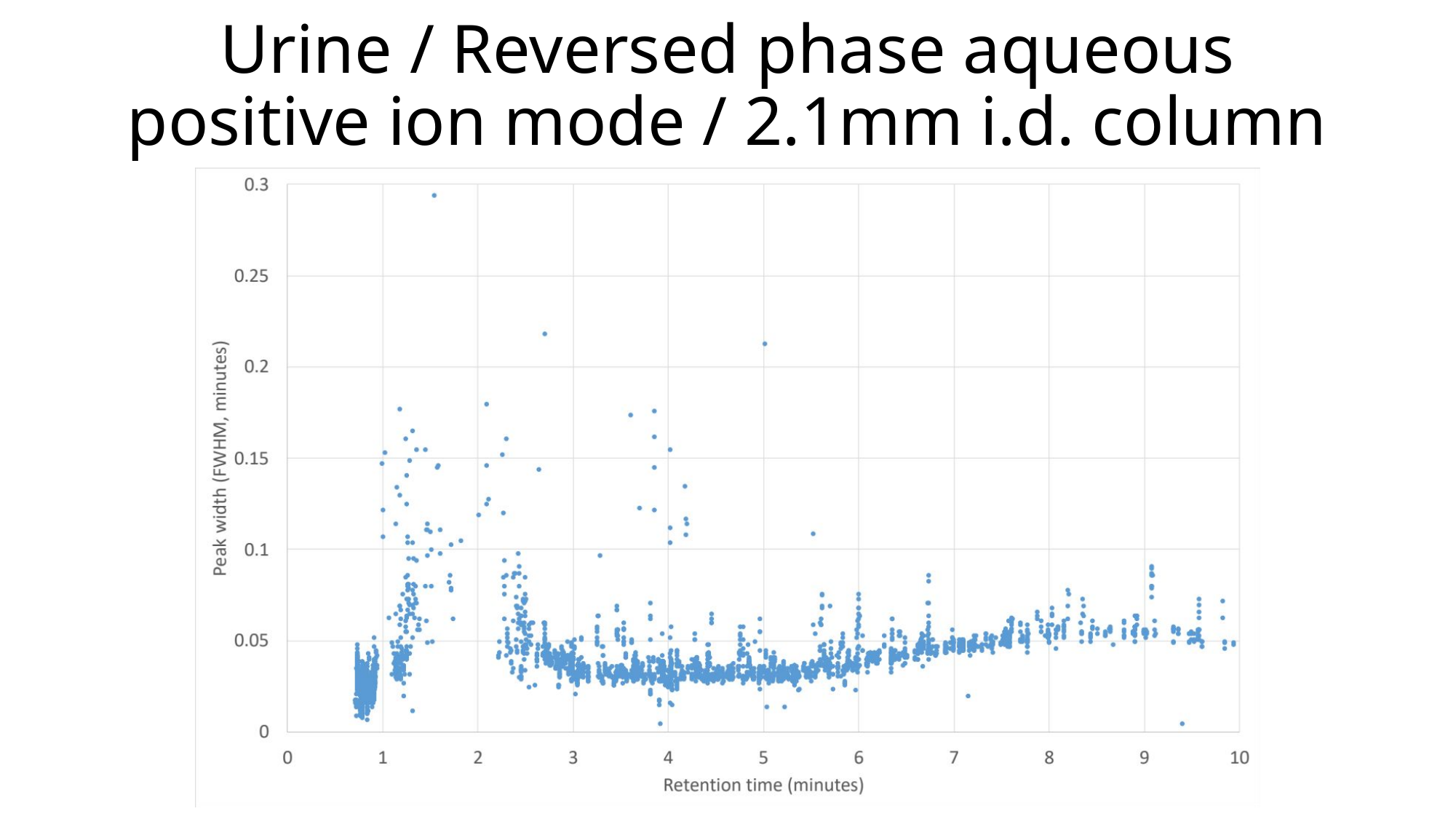

# Urine / Reversed phase aqueous positive ion mode / 2.1mm i.d. column

## Slide 100
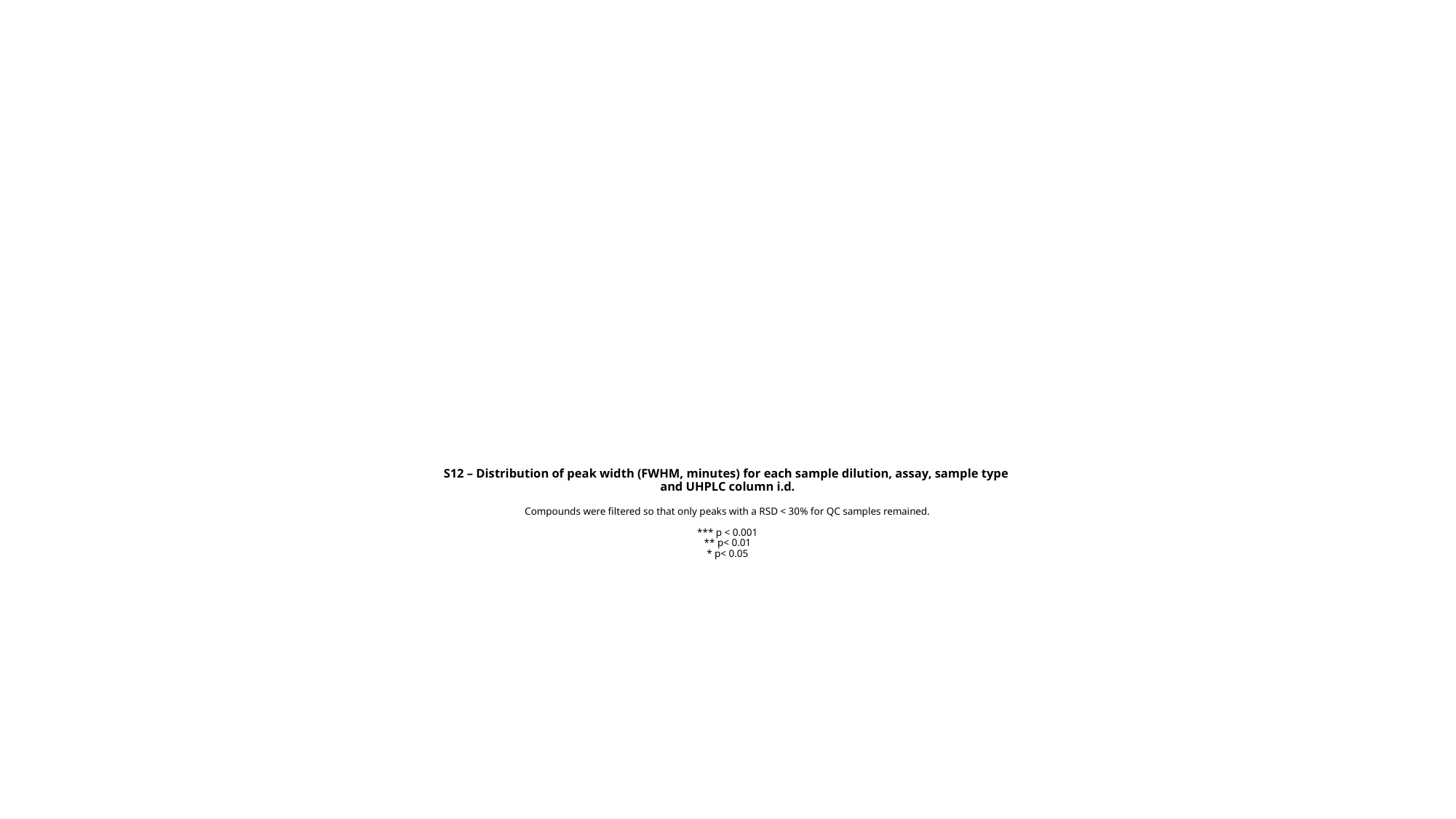

# S12 – Distribution of peak width (FWHM, minutes) for each sample dilution, assay, sample type and UHPLC column i.d. Compounds were filtered so that only peaks with a RSD < 30% for QC samples remained. *** p < 0.001 ** p< 0.01 * p< 0.05

## Slide 101
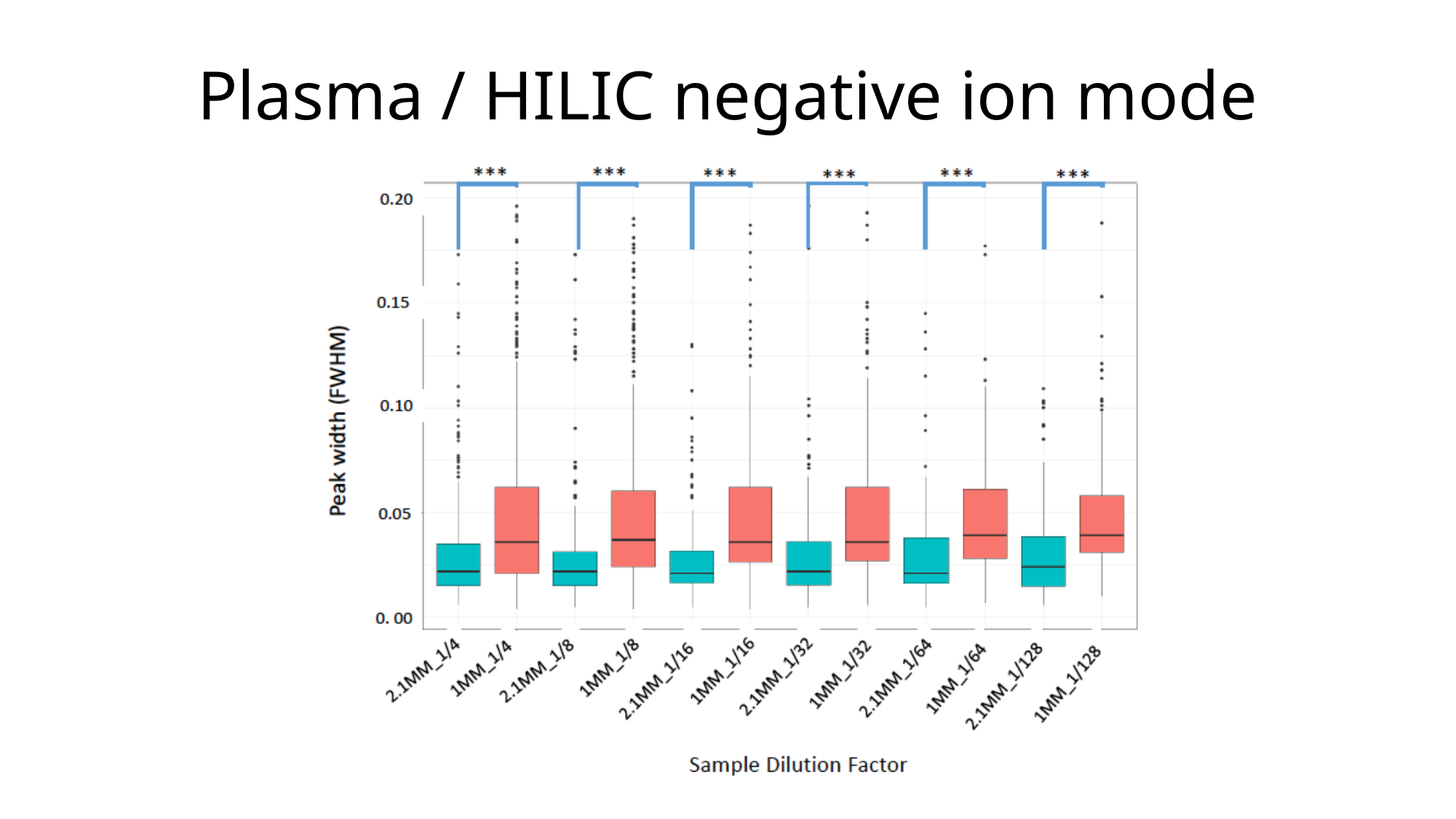

# Plasma / HILIC negative ion mode

## Slide 102
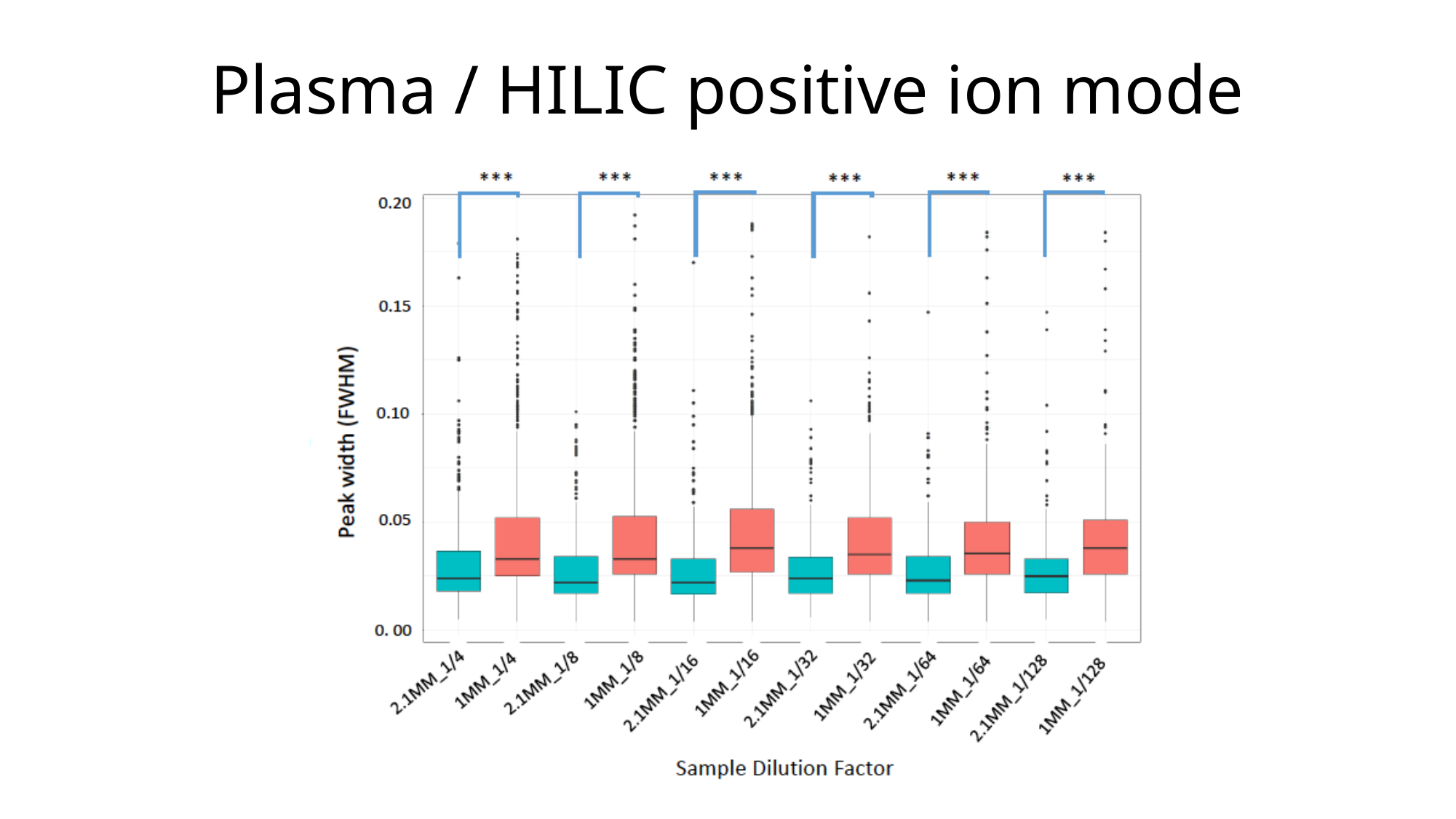

# Plasma / HILIC positive ion mode

## Slide 103
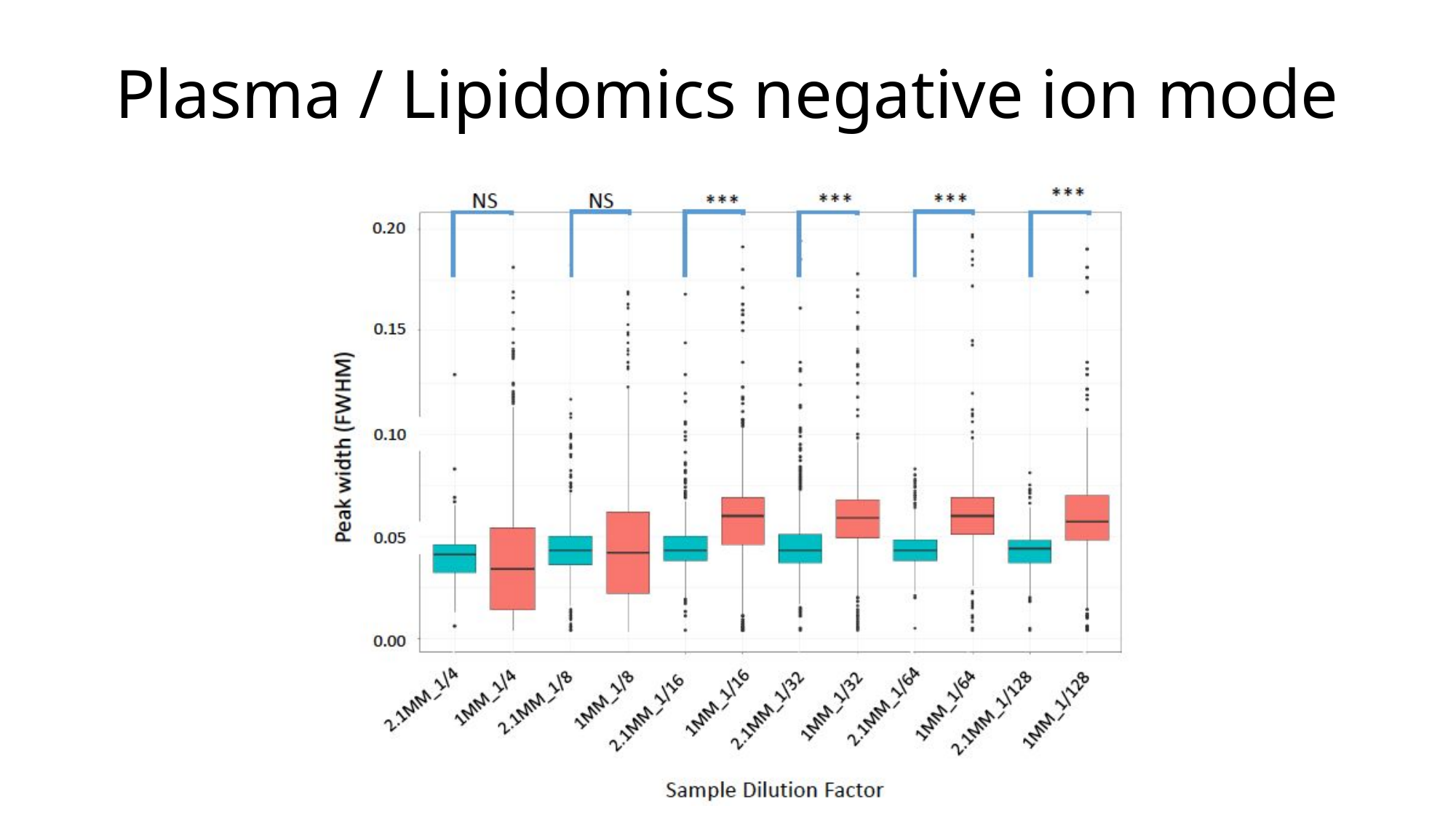

# Plasma / Lipidomics negative ion mode

## Slide 104
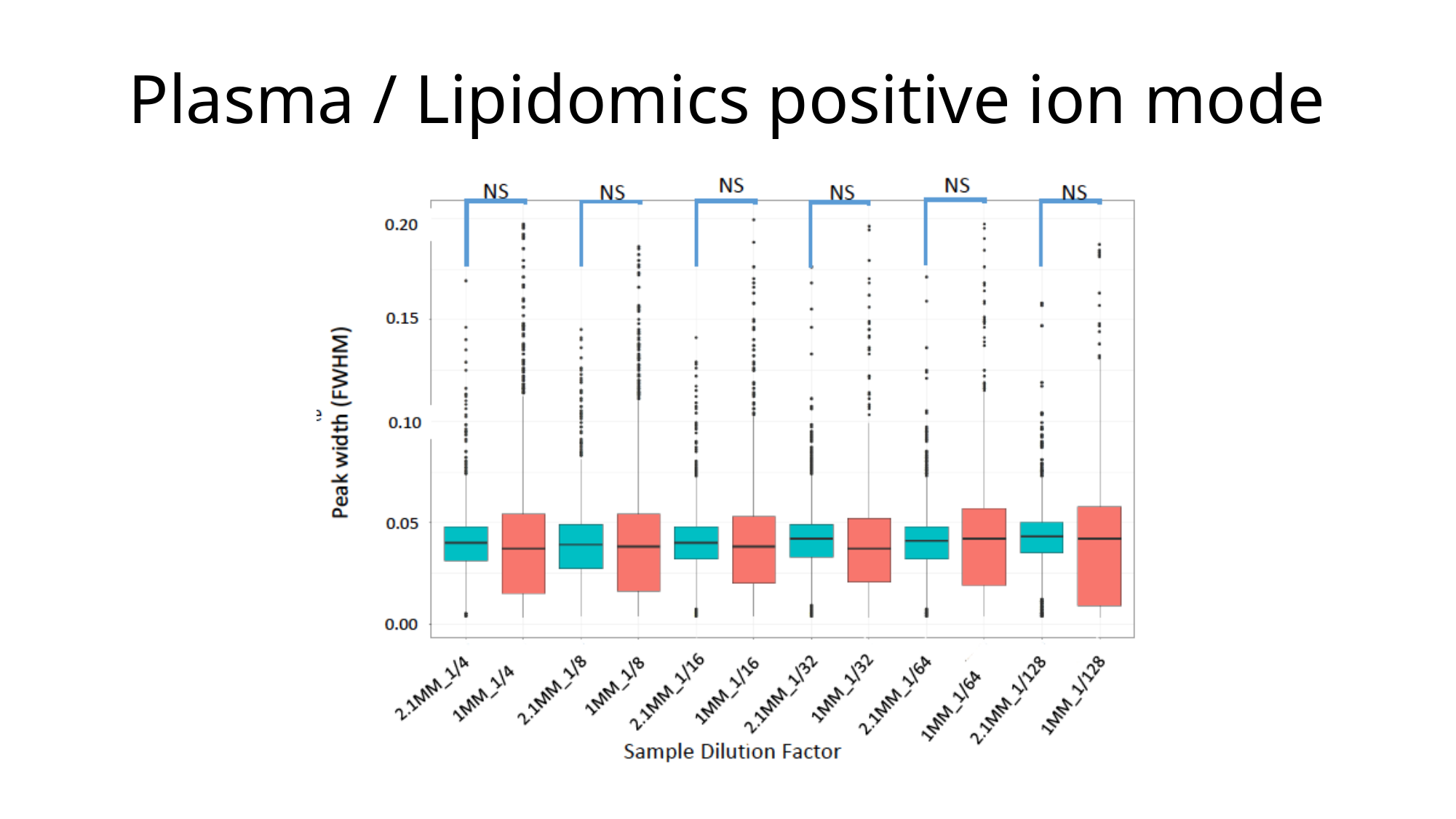

# Plasma / Lipidomics positive ion mode

## Slide 105
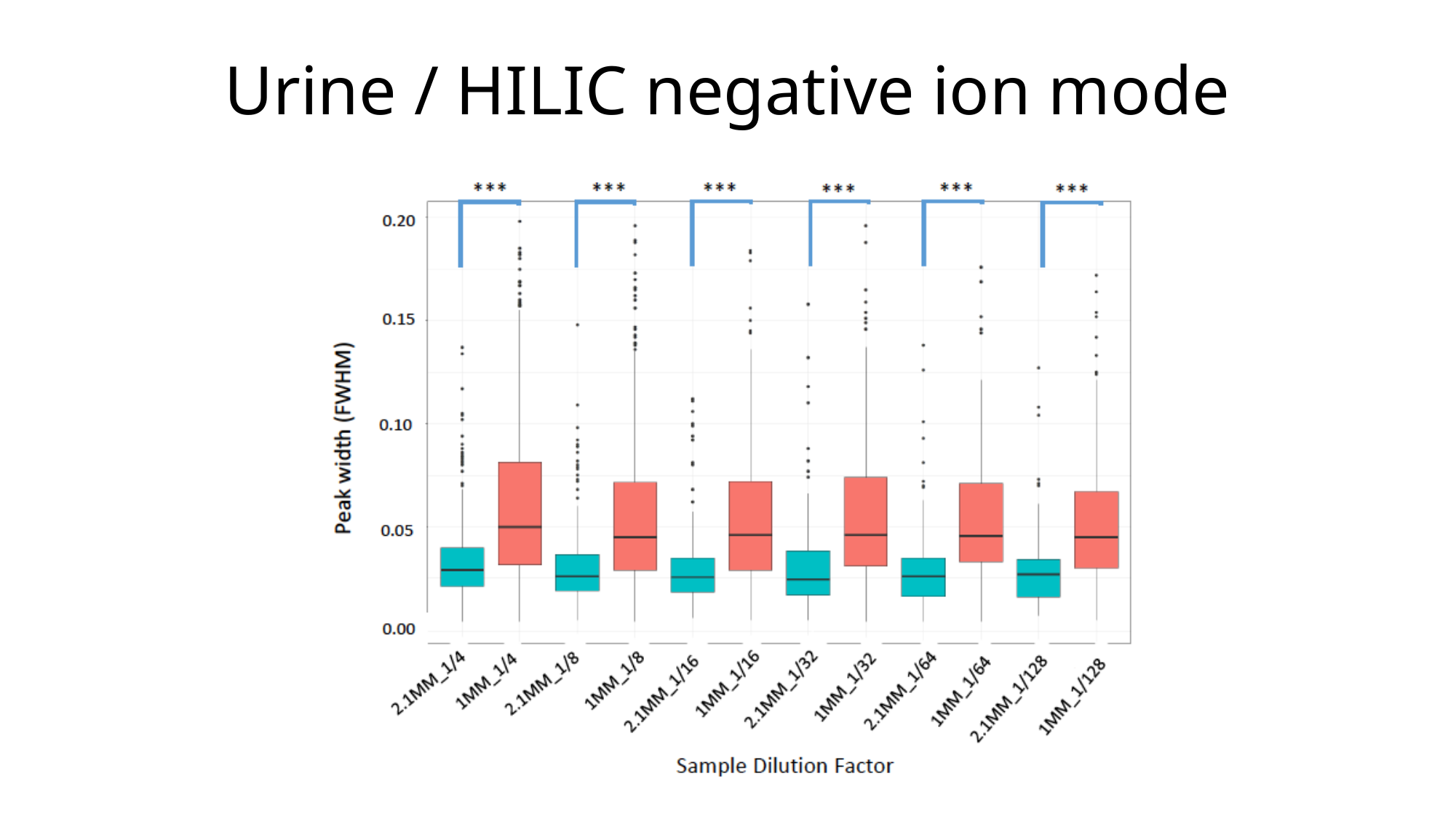

# Urine / HILIC negative ion mode

## Slide 106
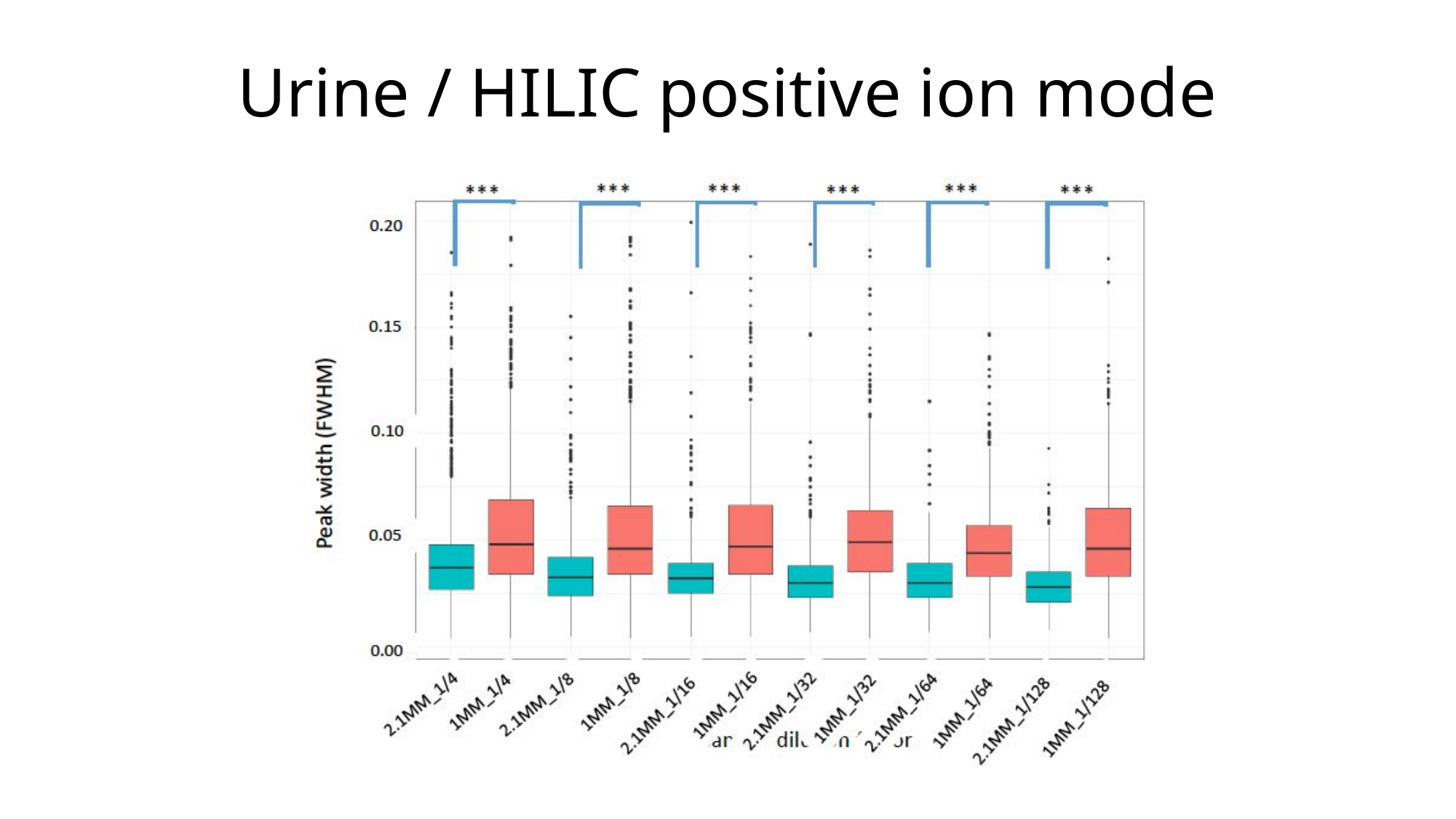

# Urine / HILIC positive ion mode

## Slide 107
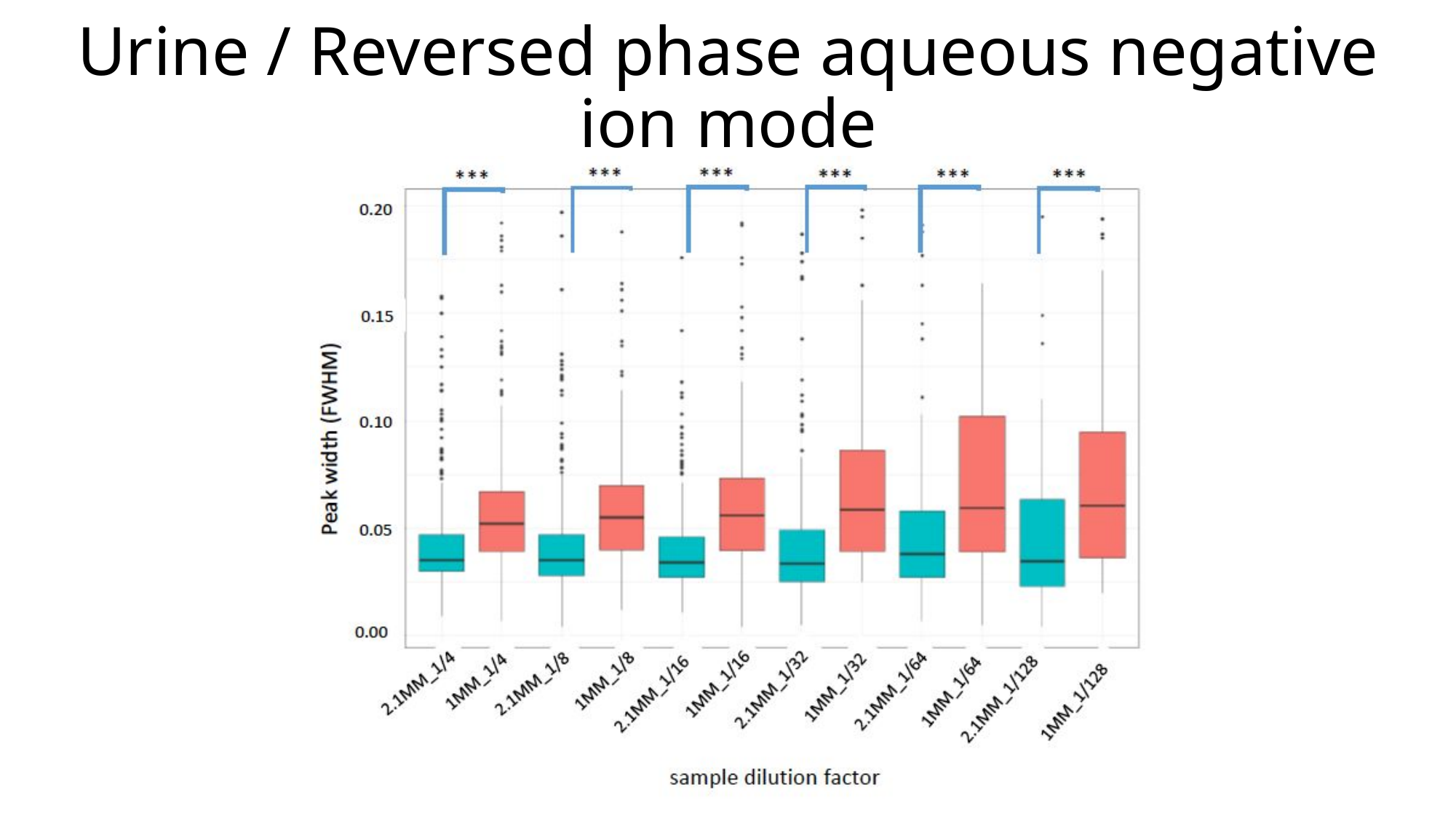

# Urine / Reversed phase aqueous negative ion mode

## Slide 108
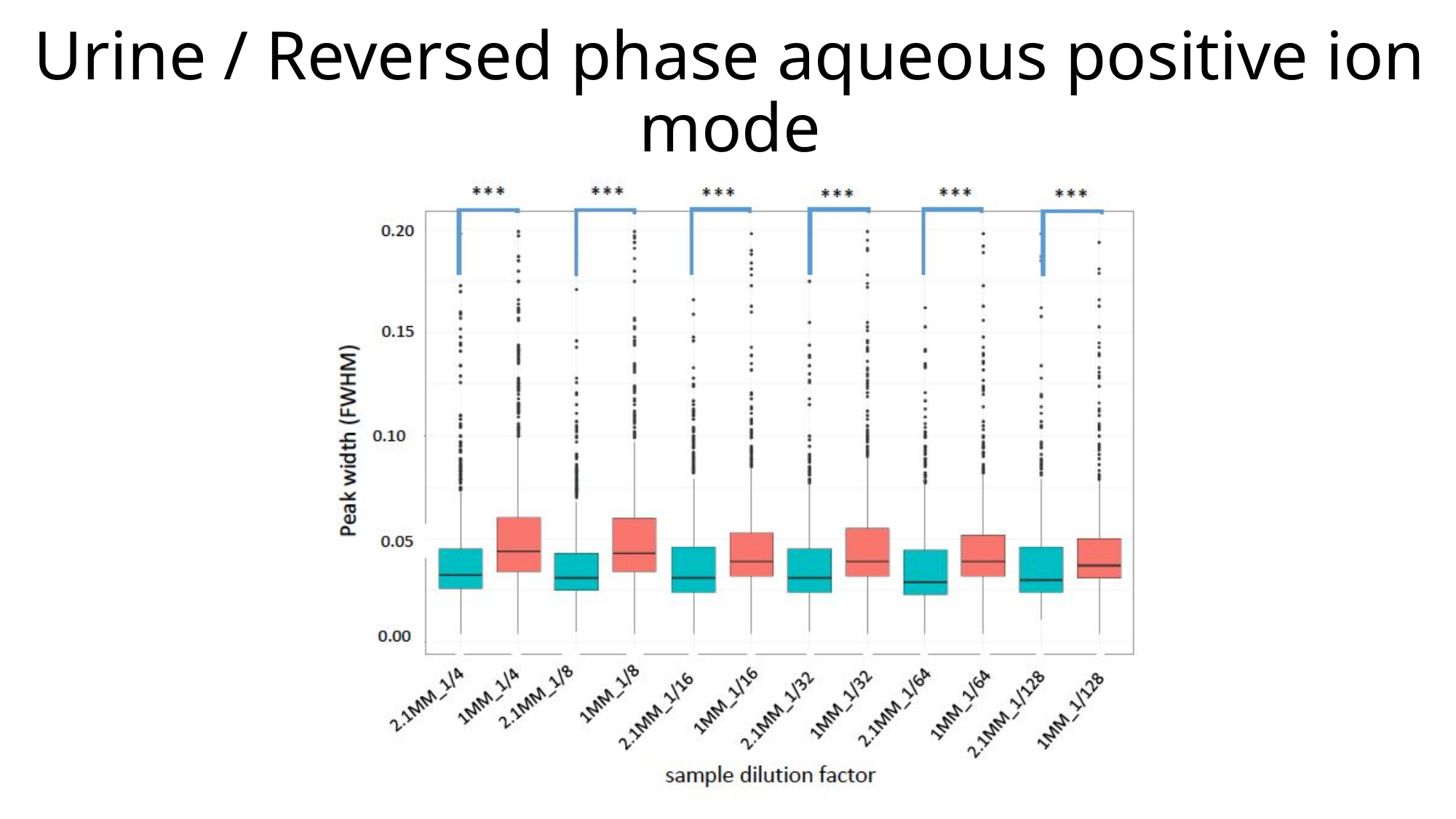

# Urine / Reversed phase aqueous positive ion mode

## Slide 109
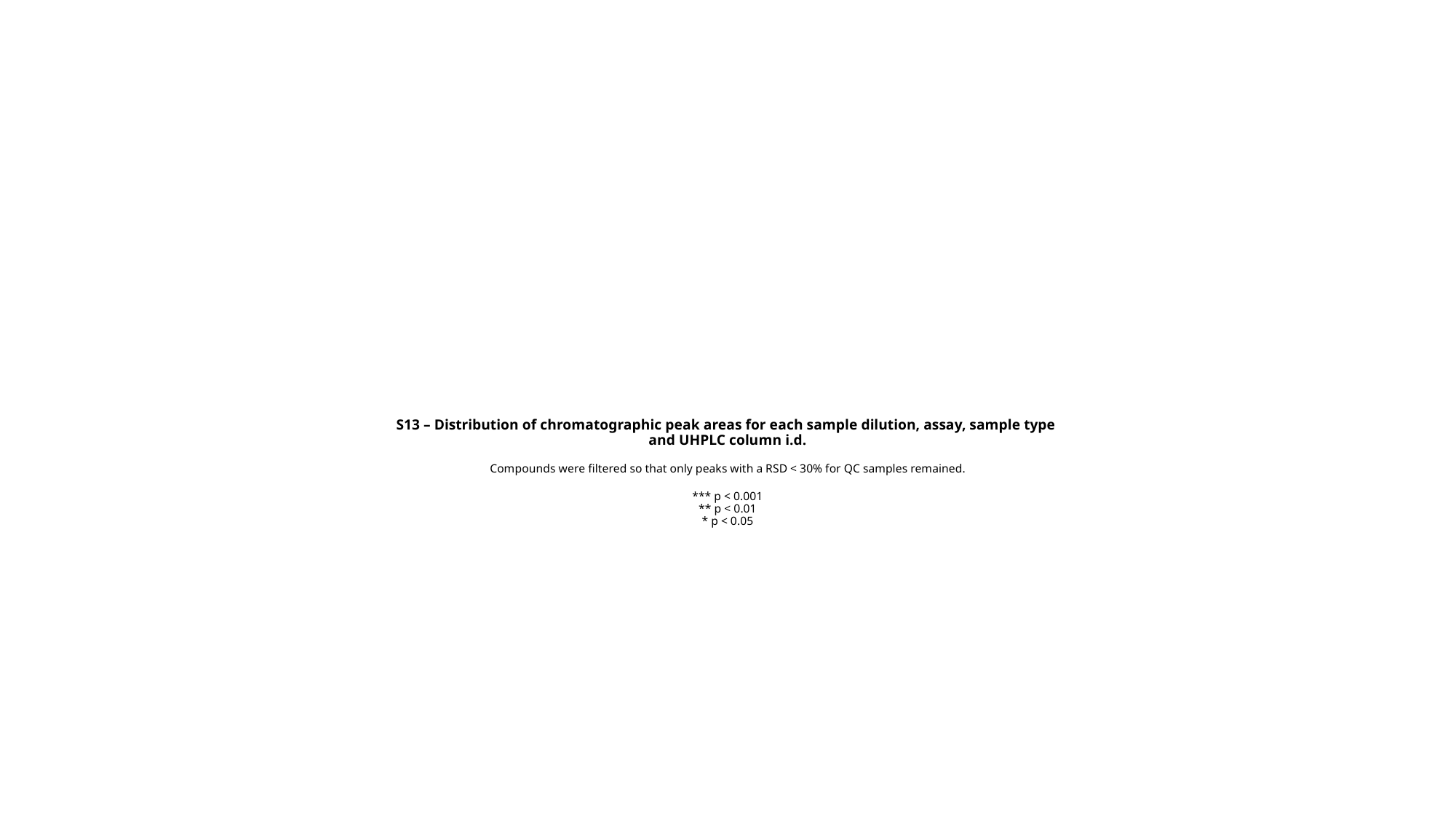

# S13 – Distribution of chromatographic peak areas for each sample dilution, assay, sample type and UHPLC column i.d. Compounds were filtered so that only peaks with a RSD < 30% for QC samples remained. *** p < 0.001 ** p < 0.01 * p < 0.05

## Slide 110
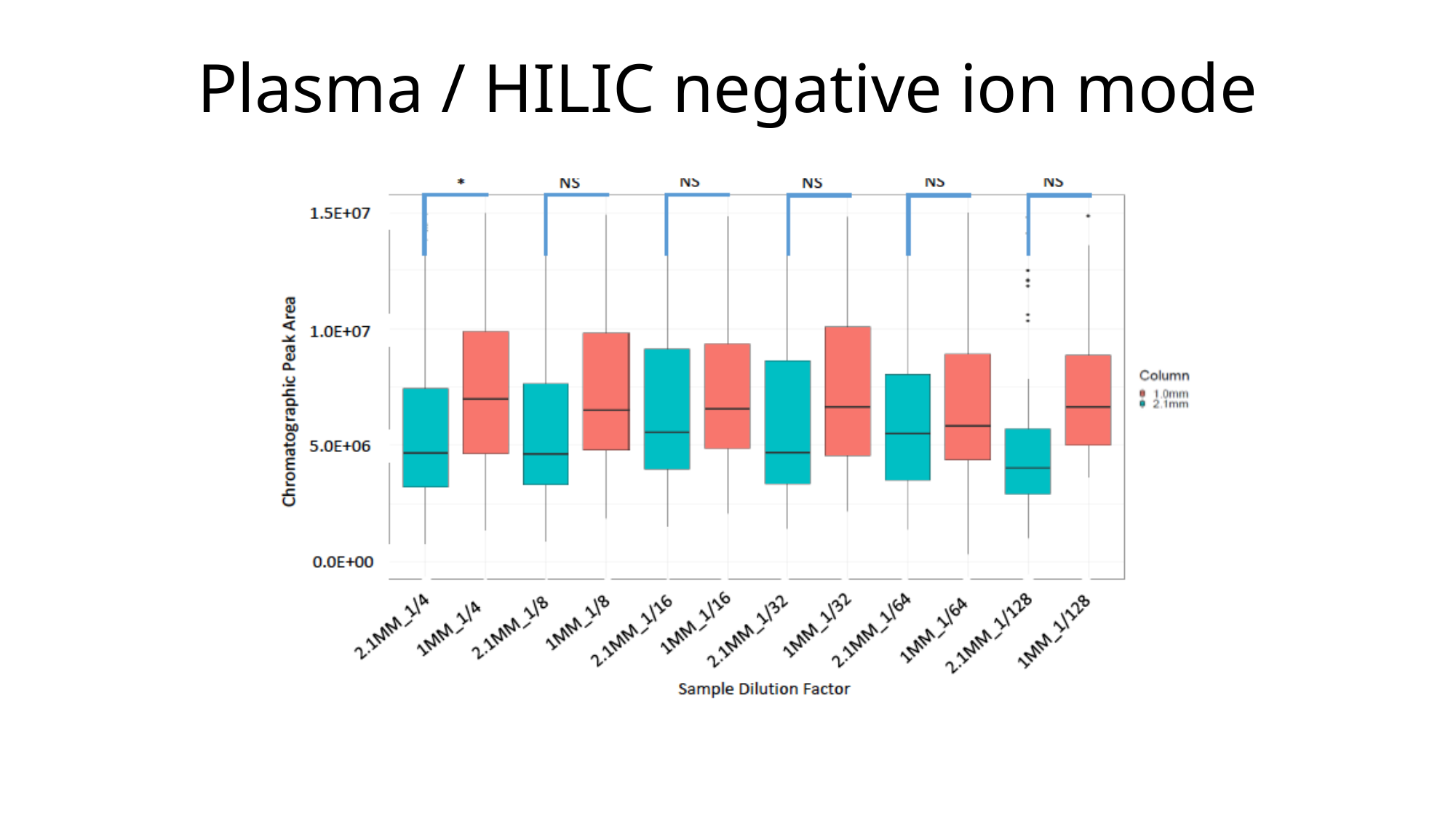

# Plasma / HILIC negative ion mode

## Slide 111
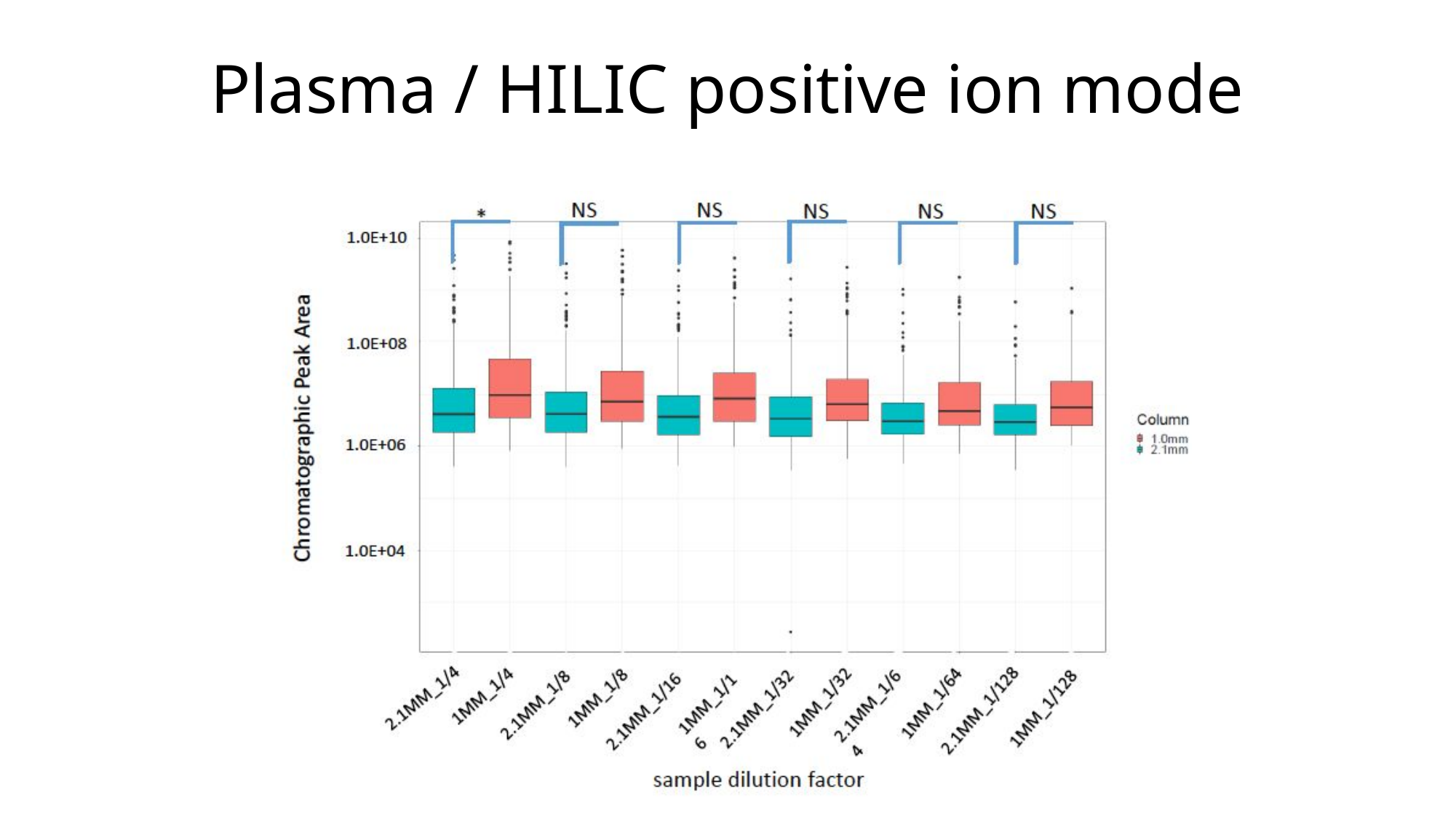

# Plasma / HILIC positive ion mode

## Slide 112
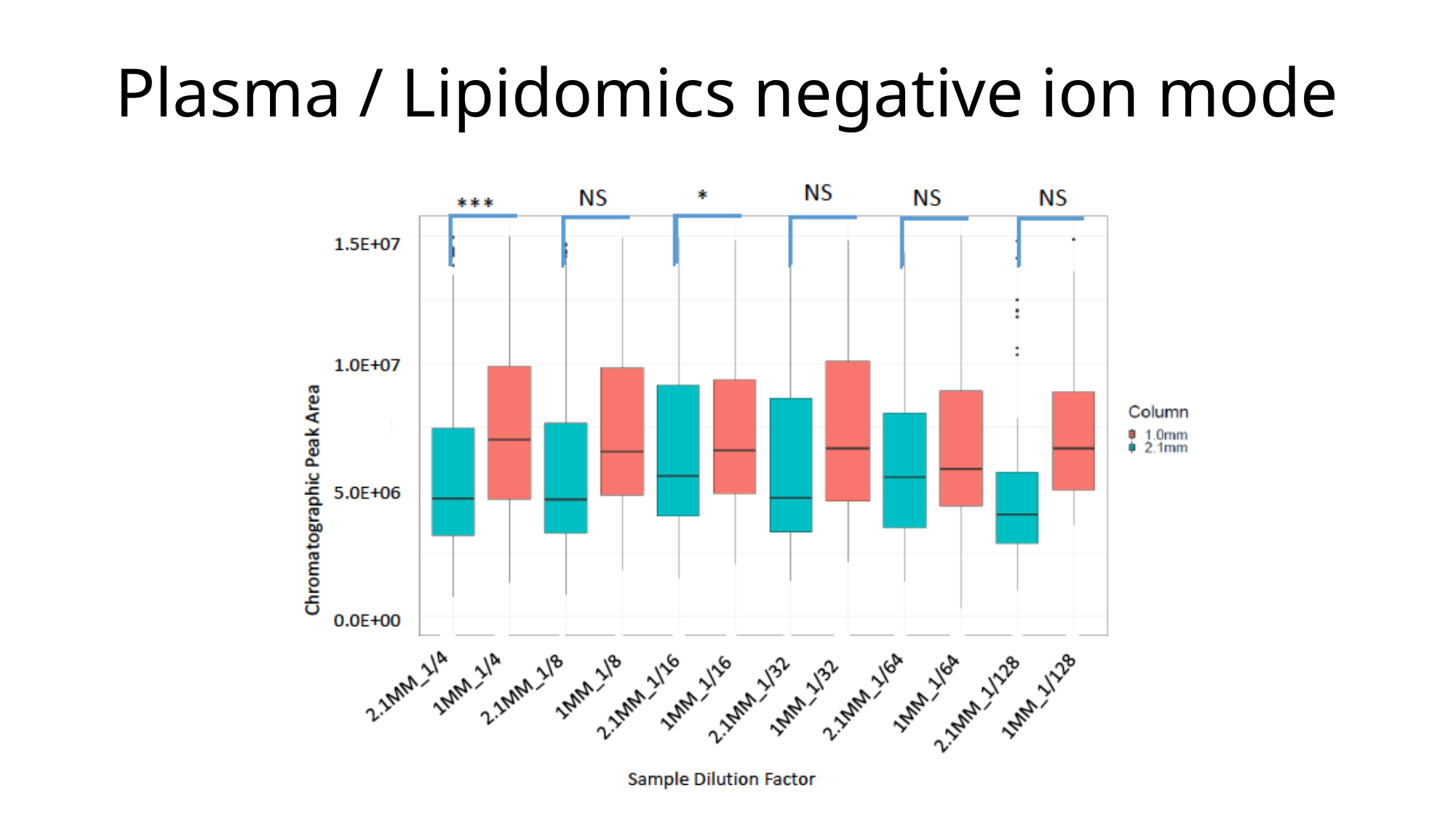

# Plasma / Lipidomics negative ion mode

## Slide 113
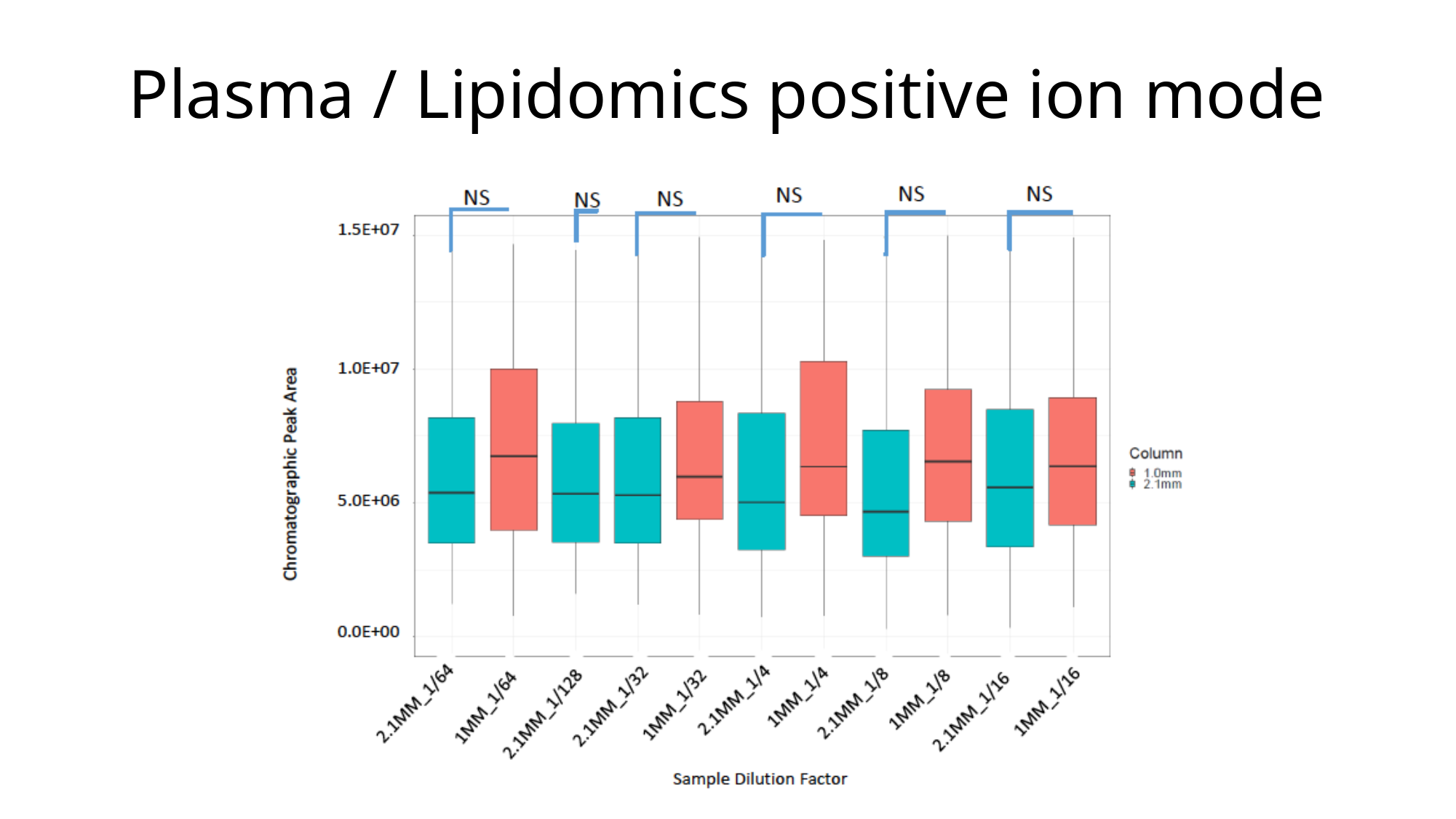

# Plasma / Lipidomics positive ion mode

## Slide 114
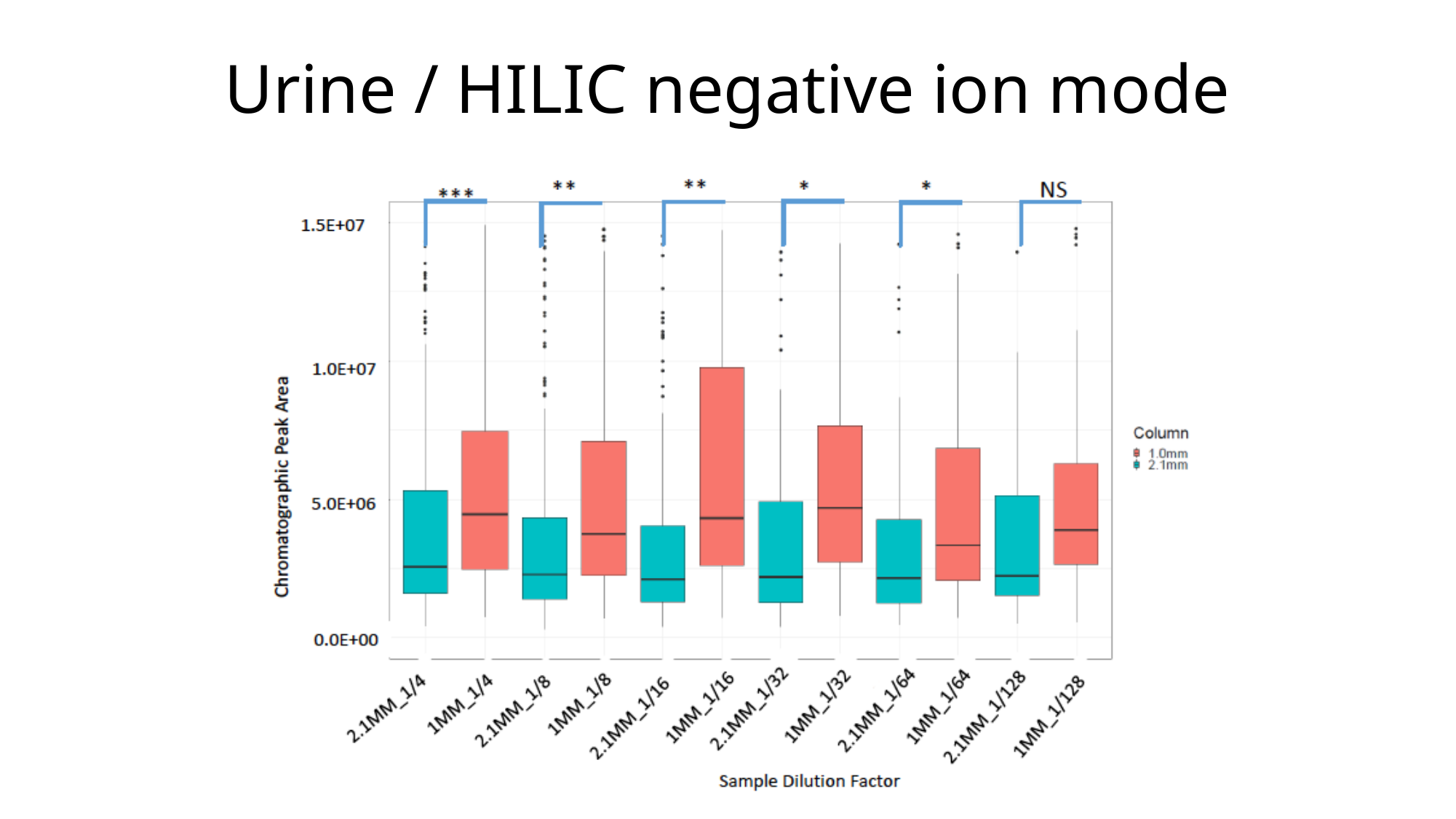

# Urine / HILIC negative ion mode

## Slide 115
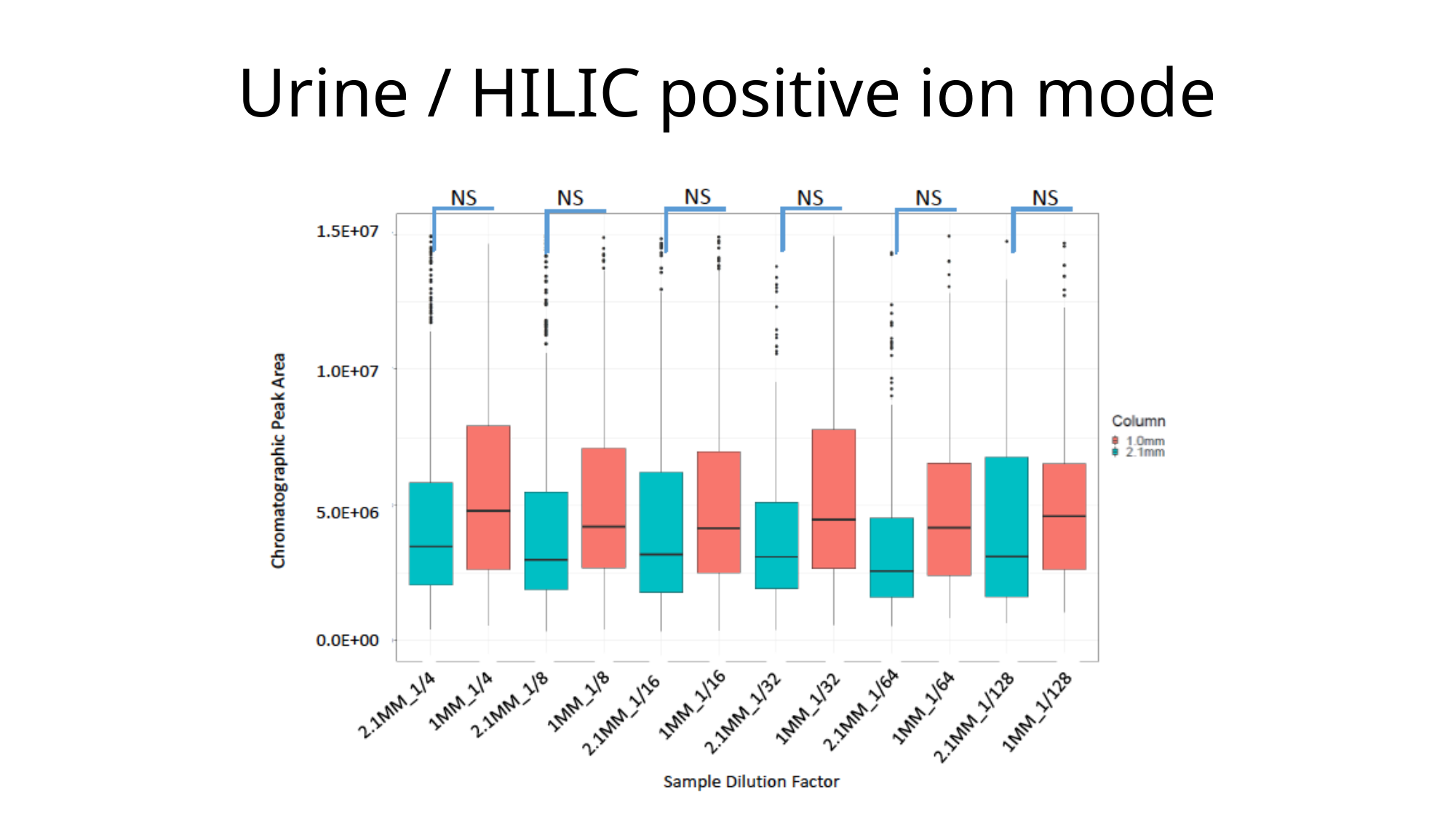

# Urine / HILIC positive ion mode

## Slide 116
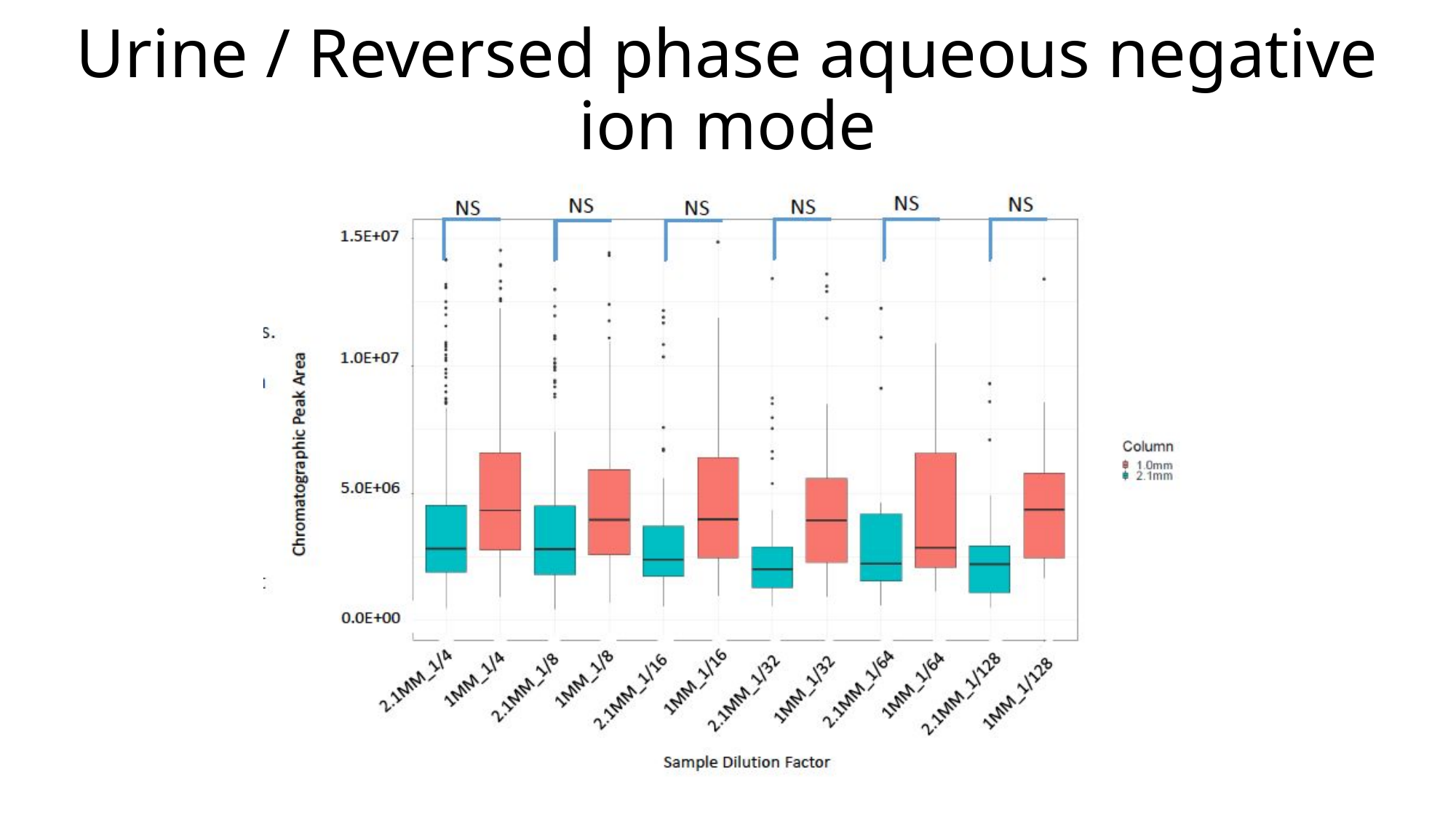

# Urine / Reversed phase aqueous negative ion mode

## Slide 117
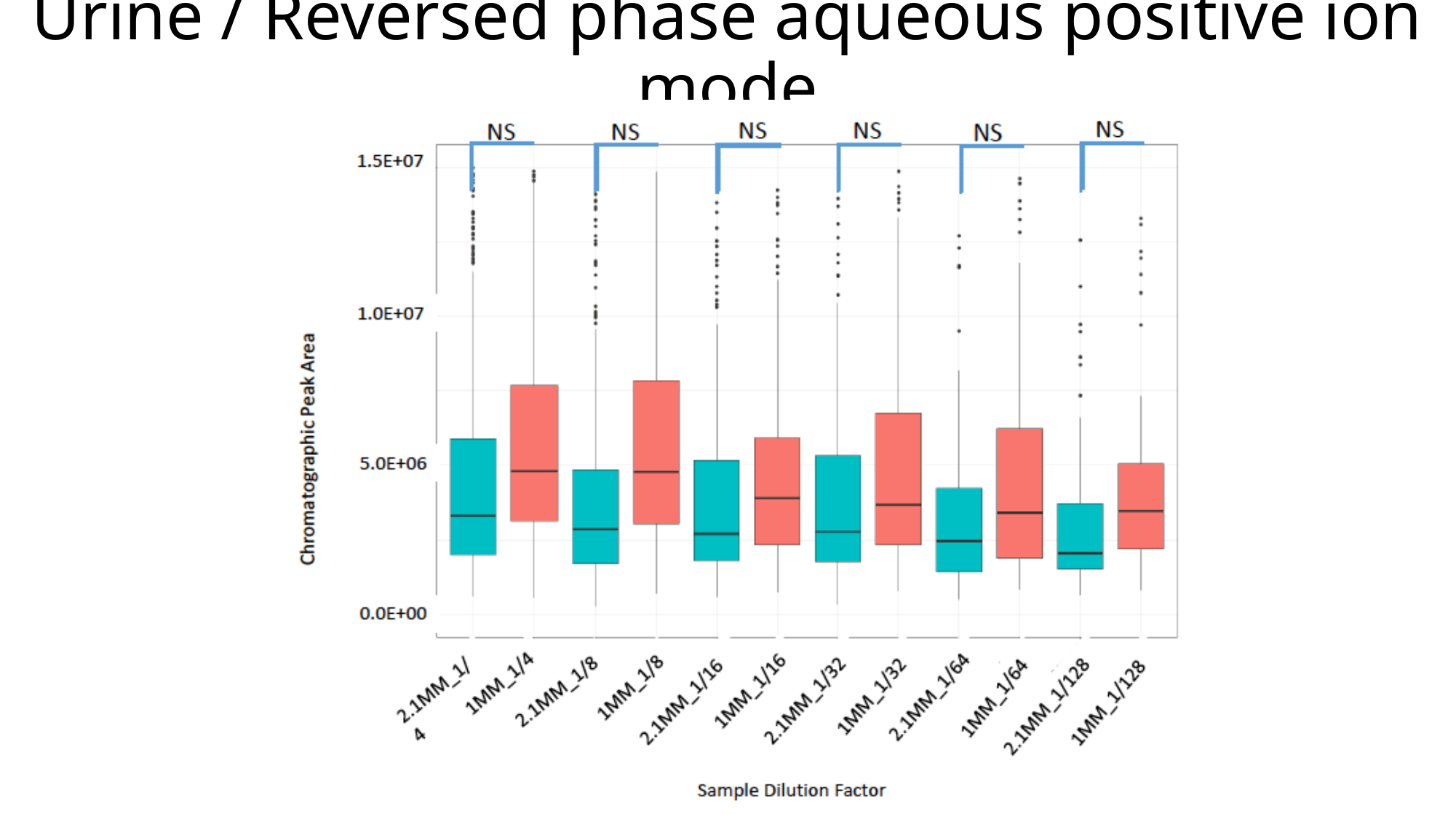

# Urine / Reversed phase aqueous positive ion mode

## Slide 118
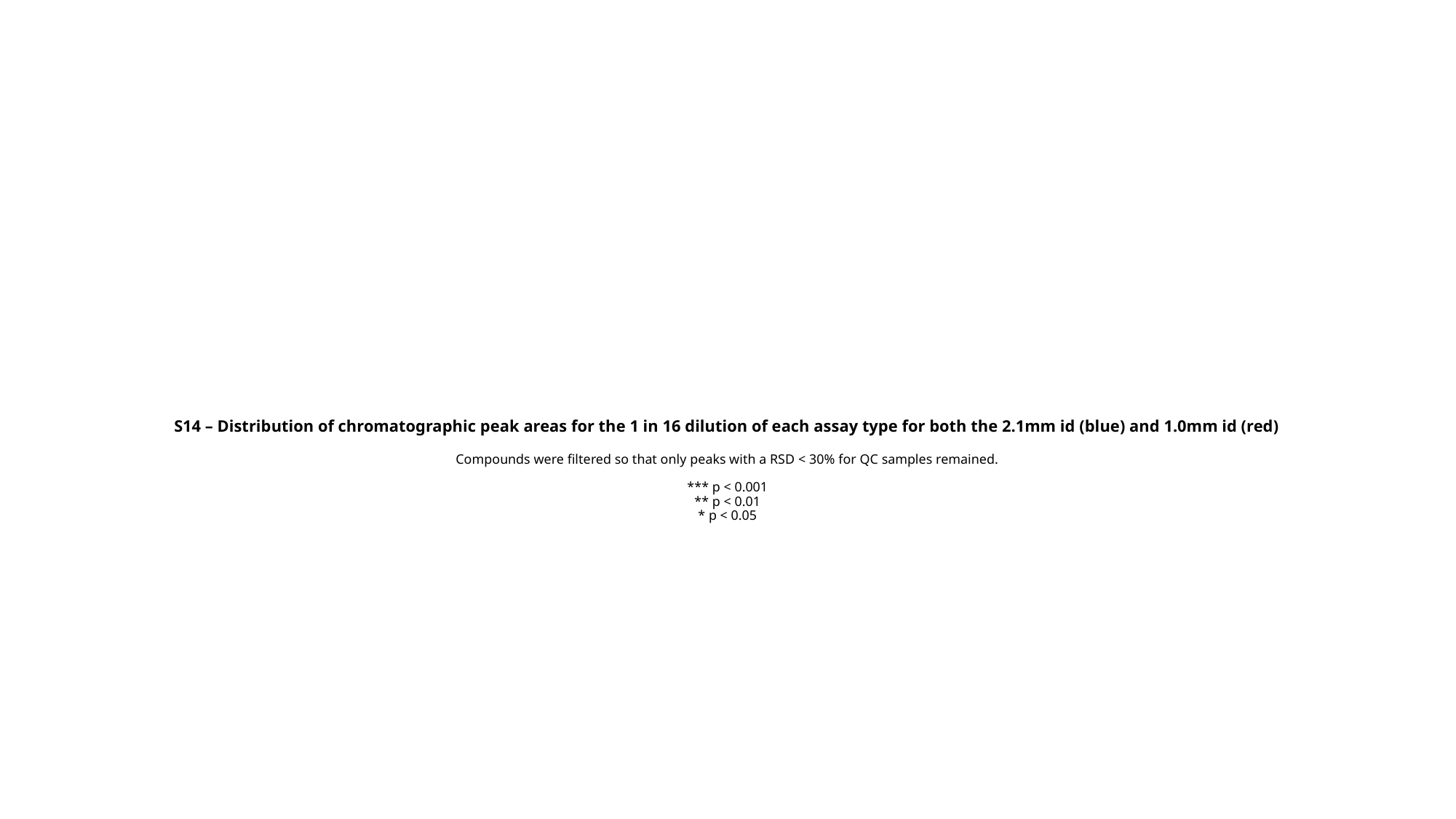

# S14 – Distribution of chromatographic peak areas for the 1 in 16 dilution of each assay type for both the 2.1mm id (blue) and 1.0mm id (red) Compounds were filtered so that only peaks with a RSD < 30% for QC samples remained. *** p < 0.001 ** p < 0.01 * p < 0.05

## Slide 119
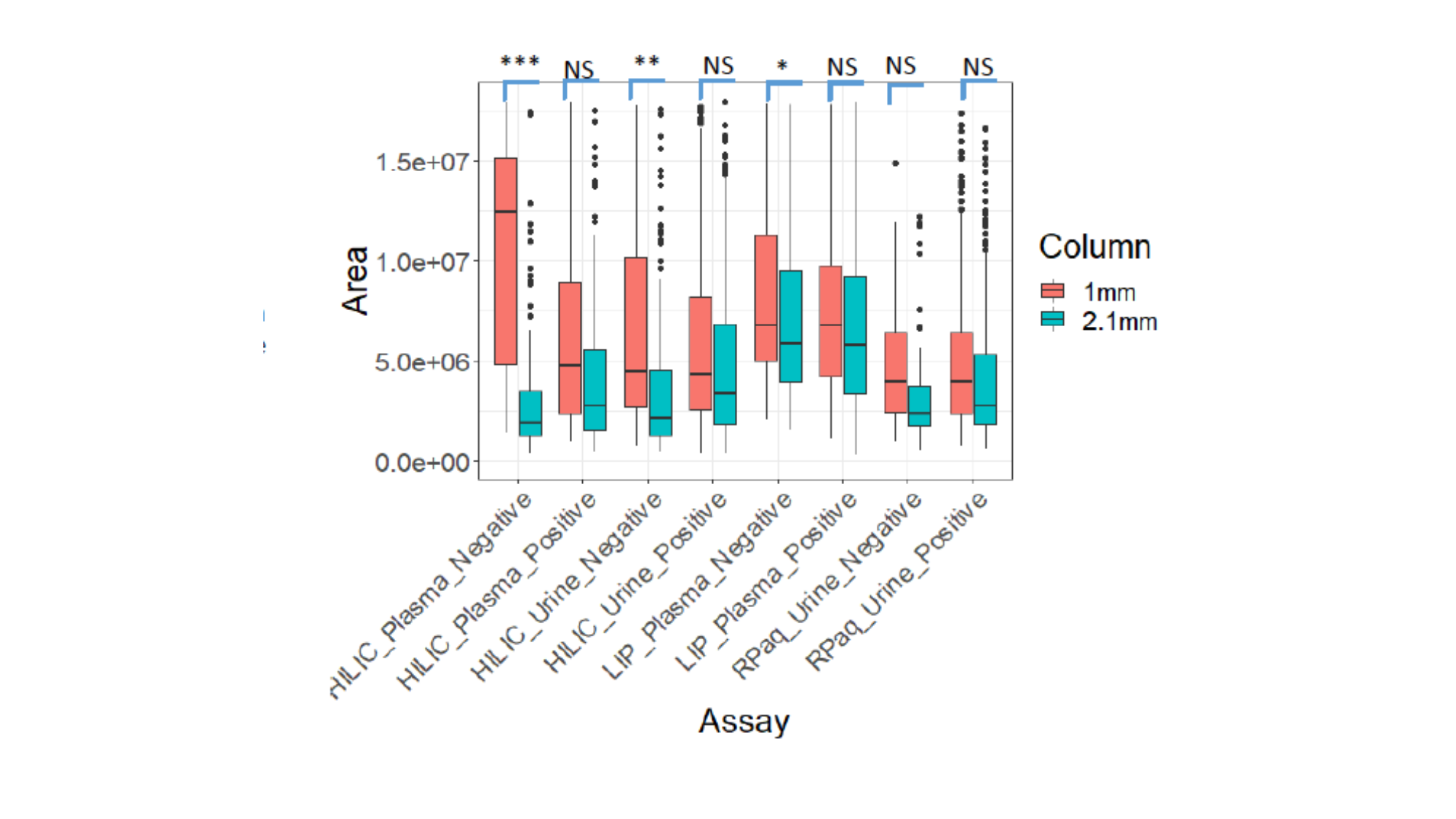

## Slide 120
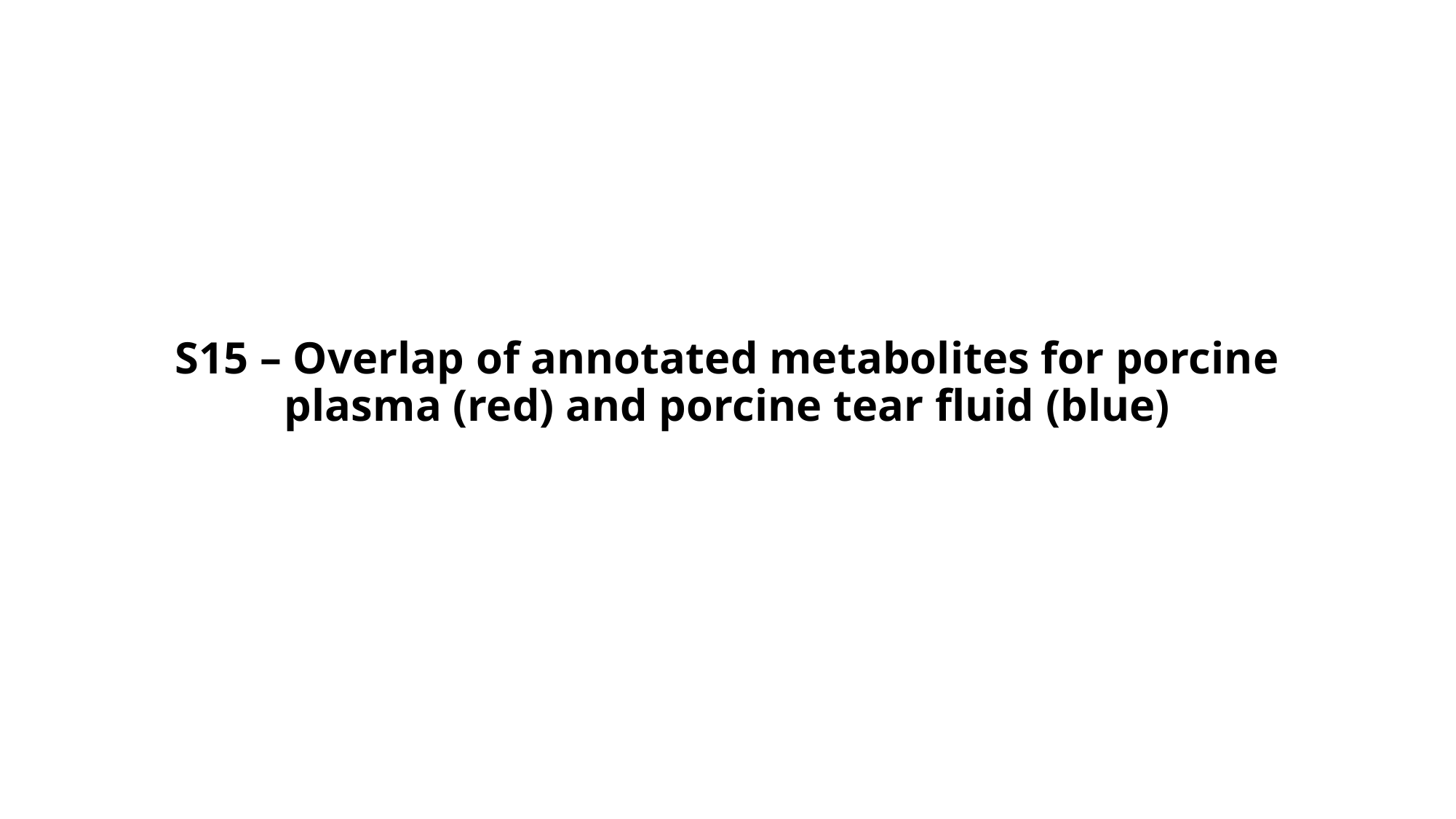

# S15 – Overlap of annotated metabolites for porcine plasma (red) and porcine tear fluid (blue)

## Slide 121
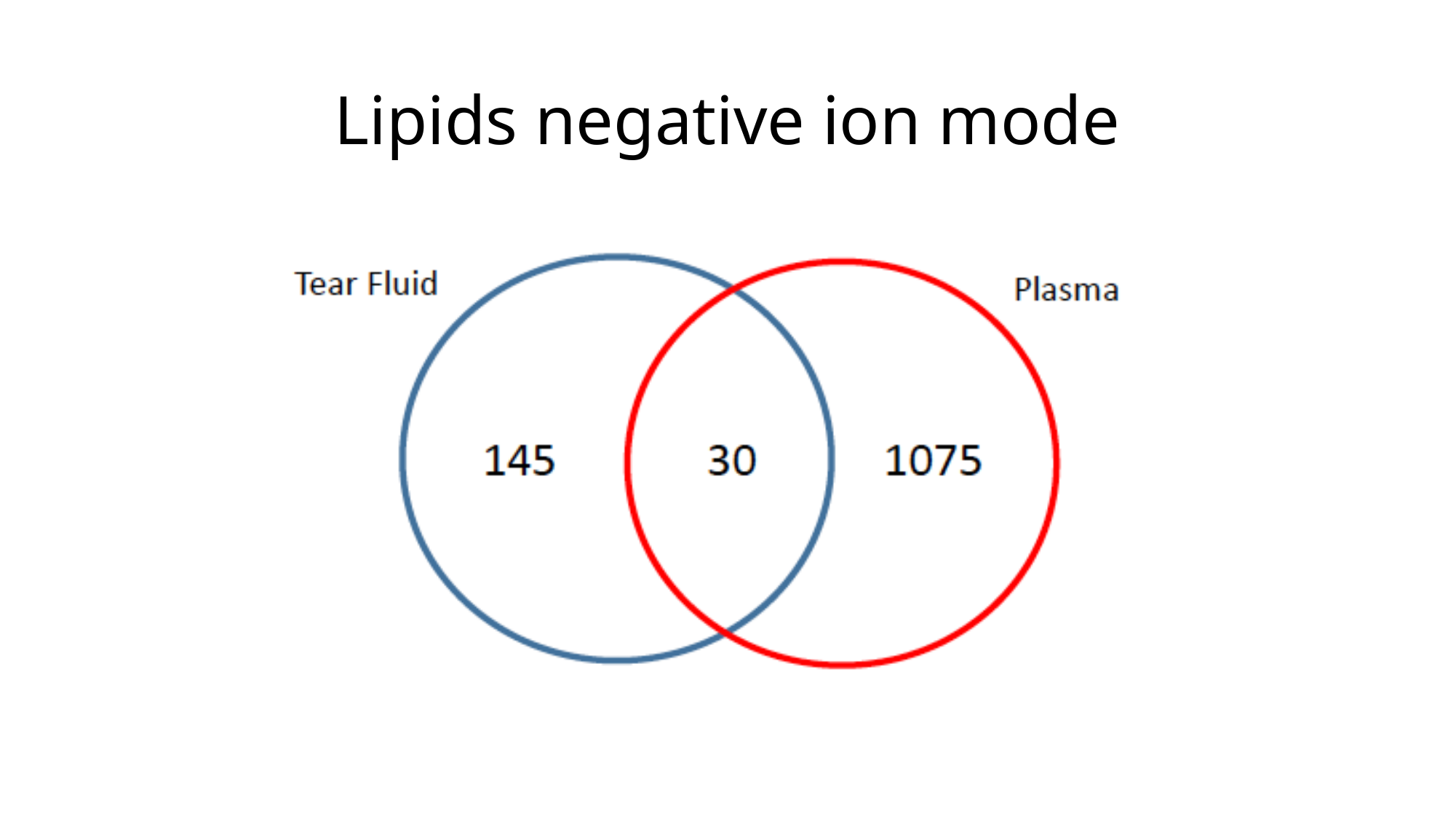

# Lipids negative ion mode

## Slide 122
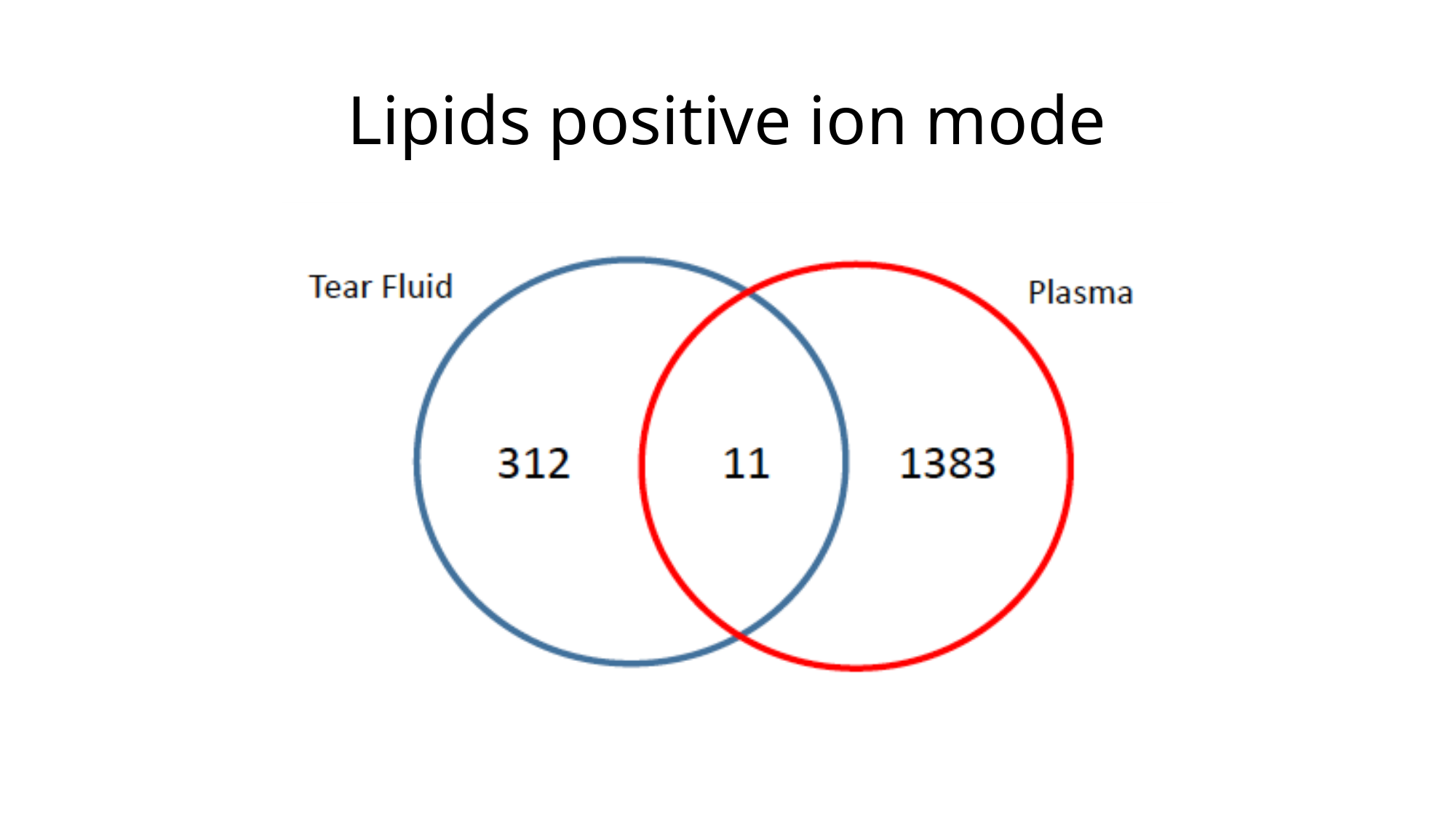

# Lipids positive ion mode

## Slide 123
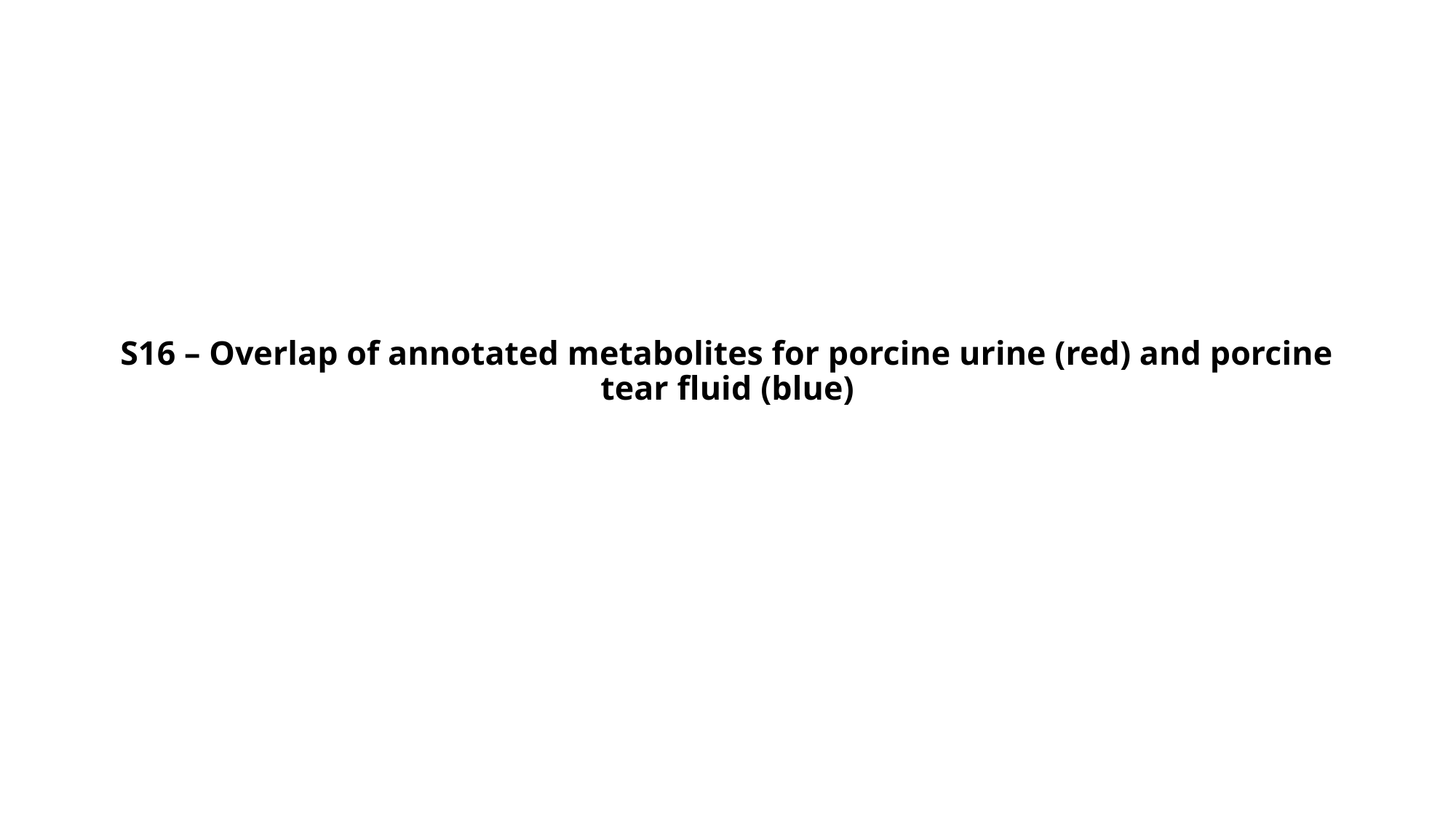

# S16 – Overlap of annotated metabolites for porcine urine (red) and porcine tear fluid (blue)

## Slide 124
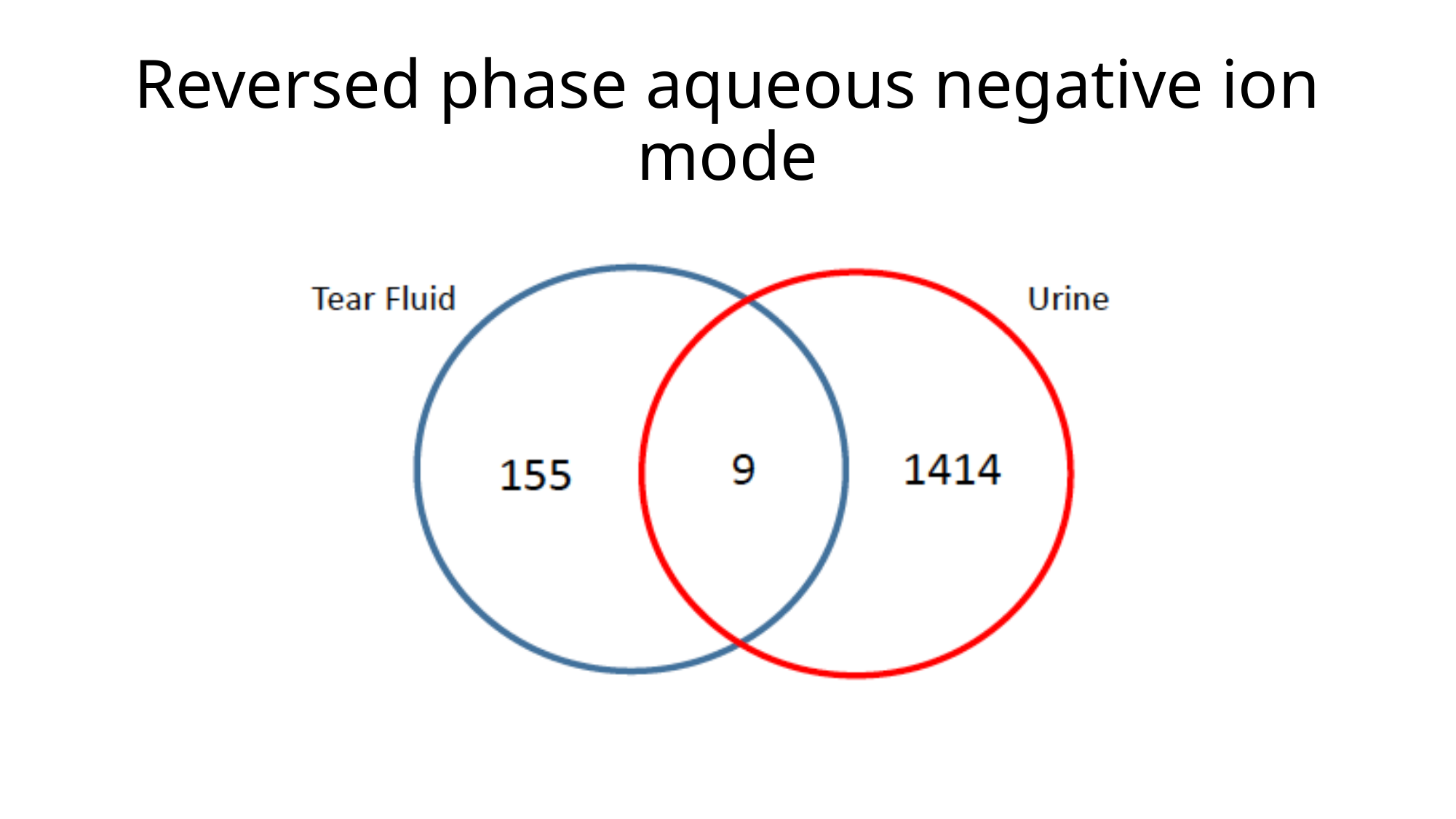

# Reversed phase aqueous negative ion mode

## Slide 125
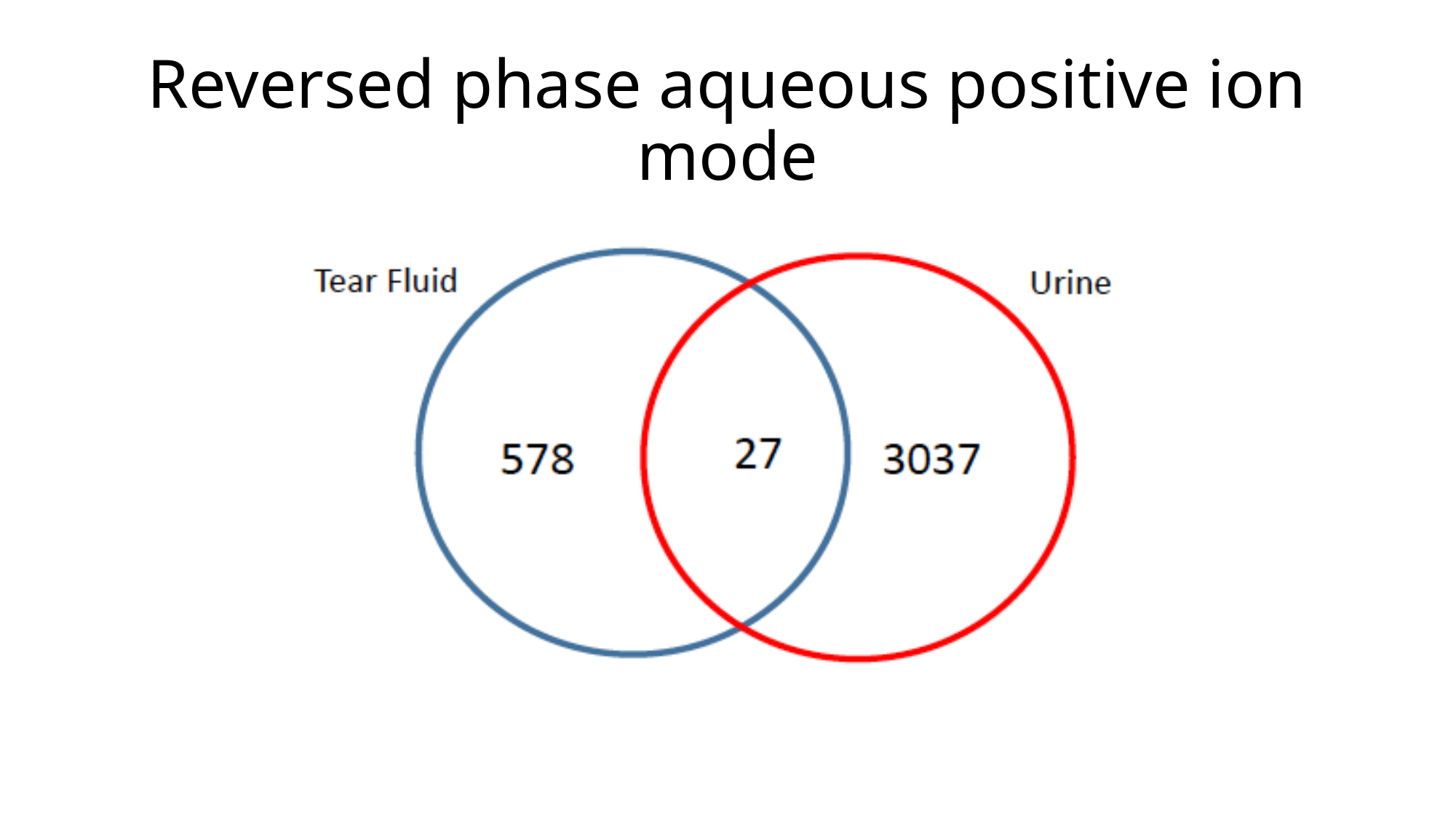

# Reversed phase aqueous positive ion mode
